# The Effect of Non-Nutritive Sweetened Beverages on Postprandial Glycemic and Endocrine Responses: A Systematic Review and Network Meta-Analysis

**DOI:** 10.3390/nu15041050

**Published:** 2023-02-20

**Authors:** Roselyn Zhang, Jarvis C. Noronha, Tauseef A. Khan, Néma McGlynn, Songhee Back, Shannan M. Grant, Cyril W. C. Kendall, John L. Sievenpiper

**Affiliations:** 1 Toronto 3D Knowledge Synthesis and Clinical Trials Unit, Clinical Nutrition and Risk Factor Modification Centre, St. Michael’s Hospital, Toronto, ON M5C 2T2, Canada; 2 Department of Applied Human Nutrition, Mount Saint Vincent University, Halifax, NS B3M 2J6, Canada; 3 Department of Applied Health Sciences, University of Waterloo, Waterloo, ON N2L 3G5, Canada; 4 School of Medicine, Faculty of Medicine, The University of Queensland, Brisbane, QLD 4006, Australia; 5 Department of Nutritional Sciences, Temerty Faculty of Medicine, University of Toronto, Toronto, ON M5S 1A8, Canada; 6 Department of Pediatrics, Dalhousie University, Halifax, NS B3H 4R2, Canada; 7 Department of Obstetrics and Gynecology, Dalhousie University, Halifax, NS B3H 4R2, Canada; 8 Department of Obstetrics & Gynecology and Department of Pediatrics, IWK Health Centre, Halifax, NS B3K 6R8, Canada; 9 College of Pharmacy and Nutrition, University of Saskatchewan, Saskatoon, SK S7N 5E5, Canada; 10 Division of Endocrinology and Metabolism, Department of Medicine, St. Michael’s Hospital, Toronto, ON M5C 2T2, Canada; 11 Department of Medicine, Temerty Faculty of Medicine, University of Toronto, Toronto, ON M5S 1A8, Canada; 12 Li Ka Shing Knowledge Institute, St. Michael’s Hospital, Toronto, ON M5B 1T8, Canada

**Keywords:** non-nutritive sweetened beverages, sugar-sweetened beverages, postprandial, glucose, insulin, GLP-1, GIP, PYY, ghrelin, glucagon, network meta-analysis

## Abstract

**Background:** There has been an emerging concern that non-nutritive sweeteners (NNS) can increase the risk of cardiometabolic disease. Much of the attention has focused on acute metabolic and endocrine responses to NNS. To examine whether these mechanisms are operational under real-world scenarios, we conducted a systematic review and network meta-analysis of acute trials comparing the effects of non-nutritive sweetened beverages (NNS beverages) with water and sugar-sweetened beverages (SSBs) in humans. **Methods:** MEDLINE, EMBASE, and The Cochrane Library were searched through to January 15, 2022. We included acute, single-exposure, randomized, and non-randomized, clinical trials in humans, regardless of health status. Three patterns of intake were examined: (1) uncoupling interventions, where NNS beverages were consumed alone without added energy or nutrients; (2) coupling interventions, where NNS beverages were consumed together with added energy and nutrients as carbohydrates; and (3) delayed coupling interventions, where NNS beverages were consumed as a preload prior to added energy and nutrients as carbohydrates. The primary outcome was a 2 h incremental area under the curve (iAUC) for blood glucose concentration. Secondary outcomes included 2 h iAUC for insulin, glucagon-like peptide 1 (GLP-1), gastric inhibitory polypeptide (GIP), peptide YY (PYY), ghrelin, leptin, and glucagon concentrations. Network meta-analysis and confidence in the network meta-analysis (CINeMA) were conducted in R-studio and CINeMA, respectively. **Results:** Thirty-six trials involving 472 predominantly healthy participants were included. Trials examined a variety of single NNS (acesulfame potassium, aspartame, cyclamate, saccharin, stevia, and sucralose) and NNS blends (acesulfame potassium + aspartame, acesulfame potassium + sucralose, acesulfame potassium + aspartame + cyclamate, and acesulfame potassium + aspartame + sucralose), along with matched water/unsweetened controls and SSBs sweetened with various caloric sugars (glucose, sucrose, and fructose). In uncoupling interventions, NNS beverages (single or blends) had no effect on postprandial glucose, insulin, GLP-1, GIP, PYY, ghrelin, and glucagon responses similar to water controls (generally, low to moderate confidence), whereas SSBs sweetened with caloric sugars (glucose and sucrose) increased postprandial glucose, insulin, GLP-1, and GIP responses with no differences in postprandial ghrelin and glucagon responses (generally, low to moderate confidence). In coupling and delayed coupling interventions, NNS beverages had no postprandial glucose and endocrine effects similar to controls (generally, low to moderate confidence). **Conclusions:** The available evidence suggests that NNS beverages sweetened with single or blends of NNS have no acute metabolic and endocrine effects, similar to water. These findings provide support for NNS beverages as an alternative replacement strategy for SSBs in the acute postprandial setting.

## 1. Introduction

Sugars have emerged as the dominant nutrient of concern in research on human health and disease [1]. This concern has resulted in calls for reductions in free sugars to ≤5–10% of energy by several international health agencies [2,3,4] and chronic disease associations [5,6]. Attention has focused especially on the major source of free sugars, sugar-sweetened beverages (SSBs), the excess consumption of which has been associated with weight gain, diabetes, and their downstream complications, including hypertension and coronary heart disease (CHD) [7,8,9,10].

Replacing SSBs with non-nutritive sweetened beverages (NNS beverages) provide a viable means to limit excess calories and potentially avoid downstream complications associated with weight gain. The U.S. Food and Drug Administration (FDA) has currently approved eight non-nutritive sweeteners (NNS): aspartame, acesulfame potassium (ace-K), luo han guo (monk) fruit extract, neotame, saccharin, stevia, sucralose, and advantame [11]. Despite safety approvals by major health and regulatory bodies [11,12,13,14], evidence from prospective cohort studies suggests that many of these NNS may increase the risk of cardiometabolic diseases [15,16].

Much of the attention to explaining such signals of harm has focused on the acute metabolic and endocrine responses to NNS [17]. Some have proposed that NNS act upon intestinal sweet taste receptors leading to the impaired postprandial release of glucagon-like peptide 1 (GLP-1) and insulin [18,19]. Others have suggested that NNS induces glucose intolerance [20] and impairs metabolic sensitivity to carbohydrates [21]. Such concerns regarding NNS are often based on studies that attribute results of single NNS to the whole class [22], despite NNS being metabolically distinct compounds [23]. Whether these proposed mechanisms are operational under real-world intakes is unclear.

Addressing these metabolic concerns requires careful consideration of key methodological and design issues including the pattern of intake, type of NNS, and nature of the comparator [17]. To address this, we undertook this systematic review and network meta-analysis to compare the effect of NNS beverages sweetened with single NNS and blends of NNS with water and SSBs sweetened with caloric sugars on postprandial glycemic and endocrine responses.

## 2. Materials and Methods

### 2.1. Protocol Registration

The study protocol was registered on the Open Science Forum (OSF) registry [24].

### 2.2. Design

The present systematic review and network meta-analysis was conducted according to the Cochrane Handbook for Systematic Reviews of Interventions [25] and reported using the Preferred Reporting Items for Systematic Reviews and Meta-Analyses Involving a Network Meta-analysis (PRISMA-Network Meta-analysis) [26].

### 2.3. Data Sources and Searches

MEDLINE, EMBASE, and the Cochrane Central Register of Controlled Trials were searched through 15 January 2022 for eligible trials using the search strategy presented in Supplementary Table S1. Electronic searches were supplemented with manual searches of references from selected studies and reviews.

### 2.4. Study Selection

Supplementary Table S2 shows our PICOTS (population, intervention, comparator, outcome, time, and settings) framework. Randomized and non-randomized, acute (i.e., 2 h follow-up duration), single-exposure, crossover, clinical trials in individuals of all health backgrounds that investigated the oral consumption of NNS beverages containing NNS (single or blends) that had been approved by the U.S. FDA were eligible [11]. Comparisons among the following single interventions were included: NNS beverages sweetened with single NNS or blends of NNS, water, and SSBs sweetened with caloric sugars. Trials were excluded if they involved sugar alcohols (e.g., erythritol), rare sugars (e.g., allulose), pregnant or breastfeeding women, non-fasting participants at baseline, had a duration of less than 2 h, did not use a comparator arm, and did not provide suitable endpoint data.

As the presence of other nutrients (e.g., calories in form of carbohydrates) and timing of administration (e.g., preload) have independent effects on glycemic and endocrine responses, three patterns of intakes were analyzed separately: (i) uncoupling interventions, where NNS beverages were consumed without added energy or nutrients, (ii) coupling interventions, where NNS beverages were consumed together with added energy and nutrients as carbohydrates, and (iii) delayed coupling interventions, where NNS beverages were consumed as a preload prior to added energy and nutrients as carbohydrates. The preload period was set to be less than or equal to 15 min.

### 2.5. Data Extraction

Two investigators (from a pool of 4: RZ, JCN, TAK, and NM) independently reviewed and extracted relevant data from each included report. Extracted data included participant characteristics (e.g., health status, age, sex, and BMI), sample size, description of interventions (name and amount), study design (randomized and non-randomized), NNS pattern of intake (uncoupling, coupling, or delayed coupling intervention), duration of follow-up, setting, funding sources, and outcome data. In studies with follow-up duration >2 h, only the 2 h incremental area under the curve (iAUC) was extracted to ensure consistency across studies. Furthermore, 2 h iAUC is a standard way of testing for and expressing postprandial blood glucose response (PPGR) across meals. In the absence of numerical values for outcome data and the inability to contact study authors, values were extracted from figures using Plot Digitizer, version 2.5.1 (Free Software Foundation, Boston, MA), and computed using standard formulas [27,28]. Disagreements were resolved by discussion or, if necessary, by consultation with senior authors (TAK and JLS).

### 2.6. Risk of Bias Assessment

Risk of bias was evaluated using version 2 of the Cochrane risk-of-bias (RoB 2) tool, where bias was assessed in five distinct domains (bias arising from the randomization process, bias due to deviations from intended interventions, bias due to missing outcome data, bias in measurement of the outcome, and bias in selection of the reported result). Within each domain, the investigators answered one or more signaling questions and these answers led to judgments of “low risk of bias”, “some concerns”, or “high risk of bias” [29].

### 2.7. Outcomes

The primary outcome was glucose iAUC. Secondary outcomes were iAUC for insulin, glucagon-like peptide 1 (GLP-1), gastric inhibitory polypeptide (GIP), peptide YY (PYY), ghrelin, leptin, and glucagon.

### 2.8. Data Synthesis

Where postprandial data at individual timepoints were extracted, the iAUC and variation were computed using the formula outlined in Supplementary Figure S1. Positive iAUC was computed for all outcomes, except for ghrelin and glucagon, where the negative iAUC was computed. Prior to analysis, all endpoints were converted to SI units (mmol/L for glucose (=mg/dL × [1/18]), pmol/L for insulin (=μU/mL × 6), pmol/L for GLP-1 (pg/mL × 0.3032), pmol/L for GIP (pg/mL × [1/4.75]), pmol/L for PYY (pg/mL × 0.25), pmol/L for ghrelin (pg/mL × 0.3), and pmol/L for glucagon (pg/mL × [1/3.45]).

All statistical analyses were performed in R (R Foundation) using the netmeta packages [30,31]. We evaluated confidence in network meta-analysis effect estimates for all outcomes and treatment comparisons using the CINeMA (Confidence In Network Meta-Analysis) framework [32,33].

## 3. Results

### 3.1. Search Results

Supplementary Figure S2 shows the literature search and selection process. Of 2846 reports identified, 2707 were excluded based on title and abstracts. Of 139 reports reviewed in full, 114 were excluded based on full article review. A total of 25 reports [18,34,35,36,37,38,39,40,41,42,43,44,45,46,47,48,49,50,51,52,53,54,55,56,57] containing data for 36 acute feeding trials of beverages (*n* = 472) met the eligibility criteria for inclusion. Of these, fifteen reports (21 trials, *n* = 266) examined uncoupling interventions [34,35,36,37,38,39,40,41,42,43,44,45,46,47,48], three reports (3 trials, *n* = 27) examined coupling interventions [49,50,51], and seven reports (12 trials, *n* = 179) examined delayed coupling interventions [18,52,53,54,55,56,57].

### 3.2. Trial Characteristics

Supplementary Tables S3–S5 show the characteristics of included trials that examined uncoupling, coupling, and delayed coupling interventions.

Uncoupling interventions [34,35,36,37,38,39,40,41,42,43,44,45,46,47,48]: trials were conducted in Europe (45%), Asia (30%), North America (20%), and South America (5%). Trial funding came from agency (35%) and industry sources (10%), with the majority of trials not reporting funding sources (55%). The trial sample size ranged from 6 to 32 participants (51% male, 49% female) aged (median (range) of the reported means) 31.3 (21.3–69.0) years with a BMI of 23.5 (21.2–33.7) kg/m^2^. Trials were mainly conducted in otherwise healthy individuals (75%), with 5 trials (18%) in individuals with type 2 diabetes and 1 trial (8%) in individuals with impaired glucose tolerance. Trials examined NNS beverages sweetened with single NNS, including ace-K (dose, 165 mg), aspartame (median dose (range), 400 mg (165–1000 mg)), cyclamate (800 mg), saccharin (135 mg (75–135 mg)), sucralose (120 mg (40–200 mg)), and NNS blends, including ace-K (56 mg) + aspartame (84 mg), and ace-K + aspartame + cyclamate (dose not provided), along with matched water controls and SSBs sweetened with glucose (75.7 g (75–100 g)), sucrose (35 g (20–76.3 g)), and fructose (76.3 g).

Coupling interventions [49,50,51]: two of three trials were conducted in Europe (66%) and one trial was conducted in North America (33%). All trials were funded by agency sources. The trial sample size ranged from 7 to 10 participants (33% male, 67% female) aged 27.0 (21.7–27.2) with a BMI of 22.3 (20.6–23.9). All trials were conducted in otherwise healthy individuals. Trials examined NNS beverages sweetened with single NNS, including aspartame (140 mg (80–200 mg)) and NNS blends including ace-K (58 mg) + aspartame (31 mg) with carbohydrates loads, including a cherry flavored beverage containing 60 g carbohydrate as partial hydrolysate (one trial), a chocolate drink containing 20 g milk protein, 5 g fat-free and sucrose-free cocoa with 5 g agar (one trial), and a 25 g oral glucose solution (one trial).

Delayed coupling interventions [18,52,53,54,55,56,57]: trials were mainly conducted in North America (83%) and Europe (17%). The majority of trials were funded by agency sources (75%), with the remainder not reporting funding sources (25%). The trial sample size ranged from 8 participants to 31 participants (37% male, 63% female) aged 28.5 (17.9–51.5) with a BMI of 26.1 (21.7–41.0). The majority of trials were conducted in otherwise healthy participants (75%), with 1 trial (8%) in individuals with type 1 diabetes and 2 trials (17%) in individuals with type 2 diabetes. Trials examined NNS beverages sweetened with single NNS, including aspartame (72 mg), saccharin (18 mg), and sucralose (48 mg(24–170 mg)), and NNS blends including ace-K (58 mg) + aspartame (31 mg), ace-K (41 mg) + sucralose (68 mg), and ace-K (18 mg) + aspartame (57 mg) + sucralose (18 mg), along with matched water controls as preloads. All trials utilized a 75 g oral glucose solution as the carbohydrate load, except one trial [57] which utilized a 25 g oral glucose solution.

### 3.3. Risk of Bias

Supplementary Figures S3–S5 present the risk of bias in each study examining uncoupling, coupling, and delayed coupling interventions. The overall risk of bias was low in 73% (11/15) of uncoupling interventions, 33% (1/3) of studies examining coupling interventions, and 86% (6/7) of delayed coupling interventions. Some concerns in the overall risk of bias were observed in 27% (4/15) of uncoupling interventions, 66% (2/3) of coupling interventions, and 14% (1/7) of delayed coupling interventions. The key limitation was bias arising from the randomization process due presence of non-randomized trials in the overall analysis.

### 3.4. Primary Outcome (Glucose)

#### 3.4.1. Uncoupling Interventions

**Figure 1:**
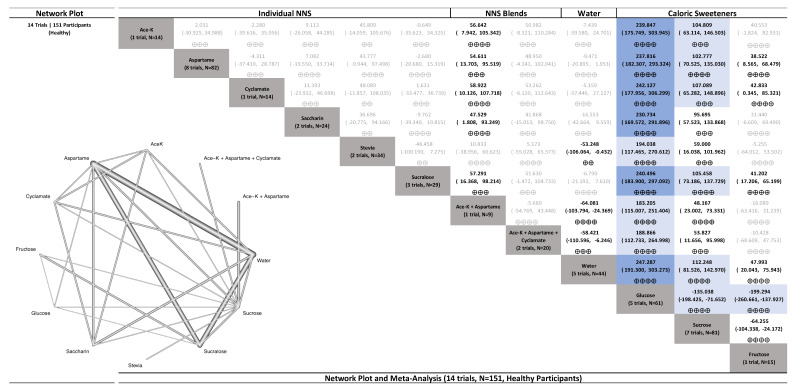
Network plot and meta-analysis of uncoupling interventions evaluating the effect of non-nutritive sweetened beverages (NNS beverages) sweetened single or blends of non-nutritive sweeteners (NNS), water, and sugar-sweetened beverages (SSBs) sweetened with caloric sweeteners on postprandial blood glucose response in healthy participants. Network plot: the size of the nodes is proportional to the number of participants and the line width is proportional to the number of studies. Network table: treatments are grouped by treatment type (i.e., single NNS, NNS blends, water, and caloric sweeteners) and are reported in alphabetical order. Treatment estimates (mmol*min/L) are MDs and 95% CIs of the column-defining treatment compared with the row-defining treatment. MDs less than 0 favor the column-defining treatment. MDs greater than 0 favor the row-defining treatment. Statistically significant results are bolded in black. Results that are not statistically significant are grey and not bolded. The minimally important difference (MID) for postprandial glucose response is 100 mmol*min/L. Trivial effects (<1 MID) or no effects have a white background; small important effects (≥1 MID) have a light blue background; moderate effects (≥2 MID) have a darker blue background; large effects (≥5 to <10 MID) have a purple background; and very large effects (≥10 MID) have a black background. Confidence in the effect estimate (CINeMA) is shown for each treatment comparison: high confidence ⊕⊕⊕⊕; moderate confidence ⊕⊕⊕; low confidence ⊕⊕; and very low confidence ⊕. See Supplementary Table S6 for overall CiNEMA assessments and Supplementary Figures S6–S9 for detailed assessments of the confidence in the effect estimate using the CINeMA framework.

The results for uncoupling interventions examining postprandial glucose in healthy participants are presented in Figure 1 with CINeMA assessments presented in Supplementary Table S6 and Figures S6–S9. NNS beverages sweetened with single NNS and NNS blends had no effect on postprandial glucose similar to water (generally, moderate to high confidence), whereas SSBs sweetened with caloric sugars (glucose and sucrose) increased postprandial glucose (mostly, high confidence).

**Figure 2:**
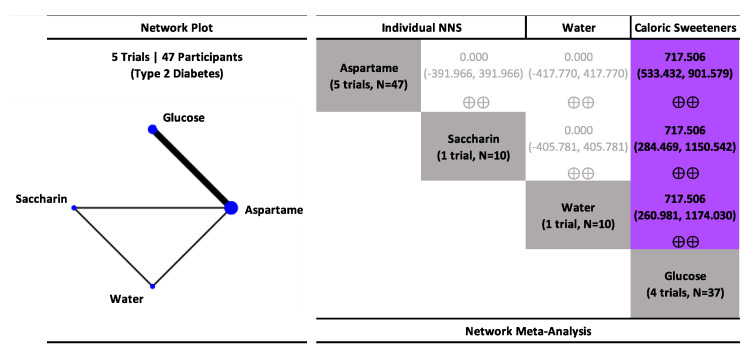
Network plot and meta-analysis of uncoupling interventions evaluating the effect of non-nutritive sweetened beverages (NNS beverages) sweetened single or blends of non-nutritive sweeteners (NNS), water, and sugar-sweetened beverages (SSBs) sweetened with caloric sweeteners on postprandial blood glucose response in participants with type 2 diabetes. Network plot: the size of the blue nodes is proportional to the number of participants and the line width is proportional to the number of studies. Network table: treatments are grouped by treatment type (i.e., individual non-nutritive sweeteners (NNS), NNS blends, water, and caloric sweeteners) and are reported in alphabetical order. Treatment estimates (mmol*min/L) are MDs and 95% CIs of the column-defining treatment compared with the row-defining treatment. MDs less than 0 favor the column-defining treatment. MDs greater than 0 favor the row-defining treatment. Significant results are bolded in black. Non-significant results are grey and not bolded. The minimally important difference (MID) for postprandial glucose response is 100 mmol*min/L. Trivial (significant) effects (<1 MID) or no effects have a white background; small important effects (≥1 MID) have a light blue background; moderate effects (≥2 MID) have a darker blue background; large effects (≥5 to <10 MID) have a purple background; and very large effects (≥10 MID) have a black background. Confidence in the effect estimate is shown for each treatment comparison: high confidence ⊕⊕⊕⊕; moderate confidence ⊕⊕⊕; low confidence ⊕⊕; and very low confidence ⊕. See Supplementary Table S7 and Figures S10–S13 for detailed assessments of the confidence in the effect estimate using the CINeMA framework.

Network meta-analysis results in participants with type 2 diabetes are presented in Figure 2, Supplementary Table S7, and Figures S10–S13. NNS beverages sweetened with aspartame and saccharin had no effect on postprandial glucose (low confidence) similar to water, whereas SSBs sweetened with glucose increased postprandial glucose (low confidence).

Only one trial comparison (*n* = 20) was identified in participants with impaired glucose tolerance that found no statistically significant difference in postprandial glucose when comparing NNS beverages sweetened with aspartame with a glucose solution (low confidence; Supplementary Table S25).

#### 3.4.2. Coupling Interventions

**Figure 3:**
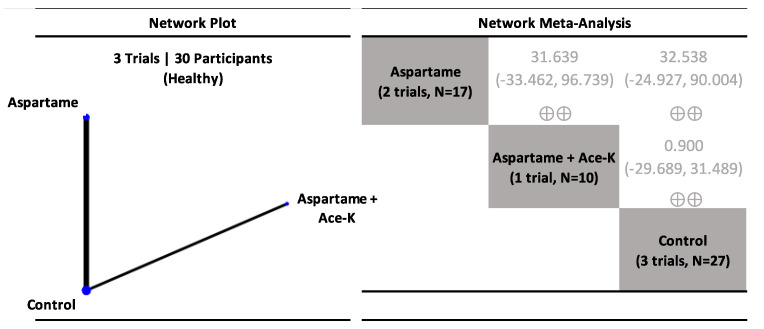
Network plot and meta-analysis of coupling interventions evaluating the effect of non-nutritive sweetened beverages (NNS beverages) sweetened single or blends of non-nutritive sweeteners (NNS) and controls on postprandial glucose response in healthy participants. Network plot: the size of the blue nodes is proportional to the number of participants and the line width is proportional to the number of studies. Network table: treatments are grouped by treatment type (i.e., individual non-nutritive sweeteners (NNS), NNS blends, water, and caloric sweeteners) and are reported in alphabetical order. Treatment estimates (mmol*min/L) are MDs and 95% CIs of the column-defining treatment compared with the row-defining treatment. MDs less than 0 favor the column-defining treatment. MDs greater than 0 favor the row-defining treatment. Significant results are bolded in black. Non-significant results are grey and not bolded. The minimally important difference (MID) for postprandial glucose response is 100 mmol*min/L. Trivial (significant) effects (<1 MID) or no effects have a white background; small important effects (≥1 MID) have a light blue background; moderate effects (≥2 MID) have a darker blue background; large effects (≥5 to <10 MID) have a purple background; and very large effects (≥10 MID) have a black background. Confidence in the effect estimate is shown for each treatment comparison: high confidence ⊕⊕⊕⊕; moderate confidence ⊕⊕⊕; low confidence ⊕⊕; and very low confidence ⊕. See Supplementary Table S15 and Figure S34 for detailed assessments of the confidence in the effect estimate using the CINeMA framework.

The results for coupling interventions examining postprandial glucose in healthy participants are presented in Figure 3, Supplementary Table S15, and Figure S34. NNS beverages sweetened with aspartame (single NNS) and aspartame + ace-K (NNS blend) had similar postprandial glucose responses to the control (low confidence).

#### 3.4.3. Delayed Coupling Interventions

**Figure 4:**
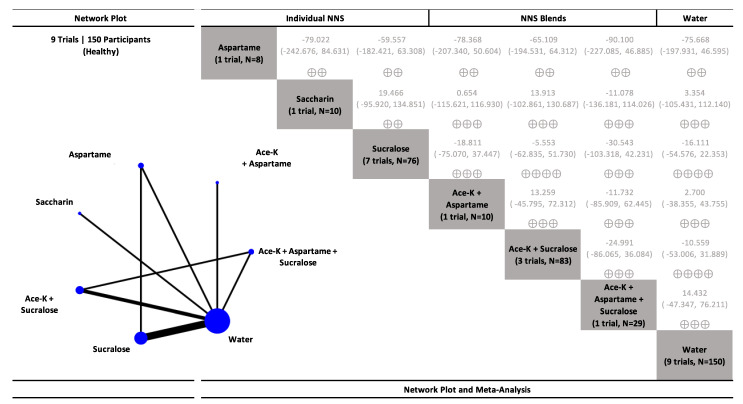
Network plot and meta-analysis of delayed coupling interventions evaluating the effect of non-nutritive sweetened beverages (NNS beverages) sweetened single or blends of non-nutritive sweeteners (NNS), water, and sugar-sweetened beverages (SSBs) sweetened with caloric sweeteners on postprandial blood glucose response in healthy participants. Network plot: the size of the blue nodes is proportional to the number of participants and the line width is proportional to the number of studies. Network table: treatments are grouped by treatment type (i.e., individual non-nutritive sweeteners (NNS), NNS blends, water, and caloric sweeteners) and are reported in alphabetical order. Treatment estimates (mmol*min/L) are MDs and 95% CIs of the column-defining treatment compared with the row-defining treatment. MDs less than 0 favor the column-defining treatment. MDs greater than 0 favor the row-defining treatment. Significant results are bolded in black. Non-significant results are grey and not bolded. The minimally important difference (MID) for postprandial glucose response is 100 mmol*min/L. Trivial (significant) effects (<1 MID) or no effects have a white background; small important effects (≥1 MID) have a light blue background; moderate effects (≥2 MID) have a darker blue background; large effects (≥5 to <10 MID) have a purple background; very large effects (≥10 MID) have a black background. Confidence in the effect estimate is shown for each treatment comparison: high confidence ⊕⊕⊕⊕; moderate confidence ⊕⊕⊕; low confidence ⊕⊕; very low confidence ⊕. See Supplementary Table S17 and Figures S37–S40 for detailed assessments of the confidence in the effect estimate using the CINeMA framework.

**Figure 5:**
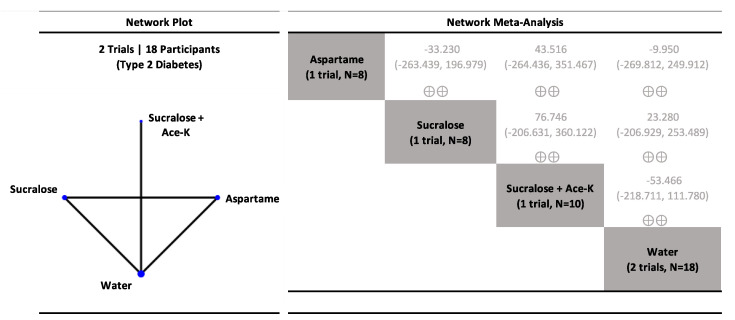
Network plot and meta-analysis of delayed coupling interventions evaluating the effect of non-nutritive sweetened beverages (NNS beverages) sweetened single or blends of non-nutritive sweeteners (NNS), water, and sugar-sweetened beverages (SSBs) sweetened with caloric sweeteners on postprandial blood glucose response in participants with type 2 diabetes. Network plot: the size of the blue nodes is proportional to the number of participants and the line width is proportional to the number of studies. Network table: treatments are grouped by treatment type (i.e., individual non-nutritive sweeteners (NNS), NNS blends, water, and caloric sweeteners) and are reported in alphabetical order. Treatment estimates (mmol*min/L) are MDs and 95% CIs of the column-defining treatment compared with the row-defining treatment. MDs less than 0 favor the column-defining treatment. MDs greater than 0 favor the row-defining treatment. Significant results are bolded in black. Non-significant results are grey and not bolded. The minimally important difference (MID) for postprandial glucose response is 100 mmol*min/L. Trivial (significant) effects (<1 MID) or no effects have a white background; small important effects (≥1 MID) have a light blue background; moderate effects (≥2 MID) have a darker blue background; large effects (≥5 to <10 MID) have a purple background; and very large effects (≥10 MID) have a black background. Confidence in the effect estimate is shown for each treatment comparison: high confidence ⊕⊕⊕⊕; moderate confidence ⊕⊕⊕; low confidence ⊕⊕; and very low confidence ⊕. See Supplementary Table S18 and Figure S41 for detailed assessments of the confidence in the effect estimate using the CINeMA framework.

The results for delayed coupling interventions examining postprandial glucose in healthy participants are presented in Figure 4, Supplementary Table S17, and Figures S37–S40, and results for participants with type 2 diabetes are presented in Figure 5, Supplementary Table S18, and Figure S41. NNS beverages sweetened with single NNS and NNS blends had no effect on postprandial glucose similar to water controls in both healthy individuals (generally, low to moderate confidence) and participants with type 2 diabetes (generally, low confidence).

Single-trial comparisons were identified in participants living with obesity (comparing NNS beverages sweetened with sucralose vs. water control) and participants with type 1 diabetes (comparing NNS beverages sweetened with sucralose + ace-K vs. water control), and effects were similar in the postprandial glucose response (low confidence; Supplementary Table S25).

### 3.5. Secondary Endocrine Outcomes (Insulin, GLP-1, PYY, GIP, Ghrelin, Leptin, and Glucagon)

#### 3.5.1. Uncoupling Interventions

The results for uncoupling interventions examining postprandial insulin response, GLP-1, GIP, ghrelin, and glucagon, are presented in Supplementary Figures S14–S33 and Table S8–S14 for healthy participants and those with type 2 diabetes in uncoupling interventions. NNS beverages (single or blends) had no effect on postprandial insulin, GLP-1, GIP, PYY, ghrelin, and glucagon responses similar to water controls (generally, low to moderate confidence), whereas SSBs sweetened with caloric sugars (glucose and sucrose) increased postprandial insulin, GLP-1, and GIP responses with no differences in postprandial ghrelin and glucagon responses (generally, low to moderate confidence).

In participants with impaired glucose tolerance, there were no differences in postprandial insulin response when NNS beverages sweetened with aspartame were compared to SSBs sweetened with glucose (low confidence; Supplementary Table S25).

No trials were identified that examined postprandial leptin response.

#### 3.5.2. Coupling Interventions

The results for postprandial insulin response for healthy participants in coupling interventions are presented in Figure S35 with CINeMA assessments in Supplementary Figure S36 and Table S16. NNS beverages sweetened with aspartame (single NNS) had a similar effect on postprandial insulin response compared with the control (low confidence). There were no coupling interventions evaluating the other postprandial endocrine outcomes (e.g., GLP-1, PYY, GIP, ghrelin, leptin, and glucagon).

#### 3.5.3. Delayed Coupling Interventions

Results for healthy participants and participants with type 2 diabetes in delayed coupling interventions for secondary outcomes are presented in Supplementary Figures S42–S62 and Tables S19–S24. NNS beverages sweetened with single NNS or NNS blends had no effect on postprandial insulin, GLP-1, GIP, and glucagon responses similar to water controls (generally, low to moderate confidence).

Single-trial comparisons were identified in participants living with obesity (comparing NNS beverages sweetened with sucralose vs. water control), examining postprandial insulin response in participants with type 1 diabetes (comparing NNS beverages sweetened with sucralose + ace-K vs. water control), examining postprandial insulin, GLP-1, GIP, and glucagon responses in type 2 diabetes (comparing NNS beverages sweetened with sucralose + ace-K vs. water control), and examining postprandial GIP and glucagon responses. No difference in the endpoints was observed among these trial comparisons (low confidence; Supplementary Table S25).

No trials were identified that examined postprandial leptin response.

## 4. Discussion

### 4.1. Summary of Findings

This systematic review and network meta-analysis of 36 acute feeding trials involving 472 predominantly healthy participants compared the effect of NNS beverages sweetened with single or blends of NNS with water and sugar-sweetened beverages (SSBs) sweetened with various caloric sugars on postprandial glucose and endocrine responses. Three pre-specified patterns of intakes were examined: (1) uncoupling interventions (NNS beverages consumed alone without added energy or nutrients), (2) coupling interventions (NNS beverages consumed together with additional energy and nutrients as carbohydrates), and (3) delayed coupling interventions (NNS beverages consumed as a preload prior to added energy and nutrients as carbohydrates). In uncoupling interventions, NNS beverages (single or blends) had no effect on postprandial glucose, insulin, GLP-1, GIP, PYY, ghrelin, and glucagon responses similar to water controls, whereas SSBs sweetened with caloric sugars (glucose and sucrose) increased postprandial glucose, insulin, GLP-1, and GIP responses with no differences in ghrelin and glucagon responses. In coupling interventions, NNS beverages (single or blends) had no effect on the postprandial glucose and insulin responses to carbohydrate loads similar to controls. In delayed coupling interventions, NNS beverages (single and blends) had no effect on postprandial glucose, insulin, GLP-1, GIP, and glucagon responses to carbohydrate loads similar to water controls.

### 4.2. Findings in the Context of Existing Studies

Our findings are in agreement with previous systematic reviews and meta-analyses that examined the effect of NNS on acute glucose and insulin responses. The findings that NNS has no acute effects on postprandial glucose and insulin response when compared to a control intervention were recently shown by Greyling et al. [58], by Nichol et al. [59], and by Tucker et al. [60] in a prior systematic review and meta-analyses. Despite similar findings, there are several differences between these reports and our study: (1) our network meta-analysis was a priori rather than post hoc, (2) in addition to postprandial glucose and insulin outcomes, we also examined other endocrine responses, including GLP-1, PYY, GIP, ghrelin, and glucagon, (3) we examined the certainty of evidence using CINeMA and the GRADE approach, and (4) in addition to water/unsweetened controls, we also compared and quantified the effect of NNS beverages with SSBs, where data were available.

The evidence from our study, which shows no association with acute metabolic and endocrine outcomes, is at odds with the systematic review and meta-analysis of cohort studies that used prevalent exposure to show long-term harm with NNS consumption [15]. Such meta-analyses have a higher risk of bias due to residual confounding, reverse causality, and behavior clustering [61,62,63]. The recently published systematic reviews and meta-analyses of both cohort studies [64] and RCTs [65] that used rigorous methods to protect against such bias are in line with our results. These studies showed that NNS is not associated with cardiometabolic harm and can be used as a replacement strategy to reduce risk from intake of empty calories from SSBs.

Our study results may also be at odds with results from select narrative reviews, in-vitro, animal, and human studies [19,20,21,22] because these failed to carefully consider key methodological and design issues, including the pattern of intake, type of NNS, and nature of the comparator. Our work addressed these gaps and provides a more rigorous body of work.

In this systematic review, we excluded two trials [66,67] due to the consumption of beverages in non-fasting conditions and excluded one trial [68] due to preload duration being more than 15 min. However, it is still worth examining the findings from these trials as they have some relevance to our study objectives. Tey et al. [66] examined the effects of aspartame-, monk fruit-, stevia-, and sucrose-sweetened beverages on postprandial glucose and insulin responses following a standardized breakfast in 30 healthy male participants. The authors concluded that the consumption of beverages sweetened with the three different NNS had minimal influences on glucose and insulin responses when compared to sucrose-sweetened beverages. Pearson et al. [67] examined the effects of 20 oz of Diet Coke, Coca-Cola, or water with a mixed meal following a pre-trial meal in eight college-aged, healthy males. The authors found that although there was no significant difference in the glucose response among treatments; the insulin response was significantly higher after consumption of the Coca-Cola treatment when compared to the Diet Coke treatment, and trended higher when compared to the water treatment (*p* = 0.054). Lastly, in a study by Brown et al. [68] which examined postprandial glucose, insulin, and ghrelin responses in eight female participants, a combination of 50 g of sucrose and 6 g of granular sucralose in 355 mL of water resulted in similar responses when compared to 50 g of sucrose dissolved in 355 mL of water, whereas 6 g of granular sucralose dissolved in 355 mL of water resulted in similar effects when compared to 355 mL of water alone. Overall, even though these trials were excluded from our analysis, the findings from these trials are in line with our findings.

### 4.3. Potential Mechanisms

The “sweet uncoupling hypothesis” purports that the uncoupling of sweet taste from caloric content in the case of NNS may disrupt the metabolic consequences of sweet taste [69,70], likely through acute hormonal changes [71]. Our study is uniquely set to test this hypothesis. The first set of studies we examined were NNS which were consumed without any additional calories and, therefore, were uncoupled from energy. The acute intake of these uncoupled beverages did not elicit any acute hormonal response and was similar to water. We saw similar results in healthy and people with type 2 diabetes. These studies confirmed that NNS were inert, and their intake did not elicit a sweet uncoupling through acute metabolic and hormonal response. Although we found that some NNS blends showed a slight increase in postprandial glucose response, the effects were trivial and likely to be noise. However, still this raises the question of blending of NNS which may require further studies to see if any unique combination may have some unknown synergistic mechanism of action.

The evidence against the sweet uncoupling hypothesis has been mounting and some argue that the NNS might alter metabolism as it is simultaneously consumed with glucose or other caloric foods [21]. In our second and third sets of studies, we examined the effect of coupling NNS with calories and NNS as a preload, in which they are consumed slightly before providing calories. In both sets of studies, the results displayed the inert nature of NNS and showed that they do not produce any alteration in acute glucose or other metabolic responses including insulin, GLP-1, GIP, and glucagon.

### 4.4. Strengths and Limitations

The present systematic review and network meta-analysis has several strengths. The use of network meta-analysis allowed for simultaneous comparison of beverages sweetened with NNS (single and blends) with water and beverages sweetened with caloric sweeteners. This was important because NNS represent a heterogeneous group of compounds with distinct absorption, distribution, metabolism, and excretion kinetics [23]. In addition, beverages sweetened with caloric sweeteners (e.g., sucrose) are the target of the intended replacement strategy, while water typically represents the “standard of care”. Other strengths included a comprehensive literature search, extension of outcomes beyond glucose and insulin, recalculation of missing iAUC data in studies, subgroups by patterns of food intake and health status, and use of CINeMA and GRADE to assess the confidence in our effect estimates.

There were also several limitations in our synthesis. First, there was evidence of serious imprecision in several pooled estimates, particularly when comparing beverages sweetened with NNS (single and blends) with each other and with water. The 95% CI crossed the prespecified minimal important difference for the primary outcome of postprandial blood glucose and several secondary outcomes across the three patterns of intakes. Evidence was downgraded further for several secondary outcomes due to the availability of only one or two direct comparisons. Second, there was evidence of within-study bias for several pooled estimates in the primary and secondary outcomes due to the inclusion of non-randomized trials. Third, a few pooled estimates in the primary and secondary outcomes were downgraded due to heterogeneity and incoherence, as computed by the CiNEMA software.

Balancing these strengths and limitations, we assessed the confidence in the estimates as generally moderate to high for postprandial glucose and insulin when comparing NNS beverages sweetened with single or blends of NNS with water/unsweetened controls and SSBs sweetened with caloric sweeteners across the three patterns of intakes. For the remaining outcomes, we assessed the confidence as generally low to moderate.

### 4.5. Implications

Our findings are relevant to the biological plausibility that has been proposed by several epidemiological and experimental studies related to NNS consumption and the potential for cardiometabolic harm [16]. In our analyses, we found no differences in acute metabolic and endocrine responses which regulate glucose and food intake regulation when comparing NNS (single and blends) with water across three patterns of intake (uncoupled, coupled, and delayed coupling interventions). Furthermore, these findings support data from recent systematic reviews and network meta-analyses of RCTs [65] and prospective cohort studies [64] that support substituting SSBs with NNS beverages for cardiometabolic benefits. In addition to single sweeteners, we also examined NNS blends which are commonly consumed making our findings relevant to the real-world [72]. Lastly, our analyses focused on beverages, the most important source of NNS in a diet, and a single food matrix [73,74].

## 5. Conclusions

This systematic review and network meta-analysis found that NNS beverages sweetened with single or blends of NNS had no meaningful effects on postprandial glucose and endocrine responses across three patterns of food intake (uncoupling interventions, coupling interventions, and delayed coupling interventions). Together, these data fall in line with recent syntheses of long-term data from RCTs and prospective cohort studies which support the use of NNS beverages as an alternative replacement strategy for SSBs similar to water (the “standard of care”).

## Data Availability

Data are available upon request.

## Supplementary Materials

The following supporting information can be downloaded at https://www.mdpi.com/article/10.3390/nu15041050/s1. Table S1: Search strategy, Table S2: PICOTS framework, Table S3: Characteristics of UNCOUPLING INTERVENTIONS, Table S4: Characteristics of COUPLING INTERVENTIONS, Table S5: Characteristics of DELAYED COUPLING INTERVENTIONS, Table S6: OVERALL CINeMA assessments of UNCOUPLING INTERVENTIONS examining the effect of non-nutritive sweetened beverages (NNS beverages) sweetened with individual or blends of non-nutritive sweeteners (NNS), water, and sugar-sweetened beverages (SSBs) sweetened with caloric sweeteners on postprandial blood GLUCOSE response in HEALTHY participants, Table S7: OVERALL CINeMA assessments of UNCOUPLING INTERVENTIONS examining the effect of non-nutritive sweetened beverages (NNS beverages) sweetened with individual or blends of non-nutritive sweeteners (NNS), water, and sugar-sweetened beverages (SSBs) sweetened with caloric sweeteners on postprandial blood GLUCOSE response in participants with TYPE 2 DIABETES, Table S8: OVERALL CINeMA assessments of UNCOUPLING INTERVENTIONS examining the effect of non-nutritive sweetened beverages (NNS beverages) sweetened with individual or blends of non-nutritive sweeteners (NNS), water, and sugar-sweetened beverages (SSBs) sweetened with caloric sweeteners on postprandial blood INSULIN response in HEALTHY participants, Table S9: OVERALL CINeMA assessments of UNCOUPLING INTERVENTIONS examining the effect of non-nutritive sweetened beverages (NNS beverages) sweetened with individual or blends of non-nutritive sweeteners (NNS), water, and sugar-sweetened beverages (SSBs) sweetened with caloric sweeteners on postprandial blood INSULIN response in participants with TYPE 2 DIABETES, Table S10: GRADE assessments of UNCOUPLING INTERVENTIONS examining the effect of non-nutritive sweetened beverages (NNS beverages) sweetened with individual or blends of non-nutritive sweeteners (NNS), water, and sugar-sweetened beverages (SSBs) sweetened with caloric sweeteners on postprandial blood GLP-1 response in HEALTHY participants, Table S11: GRADE assessments of UNCOUPLING INTERVENTIONS examining the effect of non-nutritive sweetened beverages (NNS beverages) sweetened with individual or blends of non-nutritive sweeteners (NNS), water, and sugar-sweetened beverages (SSBs) sweetened with caloric sweeteners on postprandial blood GIP response in HEALTHY participants, Table S12: GRADE assessments of UNCOUPLING INTERVENTIONS examining the effect of non-nutritive sweetened beverages (NNS beverages) sweetened with individual or blends of non-nutritive sweeteners (NNS), water, and sugar-sweetened beverages (SSBs) sweetened with caloric sweeteners on postprandial blood GHRELIN response in HEALTHY participants, Table S13: GRADE assessments of UNCOUPLING INTERVENTIONS examining the effect of non-nutritive sweetened beverages (NNS beverages) sweetened with individual or blends of non-nutritive sweeteners (NNS), water, and sugar-sweetened beverages (SSBs) sweetened with caloric sweeteners on postprandial blood GLUCAGON response in HEALTHY participants, Table S14: GRADE assessments of UNCOUPLING INTERVENTIONS examining the effect of non-nutritive sweetened beverages (NNS beverages) sweetened with individual or blends of non-nutritive sweeteners (NNS), water, and sugar-sweetened beverages (SSBs) sweetened with caloric sweeteners on postprandial blood GLUCAGON response in participants with TYPE 2 DIABETES, Table S15: GRADE assessments of COUPLING INTERVENTIONS examining the effect of non-nutritive sweetened beverages (NNS beverages) sweetened with individual or blends of non-nutritive sweeteners (NNS) and controls on postprandial blood GLUCOSE response in HEALTHY participants, Table S16: GRADE assessments of COUPLING INTERVENTIONS examining the effect of non-nutritive sweetened beverages (NNS beverages) sweetened with individual or blends of non-nutritive sweeteners (NNS) and controls on postprandial blood INSULIN response in HEALTHY participants, Table S17: OVERALL CINeMA assessments of DELAYED COUPLING INTERVENTIONS examining the effect of non-nutritive sweetened beverages (NNS beverages) sweetened with individual or blends of non-nutritive sweeteners (NNS) and water on postprandial blood GLUCOSE response in HEALTHY participants, Table S18: GRADE assessments of DELAYED COUPLING INTERVENTIONS examining the effect of non-nutritive sweetened beverages (NNS beverages) sweetened with individual or blends of non-nutritive sweeteners (NNS) and water on postprandial blood GLUCOSE response in participants with TYPE 2 DIABETES, Table S19: OVERALL CINeMA assessments of DELAYED COUPLING INTERVENTIONS examining the effect of non-nutritive sweetened beverages (NNS beverages) sweetened with individual or blends of non-nutritive sweeteners (NNS) and water on postprandial blood INSULIN response in HEALTHY participants, Table S20: GRADE assessments of DELAYED COUPLING INTERVENTIONS examining the effect of non-nutritive sweetened beverages (NNS beverages) sweetened with individual or blends of non-nutritive sweeteners (NNS) and water on postprandial blood INSULIN response in participants with TYPE 2 DIABETES, Table S21: OVERALL CINeMA assessments of DELAYED COUPLING INTERVENTIONS examining the effect of non-nutritive sweetened beverages (NNS beverages) sweetened with individual or blends of non-nutritive sweeteners (NNS) and water on postprandial blood GLP-1 response in HEALTHY participants, Table S22: GRADE assessments of DELAYED COUPLING INTERVENTIONS examining the effect of non-nutritive sweetened beverages (NNS beverages) sweetened with individual or blends of non-nutritive sweeteners (NNS) and water on postprandial blood GLP-1 response in participants with TYPE 2 DIABETES, Table S23: OVERALL CINeMA assessments of DELAYED COUPLING INTERVENTIONS examining the effect of non-nutritive sweetened beverages (NNS beverages) sweetened with individual or blends of non-nutritive sweeteners (NNS) and water on postprandial blood GIP response in HEALTHY participants, Table S24: GRADE assessments of DELAYED COUPLING INTERVENTIONS examining the effect of non-nutritive sweetened beverages (NNS beverages) sweetened with individual or blends of non-nutritive sweeteners (NNS) and water on postprandial blood GLUCAGON response in HEALTHY participants, Table S25: GRADE assessments for outcomes with single direct trial comparisons. Figure S1: Formulas used to compute incremental area under the curve (iAUC) for primary and secondary outcomes, Figure S2: Flow of literature, Figure S3: Individual (top) and summary (bottom) risk of bias assessments of studies with UNCOUPLING INTERVENTIONS, Figure S4: Individual (top) and summary (bottom) risk of bias assessment of studies with COUPLING INTERVENTIONS, Figure S5: Individual (top) and summary (bottom) risk of bias assessment of studies with DELAYED COUPLING INTERVENTIONS, Figure S6: Risk of bias assessment of UNCOUPLING INTERVENTIONS examining non-nutritive sweetened beverages (NNS beverages) sweetened with individual or blends of non-nutritive sweeteners (NNS), water, and sugar-sweetened beverages (SSBs) sweetened with caloric sweeteners on postprandial blood GLUCOSE response in HEALTHY participants, Figure S7: CINeMA output for the IMPRECISION domain of UNCOUPLING INTERVENTIONS examining non-nutritive sweetened beverages (NNS beverages) sweetened with individual or blends of non-nutritive sweeteners (NNS), water, and sugar-sweetened beverages (SSBs) sweetened with caloric sweeteners on postprandial blood GLUCOSE response in HEALTHY participants, Figure S8: CINeMA output for the HETEROGENEITY domain of UNCOUPLING INTERVENTIONS examining non-nutritive sweetened beverages (NNS beverages) sweetened with individual or blends of non-nutritive sweeteners (NNS), water, and sugar-sweetened beverages (SSBs) sweetened with caloric sweeteners on postprandial blood GLUCOSE response in HEALTHY participants, Figure S9: CINeMA output for the INCOHERENCE domain of UNCOUPLING INTERVENTIONS examining non-nutritive sweetened beverages (NNS beverages) sweetened with individual or blends of non-nutritive sweeteners (NNS), water, and sugar-sweetened beverages (SSBs) sweetened with caloric sweeteners on postprandial blood GLUCOSE response in HEALTHY participants, Figure S10: Risk of bias assessment of UNCOUPLING INTERVENTIONS examining non-nutritive sweetened beverages (NNS beverages) sweetened with individual or blends of non-nutritive sweeteners (NNS), water, and sugar-sweetened beverages (SSBs) sweetened with caloric sweeteners on postprandial blood GLUCOSE response in participants with TYPE 2 DIABETES, Figure S11: CINeMA output for the IMPRECISION domain of UNCOUPLING INTERVENTIONS examining non-nutritive sweetened beverages (NNS beverages) sweetened with individual or blends of non-nutritive sweeteners (NNS), water, and sugar-sweetened beverages (SSBs) sweetened with caloric sweeteners on postprandial blood GLUCOSE response in participants with TYPE 2 DIABETES, Figure S12: CINeMA output for the HETEROGENEITY domain of UNCOUPLING INTERVENTIONS examining non-nutritive sweetened beverages (NNS beverages) sweetened with individual or blends of non-nutritive sweeteners (NNS), water, and sugar-sweetened beverages (SSBs) sweetened with caloric sweeteners on postprandial blood GLUCOSE response in participants with TYPE 2 DIABETES, Figure S13: CINeMA output for the INCOHERENCE domain of UNCOUPLING INTERVENTIONS examining non-nutritive sweetened beverages (NNS beverages) sweetened with individual or blends of non-nutritive sweeteners (NNS), water, and sugar-sweetened beverages (SSBs) sweetened with caloric sweeteners on postprandial blood GLUCOSE response in participants with TYPE 2 DIABETES, Figure S14: Network plot and meta-analysis of UNCOUPLING INTERVENTIONS evaluating the effect of non-nutritive sweetened beverages (NNS beverages) sweetened single or blends of non-nutritive sweeteners (NNS), water, and sugar-sweetened beverages (SSBs) sweetened with caloric sweeteners on postprandial blood INSULIN response in HEALTHY participants, Figure S15: Risk of bias assessment of UNCOUPLING INTERVENTIONS evaluating the effect of non-nutritive sweetened beverages (NNS beverages) sweetened single or blends of non-nutritive sweeteners (NNS), water, and sugar-sweetened beverages (SSBs) sweetened with caloric sweeteners on postprandial blood INSULIN response in HEALTHY participants, Figure S16: CINeMA output for the IMPRECISION domain of UNCOUPLING INTERVENTIONS evaluating the effect of non-nutritive sweetened beverages (NNS beverages) sweetened single or blends of non-nutritive sweeteners (NNS), water, and sugar-sweetened beverages (SSBs) sweetened with caloric sweeteners on postprandial blood INSULIN response in HEALTHY participants, Figure S17: CINeMA output for the HETEROGENEITY domain of UNCOUPLING INTERVENTIONS evaluating the effect of non-nutritive sweetened beverages (NNS beverages) sweetened single or blends of non-nutritive sweeteners (NNS), water, and sugar-sweetened beverages (SSBs) sweetened with caloric sweeteners on postprandial blood INSULIN response in HEALTHY participants, Figure S18: CINeMA output for the INCOHERENCE domain of UNCOUPLING INTERVENTIONS evaluating the effect of non-nutritive sweetened beverages (NNS beverages) sweetened single or blends of non-nutritive sweeteners (NNS), water, and sugar-sweetened beverages (SSBs) sweetened with caloric sweeteners on postprandial blood INSULIN response in HEALTHY participants, Figure S19: Network plot and meta-analysis of UNCOUPLING INTERVENTIONS evaluating the effect of non-nutritive sweetened beverages (NNS beverages) sweetened single or blends of non-nutritive sweeteners (NNS), water, and sugar-sweetened beverages (SSBs) sweetened with caloric sweeteners on postprandial blood INSULIN response in participants with TYPE 2 DIABETES, Figure S20: Risk of bias assessment of UNCOUPLING INTERVENTIONS evaluating the effect of non-nutritive sweetened beverages (NNS beverages) sweetened single or blends of non-nutritive sweeteners (NNS), water, and sugar-sweetened beverages (SSBs) sweetened with caloric sweeteners on postprandial blood INSULIN response in participants with TYPE 2 DIABETES, Figure S21: CINeMA output for the IMPRECISION domain of UNCOUPLING INTERVENTIONS evaluating the effect of non-nutritive sweetened beverages (NNS beverages) sweetened single or blends of non-nutritive sweeteners (NNS), water, and sugar-sweetened beverages (SSBs) sweetened with caloric sweeteners on postprandial blood INSULIN response in participants with TYPE 2 DIABETES, Figure S22: CINeMA output for the HETEROGENEITY domain of UNCOUPLING INTERVENTIONS evaluating the effect of non-nutritive sweetened beverages (NNS beverages) sweetened single or blends of non-nutritive sweeteners (NNS), water, and sugar-sweetened beverages (SSBs) sweetened with caloric sweeteners on postprandial blood INSULIN response in participants with TYPE 2 DIABETES, Figure S23: CINeMA output for the INCOHERENCE domain of UNCOUPLING INTERVENTIONS evaluating the effect of non-nutritive sweetened beverages (NNS beverages) sweetened single or blends of non-nutritive sweeteners (NNS), water, and sugar-sweetened beverages (SSBs) sweetened with caloric sweeteners on postprandial blood INSULIN response in participants with TYPE 2 DIABETES, Figure S24: Network plot and meta-analysis of UNCOUPLING INTERVENTIONS evaluating the effect of non-nutritive sweetened beverages (NNS beverages) sweetened single or blends of non-nutritive sweeteners (NNS), water, and sugar-sweetened beverages (SSBs) sweetened with caloric sweeteners on postprandial blood GLP-1 response in HEALTHY participants, Figure S25: Risk of bias assessment of UNCOUPLING INTERVENTIONS evaluating the effect of non-nutritive sweetened beverages (NNS beverages) sweetened single or blends of non-nutritive sweeteners (NNS), water, and sugar-sweetened beverages (SSBs) sweetened with caloric sweeteners on postprandial blood GLP-1 response in HEALTHY participants, Figure S26: Network plot and meta-analysis of UNCOUPLING INTERVENTIONS evaluating the effect of non-nutritive sweetened beverages (NNS beverages) sweetened single or blends of non-nutritive sweeteners (NNS), water, and sugar-sweetened beverages (SSBs) sweetened with caloric sweeteners on postprandial blood GIP response in HEALTHY participants, Figure S27: Risk of bias assessment of UNCOUPLING INTERVENTIONS evaluating the effect of non-nutritive sweetened beverages (NNS beverages) sweetened single or blends of non-nutritive sweeteners (NNS), water, and sugar-sweetened beverages (SSBs) sweetened with caloric sweeteners on postprandial blood GIP response in HEALTHY participants, Figure S28: Network plot and meta-analysis of UNCOUPLING INTERVENTIONS evaluating the effect of non-nutritive sweetened beverages (NNS beverages) sweetened single or blends of non-nutritive sweeteners (NNS), water, and sugar-sweetened beverages (SSBs) sweetened with caloric sweeteners on postprandial blood GHRELIN response in HEALTHY participants, Figure S29: Risk of bias assessment of UNCOUPLING INTERVENTIONS evaluating the effect of non-nutritive sweetened beverages (NNS beverages) sweetened single or blends of non-nutritive sweeteners (NNS), water, and sugar-sweetened beverages (SSBs) sweetened with caloric sweeteners on postprandial blood GHRELIN response in HEALTHY participants, Figure S30: Network plot and meta-analysis of UNCOUPLING INTERVENTIONS evaluating the effect of non-nutritive sweetened beverages (NNS beverages) sweetened single or blends of non-nutritive sweeteners (NNS), water, and sugar-sweetened beverages (SSBs) sweetened with caloric sweeteners on postprandial blood GLUCAGON response in HEALTHY participants, Figure S31: Risk of bias assessment of UNCOUPLING INTERVENTIONS evaluating the effect of non-nutritive sweetened beverages (NNS beverages) sweetened single or blends of non-nutritive sweeteners (NNS), water, and sugar-sweetened beverages (SSBs) sweetened with caloric sweeteners on postprandial blood GLUCAGON response in HEALTHY participants, Figure S32: Network plot and meta-analysis of UNCOUPLING INTERVENTIONS evaluating the effect of non-nutritive sweetened beverages (NNS beverages) sweetened single or blends of non-nutritive sweeteners (NNS), water, and sugar-sweetened beverages (SSBs) sweetened with caloric sweeteners on postprandial blood GLUCAGON response in participants with TYPE 2 DIABETES, Figure S33: Risk of bias assessment of UNCOUPLING INTERVENTIONS evaluating the effect of non-nutritive sweetened beverages (NNS beverages) sweetened single or blends of non-nutritive sweeteners (NNS), water, and sugar-sweetened beverages (SSBs) sweetened with caloric sweeteners on postprandial blood GLUCAGON response in participants with TYPE 2 DIABETES, Figure S34: Risk of bias assessment of COUPLING INTERVENTIONS evaluating the effect of non-nutritive sweetened beverages (NNS beverages) sweetened single or blends of non-nutritive sweeteners (NNS) and controls on postprandial blood GLUCOSE response in HEALTHY participants, Figure S35: Network plot and meta-analysis of COUPLING INTERVENTIONS evaluating the effect of non-nutritive sweetened beverages (NNS beverages) sweetened single or blends of non-nutritive sweeteners (NNS) and controls on postprandial blood INSULIN response in HEALTHY participants, Figure S36: Risk of bias assessment of COUPLING INTERVENTIONS evaluating the effect of non-nutritive sweetened beverages (NNS beverages) sweetened single or blends of non-nutritive sweeteners (NNS) and controls on postprandial blood INSULIN response in HEALTHY participants, Figure S37: Risk of bias assessment of DELAYED COUPLING INTERVENTIONS evaluating the effect of non-nutritive sweetened beverages (NNS beverages) sweetened single or blends of non-nutritive sweeteners (NNS), water, and sugar-sweetened beverages (SSBs) sweetened with caloric sweeteners on postprandial blood GLUCOSE response in HEALTHY participants, Figure S38: CINeMA output for the IMPRECISION domain of DELAYED COUPLING INTERVENTIONS evaluating the effect of non-nutritive sweetened beverages (NNS beverages) sweetened single or blends of non-nutritive sweeteners (NNS), water, and sugar-sweetened beverages (SSBs) sweetened with caloric sweeteners on postprandial blood GLUCOSE response in HEALTHY participants, Figure S39: CINeMA output for the HETEROGENEITY domain of DELAYED COUPLING INTERVENTIONS evaluating the effect of non-nutritive sweetened beverages (NNS beverages) sweetened single or blends of non-nutritive sweeteners (NNS), water, and sugar-sweetened beverages (SSBs) sweetened with caloric sweeteners on postprandial blood GLUCOSE response in HEALTHY participants, Figure S40: CINeMA output for the INCOHERENCE domain of DELAYED COUPLING INTERVENTIONS evaluating the effect of non-nutritive sweetened beverages (NNS beverages) sweetened single or blends of non-nutritive sweeteners (NNS), water, and sugar-sweetened beverages (SSBs) sweetened with caloric sweeteners on postprandial blood GLUCOSE response in HEALTHY participants, Figure S41: Risk of bias assessment of DELAYED COUPLING INTERVENTIONS evaluating the effect of non-nutritive sweetened beverages (NNS beverages) sweetened single or blends of non-nutritive sweeteners (NNS), water, and sugar-sweetened beverages (SSBs) sweetened with caloric sweeteners on postprandial blood GLUCOSE response in participants with TYPE 2 DIABETES, Figure S42: Network plot and meta-analysis of DELAYED COUPLING INTERVENTIONS evaluating the effect of non-nutritive sweetened beverages (NNS beverages) sweetened single or blends of non-nutritive sweeteners (NNS), water, and sugar-sweetened beverages (SSBs) sweetened with caloric sweeteners on postprandial blood INSULIN response in HEALTHY participants, Figure S43: Risk of bias assessment of DELAYED COUPLING INTERVENTIONS evaluating the effect of non-nutritive sweetened beverages (NNS beverages) sweetened single or blends of non-nutritive sweeteners (NNS), water, and sugar-sweetened beverages (SSBs) sweetened with caloric sweeteners on postprandial blood INSULIN response in HEALTHY participants, Figure S44: CINeMA output for the IMPRECISION domain of DELAYED COUPLING INTERVENTIONS evaluating the effect of non-nutritive sweetened beverages (NNS beverages) sweetened single or blends of non-nutritive sweeteners (NNS), water, and sugar-sweetened beverages (SSBs) sweetened with caloric sweeteners on postprandial blood INSULIN response in HEALTHY participants, Figure S45: CINeMA output for the HETEROGENEITY domain of DELAYED COUPLING INTERVENTIONS evaluating the effect of non-nutritive sweetened beverages (NNS beverages) sweetened single or blends of non-nutritive sweeteners (NNS), water, and sugar-sweetened beverages (SSBs) sweetened with caloric sweeteners on postprandial blood INSULIN response in HEALTHY participants, Figure S46: CINeMA output for the INCOHERENCE domain of DELAYED COUPLING INTERVENTIONS evaluating the effect of non-nutritive sweetened beverages (NNS beverages) sweetened single or blends of non-nutritive sweeteners (NNS), water, and sugar-sweetened beverages (SSBs) sweetened with caloric sweeteners on postprandial blood INSULIN response in HEALTHY participants, Figure S47: Network plot and meta-analysis of DELAYED COUPLING INTERVENTIONS evaluating the effect of non-nutritive sweetened beverages (NNS beverages) sweetened single or blends of non-nutritive sweeteners (NNS), water, and sugar-sweetened beverages (SSBs) sweetened with caloric sweeteners on postprandial blood INSULIN response in participants with TYPE 2 DIABETES, Figure S48: Risk of bias assessment of DELAYED COUPLING INTERVENTIONS evaluating the effect of non-nutritive sweetened beverages (NNS beverages) sweetened single or blends of non-nutritive sweeteners (NNS), water, and sugar-sweetened beverages (SSBs) sweetened with caloric sweeteners on postprandial blood INSULIN response in participants with TYPE 2 DIABETES, Figure S49: Network plot and meta-analysis of DELAYED COUPLING INTERVENTIONS evaluating the effect of non-nutritive sweetened beverages (NNS beverages) sweetened single or blends of non-nutritive sweeteners (NNS), water, and sugar-sweetened beverages (SSBs) sweetened with caloric sweeteners on postprandial blood GLP-1 response in HEALTHY participants, Figure S50: Risk of bias assessment of DELAYED COUPLING INTERVENTIONS evaluating the effect of non-nutritive sweetened beverages (NNS beverages) sweetened single or blends of non-nutritive sweeteners (NNS), water, and sugar-sweetened beverages (SSBs) sweetened with caloric sweeteners on postprandial blood GLP-1 response in HEALTHY participants, Figure S51: CINeMA output for the IMPRECISION domain of DELAYED COUPLING INTERVENTIONS evaluating the effect of non-nutritive sweetened beverages (NNS beverages) sweetened single or blends of non-nutritive sweeteners (NNS), water, and sugar-sweetened beverages (SSBs) sweetened with caloric sweeteners on postprandial blood GLP-1 response in HEALTHY participants, Figure S52: CINeMA output for the HETEROGENEITY domain of DELAYED COUPLING INTERVENTIONS evaluating the effect of non-nutritive sweetened beverages (NNS beverages) sweetened single or blends of non-nutritive sweeteners (NNS), water, and sugar-sweetened beverages (SSBs) sweetened with caloric sweeteners on postprandial blood GLP-1 response in HEALTHY participants, Figure S53: CINeMA output for the INCOHERENCE domain of DELAYED COUPLING INTERVENTIONS evaluating the effect of non-nutritive sweetened beverages (NNS beverages) sweetened single or blends of non-nutritive sweeteners (NNS), water, and sugar-sweetened beverages (SSBs) sweetened with caloric sweeteners on postprandial blood GLP-1 response in HEALTHY participants, Figure S54: Network plot and meta-analysis of DELAYED COUPLING INTERVENTIONS evaluating the effect of non-nutritive sweetened beverages (NNS beverages) sweetened single or blends of non-nutritive sweeteners (NNS), water, and sugar-sweetened beverages (SSBs) sweetened with caloric sweeteners on postprandial blood GLP-1 response in participants with TYPE 2 DIABETES, Figure S55: Risk of bias assessment of DELAYED COUPLING INTERVENTIONS evaluating the effect of non-nutritive sweetened beverages (NNS beverages) sweetened single or blends of non-nutritive sweeteners (NNS), water, and sugar-sweetened beverages (SSBs) sweetened with caloric sweeteners on postprandial blood GLP-1 response in participants with TYPE 2 DIABETES, Figure S56. Network plot and meta-analysis of DELAYED COUPLING INTERVENTIONS evaluating the effect of non-nutritive sweetened beverages (NNS beverages) sweetened single or blends of non-nutritive sweeteners (NNS), water, and sugar-sweetened beverages (SSBs) sweetened with caloric sweeteners on postprandial blood GIP response in HEALTHY participants, Figure S57: Risk of bias assessment of DELAYED COUPLING INTERVENTIONS evaluating the effect of non-nutritive sweetened beverages (NNS beverages) sweetened single or blends of non-nutritive sweeteners (NNS), water, and sugar-sweetened beverages (SSBs) sweetened with caloric sweeteners on postprandial blood GIP response in HEALTHY participants, Figure S58: CINeMA output for the IMPRECISION domain of DELAYED COUPLING INTERVENTIONS evaluating the effect of non-nutritive sweetened beverages (NNS beverages) sweetened single or blends of non-nutritive sweeteners (NNS), water, and sugar-sweetened beverages (SSBs) sweetened with caloric sweeteners on postprandial blood GIP response in HEALTHY participants, Figure S59: CINeMA output for the HETEROGENEITY domain of DELAYED COUPLING INTERVENTIONS evaluating the effect of non-nutritive sweetened beverages (NNS beverages) sweetened single or blends of non-nutritive sweeteners (NNS), water, and sugar-sweetened beverages (SSBs) sweetened with caloric sweeteners on postprandial blood GIP response in HEALTHY participants, Figure S60: CINeMA output for the INCOHERENCE domain of DELAYED COUPLING INTERVENTIONS evaluating the effect of non-nutritive sweetened beverages (NNS beverages) sweetened single or blends of non-nutritive sweeteners (NNS), water, and sugar-sweetened beverages (SSBs) sweetened with caloric sweeteners on postprandial blood GIP response in HEALTHY participants, Figure S61: Network plot and meta-analysis of DELAYED COUPLING INTERVENTIONS evaluating the effect of non-nutritive sweetened beverages (NNS beverages) sweetened single or blends of non-nutritive sweeteners (NNS), water, and sugar-sweetened beverages (SSBs) sweetened with caloric sweeteners on postprandial blood GLUCAGON response in HEALTHY participants, Figure S62: Risk of bias assessment of DELAYED COUPLING INTERVENTIONS evaluating the effect of non-nutritive sweetened beverages (NNS beverages) sweetened single or blends of non-nutritive sweeteners (NNS), water, and sugar-sweetened beverages (SSBs) sweetened with caloric sweeteners on postprandial blood GLUCAGON response in HEALTHY participants. [75,76,77,78,79,80,81,82].
