# Supplementary material for: The Effect of Non-Nutritive Sweetened Beverages on Postprandial Glycemic and Endocrine Responses: A Systematic Review and Network Meta-Analysis
Source: Nutrients. 2023 Feb 20;15(4):1050. doi: 10.3390/nu15041050 (PMC9965414; doi:10.3390/nu15041050)
Supplement: Supplementary file 1 [file nutrients-15-01050-s001.zip › nutrients-2104449-supplementary.pdf]

SUPPLEMENTARY TABLES

Table S1. Search strategy 10
Table S2. PICOTS framework 11
Table S3. Characteristics of UNCOUPLING INTERVENTIONS 12
Table S4. Characteristics of COUPLING INTERVENTIONS 14
Table S5. Characteristics of DELAYED COUPLING INTERVENTIONS 15
Table S6. OVERALL CINeMA assessments of UNCOUPLING INTERVENTIONS examining the effect of non-nutritive sweetened beverages (NNS beverages) sweetened with individual or blends of non-nutritive sweeteners (NNS), water, and sugar-sweetened beverages (SSBs) sweetened with caloric sweeteners on postprandial blood GLUCOSE response in HEALTHY participants........... 17
Table S7. OVERALL CINeMA assessments of UNCOUPLING INTERVENTIONS examining the effect of non-nutritive sweetened beverages (NNS beverages) sweetened with individual or blends of non-nutritive sweeteners (NNS), water, and sugar-sweetened beverages (SSBs) sweetened with caloric sweeteners on postprandial blood GLUCOSE response in participants with TYPE 2 DIABETES 18
Table S8. OVERALL CINeMA assessments of UNCOUPLING INTERVENTIONS examining the effect of non-nutritive sweetened beverages (NNS beverages) sweetened with individual or blends of non-nutritive sweeteners (NNS), water, and sugar-sweetened beverages (SSBs) sweetened with caloric sweeteners on postprandial blood INSULIN response in HEALTHY participants............. 19
Table S9. OVERALL CINeMA assessments of UNCOUPLING INTERVENTIONS examining the effect of non-nutritive sweetened beverages (NNS beverages) sweetened with individual or blends of non-nutritive sweeteners (NNS), water, and sugar-sweetened beverages (SSBs) sweetened with caloric sweeteners on postprandial blood INSULIN response in participants with TYPE 2 DIABETES 20
Table S10. GRADE assessments of UNCOUPLING INTERVENTIONS examining the effect of non-nutritive sweetened beverages (NNS beverages) sweetened with individual or blends of non-nutritive sweeteners (NNS), water, and sugar-sweetened beverages (SSBs) sweetened with caloric sweeteners on postprandial blood GLP-1 response in HEALTHY participants 21
Table S11. GRADE assessments of UNCOUPLING INTERVENTIONS examining the effect of non-nutritive sweetened beverages (NNS beverages) sweetened with individual or blends of non-nutritive sweeteners (NNS), water, and sugar-sweetened beverages (SSBs) sweetened with caloric sweeteners on postprandial blood GIP response in HEALTHY participants 22
Table S12. GRADE assessments of UNCOUPLING INTERVENTIONS examining the effect of non-nutritive sweetened beverages (NNS beverages) sweetened with individual or blends of non-nutritive sweeteners (NNS), water, and sugar-sweetened beverages (SSBs) sweetened with caloric sweeteners on postprandial blood GHRELIN response in HEALTHY participants............ 23
Table S13. GRADE assessments of UNCOUPLING INTERVENTIONS examining the effect of non-nutritive sweetened beverages (NNS beverages) sweetened with individual or blends of non-nutritive sweeteners (NNS), water, and sugar-sweetened beverages (SSBs) sweetened with caloric sweeteners on postprandial blood GLUCAGON response in HEALTHY participants 24
Table S14. GRADE assessments of UNCOUPLING INTERVENTIONS examining the effect of non-nutritive sweetened beverages (NNS beverages) sweetened with individual or blends of non-nutritive sweeteners (NNS), water, and sugar-sweetened beverages (SSBs) sweetened with

caloric sweeteners on postprandial blood GLUCAGON response in participants with TYPE 2 DIABETES 25
Table S15. GRADE assessments of COUPLING INTERVENTIONS examining the effect of non-nutritive sweetened beverages (NNS beverages) sweetened with individual or blends of non-nutritive sweeteners (NNS) and controls on postprandial blood GLUCOSE response in HEALTHY participants 26
Table S16. GRADE assessments of COUPLING INTERVENTIONS examining the effect of non-nutritive sweetened beverages (NNS beverages) sweetened with individual or blends of non-nutritive sweeteners (NNS) and controls on postprandial blood INSULIN response in HEALTHY participants 27
Table S17. OVERALL CINeMA assessments of DELAYED COUPLING INTERVENTIONS examining the effect of non-nutritive sweetened beverages (NNS beverages) sweetened with individual or blends of non-nutritive sweeteners (NNS) and water on postprandial blood GLUCOSE response in HEALTHY participants........ 28
Table S18. GRADE assessments of DELAYED COUPLING INTERVENTIONS examining the effect of non-nutritive sweetened beverages (NNS beverages) sweetened with individual or blends of non-nutritive sweeteners (NNS) and water on postprandial blood GLUCOSE response in participants with TYPE 2 DIABETES........ 29
Table S19. OVERALL CINeMA assessments of DELAYED COUPLING INTERVENTIONS examining the effect of non-nutritive sweetened beverages (NNS beverages) sweetened with individual or blends of non-nutritive sweeteners (NNS) and water on postprandial blood INSULIN response in HEALTHY participants 30
Table S20. GRADE assessments of DELAYED COUPLING INTERVENTIONS examining the effect of non-nutritive sweetened beverages (NNS beverages) sweetened with individual or blends of non-nutritive sweeteners (NNS) and water on postprandial blood INSULIN response in participants with TYPE 2 DIABETES........ 31
Table S21. OVERALL CINeMA assessments of DELAYED COUPLING INTERVENTIONS examining the effect of non-nutritive sweetened beverages (NNS beverages) sweetened with individual or blends of non-nutritive sweeteners (NNS) and water on postprandial blood GLP-1 response in HEALTHY participants 32
Table S22. GRADE assessments of DELAYED COUPLING INTERVENTIONS examining the effect of non-nutritive sweetened beverages (NNS beverages) sweetened with individual or blends of non-nutritive sweeteners (NNS) and water on postprandial blood GLP-1 response in participants with TYPE 2 DIABETES........ 33
Table S23. OVERALL CINeMA assessments of DELAYED COUPLING INTERVENTIONS examining the effect of non-nutritive sweetened beverages (NNS beverages) sweetened with individual or blends of non-nutritive sweeteners (NNS) and water on postprandial blood GIP response in HEALTHY participants 34
Table S24. GRADE assessments of DELAYED COUPLING INTERVENTIONS examining the effect of non-nutritive sweetened beverages (NNS beverages) sweetened with individual or blends of non-nutritive sweeteners (NNS) and water on postprandial blood GLUCAGON response in HEALTHY participants 35
Table S25. GRADE assessments for outcomes with single direct trial comparisons........ 36

SUPPLEMENTARY FIGURES

Figure S1. Formulas used to compute incremental area under the curve (iAUC) for primary and secondary outcomes 37
Figure S2. Flow of literature 38
Figure S3. Individual (top) and summary (bottom) risk of bias assessments of studies with UNCOUPLING INTERVENTIONS 39
Figure S4. Individual (top) and summary (bottom) risk of bias assessment of studies with COUPLING INTERVENTIONS 40
Figure S5. Individual (top) and summary (bottom) risk of bias assessment of studies with DELAYED COUPLING INTERVENTIONS 41
Figure S6. Risk of bias assessment of UNCOUPLING INTERVENTIONS examining non-nutritive sweetened beverages (NNS beverages) sweetened with individual or blends of non-nutritive sweeteners (NNS), water, and sugar-sweetened beverages (SSBs) sweetened with caloric sweeteners on postprandial blood GLUCOSE response in HEALTHY participants 42
Figure S7. CINeMA output for the IMPRECISION domain of UNCOUPLING INTERVENTIONS examining non-nutritive sweetened beverages (NNS beverages) sweetened with individual or blends of non-nutritive sweeteners (NNS), water, and sugar-sweetened beverages (SSBs) sweetened with caloric sweeteners on postprandial blood GLUCOSE response in HEALTHY participants 44
Figure S8. CINeMA output for the HETEROGENEITY domain of UNCOUPLING INTERVENTIONS examining non-nutritive sweetened beverages (NNS beverages) sweetened with individual or blends of non-nutritive sweeteners (NNS), water, and sugar-sweetened beverages (SSBs) sweetened with caloric sweeteners on postprandial blood GLUCOSE response in HEALTHY participants 47
Figure S9. CINeMA output for the INCOHERENCE domain of UNCOUPLING INTERVENTIONS examining non-nutritive sweetened beverages (NNS beverages) sweetened with individual or blends of non-nutritive sweeteners (NNS), water, and sugar-sweetened beverages (SSBs) sweetened with caloric sweeteners on postprandial blood GLUCOSE response in HEALTHY participants 51
Figure S10. Risk of bias assessment of UNCOUPLING INTERVENTIONS examining non-nutritive sweetened beverages (NNS beverages) sweetened with individual or blends of non-nutritive sweeteners (NNS), water, and sugar-sweetened beverages (SSBs) sweetened with caloric sweeteners on postprandial blood GLUCOSE response in participants with TYPE 2 DIABETES .. 53
Figure S11. CINeMA output for the IMPRECISION domain of UNCOUPLING INTERVENTIONS examining non-nutritive sweetened beverages (NNS beverages) sweetened with individual or blends of non-nutritive sweeteners (NNS), water, and sugar-sweetened beverages (SSBs) sweetened with caloric sweeteners on postprandial blood GLUCOSE response in participants with TYPE 2 DIABETES 54
Figure S12. CINeMA output for the HETEROGENEITY domain of UNCOUPLING INTERVENTIONS examining non-nutritive sweetened beverages (NNS beverages) sweetened with individual or blends of non-nutritive sweeteners (NNS), water, and sugar-sweetened beverages (SSBs) sweetened with caloric sweeteners on postprandial blood GLUCOSE response in participants with TYPE 2 DIABETES 55

Figure S13. CINeMA output for the INCOHERENCE domain of UNCOUPLING INTERVENTIONS examining non-nutritive sweetened beverages (NNS beverages) sweetened with individual or blends of non-nutritive sweeteners (NNS), water, and sugar-sweetened beverages (SSBs) sweetened with caloric sweeteners on postprandial blood GLUCOSE response in participants with TYPE 2 DIABETES 56
Figure S14. Network plot and meta-analysis of UNCOUPLING INTERVENTIONS evaluating the effect of non-nutritive sweetened beverages (NNS beverages) sweetened single or blends of non-nutritive sweeteners (NNS), water, and sugar-sweetened beverages (SSBs) sweetened with caloric sweeteners on postprandial blood INSULIN response in HEALTHY participants 57
Figure S15. Risk of bias assessment of UNCOUPLING INTERVENTIONS evaluating the effect of non-nutritive sweetened beverages (NNS beverages) sweetened single or blends of non-nutritive sweeteners (NNS), water, and sugar-sweetened beverages (SSBs) sweetened with caloric sweeteners on postprandial blood INSULIN response in HEALTHY participants 59
Figure S16. CINeMA output for the IMPRECISION domain of UNCOUPLING INTERVENTIONS evaluating the effect of non-nutritive sweetened beverages (NNS beverages) sweetened single or blends of non-nutritive sweeteners (NNS), water, and sugar-sweetened beverages (SSBs) sweetened with caloric sweeteners on postprandial blood INSULIN response in HEALTHY participants 61
Figure S17. CINeMA output for the HETEROGENEITY domain of UNCOUPLING INTERVENTIONS evaluating the effect of non-nutritive sweetened beverages (NNS beverages) sweetened single or blends of non-nutritive sweeteners (NNS), water, and sugar-sweetened beverages (SSBs) sweetened with caloric sweeteners on postprandial blood INSULIN response in HEALTHY participants 64
Figure S18. CINeMA output for the INCOHERENCE domain of UNCOUPLING INTERVENTIONS evaluating the effect of non-nutritive sweetened beverages (NNS beverages) sweetened single or blends of non-nutritive sweeteners (NNS), water, and sugar-sweetened beverages (SSBs) sweetened with caloric sweeteners on postprandial blood INSULIN response in HEALTHY participants 67
Figure S19. Network plot and meta-analysis of UNCOUPLING INTERVENTIONS evaluating the effect of non-nutritive sweetened beverages (NNS beverages) sweetened single or blends of non-nutritive sweeteners (NNS), water, and sugar-sweetened beverages (SSBs) sweetened with caloric sweeteners on postprandial blood INSULIN response in participants with TYPE 2 DIABETES 70
Figure S20. Risk of bias assessment of UNCOUPLING INTERVENTIONS evaluating the effect of non-nutritive sweetened beverages (NNS beverages) sweetened single or blends of non-nutritive sweeteners (NNS), water, and sugar-sweetened beverages (SSBs) sweetened with caloric sweeteners on postprandial blood INSULIN response in participants with TYPE 2 DIABETES 71
Figure S21. CINeMA output for the IMPRECISION domain of UNCOUPLING INTERVENTIONS evaluating the effect of non-nutritive sweetened beverages (NNS beverages) sweetened single or blends of non-nutritive sweeteners (NNS), water, and sugar-sweetened beverages (SSBs) sweetened with caloric sweeteners on postprandial blood INSULIN response in participants with TYPE 2 DIABETES 72

Figure S22. CINeMA output for the HETEROGENEITY domain of UNCOUPLING INTERVENTIONS evaluating the effect of non-nutritive sweetened beverages (NNS beverages) sweetened single or blends of non-nutritive sweeteners (NNS), water, and sugar-sweetened beverages (SSBs) sweetened with caloric sweeteners on postprandial blood INSULIN response in participants with TYPE 2 DIABETES 73
Figure S23. CINeMA output for the INCOHERENCE domain of UNCOUPLING INTERVENTIONS evaluating the effect of non-nutritive sweetened beverages (NNS beverages) sweetened single or blends of non-nutritive sweeteners (NNS), water, and sugar-sweetened beverages (SSBs) sweetened with caloric sweeteners on postprandial blood INSULIN response in participants with TYPE 2 DIABETES 74
Figure S24. Network plot and meta-analysis of UNCOUPLING INTERVENTIONS evaluating the effect of non-nutritive sweetened beverages (NNS beverages) sweetened single or blends of non-nutritive sweeteners (NNS), water, and sugar-sweetened beverages (SSBs) sweetened with caloric sweeteners on postprandial blood GLP-1 response in HEALTHY participants 75
Figure S25. Risk of bias assessment of UNCOUPLING INTERVENTIONS evaluating the effect of non-nutritive sweetened beverages (NNS beverages) sweetened single or blends of non-nutritive sweeteners (NNS), water, and sugar-sweetened beverages (SSBs) sweetened with caloric sweeteners on postprandial blood GLP-1 response in HEALTHY participants 76
Figure S26. Network plot and meta-analysis of UNCOUPLING INTERVENTIONS evaluating the effect of non-nutritive sweetened beverages (NNS beverages) sweetened single or blends of non-nutritive sweeteners (NNS), water, and sugar-sweetened beverages (SSBs) sweetened with caloric sweeteners on postprandial blood GIP response in HEALTHY participants 77
Figure S27. Risk of bias assessment of UNCOUPLING INTERVENTIONS evaluating the effect of non-nutritive sweetened beverages (NNS beverages) sweetened single or blends of non-nutritive sweeteners (NNS), water, and sugar-sweetened beverages (SSBs) sweetened with caloric sweeteners on postprandial blood GIP response in HEALTHY participants 78
Figure S28. Network plot and meta-analysis of UNCOUPLING INTERVENTIONS evaluating the effect of non-nutritive sweetened beverages (NNS beverages) sweetened single or blends of non-nutritive sweeteners (NNS), water, and sugar-sweetened beverages (SSBs) sweetened with caloric sweeteners on postprandial blood GHRELIN response in HEALTHY participants........ 79
Figure S29. Risk of bias assessment of UNCOUPLING INTERVENTIONS evaluating the effect of non-nutritive sweetened beverages (NNS beverages) sweetened single or blends of non-nutritive sweeteners (NNS), water, and sugar-sweetened beverages (SSBs) sweetened with caloric sweeteners on postprandial blood GHRELIN response in HEALTHY participants........ 80
Figure S30. Network plot and meta-analysis of UNCOUPLING INTERVENTIONS evaluating the effect of non-nutritive sweetened beverages (NNS beverages) sweetened single or blends of non-nutritive sweeteners (NNS), water, and sugar-sweetened beverages (SSBs) sweetened with caloric sweeteners on postprandial blood GLUCAGON response in HEALTHY participants 81
Figure S31. Risk of bias assessment of UNCOUPLING INTERVENTIONS evaluating the effect of non-nutritive sweetened beverages (NNS beverages) sweetened single or blends of non-nutritive sweeteners (NNS), water, and sugar-sweetened beverages (SSBs) sweetened with caloric sweeteners on postprandial blood GLUCAGON response in HEALTHY participants 82
Figure S32. Network plot and meta-analysis of UNCOUPLING INTERVENTIONS evaluating the effect of non-nutritive sweetened beverages (NNS beverages) sweetened single or blends of

non-nutritive sweeteners (NNS), water, and sugar-sweetened beverages (SSBs) sweetened with caloric sweeteners on postprandial blood GLUCAGON response in participants with TYPE 2 DIABETES 83
Figure S33. Risk of bias assessment of UNCOUPLING INTERVENTIONS evaluating the effect of non-nutritive sweetened beverages (NNS beverages) sweetened single or blends of non-nutritive sweeteners (NNS), water, and sugar-sweetened beverages (SSBs) sweetened with caloric sweeteners on postprandial blood GLUCAGON response in participants with TYPE 2 DIABETES 84
Figure S34. Risk of bias assessment of COUPLING INTERVENTIONS evaluating the effect of non-nutritive sweetened beverages (NNS beverages) sweetened single or blends of non-nutritive sweeteners (NNS) and controls on postprandial blood GLUCOSE response in HEALTHY participants. 85
Figure S35. Network plot and meta-analysis of COUPLING INTERVENTIONS evaluating the effect of non-nutritive sweetened beverages (NNS beverages) sweetened single or blends of non-nutritive sweeteners (NNS) and controls on postprandial blood INSULIN response in HEALTHY participants. 86
Figure S36. Risk of bias assessment of COUPLING INTERVENTIONS evaluating the effect of non-nutritive sweetened beverages (NNS beverages) sweetened single or blends of non-nutritive sweeteners (NNS) and controls on postprandial blood INSULIN response in HEALTHY participants. 87
Figure S37. Risk of bias assessment of DELAYED COUPLING INTERVENTIONS evaluating the effect of non-nutritive sweetened beverages (NNS beverages) sweetened single or blends of non-nutritive sweeteners (NNS), water, and sugar-sweetened beverages (SSBs) sweetened with caloric sweeteners on postprandial blood GLUCOSE response in HEALTHY participants............ 88
Figure S38. CINeMA output for the IMPRECISION domain of DELAYED COUPLING INTERVENTIONS evaluating the effect of non-nutritive sweetened beverages (NNS beverages) sweetened single or blends of non-nutritive sweeteners (NNS), water, and sugar-sweetened beverages (SSBs) sweetened with caloric sweeteners on postprandial blood GLUCOSE response in HEALTHY participants.. 89
Figure S39. CINeMA output for the HETEROGENEITY domain of DELAYED COUPLING INTERVENTIONS evaluating the effect of non-nutritive sweetened beverages (NNS beverages) sweetened single or blends of non-nutritive sweeteners (NNS), water, and sugar-sweetened beverages (SSBs) sweetened with caloric sweeteners on postprandial blood GLUCOSE response in HEALTHY participants.. 91
Figure S40. CINeMA output for the INCOHERENCE domain of DELAYED COUPLING INTERVENTIONS evaluating the effect of non-nutritive sweetened beverages (NNS beverages) sweetened single or blends of non-nutritive sweeteners (NNS), water, and sugar-sweetened beverages (SSBs) sweetened with caloric sweeteners on postprandial blood GLUCOSE response in HEALTHY participants.. 93
Figure S41. Risk of bias assessment of DELAYED COUPLING INTERVENTIONS evaluating the effect of non-nutritive sweetened beverages (NNS beverages) sweetened single or blends of non-nutritive sweeteners (NNS), water, and sugar-sweetened beverages (SSBs) sweetened with caloric sweeteners on postprandial blood GLUCOSE response in participants with TYPE 2 DIABETES 94

Figure S42. Network plot and meta-analysis of DELAYED COUPLING INTERVENTIONS evaluating the effect of non-nutritive sweetened beverages (NNS beverages) sweetened single or blends of non-nutritive sweeteners (NNS), water, and sugar-sweetened beverages (SSBs) sweetened with caloric sweeteners on postprandial blood INSULIN response in HEALTHY participants...... 95
Figure S43. Risk of bias assessment of DELAYED COUPLING INTERVENTIONS evaluating the effect of non-nutritive sweetened beverages (NNS beverages) sweetened single or blends of non-nutritive sweeteners (NNS), water, and sugar-sweetened beverages (SSBs) sweetened with caloric sweeteners on postprandial blood INSULIN response in HEALTHY participants............. 97
Figure S44. CINeMA output for the IMPRECISION domain of DELAYED COUPLING INTERVENTIONS evaluating the effect of non-nutritive sweetened beverages (NNS beverages) sweetened single or blends of non-nutritive sweeteners (NNS), water, and sugar-sweetened beverages (SSBs) sweetened with caloric sweeteners on postprandial blood INSULIN response in HEALTHY participants.. 98
Figure S45. CINeMA output for the HETEROGENEITY domain of DELAYED COUPLING INTERVENTIONS evaluating the effect of non-nutritive sweetened beverages (NNS beverages) sweetened single or blends of non-nutritive sweeteners (NNS), water, and sugar-sweetened beverages (SSBs) sweetened with caloric sweeteners on postprandial blood INSULIN response in HEALTHY participants.. 100
Figure S46. CINeMA output for the INCOHERENCE domain of DELAYED COUPLING INTERVENTIONS evaluating the effect of non-nutritive sweetened beverages (NNS beverages) sweetened single or blends of non-nutritive sweeteners (NNS), water, and sugar-sweetened beverages (SSBs) sweetened with caloric sweeteners on postprandial blood INSULIN response in HEALTHY participants.. 101
Figure S47. Network plot and meta-analysis of DELAYED COUPLING INTERVENTIONS evaluating the effect of non-nutritive sweetened beverages (NNS beverages) sweetened single or blends of non-nutritive sweeteners (NNS), water, and sugar-sweetened beverages (SSBs) sweetened with caloric sweeteners on postprandial blood INSULIN response in participants with TYPE 2 DIABETES.. 102
Figure S48. Risk of bias assessment of DELAYED COUPLING INTERVENTIONS evaluating the effect of non-nutritive sweetened beverages (NNS beverages) sweetened single or blends of non-nutritive sweeteners (NNS), water, and sugar-sweetened beverages (SSBs) sweetened with caloric sweeteners on postprandial blood INSULIN response in participants with TYPE 2 DIABETES.. 103
Figure S49. Network plot and meta-analysis of DELAYED COUPLING INTERVENTIONS evaluating the effect of non-nutritive sweetened beverages (NNS beverages) sweetened single or blends of non-nutritive sweeteners (NNS), water, and sugar-sweetened beverages (SSBs) sweetened with caloric sweeteners on postprandial blood GLP-1 response in HEALTHY participants 104
Figure S50. Risk of bias assessment of DELAYED COUPLING INTERVENTIONS evaluating the effect of non-nutritive sweetened beverages (NNS beverages) sweetened single or blends of non-nutritive sweeteners (NNS), water, and sugar-sweetened beverages (SSBs) sweetened with caloric sweeteners on postprandial blood GLP-1 response in HEALTHY participants 106
Figure S51. CINeMA output for the IMPRECISION domain of DELAYED COUPLING INTERVENTIONS evaluating the effect of non-nutritive sweetened beverages (NNS beverages) sweetened single or blends of non-nutritive sweeteners (NNS), water, and sugar-sweetened

beverages (SSBs) sweetened with caloric sweeteners on postprandial blood GLP-1 response in HEALTHY participants 107
Figure S52. CINeMA output for the HETEROGENEITY domain of DELAYED COUPLING INTERVENTIONS evaluating the effect of non-nutritive sweetened beverages (NNS beverages) sweetened single or blends of non-nutritive sweeteners (NNS), water, and sugar-sweetened beverages (SSBs) sweetened with caloric sweeteners on postprandial blood GLP-1 response in HEALTHY participants 109
Figure S53. CINeMA output for the INCOHERENCE domain of DELAYED COUPLING INTERVENTIONS evaluating the effect of non-nutritive sweetened beverages (NNS beverages) sweetened single or blends of non-nutritive sweeteners (NNS), water, and sugar-sweetened beverages (SSBs) sweetened with caloric sweeteners on postprandial blood GLP-1 response in HEALTHY participants 110
Figure S54. Network plot and meta-analysis of DELAYED COUPLING INTERVENTIONS evaluating the effect of non-nutritive sweetened beverages (NNS beverages) sweetened single or blends of non-nutritive sweeteners (NNS), water, and sugar-sweetened beverages (SSBs) sweetened with caloric sweeteners on postprandial blood GLP-1 response in participants with TYPE 2 DIABETES 111
Figure S55. Risk of bias assessment of DELAYED COUPLING INTERVENTIONS evaluating the effect of non-nutritive sweetened beverages (NNS beverages) sweetened single or blends of non-nutritive sweeteners (NNS), water, and sugar-sweetened beverages (SSBs) sweetened with caloric sweeteners on postprandial blood GLP-1 response in participants with TYPE 2 DIABETES 112
Figure S56. Network plot and meta-analysis of DELAYED COUPLING INTERVENTIONS evaluating the effect of non-nutritive sweetened beverages (NNS beverages) sweetened single or blends of non-nutritive sweeteners (NNS), water, and sugar-sweetened beverages (SSBs) sweetened with caloric sweeteners on postprandial blood GIP response in HEALTHY participants 113
Figure S57. Risk of bias assessment of DELAYED COUPLING INTERVENTIONS evaluating the effect of non-nutritive sweetened beverages (NNS beverages) sweetened single or blends of non-nutritive sweeteners (NNS), water, and sugar-sweetened beverages (SSBs) sweetened with caloric sweeteners on postprandial blood GIP response in HEALTHY participants 114
Figure S58. CINeMA output for the IMPRECISION domain of DELAYED COUPLING INTERVENTIONS evaluating the effect of non-nutritive sweetened beverages (NNS beverages) sweetened single or blends of non-nutritive sweeteners (NNS), water, and sugar-sweetened beverages (SSBs) sweetened with caloric sweeteners on postprandial blood GIP response in HEALTHY participants 115
Figure S59. CINeMA output for the HETEROGENEITY domain of DELAYED COUPLING INTERVENTIONS evaluating the effect of non-nutritive sweetened beverages (NNS beverages) sweetened single or blends of non-nutritive sweeteners (NNS), water, and sugar-sweetened beverages (SSBs) sweetened with caloric sweeteners on postprandial blood GIP response in HEALTHY participants 116
Figure S60. CINeMA output for the INCOHERENCE domain of DELAYED COUPLING INTERVENTIONS evaluating the effect of non-nutritive sweetened beverages (NNS beverages) sweetened single or blends of non-nutritive sweeteners (NNS), water, and sugar-sweetened

beverages (SSBs) sweetened with caloric sweeteners on postprandial blood GIP response in HEALTHY participants 117
Figure S61. Network plot and meta-analysis of DELAYED COUPLING INTERVENTIONS evaluating the effect of non-nutritive sweetened beverages (NNS beverages) sweetened single or blends of non-nutritive sweeteners (NNS), water, and sugar-sweetened beverages (SSBs) sweetened with caloric sweeteners on postprandial blood GLUCAGON response in HEALTHY participants 118
Figure S62. Risk of bias assessment of DELAYED COUPLING INTERVENTIONS evaluating the effect of non-nutritive sweetened beverages (NNS beverages) sweetened single or blends of non-nutritive sweeteners (NNS), water, and sugar-sweetened beverages (SSBs) sweetened with caloric sweeteners on postprandial blood GLUCAGON response in HEALTHY participants 119

Table S1. *Search strategy*

MEDLINE	EMBASE	Cochrane Central Register of Controlled Trials
1. exp Non-Nutritive Sweeteners/	1. exp nonnutritive sweetener/	1. exp Non-Nutritive Sweeteners/
2. exp Aspartame/	2. exp aspartame/	2. exp Aspartame/
3. aspartame.mp.	3. aspartame.mp.	3. aspartame.mp.
4. exp Saccharin/	4. exp saccharin/	4. exp Saccharin/
5. saccharin.mp.	5. saccharin.mp.	5. saccharin.mp.
6. sucralose.mp.	6. exp sucralose/	6. sucralose.mp.
7. acesulfame.mp.	7. sucralose.mp.	7. acesulfame.mp.
8. neotame.mp.	8. exp acesulfame/	8. neotame.mp.
9. advantame.mp.	9. acesulfame.mp.	9. advantame.mp.
10. alitame.mp.	10. exp neotame/	10. exp Cyclamates/
11. exp Cyclamates/	11. neotame.mp.	11. cyclamate.mp.
12. cyclamate.mp.	12. advantame.mp.	12. exp Stevia/
13. exp Stevia/	13. exp alitame/	13. stevia.mp.
14. stevia.mp.	14. alitame.mp.	14. steviol glycoside.mp.
15. steviol glycoside.mp.	15. exp cyclamate sodium/	15. rebaudioside.mp.
16. rebaudioside.mp.	16. cyclamate.mp.	16. stevioside.mp.
17. stevioside.mp.	17. exp Stevia/	17. luo han guo.mp.
18. siraitia grosvenorii.mp.	18. stevia.mp.	18. monk fruit.mp.
19. luo han guo.mp.	19. steviol glycoside.mp.	19. exp Postprandial Period/
20. monk fruit.mp.	20. rebaudioside.mp.	20. postprandial.mp.
21. exp Postprandial Period/	21. stevioside.mp.	21. acute.mp.
22. postprandial.mp.	22. exp Siraitia grosvenorii/	22. exp Blood Glucose/
23. acute.mp.	23. luo han guo.mp.	23. exp Glucose Tolerance Test/
24. exp Blood Glucose/	24. monk fruit.mp.	24. glucose.mp.
25. exp Glucose Tolerance Test/	25. exp postprandial state/	25. glycemia.mp.
26. glucose.mp.	26. postprandial.mp.	26. glycaemia.mp.
27. glycemia.mp.	27. acute.mp.	27. glycemic.mp.
28. glycaemia.mp.	28. exp glucose blood level/	28. glycaemic.mp.
29. glycemic.mp.	29. exp glucose tolerance test/	29. exp Insulin/
30. glycaemic.mp.	30. glucose.mp.	30. insulin.mp.
31. exp Insulin/	31. glycemia.mp.	31. exp Glucagon-Like Peptide 1/
32. insulin.mp.	32. glycaemia.mp.	32. glucagon-like peptide 1.mp.
33. exp Glucagon-Like Peptide 1/	33. glycemic.mp.	33. GLP-1.mp.
34. glucagon-like peptide 1.mp.	34. glycaemic.mp.	34. exp Peptide YY/
35. glp-1.mp.	35. exp insulin/	35. peptide yy.mp.
36. exp Peptide YY/	36. insulin.mp.	36. PYY.mp.
37. peptide yy.mp.	37. exp glucagon like peptide 1/	37. exp Gastric Inhibitory Polypeptide/
38. PYY.mp.	38. glucagon-like peptide 1.mp.	38. gastric inhibitory polypeptide.mp.
39. exp Gastric Inhibitory Polypeptide/	39. glp-1.mp.	39. GIP.mp.
40. gastric inhibitory polypeptide.mp.	40. exp peptide YY/	40. exp Ghrelin/
41. GIP.mp.	41. peptide yy.mp.	41. ghrelin.mp.
42. exp Ghrelin/	42. PYY.mp.	42. exp Leptin/
43. ghrelin.mp.	43. exp gastric inhibitory polypeptide/	43. leptin.mp.
44. exp Leptin/	44. gastric inhibitory polypeptide.mp.	44. exp Glucagon/
45. leptin.mp.	45. GIP.mp.	45. glucagon.mp.
46. exp Glucagon/	46. exp ghrelin/	46. or/1-18
47. glucagon.mp.	47. ghrelin.mp.	47. or/19-45
48. or/1-20	48. exp leptin/	48. 46 and 47
49. or/21-47	49. leptin.mp.	
50. 48 and 49	50. exp glucagon/	
51. limit 50 to animals	51. glucagon.mp.	
52. 50 not 51	52. or/1-24	
	53. or/25-51	
	54. 52 and 53	
	55. limit 54 to animals	
	56. 54 not 55	

Table S2. PICOTS framework

Participants	Intervention	Comparison	Outcomes	Time	Study Design
Males and females of all health backgrounds and ages (i.e., adults and children), excluding pregnant or breastfeeding women	NNS beverages sweetened single or blends of NNS, water, or SSBs sweetened with caloric sugars	NNS beverages sweetened single or blends of NNS, water, or SSBs sweetened with caloric sugars	Glucose iAUC<br>Insulin iAUC<br>GLP-1 iAUC<br>PYY iAUC<br>GIP iAUC<br>Ghrelin iAUC<br>Glucagon iAUC	2 hours	Acute, randomized and non-randomized, controlled, feeding trials

NNS beverage, non-nutritive sweetened beverage; NNS, non-nutritive sweetener; SSBs, sugar-sweetened beverage; iAUC, incremental area under the curve; GLP-1, glucagon-like peptide 1; PYY, peptide YY; GIP, gastric inhibitory peptide. NNS include, aspartame, acesulfame potassium (Ace-K), luo han guo (monk) fruit extract, neotame, saccharin, stevia, sucralose and advantame (1)

Table S3. *Characteristics of UNCOUPLING INTERVENTIONS*

First author, year (reference)	Arm description	Study Design	Participants	Health status	Age, years	BMI, kg/m²	Duration (mins)	Outcomes extracted	Funding source	Country
**Shigeta, 1985 (2)**		NRS	15 (10M, 5F)	DM2	62.9 (12.8)	-	180	Glucose	-	Japan
Aspartame	225mg oral aspartame loading test							Insulin		
Glucose	75g OGTT									
**Okuno, 1986 (3)**		NRS	7	Healthy	46.7 (31-60)	-	180	Glucose	-	Japan
Aspartame	500mg aspartame in 300ml water		6	DM2	47.3 (18-64)			Insulin		
Glucose	100g glucose in 300ml water		8	DM2	50.3 (38-60)			Glucagon		
			8	DM2	46.5 (46-54)					
**Horwitz, 1988 (4)**		RCT	12 (0M, 12F)	Healthy	28.0 (8.0)	22.5	180	Glucose	Industry	USA
Aspartame	300ml Kool-Aid sweetened with 400mg aspartame		10 (5M, 5F)	DM2	57.0 (8.0)	33.7		Insulin		
Saccharin	300ml Kool-Aid sweetened with 135mg saccharin							Glucagon		
Water	300ml unsweetened Kool-Aid									
**Moller, 1991 (5)**		RCT	6 (6M, 0F)	Healthy	29 (22-37)	-	240	Glucose	Agency	Denmark
Aspartame	1g aspartame dissolved in 200ml water							Insulin		
Water	200ml water									
**Härtel, 1993 (6)**		NRS	14 (6M, 8F)	Healthy	19-52	-	120	Glucose	-	Germany
Aspartame	165mg aspartame in 330ml water							Insulin		
Ace-K	165mg ace-K in 330ml water									
Cyclamate	800mg cyclamate in 330ml water									
Saccharin	75mg saccharin in 330ml water									
Sucrose	33g sucrose in 330ml water									
Water	330ml water									
**Nguyen, 1998 (7)**		RCT	7 (4M, 3F)	Healthy	30-47	-	120	Glucose	-	USA
Aspartame	250mg aspartame dissolved in 250ml water							Insulin		
Glucose	75g glucose dissolved in 250ml water									
**Coppola, 2004 (8)**		RCT	20 (10M, 10F)	Healthy	68.0 (8.0)	26.4 (3.0)	120	Glucose	-	Italy
Aspartame	250mg aspartame dissolved in 250ml water		20 (10M, 10F)	IGT	69.0 (11.0)	27.0 (3.6)		Insulin		
Glucose	75g glucose dissolved in 250ml water									
**Berlin, 2005 (9)**		RCT	12 (7M, 5F)	Healthy	28.6 (7.9)	21.2 (0.4)	300	Glucose	Agency	France
Aspartame	0.6g aspartame in 200ml water							Insulin		
Glucose	75g glucose in 200ml water									
**Ford, 2011 (10)**		RCT	8 (1M, 7F)	Healthy	22-27	18.8-23.9	120	Glucose	Agency	UK
Sucralose	50ml sucralose (0.083% w/v, 2mmol/L)							Insulin		
Water	50ml water							GLP-1		
								PYY		
**Maersk, 2012 (11)**		RCT	24 (12M, 12F)	Healthy	33.5 (9.2)	31.4 (3.1)	240	Ghrelin	Agency	Denmark
Aspartame	500ml aspartame-sweetened diet cola (Coca Cola)							GLP-1		
Sucrose	500ml sucrose-sweetened regular cola (Coca Cola)							GIP		
Water	500ml bottled still water (Aqua d'or mineral water)									
**Hazali, 2014 (12)**		NRS	32 (4M, 28F)	Healthy	21.3 (1.1)	21.6 (3.3)	120	Glucose	Agency	Malaysia
Stevia (500mg)	500mg stevia in 100ml water									
Stevia (1000mg)	1000mg stevia in 100ml water									
Sucrose	20g sucrose in 100ml water									
**Bloomer, 2016 (13)**		RCT	12 (12M, 0F)	Healthy	26.8 (7.6)	27.1 (2.8)	120	Glucose	Agency	USA
Aspartame	20oz aspartame sweetened soda (Sunkist)							Insulin		
Sucralose	20oz splenda/sucralose sweetened soda (Diet Rite)									
Sucrose	20oz HFCS and sugar sweetened soda (Sunkist)									
Water	20oz carbonated water									

**Gonzalez, 2017 (14)**		RCT	10 (10M, 0F)	Healthy	22-26	20-24	120	Glucose	-	Spain
Ace-K+Asp+Cycl	330ml of Coke Zero with caffeine but without sugar							Insulin		
Ace-K+Asp+Cycl	330ml of caffeine-free Coke Zero without sugar									
Sucrose	330ml of Regular Coke with sugar and caffeine									
Sucrose	330ml of Regular Coke with sugar but without caffeine									
**Goza, 2018 (15)**		RCT	10 (5M, 5F)	Healthy	27.4 (3.5)	23.5 (2.6)	120	Glucose	Agency	Chile
Ace-K+Aspartame	350ml beverage with 84mg aspartame and 56mg Ace-K							Insulin		
Sucrose	350ml beverage with 38.7g sucrose									
**Eckstein 2021 (16)**		RCT	15 (10M, 5F)	Healthy	25.4 (2.5)	23.7 (1.7)	120	Glucose	-	Germany
Sucralose	300mL water with 0.2g sucralose							Insulin		
Glucose	300mL water with 1g/kg BM glucose									
Fructose	300mL water with 1g/kg BM fructose									
Sucrose	300mL water with 0.5g/kg BM glucose and fructose (each)									

Ace-K, acesulfame potassium; Asp, aspartame; Cycl, cyclamate; RCT, randomized controlled trial; NRS, non-randomized study; OGTT, oral glucose tolerance test; iAUC, incremental area under the curve; M, male; F, female; DM2, type 2 diabetes; N, normal; IGT, impaired glucose tolerance; HFCS, high-fructose corn syrup; BM, body mass

Table S4. *Characteristics of COUPLING INTERVENTIONS*

First author, year (reference)	Meal description	Study Design	Participants	Health status	Age, years	BMI, kg/m2	Duration (mins)	Outcomes extracted	Funding source	Country
**Wolf-Novak, 1988 (17)**		NRS	7 (2M, 5F)	Healthy	27.0 (3.0)	-	360	Glucose<br>Insulin	Agency	USA
Aspartame	12oz cherry-flavored beverage sweetened with 200mg aspartame and 60g CHO (partial hydrolysate of starch)									
Control	12oz cherry-flavored beverage with 60g CHO (partial hydrolysate of starch)									
**Melchoir, 1991 (18)**		RCT	10 (3M, 7F)	Healthy	21.7 (20-25)	20.6 (18.9-23.5)	180	Glucose<br>Insulin	Agency	France
Aspartame	400ml chocolate drink sweetened with 80mg aspartame									
Control	400ml chocolate drink sweetened with 50g sucrose									
**Solomi, 2019 (19)**		NRS	10 (4M, 6F)	Healthy	27.2 (6.9)	23.9 (2.4)	120	Glucose	Agency	UK
Ace-K+Aspartame	25g glucose in 125ml water consumed with 236ml diet cola sweetened with aspartame and ace-K (Caffeine-Free Diet Coke)									
Control	25g glucose in 125ml water consumed with 236ml water									

RCT, randomized controlled trial; NRS, non-randomized study; OGTT, oral glucose tolerance test; iAUC, incremental area under the curve; M, male; F, female; DM2, type 2 diabetes; N, normal; IGT, impaired glucose tolerance; HFCS, high-fructose corn syrup; CHO, carbohydrate; IDDM, insulin-dependent diabetes mellitus; NIDDM, non-insulin dependent diabetes mellitus

Table S5. *Characteristics of DELAYED COUPLING INTERVENTIONS*

First author, year (reference)	Meal description	Study Design	Participants	Health status	Age, years	BMI, kg/m2	Duration (mins)	Outcomes extracted	Funding source	Country
**Brown, 2012 (20)**		RCT	25 (13M, 12F)	Healthy	18.8 (4.4)	25.7 (4.6)	180	Glucose	Agency	USA
Diet Soda	240ml of diet soda (Diet Rite Cola containing sucralose and ace-K) consumed 10mins prior to 75g OGTT		9 (3M, 6F)<br>10 (1M, 9F)	DM1<br>DM2	18.2 (3.4)<br>17.9 (3.13)	21.7 (2.4)<br>35.0 (6.8)		Insulin<br>Glucagon		
Carbonated water	240ml of carbonated water (Zazz Seltzer) consumed 10mins prior to 75g OGTT							GLP-1<br>GIP<br>PYY		
**Pepino, 2013 (21)**		RCT	17 (2M, 15F)	Healthy	35.1 (4.1)	41.0 (6.2)	300	Glucose	Agency	USA
Sucralose	60ml solution containing 48mg sucralose was consumed 10mins before ingestion of 75g glucose solution							Insulin<br>GLP-1		
Water	60ml of distilled water was consumed 10mins prior to ingestion of 75g glucose solution							GIP		
**Temizkhan, 2014 (22)**		RCT	8 (4M, 4F)	Healthy	45.0 (4.1)	30.3 (4.5)	120	Glucose	-	Turkey
Aspartame	72mg aspartame in 200ml water was consumed 15mins prior to 75g OGTT		8 (4M, 4F)	DM2	51.5 (9.2)	33.7 (5.4)		Insulin<br>GLP-1		
Sucralose	24mg sucralose in 200ml water was consumed 15mins prior to 75g OGTT									
Water	200ml water was consumed 15mins prior to 75g OGTT									
**Sylvetsky, 2016 (23)**		RCT	30 (14M, 16F)	Healthy	29.7 (7.6)	25.8 (4.2)	120	Glucose	Agency	USA
Arm 1 Sucralose (68mg)	A drink containing 68mg sucralose mixed with 355ml water was consumed 10mins prior to 75g OGTT							Insulin<br>Glucagon		
Arm 1 Sucralose (170mg)	A drink containing 170mg sucralose mixed with 355ml water was consumed 10mins prior to 75g OGTT							GLP-1<br>GIP		
Arm 1 Sucralose (250mg)	A drink containing 250mg sucralose mixed with 355ml water was consumed 10mins prior to 75g OGTT							PYY		
Arm 1 Water	A drink containing 355ml water was consumed 10mins prior to 75g OGTT									
Arm 2 Diet Rite Cola	355ml caffeine-free Diet Rite Cola sweetened with 68mg sucralose and 41mg ace-K was consumed 10mins prior to 75g OGTT	RCT	31 (14M, 17F)	Healthy	27.4 (6.7)	26.3 (7.5)	120	Glucose<br>Insulin<br>GLP-1	Agency	USA
Arm 2 Diet Mountain Dew	355ml caffeine-free Diet Mountain Dew sweetened with 18mg sucralose, 18mg ace-K and 57mg aspartame was consumed 10mins prior to 75g OGTT							GIP<br>PYY		
Arm 2 NNS solution	A drink containing 68mg sucralose and 41mg ace-K in 355ml water was consumed 10mins prior to 75g OGTT									
Arm 2 Water	355ml water was consumed 10mins prior to 75g OGTT									
**Kariman Azari, 2017 (24)**		RCT	10 (3M, 7F)	Healthy	33.5 (11.1)	22.4 (2.5)	120	Glucose	Agency	USA
Saccharin	A drink containing 18mg saccharin dissolved in 60ml water was consumed 10mins prior to glucose solution (75g glucose in 600ml water)							Insulin<br>Glucagon<br>GLP-1		
Water	60ml distilled water was consumed 10mins prior to glucose solution (75g glucose in 600ml water)							GIP		
**Nichol, 2019 (25)**		RCT	10 (3M, 7F)	Lean	27.0 (4.2)	22.8 (0.9)	300	Glucose	Agency	USA
Sucralose	60ml solution containing 48mg sucralose was consumed 10mins prior to 75g OGTT		11 (1M, 10F)	Obese	29.5 (4.0)	37.7 (5.5)		Insulin<br>GIP		
Water	60ml distilled water was consumed 10mins prior to 75g OGTT									

**Solomi, 2020 (26)**		NRS	10 (5M, 5F)	Healthy	26.9 (3.3)	24.7 (1.1)	120	Glucose	-	USA
Diet cola	250ml diet soda (Caffeine-Free Diet Coke) sweetened with aspartame and ace-K was consumed 10mins prior to beverage containing 25g glucose									
Carbonated water	250ml carbonated water was consumed 10mins prior to beverage containing 25g glucose									

RCT, randomized controlled trial; NRS, non-randomized study; OGTT, oral glucose tolerance test; iAUC, incremental area under the curve; M, male; F, female; DM2, type 2 diabetes; N, normal; IGT, impaired glucose tolerance; HFCS, high-fructose corn syrup; CHO, carbohydrate; IDDM, insulin-dependent diabetes mellitus; NIDDM, non-insulin dependent diabetes mellitus

Table S6. *OVERALL CINeMA assessments of UNCOUPLING INTERVENTIONS examining the effect of non-nutritive sweetened beverages (NNS beverages) sweetened with individual or blends of non-nutritive sweeteners (NNS), water, and sugar-sweetened beverages (SSBs) sweetened with caloric sweeteners on postprandial blood GLUCOSE response in HEALTHY participants*

Comparison	Number of studies	Within-study bias	Reporting bias	Indirectness	Imprecision	Heterogeneity	Incoherence	Confidence rating	Reason(s) for downgrading
				DIRECT EVIDENCE					
Ace-K + Aspartame:Sucrose	1	No concerns	Low risk	No concerns	No concerns	No concerns	No concerns	High	
Ace-K + Aspartame + Cyclamate:Sucrose	2	No concerns	Low risk	No concerns	No concerns	Some concerns	No concerns	Moderate	Heterogeneity
AceK:Aspartame	1	Some concerns	Low risk	No concerns	No concerns	No concerns	No concerns	Moderate	Within-study bias
AceK:Cyclamate	1	Some concerns	Low risk	No concerns	No concerns	No concerns	No concerns	Moderate	Within-study bias
AceK:Saccharin	1	Some concerns	Low risk	No concerns	No concerns	No concerns	No concerns	Moderate	Within-study bias
AceK:Sucrose	1	Some concerns	Low risk	No concerns	No concerns	No concerns	No concerns	Moderate	Within-study bias
AceK:Water	1	Some concerns	Low risk	No concerns	No concerns	No concerns	No concerns	Moderate	Within-study bias
Aspartame:Cyclamate	1	Some concerns	Low risk	No concerns	No concerns	No concerns	No concerns	Moderate	Within-study bias
Aspartame:Glucose	4	No concerns	Low risk	No concerns	No concerns	No concerns	No concerns	High	
Aspartame:Saccharin	2	No concerns	Low risk	No concerns	No concerns	No concerns	No concerns	High	
Aspartame:Sucralose	1	No concerns	Low risk	No concerns	No concerns	No concerns	No concerns	High	
Aspartame:Sucrose	2	No concerns	Low risk	No concerns	No concerns	No concerns	No concerns	High	
Aspartame:Water	4	No concerns	Low risk	No concerns	No concerns	No concerns	No concerns	High	
Cyclamate:Saccharin	1	Some concerns	Low risk	No concerns	No concerns	No concerns	No concerns	Moderate	Within-study bias
Cyclamate:Sucrose	1	Some concerns	Low risk	No concerns	No concerns	No concerns	No concerns	Moderate	Within-study bias
Cyclamate:Water	1	Some concerns	Low risk	No concerns	No concerns	No concerns	No concerns	Moderate	Within-study bias
Fructose:Glucose	1	No concerns	Low risk	No concerns	No concerns	No concerns	No concerns	High	
Fructose:Sucralose	1	No concerns	Low risk	No concerns	No concerns	No concerns	No concerns	High	
Fructose:Sucrose	1	No concerns	Low risk	No concerns	No concerns	No concerns	No concerns	High	
Glucose:Sucralose	1	No concerns	Low risk	No concerns	No concerns	No concerns	No concerns	High	
Glucose:Sucrose	1	No concerns	Low risk	No concerns	No concerns	No concerns	No concerns	High	
Saccharin:Sucrose	1	Some concerns	Low risk	No concerns	No concerns	No concerns	No concerns	Moderate	Within-study bias
Saccharin:Water	2	Some concerns	Low risk	No concerns	No concerns	No concerns	No concerns	Moderate	Within-study bias
Stevia:Sucrose	1	Some concerns	Low risk	No concerns	No concerns	No concerns	No concerns	Moderate	Within-study bias
Sucralose:Sucrose	2	No concerns	Low risk	No concerns	No concerns	No concerns	No concerns	High	
Sucralose:Water	2	No concerns	Low risk	No concerns	No concerns	No concerns	No concerns	High	
Sucrose:Water	2	No concerns	Low risk	No concerns	No concerns	No concerns	No concerns	High	
				INDIRECT EVIDENCE					
Ace-K + Aspartame:Ace-K + Aspartame + Cyclamate	0	No concerns	Low risk	No concerns	No concerns	No concerns	No concerns	High	
AceK:Ace-K + Aspartame	0	No concerns	Low risk	No concerns	No concerns	No concerns	No concerns	High	
Ace-K + Aspartame:Aspartame	0	No concerns	Low risk	No concerns	No concerns	Some concerns	No concerns	Moderate	Heterogeneity
Ace-K + Aspartame:Cyclamate	0	No concerns	Low risk	No concerns	No concerns	No concerns	No concerns	High	
Ace-K + Aspartame:Fructose	0	No concerns	Low risk	No concerns	No concerns	No concerns	No concerns	High	
Ace-K + Aspartame:Glucose	0	No concerns	Low risk	No concerns	No concerns	No concerns	No concerns	High	
Ace-K + Aspartame:Saccharin	0	No concerns	Low risk	No concerns	No concerns	No concerns	No concerns	High	
Ace-K + Aspartame:Stevia	0	No concerns	Low risk	No concerns	No concerns	No concerns	No concerns	High	
Ace-K + Aspartame:Sucralose	0	No concerns	Low risk	No concerns	No concerns	Some concerns	No concerns	Moderate	Heterogeneity
Ace-K + Aspartame:Water	0	No concerns	Low risk	No concerns	No concerns	No concerns	No concerns	High	
AceK:Ace-K + Aspartame + Cyclamate	0	No concerns	Low risk	No concerns	Some concerns	No concerns	No concerns	Moderate	Imprecision
Ace-K + Aspartame + Cyclamate:Aspartame	0	No concerns	Low risk	No concerns	Some concerns	No concerns	No concerns	Moderate	Imprecision
Ace-K + Aspartame + Cyclamate:Cyclamate	0	No concerns	Low risk	No concerns	Some concerns	No concerns	No concerns	Moderate	Imprecision
Ace-K + Aspartame + Cyclamate:Fructose	0	No concerns	Low risk	No concerns	No concerns	No concerns	No concerns	High	
Ace-K + Aspartame + Cyclamate:Glucose	0	No concerns	Low risk	No concerns	No concerns	No concerns	No concerns	High	
Ace-K + Aspartame + Cyclamate:Saccharin	0	No concerns	Low risk	No concerns	No concerns	Some concerns	No concerns	Moderate	Heterogeneity
Ace-K + Aspartame + Cyclamate:Stevia	0	No concerns	Low risk	No concerns	No concerns	No concerns	No concerns	High	
Ace-K + Aspartame + Cyclamate:Sucralose	0	No concerns	Low risk	No concerns	Some concerns	No concerns	No concerns	Moderate	Imprecision
Ace-K + Aspartame + Cyclamate:Water	0	No concerns	Low risk	No concerns	No concerns	Some concerns	No concerns	Moderate	Heterogeneity
AceK:Fructose	0	No concerns	Low risk	No concerns	No concerns	No concerns	No concerns	High	
AceK:Glucose	0	No concerns	Low risk	No concerns	No concerns	No concerns	No concerns	High	
AceK:Stevia	0	Some concerns	Low risk	No concerns	Some concerns	No concerns	No concerns	Low	Within-study bias, Imprecision
AceK:Sucralose	0	Some concerns	Low risk	No concerns	No concerns	No concerns	No concerns	Moderate	Within-study bias
Aspartame:Fructose	0	No concerns	Low risk	No concerns	No concerns	No concerns	No concerns	High	
Aspartame:Stevia	0	Some concerns	Low risk	No concerns	No concerns	Some concerns	No concerns	Low	Within-study bias, Heterogeneity
Cyclamate:Fructose	0	No concerns	Low risk	No concerns	No concerns	No concerns	No concerns	High	
Cyclamate:Glucose	0	No concerns	Low risk	No concerns	No concerns	No concerns	No concerns	High	
Cyclamate:Stevia	0	Some concerns	Low risk	No concerns	Some concerns	No concerns	No concerns	Low	Within-study bias, Imprecision
Cyclamate:Sucralose	0	Some concerns	Low risk	No concerns	No concerns	No concerns	No concerns	Moderate	Within-study bias
Fructose:Saccharin	0	No concerns	Low risk	No concerns	No concerns	No concerns	No concerns	High	
Fructose:Stevia	0	No concerns	Low risk	No concerns	No concerns	No concerns	No concerns	High	
Fructose:Water	0	No concerns	Low risk	No concerns	No concerns	No concerns	No concerns	High	
Glucose:Saccharin	0	No concerns	Low risk	No concerns	No concerns	No concerns	No concerns	High	
Glucose:Stevia	0	No concerns	Low risk	No concerns	No concerns	No concerns	No concerns	High	
Glucose:Water	0	No concerns	Low risk	No concerns	No concerns	No concerns	No concerns	High	
Saccharin:Stevia	0	Some concerns	Low risk	No concerns	No concerns	Some concerns	No concerns	Low	Within-study bias, Heterogeneity
Saccharin:Sucralose	0	No concerns	Low risk	No concerns	No concerns	No concerns	No concerns	High	
Stevia:Sucralose	0	Some concerns	Low risk	No concerns	Some concerns	No concerns	No concerns	Low	Within-study bias, Imprecision
Stevia:Water	0	Some concerns	Low risk	No concerns	No concerns	Some concerns	No concerns	Low	Within-study bias, Heterogeneity

Table S7. *OVERALL CINeMA assessments of UNCOUPLING INTERVENTIONS examining the effect of non-nutritive sweetened beverages (NNS beverages) sweetened with individual or blends of non-nutritive sweeteners (NNS), water, and sugar-sweetened beverages (SSBs) sweetened with caloric sweeteners on postprandial blood GLUCOSE response in participants with TYPE 2 DIABETES*

Comparison	Number of studies	Within-study bias	Reporting bias	Indirectness	Imprecision	Heterogeneity	Incoherence	Confidence rating	Reason(s) for downgrading
**DIRECT EVIDENCE**									
**Aspartame:Glucose**	4	Some concerns	Low risk	No concerns	No concerns	No concerns	Major concerns	Low	Within-study bias, Incoherence
**Aspartame:Saccharin**	1	No concerns	Low risk	No concerns	Major concerns	No concerns	Major concerns	Low	Imprecision, Incoherence
**Aspartame:Water**	1	No concerns	Low risk	No concerns	Major concerns	No concerns	Major concerns	Low	Imprecision, Incoherence
**Saccharin:Water**	1	No concerns	Low risk	No concerns	Major concerns	No concerns	Major concerns	Low	Imprecision, Incoherence
**INDIRECT EVIDENCE**									
**Glucose:Saccharin**	0	No concerns	Low risk	No concerns	No concerns	Major concerns	Major concerns	Low	Heterogeneity, Incoherence
**Glucose:Water**	0	No concerns	Low risk	No concerns	No concerns	Major concerns	Major concerns	Low	Heterogeneity, Incoherence

Table S8. *OVERALL CINeMA assessments of UNCOUPLING INTERVENTIONS examining the effect of non-nutritive sweetened beverages (NNS beverages) sweetened with individual or blends of non-nutritive sweeteners (NNS), water, and sugar-sweetened beverages (SSBs) sweetened with caloric sweeteners on postprandial blood INSULIN response in HEALTHY participants*

Comparison	Number of studies	Within-study bias	Reporting bias	Indirectness	Imprecision	Heterogeneity	Incoherence	Confidence rating	Reason(s) for downgrading
				DIRECT EVIDENCE					
Ace-K + Aspartame:Sucrose	1	No concerns	Low risk	No concerns	No concerns	No concerns	No concerns	High	
Ace-K + Aspartame + Cyclamate:Sucrose	2	No concerns	Low risk	No concerns	No concerns	No concerns	No concerns	High	
AceK:Aspartame	1	Some concerns	Low risk	No concerns	No concerns	Major concerns	No concerns	Low	Within-study bias, Heterogeneity
AceK:Cyclamate	1	Some concerns	Low risk	No concerns	No concerns	Major concerns	No concerns	Low	Within-study bias, Heterogeneity
AceK:Saccharin	1	Some concerns	Low risk	No concerns	No concerns	Major concerns	No concerns	Low	Within-study bias, Heterogeneity
AceK:Sucrose	1	Some concerns	Low risk	No concerns	No concerns	No concerns	No concerns	Moderate	Within-study bias
AceK:Water	1	Some concerns	Low risk	No concerns	No concerns	Major concerns	No concerns	Low	Within-study bias, Heterogeneity
Aspartame:Cyclamate	1	Some concerns	Low risk	No concerns	No concerns	Major concerns	No concerns	Low	Within-study bias, Heterogeneity
Aspartame:Glucose	4	No concerns	Low risk	No concerns	No concerns	No concerns	No concerns	High	
Aspartame:Saccharin	2	No concerns	Low risk	No concerns	No concerns	Some concerns	No concerns	Moderate	Heterogeneity
Aspartame:Sucralose	1	No concerns	Low risk	No concerns	No concerns	No concerns	No concerns	High	
Aspartame:Sucrose	2	Some concerns	Low risk	No concerns	No concerns	No concerns	No concerns	Moderate	Within-study bias
Aspartame:Water	4	No concerns	Low risk	No concerns	No concerns	No concerns	No concerns	High	
Cyclamate:Saccharin	1	Some concerns	Low risk	No concerns	No concerns	Major concerns	No concerns	Low	Within-study bias, Heterogeneity
Cyclamate:Sucrose	1	Some concerns	Low risk	No concerns	No concerns	No concerns	No concerns	Moderate	Within-study bias
Cyclamate:Water	1	Some concerns	Low risk	No concerns	No concerns	Major concerns	No concerns	Low	Within-study bias, Heterogeneity
Fructose:Glucose	1	No concerns	Low risk	No concerns	No concerns	No concerns	No concerns	High	
Fructose:Sucralose	1	No concerns	Low risk	No concerns	No concerns	No concerns	No concerns	High	
Fructose:Sucrose	1	No concerns	Low risk	No concerns	Some concerns	No concerns	No concerns	Moderate	Imprecision
Glucose:Sucralose	1	No concerns	Low risk	No concerns	No concerns	No concerns	No concerns	High	
Glucose:Sucrose	1	No concerns	Low risk	No concerns	No concerns	No concerns	Some concerns	Moderate	Incoherence
Saccharin:Sucrose	1	Some concerns	Low risk	No concerns	No concerns	No concerns	No concerns	Moderate	Within-study bias
Saccharin:Water	2	No concerns	Low risk	No concerns	No concerns	Some concerns	No concerns	Moderate	Heterogeneity
Sucralose:Sucrose	2	No concerns	Low risk	No concerns	No concerns	No concerns	No concerns	High	
Sucralose:Water	2	No concerns	Low risk	No concerns	No concerns	No concerns	No concerns	High	
Sucrose:Water	2	Some concerns	Low risk	No concerns	No concerns	No concerns	No concerns	Moderate	Within-study bias
				INDIRECT EVIDENCE					
Ace-K + Aspartame:Ace-K + Aspartame + Cyclamate	0	No concerns	Low risk	No concerns	Some concerns	Some concerns	No concerns	Low	Imprecision, Heterogeneity
AceK:Ace-K + Aspartame	0	Some concerns	Low risk	No concerns	Some concerns	Some concerns	No concerns	Low	Within-study bias, Imprecision, Heterogeneity
Ace-K + Aspartame:Aspartame	0	No concerns	Low risk	No concerns	Some concerns	Some concerns	No concerns	Low	Imprecision, Heterogeneity
Ace-K + Aspartame:Cyclamate	0	Some concerns	Low risk	No concerns	Some concerns	Some concerns	No concerns	Low	Within-study bias, Imprecision, Heterogeneity
Ace-K + Aspartame:Fructose	0	No concerns	Low risk	No concerns	No concerns	No concerns	No concerns	High	
Ace-K + Aspartame:Glucose	0	No concerns	Low risk	No concerns	No concerns	No concerns	No concerns	High	
Ace-K + Aspartame:Saccharin	0	No concerns	Low risk	No concerns	Some concerns	Some concerns	No concerns	Low	Imprecision, Heterogeneity
Ace-K + Aspartame:Sucralose	0	No concerns	Low risk	No concerns	Some concerns	Some concerns	No concerns	Low	Imprecision, Heterogeneity
Ace-K + Aspartame:Water	0	No concerns	Low risk	No concerns	Some concerns	Some concerns	No concerns	Low	Imprecision, Heterogeneity
AceK:Ace-K + Aspartame + Cyclamate	0	Some concerns	Low risk	No concerns	Major concerns	No concerns	No concerns	Moderate	Within-study bias, Imprecision
Ace-K + Aspartame + Cyclamate:Aspartame	0	No concerns	Low risk	No concerns	Some concerns	Some concerns	No concerns	Low	Imprecision, Heterogeneity
Ace-K + Aspartame + Cyclamate:Cyclamate	0	Some concerns	Low risk	No concerns	Some concerns	Some concerns	No concerns	Low	Within-study bias, Imprecision, Heterogeneity
Ace-K + Aspartame + Cyclamate:Fructose	0	No concerns	Low risk	No concerns	No concerns	Some concerns	No concerns	Moderate	Heterogeneity
Ace-K + Aspartame + Cyclamate:Glucose	0	No concerns	Low risk	No concerns	No concerns	No concerns	No concerns	High	
Ace-K + Aspartame + Cyclamate:Saccharin	0	No concerns	Low risk	No concerns	Major concerns	No concerns	No concerns	Low	Imprecision
Ace-K + Aspartame + Cyclamate:Sucralose	0	No concerns	Low risk	No concerns	Some concerns	Some concerns	No concerns	Low	Imprecision, Heterogeneity
Ace-K + Aspartame + Cyclamate:Water	0	No concerns	Low risk	No concerns	Some concerns	Some concerns	No concerns	Low	Imprecision, Heterogeneity
AceK:Fructose	0	No concerns	Low risk	No concerns	No concerns	No concerns	No concerns	High	
AceK:Glucose	0	No concerns	Low risk	No concerns	No concerns	No concerns	No concerns	High	
AceK:Sucralose	0	Some concerns	Low risk	No concerns	No concerns	Major concerns	No concerns	Low	Within-study bias, Heterogeneity
Aspartame:Fructose	0	No concerns	Low risk	No concerns	No concerns	No concerns	No concerns	High	
Cyclamate:Fructose	0	No concerns	Low risk	No concerns	No concerns	Some concerns	No concerns	Moderate	Heterogeneity
Cyclamate:Glucose	0	No concerns	Low risk	No concerns	No concerns	No concerns	No concerns	High	
Cyclamate:Sucralose	0	Some concerns	Low risk	No concerns	No concerns	Major concerns	No concerns	Low	Within-study bias, Heterogeneity
Fructose:Saccharin	0	No concerns	Low risk	No concerns	No concerns	No concerns	No concerns	High	
Fructose:Water	0	No concerns	Low risk	No concerns	No concerns	No concerns	No concerns	High	
Glucose:Saccharin	0	No concerns	Low risk	No concerns	No concerns	No concerns	No concerns	High	
Glucose:Water	0	No concerns	Low risk	No concerns	No concerns	No concerns	No concerns	High	
Saccharin:Sucralose	0	No concerns	Low risk	No concerns	No concerns	Major concerns	No concerns	Low	Heterogeneity

Table S9. *OVERALL CINeMA assessments of UNCOUPLING INTERVENTIONS examining the effect of non-nutritive sweetened beverages (NNS beverages) sweetened with individual or blends of non-nutritive sweeteners (NNS), water, and sugar-sweetened beverages (SSBs) sweetened with caloric sweeteners on postprandial blood INSULIN response in participants* with *TYPE 2 DIABETES*

Comparison	Number of studies	Within-study bias	Reporting bias	Indirectness	Imprecision	Heterogeneity	Incoherence	Confidence rating	Reason(s) for downgrading
				DIRECT EVIDENCE					
**Aspartame:Glucose**	4	Some concerns	Low risk	No concerns	No concerns	Major concerns	Major concerns	Very low	["Within-study bias","Heterogeneity","Incoherence"]
**Aspartame:Saccharin**	1	No concerns	Low risk	No concerns	Major concerns	No concerns	Major concerns	Low	["Imprecision","Incoherence"]
**Aspartame:Water**	1	No concerns	Low risk	No concerns	Major concerns	No concerns	Major concerns	Low	["Imprecision","Incoherence"]
**Saccharin:Water**	1	No concerns	Low risk	No concerns	Major concerns	No concerns	Major concerns	Low	["Imprecision","Incoherence"]
				INDIRECT EVIDENCE					
**Glucose:Saccharin**	0	No concerns	Low risk	No concerns	Some concerns	Some concerns	Major concerns	Very low	["Imprecision","Heterogeneity","Incoherence"]
**Glucose:Water**	0	No concerns	Low risk	No concerns	Some concerns	Some concerns	Major concerns	Very low	["Imprecision","Heterogeneity","Incoherence"]

Table S10. *GRADE assessments of UNCOUPLING INTERVENTIONS examining the effect of non-nutritive sweetened beverages (NNS beverages) sweetened with individual or blends of non-nutritive sweeteners (NNS), water, and sugar-sweetened beverages (SSBs) sweetened with caloric sweeteners on postprandial blood GLP-1 response in HEALTHY participants*

**Treatments**	**Effect Estimate**	**GRADE Assessments**						
		**1**	**2**	**3**	**4**	**5**	**6**	**Overall**
Aspartame vs. Sucrose	262.15 (-12.991, 537.291)	-	-	↓	↓	-	-	LOW ⊕⊕
Aspartame vs. Water	-86.97 (-345.469, 171.529)	-	-	↓	↓	-	-	LOW ⊕⊕
Sucralose vs. Water	0.00 (-3251.947, 3251.947)	-	-	↓	↓	-	-	LOW ⊕⊕
Water vs. Sucrose	**349.12 (63.721, 634.519)**	-	-	↓	↓	-	-	LOW ⊕⊕
Aspartame vs. Sucralose	-86.97 (-3349.174, 3175.234)	-	-	↓	↓	-	-	LOW ⊕⊕
Sucralose vs. Sucrose	349.12 (-2915.326, 3613.566)	-	-	↓	↓	-	-	LOW ⊕⊕

Data reported as mean difference with 95% confidence intervals (CIs). Due to lack of available trial data, the CINeMA (Confidence in Network Meta-Analysis) web application was unable to make judgements for the six domains. Thus, manual judgements were made based on the CiNEMA framework (27, 28). GRADE domains refer to: (1), within-study bias; (2), reporting bias; (3), indirectness; (4), imprecision; (5) heterogeneity; and (6) incoherence. The risk of bias domain was assessed using the Cochrane RoB2 tool (no downgrade if there was no concern for overall risk of bias; downgraded if there was some or major concern for overall risk of bias). Evidence was downgraded for imprecision if 95% CIs overlapped the minimally important difference of 82 pmol*min/L. Evidence was downgraded for indirectness due lack of available trial data. There is no general consensus on a clinically important size difference for 2-hour blood GLP-1 incremental area under the curve (iAUC). Health Canada considers a 20% reduction in glucose iAUC to be the minimum physiologically relevant difference as per “Guideline Concerning the Safety and Physiological Effects of Novel Fibre Sources and Food Products Containing Them” (29) [xx]. Extrapolating the 20% threshold in glucose iAUC, we calculated the MID of GLP-1 iAUC by taking 20% of 412 pmol*mins/L which is the median of the difference in GLP-1 iAUC among healthy participants (~95 pmol*mins/L) and those with type 2 diabetes (~633 pmol*mins/L) after a glucose or meal tolerance test (30). Significant results are bolded in black. Non-significant results are grey and not bolded. Trivial (significant) effects (<1 MID) or no effects have a white background; small important effects (>1 MID) have a light blue background; moderate effects (>2 MID) have a darker blue background; large effects (>5 to <10 MID) have a purple background; very large effects (>10 MID) have a black background. Confidence in the effect estimate is shown for each treatment comparison: high confidence ⊕⊕⊕⊕; moderate confidence ⊕⊕⊕; low confidence ⊕⊕; very low confidence ⊕

Table S11. *GRADE assessments of UNCOUPLING INTERVENTIONS examining the effect of non-nutritive sweetened beverages (NNS beverages) sweetened with individual or blends of non-nutritive sweeteners (NNS), water, and sugar-sweetened beverages (SSBs) sweetened with caloric sweeteners on postprandial blood GIP response in HEALTHY participants*

**Treatments**	**Effect Estimate**	**GRADE Assessments**						
		**1**	**2**	**3**	**4**	**5**	**6**	**Overall**
Aspartame vs. Sucrose	**1007.80 (675.588, 1340.012)**	-	-	↓	-	-	-	MODERATE ⊕⊕
Aspartame vs. Water	23.21 (-238.591, 285.011)	-		↓	↓	-	-	LOW ⊕⊕
Water vs. Sucrose	**984.59 (649.367, 1319.813)**	-	-	↓	-	-	-	MODERATE ⊕⊕

Data reported as mean difference with 95% confidence intervals (CIs). Due to lack of available trial data, the CINeMA (Confidence in Network Meta-Analysis) web application was unable to make judgements for the six domains. Thus, manual judgements were made based on the CiNEMA framework (27, 28). GRADE domains refer to: (1), within-study bias; (2), reporting bias; (3), indirectness; (4), imprecision; (5) heterogeneity; and (6) incoherence. The risk of bias domain was assessed using the Cochrane RoB2 tool (no downgrade if there was no concern for overall risk of bias; downgraded if there was some or major concern for overall risk of bias). Evidence was downgraded for imprecision if 95% CIs overlapped the minimally important difference of 94 pmol*min/L. Evidence was downgraded for indirectness due lack of available trial data. There is no general consensus on a clinically important size difference for 2-hour blood GIP incremental area under the curve (iAUC). Health Canada considers a 20% reduction in glucose iAUC to be the minimum physiologically relevant difference as per "Guideline Concerning the Safety and Physiological Effects of Novel Fibre Sources and Food Products Containing Them" (29). Extrapolating the 20% threshold in glucose iAUC, we calculated the MID of GIP iAUC by taking 20% of 468 pmol*mins/L which is the median of the difference in GIP iAUC among healthy participants (~168 pmol*mins/L) and those with type 2 diabetes (~768 pmol*mins/L) after a glucose or meal tolerance test (31). Significant results are bolded in white. Non-significant results are grey and not bolded. Trivial (significant) effects (<1 MID) or no effects have a white background; small important effects (>1 MID) have a light blue background; moderate effects (>2 MID) have a darker blue background; large effects (>5 to <10 MID) have a purple background; very large effects (>10 MID) have a black background. Confidence in the effect estimate is shown for each treatment comparison: high confidence ⊕⊕⊕⊕; moderate confidence ⊕⊕⊕; low confidence ⊕⊕; very low confidence ⊕

Table S12. *GRADE assessments of UNCOUPLING INTERVENTIONS examining the effect of non-nutritive sweetened beverages (NNS beverages) sweetened with individual or blends of non-nutritive sweeteners (NNS), water, and sugar-sweetened beverages (SSBs) sweetened with caloric sweeteners on postprandial blood GHRELIN response in HEALTHY participants*

**Treatments**	**Effect Estimate**	**GRADE Assessments**						
		**1**	**2**	**3**	**4**	**5**	**6**	**Overall**
Aspartame vs. Sucrose	-29.56 (-357.818, 298.698)	-	-	↓	↓	-	-	LOW ⊕⊕
Aspartame vs. Water	19.40 (-330.006, 368.806)	-	-	↓	↓	-	-	LOW ⊕⊕
Water vs. Sucrose	-48.96 (-425.914, 327.994)	-	-	↓	↓	-	-	LOW ⊕⊕

Data reported as mean difference with 95% confidence intervals (CIs). Due to lack of available trial data, the CINeMA (Confidence in Network Meta-Analysis) web application was unable to make judgements for the six domains. Thus, manual judgements were made based on the CiNEMA framework (27, 28) (reference). GRADE domains refer to: (1), within-study bias; (2), reporting bias; (3), indirectness; (4), imprecision; (5) heterogeneity; and (6) incoherence. The risk of bias domain was assessed using the Cochrane RoB2 tool (no downgrade if there was no concern for overall risk of bias; downgraded if there was some or major concern for overall risk of bias). Evidence was downgraded for imprecision if 95% CIs overlapped the minimally important difference of 9.8 pmol*min/L. Evidence was downgraded for indirectness due lack of available trial data. There is no general consensus on a clinically important size difference for 2-hour blood ghrelin incremental area under the curve (iAUC). Health Canada considers a 20% reduction in glucose iAUC to be the minimum physiologically relevant difference as per "Guideline Concerning the Safety and Physiological Effects of Novel Fibre Sources and Food Products Containing Them" (29). Extrapolating the 20% threshold in glucose iAUC, we calculated the MID of ghrelin iAUC by taking 20% of 20% of -49.0 pmol*min/L (sucrose vs. water comparison) which was -9.8 pmol*mins/L. Significant results are bolded in black. Non-significant results are grey and not bolded. Trivial (significant) effects (<1 MID) or no effects have a white background; small important effects (>1 MID) have a light blue background; moderate effects (>2 MID) have a darker blue background; large effects (>5 to <10 MID) have a purple background; very large effects (>10 MID) have a black background. Confidence in the effect estimate is shown for each treatment comparison: high confidence ⊕⊕⊕⊕; moderate confidence ⊕⊕⊕; low confidence ⊕⊕; very low confidence ⊕

Table S13. *GRADE assessments of UNCOUPLING INTERVENTIONS examining the effect of non-nutritive sweetened beverages (NNS beverages) sweetened with individual or blends of non-nutritive sweeteners (NNS), water, and sugar-sweetened beverages (SSBs) sweetened with caloric sweeteners on postprandial blood GLUCAGON response in HEALTHY participants*

**Treatments**	**Effect Estimate**	**GRADE Assessments**						
		**1**	**2**	**3**	**4**	**5**	**6**	**Overall**
Aspartame vs. Saccharin	-1203.101 (-7381.296, 4975.093)	-	-	↓	↓	-	-	LOW ⊕⊕
Aspartame vs. Water	914.930 (-4370.027, 6199.888)	-	-	↓	↓	-	-	LOW ⊕⊕
Saccharin vs. Water	2118.032 (-3598.818, 7834.882)	-	-	↓	↓	-	-	LOW ⊕⊕
Aspartame vs. Glucose	59.273 (-1170.323, 1288.868)	-	-	↓	↓	-	-	LOW ⊕⊕
Saccharin vs. Glucose	1262.374 (-5036.991, 7561.739)	-	-	↓	↓	-	-	LOW ⊕⊕
Water vs. Glucose	-855.658 (-6281.769, 4570.453)	-	-	↓	↓	-	-	LOW ⊕⊕

Data reported as mean difference with 95% confidence intervals (CIs). Due to lack of available trial data, the CINeMA (Confidence in Network Meta-Analysis) web application was unable to make judgements for the six domains. Thus, manual judgements were made based on the CiNEMA framework (27, 28). GRADE domains refer to: (1), within-study bias; (2), reporting bias; (3), indirectness; (4), imprecision; (5) heterogeneity; and (6) incoherence. The risk of bias domain was assessed using the Cochrane RoB2 tool (no downgrade if there was no concern for overall risk of bias; downgraded if there was some or major concern for overall risk of bias). Evidence was downgraded for imprecision if 95% CIs overlapped the minimally important difference of 205 pmol*min/L. Evidence was downgraded for indirectness due lack of available trial data. There is no general consensus on a clinically important size difference for 2-hour blood glucagon incremental area under the curve (iAUC). Health Canada considers a 20% reduction in glucose iAUC to be the minimum physiologically relevant difference as per "Guideline Concerning the Safety and Physiological Effects of Novel Fibre Sources and Food Products Containing Them" (29). Extrapolating the 20% threshold in glucose iAUC, we calculated the MID of glucagon iAUC by taking 20% of 1025 pmol*mins/L which is the median value of glucagon iAUC (water vs. glucose comparisons) in healthy individuals (~850 pmol*mins/L) and those with type 2 diabetes (~1200 pmol*mins/L). Significant results are bolded in black. Non-significant results are grey and not bolded. Trivial (significant) effects (<1 MID) or no effects have a white background; small important effects (>1 MID) have a light blue background; moderate effects (>2 MID) have a darker blue background; large effects (>5 to <10 MID) have a purple background; very large effects (>10 MID) have a black background. Confidence in the effect estimate is shown for each treatment comparison: high confidence ⊕⊕⊕⊕; moderate confidence ⊕⊕⊕; low confidence ⊕⊕; very low confidence ⊕

Table S14. *GRADE assessments of UNCOUPLING INTERVENTIONS examining the effect of non-nutritive sweetened beverages (NNS beverages) sweetened with individual or blends of non-nutritive sweeteners (NNS), water, and sugar-sweetened beverages (SSBs) sweetened with caloric sweeteners on postprandial blood GLUCAGON response in participants with TYPE 2 DIABETES*

**Treatments**	**Effect Estimate**	**GRADE Assessments**						
		**1**	**2**	**3**	**4**	**5**	**6**	**Overall**
Aspartame vs. Saccharin	-140.368 (-4533.755, 4253.019)	-	-	↓	↓	-	-	LOW ⊕⊕
Aspartame vs. Water	1003.800 (-4010.785, 6018.385)	-	-	↓	↓	-	-	LOW ⊕⊕
Saccharin vs. Water	1144.168 (-3847.925, 6136.261)	-	-	↓	↓	-	-	LOW ⊕⊕
Aspartame vs. Glucose	-198.894 (-975.641, 577.854)	-	-	↓	↓	-	-	LOW ⊕⊕
Saccharin vs. Glucose	-58.526 (-4520.048, 4402.997)	-	-	↓	↓	-	-	LOW ⊕⊕
Water vs. Glucose	-1202.694 (-6277.080, 3871.693)	-	-	↓	↓	-	-	LOW ⊕⊕

Data reported as mean difference with 95% confidence intervals (CIs). Due to lack of available trial data, the CINeMA (Confidence in Network Meta-Analysis) web application was unable to make judgements for the six domains. Thus, manual judgements were made based on the CiNEMA framework (27, 28) (reference). GRADE domains refer to: (1), within-study bias; (2), reporting bias; (3), indirectness; (4), imprecision; (5) heterogeneity; and (6) incoherence. The risk of bias domain was assessed using the Cochrane RoB2 tool (no downgrade if there was no concern for overall risk of bias; downgraded if there was some or major concern for overall risk of bias). Evidence was downgraded for imprecision if 95% CIs overlapped the minimally important difference of 205 pmol*min/L. Evidence was downgraded for indirectness due lack of available trial data. There is no general consensus on a clinically important size difference for 2-hour blood glucagon incremental area under the curve (iAUC). Health Canada considers a 20% reduction in glucose iAUC to be the minimum physiologically relevant difference as per "Guideline Concerning the Safety and Physiological Effects of Novel Fibre Sources and Food Products Containing Them" (29). Extrapolating the 20% threshold in glucose iAUC, we calculated the MID of glucagon iAUC by taking 20% of 1025 pmol*mins/L which is the median value of glucagon iAUC (water vs. glucose comparisons) in healthy individuals (~850 pmol*mins/L) and those with type 2 diabetes (~1200 pmol*mins/L). Significant results are bolded in black. Non-significant results are grey and not bolded. Trivial (significant) effects (<1 MID) or no effects have a white background; small important effects (>1 MID) have a light blue background; moderate effects (>2 MID) have a darker blue background; large effects (>5 to <10 MID) have a purple background; very large effects (>10 MID) have a black background. Confidence in the effect estimate is shown for each treatment comparison: high confidence ⊕⊕⊕⊕; moderate confidence ⊕⊕⊕; low confidence ⊕⊕; very low confidence ⊕

Table S15. *GRADE assessments of COUPLING INTERVENTIONS examining the effect of non-nutritive sweetened beverages (NNS beverages) sweetened with individual or blends of non-nutritive sweeteners (NNS) and controls on postprandial blood GLUCOSE response in HEALTHY participants*

**Treatments**	**Effect Estimate**	**GRADE Assessments**						
		**1**	**2**	**3**	**4**	**5**	**6**	**Overall**
Aspartame vs. Aspartame+Ace-K	31.639<br>(-33.462, 96.739)	↓	-	↓	↓	-	-	LOW<br>⊕⊕
Aspartame vs. Control	32.538<br>(-24.927, 90.004)	-	-	↓	↓	-	-	LOW<br>⊕⊕
Aspartame+Ace-K vs. Control	0.900<br>(-29.689, 31.489)	↓	-	↓	↓	-	-	LOW<br>⊕⊕

Data reported as mean difference with 95% confidence intervals (CIs). Due to lack of available trial data, the CINeMA (Confidence in Network Meta-Analysis) web application was unable to make judgements for the six domains. Thus, manual judgements were made based on the CiNEMA framework (27, 28). GRADE domains refer to: (1), within-study bias; (2), reporting bias; (3), indirectness; (4), imprecision; (5) heterogeneity; and (6) incoherence. The risk of bias domain was assessed using the Cochrane RoB2 tool (no downgrade if there was no concern for overall risk of bias; downgraded if there was some or major concern for overall risk of bias). Evidence was downgraded for imprecision if 95% CIs overlapped the minimally important difference of 100 mmol*min/L. Evidence was downgraded for indirectness due lack of available trial data. There is no general consensus on a clinically important size difference for 2-hour blood glucose incremental area under the curve (iAUC). Health Canada considers a 20% reduction in glucose iAUC to be the minimum physiologically relevant difference as per “Guideline Concerning the Safety and Physiological Effects of Novel Fibre Sources and Food Products Containing Them” (29). Thus, we calculated the MID of glucose iAUC by taking 20% of 500 mmol*mins/L which is the median of glucose iAUC in healthy participants (~300 mmol*mins/L) and those with type 2 diabetes (~700 mmol*mins/L) after a 75-g oral glucose tolerance test (32-34). Significant results are bolded in black. Non-significant results are grey and not bolded. Trivial (significant) effects (<1 MID) or no effects have a white background; small important effects (>1 MID) have a light blue background; moderate effects (>2 MID) have a darker blue background; large effects (>5 to <10 MID) have a purple background; very large effects (>10 MID) have a black background. Confidence in the effect estimate is shown for each treatment comparison: high confidence ⊕⊕⊕⊕; moderate confidence ⊕⊕⊕; low confidence ⊕⊕; very low confidence ⊕

Table S16. *GRADE assessments of COUPLING INTERVENTIONS examining the effect of non-nutritive sweetened beverages (NNS beverages) sweetened with individual or blends of non-nutritive sweeteners (NNS) and controls on postprandial blood INSULIN response in HEALTHY participants*

**Treatments**	**Effect Estimate**	**GRADE Assessments**						
		**1**	**2**	**3**	**4**	**5**	**6**	**Overall**
Aspartame vs. Unsweetened Control	1718.16 (-2926.557, 6362.877)	↓	-	↓	↓	-	-	LOW ⊕⊕
Aspartame vs. Sucrose	33234.84 (-9197.392, 75667.071)	-	-	↓	↓	-	-	LOW ⊕⊕
Unsweetened Control vs. Sucrose	31516.68 (-11169.005, 74202.364)	↓	-	↓	↓	-	-	LOW ⊕⊕

Data reported as mean difference with 95% confidence intervals (CIs). Due to lack of available trial data, the CINeMA (Confidence in Network Meta-Analysis) web application was unable to make judgements for the six domains. Thus, manual judgements were made based on the CiNEMA framework (27, 28). GRADE domains refer to: (1), within-study bias; (2), reporting bias; (3), indirectness; (4), imprecision; (5) heterogeneity; and (6) incoherence. The risk of bias domain was assessed using the Cochrane RoB2 tool (no downgrade if there was no concern for overall risk of bias; downgraded if there was some or major concern for overall risk of bias). Evidence was downgraded for imprecision if 95% CIs overlapped the minimally important difference of 2250 pmol*min/L. Evidence was downgraded for indirectness due lack of available trial data. There is no general consensus on a clinically important size difference for 2-hour blood insulin incremental area under the curve (iAUC). Health Canada considers a 20% reduction in glucose iAUC to be the minimum physiologically relevant difference as per "Guideline Concerning the Safety and Physiological Effects of Novel Fibre Sources and Food Products Containing Them" (29). Extrapolating the 20% threshold in glucose iAUC, we calculated the MID of insulin iAUC by taking 20% of 11250 pmol*mins/L which is the median of insulin iAUC in participants with type 2 diabetes (~6200 pmol*mins/L) and healthy participants (~16300 pmol*mins/L) after a 75-g oral glucose tolerance test (32-34). Non-significant results are grey and not bolded. Trivial (significant) effects (<1 MID) or no effects have a white background; small important effects (>1 MID) have a light blue background; moderate effects (>2 MID) have a darker blue background; large effects (>5 to <10 MID) have a purple background; very large effects (>10 MID) have a black background. Confidence in the effect estimate is shown for each treatment comparison: high confidence ⊕⊕⊕⊕; moderate confidence ⊕⊕⊕; low confidence ⊕⊕; very low confidence ⊕

Table S17. *OVERALL CINeMA assessments of DELAYED COUPLING INTERVENTIONS examining the effect of non-nutritive sweetened beverages (NNS beverages) sweetened with individual or blends of non-nutritive sweeteners (NNS) and water on postprandial blood GLUCOSE response in HEALTHY participants*

Comparison	Number of studies	Within-study bias	Reporting bias	Indirectness	Imprecision	Heterogeneity	Incoherence	Confidence rating	Reason(s) for downgrading
DIRECT EVIDENCE									
Ace-K + Aspartame:Water	1	Some concerns	Low risk	No concerns	No concerns	No concerns	No concerns	Moderate	Within-study bias
Ace-K + Aspartame + Sucralose:Ace-K + Sucralose	1	No concerns	Low risk	No concerns	No concerns	Some concerns	No concerns	Moderate	Heterogeneity
Ace-K + Aspartame + Sucralose:Water	1	No concerns	Low risk	No concerns	No concerns	Some concerns	No concerns	Moderate	Heterogeneity
Ace-K + Sucralose:Water	2	No concerns	Low risk	No concerns	No concerns	No concerns	No concerns	High	
Aspartame:Sucralose	1	No concerns	Low risk	No concerns	Some concerns	Some concerns	No concerns	Low	Imprecision, Heterogeneity
Aspartame:Water	1	No concerns	Low risk	No concerns	Some concerns	Some concerns	No concerns	Low	Imprecision, Heterogeneity
Saccharin:Water	1	No concerns	Low risk	No concerns	Major concerns	No concerns	No concerns	Moderate	Imprecision
Sucralose:Water	4	No concerns	Low risk	No concerns	No concerns	No concerns	No concerns	High	
INDIRECT EVIDENCE									
Ace-K + Aspartame:Ace-K + Aspartame + Sucralose	0	No concerns	Low risk	No concerns	No concerns	Major concerns	No concerns	Moderate	Heterogeneity
Ace-K + Aspartame:Ace-K + Sucralose	0	No concerns	Low risk	No concerns	No concerns	Some concerns	No concerns	Moderate	Heterogeneity
Ace-K + Aspartame:Aspartame	0	No concerns	Low risk	No concerns	Some concerns	Some concerns	No concerns	Low	Imprecision, Heterogeneity
Ace-K + Aspartame:Saccharin	0	No concerns	Low risk	No concerns	Major concerns	No concerns	No concerns	Moderate	Imprecision
Ace-K + Aspartame:Sucralose	0	No concerns	Low risk	No concerns	No concerns	Some concerns	No concerns	Moderate	Heterogeneity
Ace-K + Aspartame + Sucralose:Aspartame	0	No concerns	Low risk	No concerns	Some concerns	Some concerns	No concerns	Low	Imprecision, Heterogeneity
Ace-K + Aspartame + Sucralose:Saccharin	0	No concerns	Low risk	No concerns	Major concerns	No concerns	No concerns	Moderate	Imprecision
Ace-K + Aspartame + Sucralose:Sucralose	0	No concerns	Low risk	No concerns	Some concerns	No concerns	No concerns	Moderate	Imprecision
Ace-K + Sucralose:Aspartame	0	No concerns	Low risk	No concerns	Some concerns	Some concerns	No concerns	Low	Imprecision, Heterogeneity
Ace-K + Sucralose:Saccharin	0	No concerns	Low risk	No concerns	Major concerns	No concerns	No concerns	Moderate	Imprecision
Ace-K + Sucralose:Sucralose	0	No concerns	Low risk	No concerns	No concerns	No concerns	No concerns	High	
Aspartame:Saccharin	0	No concerns	Low risk	No concerns	Some concerns	Some concerns	No concerns	Low	Imprecision, Heterogeneity
Saccharin:Sucralose	0	No concerns	Low risk	No concerns	Some concerns	Some concerns	No concerns	Low	Imprecision, Heterogeneity

Table S18. *GRADE assessments of DELAYED COUPLING INTERVENTIONS examining the effect of non-nutritive sweetened beverages (NNS beverages) sweetened with individual or blends of non-nutritive sweeteners (NNS) and water on postprandial blood GLUCOSE response in participants with TYPE 2 DIABETES*

**Treatments**	**Effect Estimate**	**GRADE Assessments**						
		**1**	**2**	**3**	**4**	**5**	**6**	**Overall**
Aspartame vs. Sucralose	-33.230 (-263.439, 196.979)	-	-	↓	↓	-	-	LOW ⊕⊕
Aspartame vs. Sucralose+Ace-K	43.516 (-264.436, 351.467)	-	-	↓	↓	-	-	LOW ⊕⊕
Aspartame vs. Water	-9.950 (-269.812, 249.912)	-	-	↓	↓	-	-	LOW ⊕⊕
Sucralose vs. Sucralose+Ace-K	76.746 (-206.631, 360.122)	-	-	↓	↓	-	-	LOW ⊕⊕
Sucralose vs. Water	23.280 (-206.929, 253.489)	-	-	↓	↓	-	-	LOW ⊕⊕
Sucralose+Ace-K vs. Water	-53.466 (-218.711, 111.780)	-	-	↓	↓	-	-	LOW ⊕⊕

Data reported as mean difference with 95% confidence intervals (CIs). Due to lack of available trial data, the CINeMA (Confidence in Network Meta-Analysis) web application was unable to make judgements for the six domains. Thus, manual judgements were made based on the CiNEMA framework (27, 28). GRADE domains refer to: (1), within-study bias; (2), reporting bias; (3), indirectness; (4), imprecision; (5) heterogeneity; and (6) incoherence. The risk of bias domain was assessed using the Cochrane RoB2 tool (no downgrade if there was no concern for overall risk of bias; downgraded if there was some or major concern for overall risk of bias). Evidence was downgraded for imprecision if 95% CIs overlapped the minimally important difference of 100 mmol*min/L. Evidence was downgraded for indirectness due lack of available trial data. There is no general consensus on a clinically important size difference for 2-hour blood glucose incremental area under the curve (iAUC). Health Canada considers a 20% reduction in glucose iAUC to be the minimum physiologically relevant difference as per "Guideline Concerning the Safety and Physiological Effects of Novel Fibre Sources and Food Products Containing Them" (29). Thus, we calculated the MID of glucose iAUC by taking 20% of 500 mmol*mins/L which is the median of glucose iAUC in healthy participants (~300 mmol*mins/L) and those with type 2 diabetes (~700 mmol*mins/L). Significant results are bolded in black. Non-significant results are grey and not bolded. Trivial (significant) effects (<1 MID) or no effects have a white background; small important effects (>1 MID) have a light blue background; moderate effects (>2 MID) have a darker blue background; large effects (>5 to <10 MID) have a purple background; very large effects (>10 MID) have a black background. Confidence in the effect estimate is shown for each treatment comparison: high confidence ⊕⊕⊕⊕; moderate confidence ⊕⊕⊕; low confidence ⊕⊕; very low confidence ⊕

Table S19. *OVERALL CINeMA assessments of DELAYED COUPLING INTERVENTIONS examining the effect of non-nutritive sweetened beverages (NNS beverages) sweetened with individual or blends of non-nutritive sweeteners (NNS) and water on postprandial blood INSULIN response in HEALTHY participants*

Comparison	Number of studies	Within-study bias	Reporting bias	Indirectness	Imprecision	Heterogeneity	Incoherence	Confidence rating	Reason(s) for downgrading
DIRECT EVIDENCE									
**Ace-K + Aspartame + Sucralose:Ace-K + Sucralose**	1	No concerns	Low risk	No concerns	Major concerns	No concerns	No concerns	Moderate	Imprecision
**Ace-K + Aspartame + Sucralose:Water**	1	No concerns	Low risk	No concerns	Major concerns	No concerns	No concerns	Moderate	Imprecision
**Ace-K + Sucralose:Water**	2	No concerns	Low risk	No concerns	Major concerns	No concerns	No concerns	Moderate	Imprecision
**Aspartame:Sucralose**	1	No concerns	Low risk	No concerns	Major concerns	No concerns	No concerns	Moderate	Imprecision
**Aspartame:Water**	1	No concerns	Low risk	No concerns	Major concerns	No concerns	No concerns	Moderate	Imprecision
**Saccharin:Water**	1	No concerns	Low risk	No concerns	Major concerns	No concerns	No concerns	Moderate	Imprecision
**Sucralose:Water**	4	No concerns	Low risk	No concerns	Major concerns	No concerns	No concerns	Moderate	Imprecision
INDIRECT EVIDENCE									
**Ace-K + Aspartame + Sucralose:Aspartame**	0	No concerns	Low risk	No concerns	Major concerns	No concerns	No concerns	Moderate	Imprecision
**Ace-K + Aspartame + Sucralose:Saccharin**	0	No concerns	Low risk	No concerns	Major concerns	No concerns	No concerns	Moderate	Imprecision
**Ace-K + Aspartame + Sucralose:Sucralose**	0	No concerns	Low risk	No concerns	Major concerns	No concerns	No concerns	Moderate	Imprecision
**Ace-K + Sucralose:Aspartame**	0	No concerns	Low risk	No concerns	Major concerns	No concerns	No concerns	Moderate	Imprecision
**Ace-K + Sucralose:Saccharin**	0	No concerns	Low risk	No concerns	Major concerns	No concerns	No concerns	Moderate	Imprecision
**Ace-K + Sucralose:Sucralose**	0	No concerns	Low risk	No concerns	Major concerns	No concerns	No concerns	Moderate	Imprecision
**Aspartame:Saccharin**	0	No concerns	Low risk	No concerns	Major concerns	No concerns	No concerns	Moderate	Imprecision
**Saccharin:Sucralose**	0	No concerns	Low risk	No concerns	Major concerns	No concerns	No concerns	Moderate	Imprecision

Table S20. *GRADE assessments of DELAYED COUPLING INTERVENTIONS examining the effect of non-nutritive sweetened beverages (NNS beverages) sweetened with individual or blends of non-nutritive sweeteners (NNS) and water on postprandial blood INSULIN response in participants with TYPE 2 DIABETES*

**Treatments**	**Effect Estimate**	**GRADE Assessments**						
		**1**	**2**	**3**	**4**	**5**	**6**	**Overall**
Aspartame vs. Sucralose	-6897.480 (-26800.86, 13005.90)	-	-	↓	↓	-	-	LOW ⊕⊕
Aspartame vs. Sucralose+Ace-K	-1254.645 (-33264.83, 30755.54)	-	-	↓	↓	-	-	LOW ⊕⊕
Aspartame vs. Water	3700.840 (-18897.51, 26299.19)	-	-	↓	↓	-	-	LOW ⊕⊕
Sucralose vs. Sucralose+Ace-K	5642.835 (-25142.04, 36427.71)	-	-	↓	↓	-	-	LOW ⊕⊕
Sucralose vs. Water	10598.320 (-10228.15, 31424.79)	-	-	↓	↓	-	-	LOW ⊕⊕
Sucralose+Ace-K vs. Water	4955.485 (-17715.34, 27626.31)	-	-	↓	↓	-	-	LOW ⊕⊕

Data reported as mean difference with 95% confidence intervals (CIs). Due to lack of available trial data, the CINeMA (Confidence in Network Meta-Analysis) web application was unable to make judgements for the six domains. Thus, manual judgements were made based on the CiNEMA framework (27, 28). GRADE domains refer to: (1), within-study bias; (2), reporting bias; (3), indirectness; (4), imprecision; (5) heterogeneity; and (6) incoherence. The risk of bias domain was assessed using the Cochrane RoB2 tool (no downgrade if there was no concern for overall risk of bias; downgraded if there was some or major concern for overall risk of bias). Evidence was downgraded for imprecision if 95% CIs overlapped the minimally important difference of 2250 pmol*min/L. Evidence was downgraded for indirectness due lack of available trial data. There is no general consensus on a clinically important size difference for 2-hour blood insulin incremental area under the curve (iAUC). Health Canada considers a 20% reduction in glucose iAUC to be the minimum physiologically relevant difference as per “Guideline Concerning the Safety and Physiological Effects of Novel Fibre Sources and Food Products Containing Them” (29). Extrapolating the 20% threshold in glucose iAUC, we calculated the MID of insulin iAUC by taking 20% of 11250 pmol*mins/L which is the median of insulin iAUC in participants with type 2 diabetes (~6200 pmol*mins/L) and healthy participants (~16300 pmol*mins/L) after a 75-g oral glucose tolerance test (32-34). Non-significant results are grey and not bolded. Trivial (significant) effects (<1 MID) or no effects have a white background; small important effects (>1 MID) have a light blue background; moderate effects (>2 MID) have a darker blue background; large effects (>5 to <10 MID) have a purple background; very large effects (>10 MID) have a black background. Confidence in the effect estimate is shown for each treatment comparison: high confidence ⊕⊕⊕⊕; moderate confidence ⊕⊕⊕; low confidence ⊕⊕; very low confidence ⊕

Table S21. *OVERALL CINeMA assessments of DELAYED COUPLING INTERVENTIONS examining the effect of non-nutritive sweetened beverages (NNS beverages) sweetened with individual or blends of non-nutritive sweeteners (NNS) and water on postprandial blood GLP-1 response in HEALTHY participants*

Comparison	Number of studies	Within-study bias	Reporting bias	Indirectness	Imprecision	Heterogeneity	Incoherence	Confidence rating	Reason(s) for downgrading
				DIRECT EVIDENCE					
**Ace-K + Aspartame + Sucralose:Ace-K + Sucralose**	1	No concerns	Low risk	No concerns	Some concerns	Some concerns	No concerns	Low	Imprecision, Heterogeneity
**Ace-K + Aspartame + Sucralose:Water**	1	No concerns	Low risk	No concerns	Some concerns	Some concerns	No concerns	Low	Imprecision, Heterogeneity
**Ace-K + Sucralose:Water**	2	No concerns	Low risk	No concerns	Some concerns	Some concerns	No concerns	Low	Imprecision, Heterogeneity
**Aspartame:Sucralose**	1	No concerns	Low risk	No concerns	Major concerns	No concerns	No concerns	Moderate	Imprecision
**Aspartame:Water**	1	No concerns	Low risk	No concerns	Major concerns	No concerns	No concerns	Moderate	Imprecision
**Saccharin:Water**	1	No concerns	Low risk	No concerns	Major concerns	No concerns	No concerns	Moderate	Imprecision
**Sucralose:Water**	3	No concerns	Low risk	No concerns	No concerns	Some concerns	No concerns	Moderate	Heterogeneity
				INDIRECT EVIDENCE					
**Ace-K + Aspartame + Sucralose:Aspartame**	0	No concerns	Low risk	No concerns	Major concerns	No concerns	No concerns	Moderate	Imprecision
**Ace-K + Aspartame + Sucralose:Saccharin**	0	No concerns	Low risk	No concerns	Major concerns	No concerns	No concerns	Moderate	Imprecision
**Ace-K + Aspartame + Sucralose:Sucralose**	0	No concerns	Low risk	No concerns	Some concerns	Some concerns	No concerns	Low	Imprecision, Heterogeneity
**Ace-K + Sucralose:Aspartame**	0	No concerns	Low risk	No concerns	Major concerns	No concerns	No concerns	Moderate	Imprecision
**Ace-K + Sucralose:Saccharin**	0	No concerns	Low risk	No concerns	Major concerns	No concerns	No concerns	Moderate	Imprecision
**Ace-K + Sucralose:Sucralose**	0	No concerns	Low risk	No concerns	Some concerns	Some concerns	No concerns	Low	Imprecision, Heterogeneity
**Aspartame:Saccharin**	0	No concerns	Low risk	No concerns	Major concerns	No concerns	No concerns	Moderate	Imprecision
**Saccharin:Sucralose**	0	No concerns	Low risk	No concerns	Major concerns	No concerns	No concerns	Moderate	Imprecision

Table S22. *GRADE assessments of DELAYED COUPLING INTERVENTIONS examining the effect of non-nutritive sweetened beverages (NNS beverages) sweetened with individual or blends of non-nutritive sweeteners (NNS) and water on postprandial blood GLP-1 response in participants with TYPE 2 DIABETES*

**Treatments**	**Effect Estimate**	**GRADE Assessments**						
		**1**	**2**	**3**	**4**	**5**	**6**	**Overall**
Aspartame vs. Sucralose	-483.260 (-1314.391, 347.871)	-	-	↓	↓	-	-	LOW ⊕⊕
Aspartame vs. Sucralose+Ace-K	-332.706 (-1360.600, 695.188)	-	-	↓	↓	-	-	LOW ⊕⊕
Aspartame vs. Water	-156.770 (-1127.779, 814.239)	-	-	↓	↓	-	-	LOW ⊕⊕
Sucralose vs. Sucralose+Ace-K	150.554 (-790.271, 1091.379)	-	-	↓	↓	-	-	LOW ⊕⊕
Sucralose vs. Water	326.490 (-551.829, 1204.809)	-	-	↓	↓	-	-	LOW ⊕⊕
Sucralose+Ace-K vs. Water	175.936 (-161.268, 513.141)	-	-	↓	↓	-	-	LOW ⊕⊕

Data reported as mean difference with 95% confidence intervals (CIs). Due to lack of available trial data, the CINeMA (Confidence in Network Meta-Analysis) web application was unable to make judgements for the six domains. Thus, manual judgements were made based on the CiNEMA framework (27, 28). GRADE domains refer to: (1), within-study bias; (2), reporting bias; (3), indirectness; (4), imprecision; (5) heterogeneity; and (6) incoherence. The risk of bias domain was assessed using the Cochrane RoB2 tool (no downgrade if there was no concern for overall risk of bias; downgraded if there was some or major concern for overall risk of bias). Evidence was downgraded for imprecision if 95% CIs overlapped the minimally important difference of 82 pmol*min/L. Evidence was downgraded for indirectness due lack of available trial data. There is no general consensus on a clinically important size difference for 2-hour blood GLP-1 incremental area under the curve (iAUC). Health Canada considers a 20% reduction in glucose iAUC to be the minimum physiologically relevant difference as per “Guideline Concerning the Safety and Physiological Effects of Novel Fibre Sources and Food Products Containing Them” (29). Extrapolating the 20% threshold in glucose iAUC, we calculated the MID of GLP-1 iAUC by taking 20% of 412 pmol*mins/L which is the median of the difference in GLP-1 iAUC among healthy participants (~95 pmol*mins/L) and those with type 2 diabetes (~633 pmol*mins/L) after a glucose or meal tolerance test (30). Significant results are bolded in black. Non-significant results are grey and not bolded. Trivial (significant) effects (<1 MID) or no effects have a white background; small important effects (>1 MID) have a light blue background; moderate effects (>2 MID) have a darker blue background; large effects (>5 to <10 MID) have a purple background; very large effects (>10 MID) have a black background. Confidence in the effect estimate is shown for each treatment comparison: high confidence ⊕⊕⊕⊕; moderate confidence ⊕⊕⊕; low confidence ⊕⊕; very low confidence ⊕

Table S23. *OVERALL CINeMA assessments of DELAYED COUPLING INTERVENTIONS examining the effect of non-nutritive sweetened beverages (NNS beverages) sweetened with individual or blends of non-nutritive sweeteners (NNS) and water on postprandial blood GIP response in HEALTHY participants*

Comparison	Number of studies	Within-study bias	Reporting bias	Indirectness	Imprecision	Heterogeneity	Incoherence	Confidence rating	Reason(s) for downgrading
**DIRECT EVIDENCE**									
**Ace-K + Aspartame + Sucralose:Ace-K + Sucralose**	1	No concerns	Low risk	No concerns	Major concerns	No concerns	No concerns	Moderate	Imprecision
**Ace-K + Aspartame + Sucralose:Water**	1	No concerns	Low risk	No concerns	Major concerns	No concerns	No concerns	Moderate	Imprecision
**Ace-K + Sucralose:Water**	2	No concerns	Low risk	No concerns	Major concerns	No concerns	No concerns	Moderate	Imprecision
**Saccharin:Water**	1	No concerns	Low risk	No concerns	Major concerns	No concerns	No concerns	Moderate	Imprecision
**Sucralose:Water**	2	No concerns	Low risk	No concerns	Major concerns	No concerns	No concerns	Moderate	Imprecision
**INDIRECT EVIDENCE**									
**Ace-K + Aspartame + Sucralose:Saccharin**	0	No concerns	Low risk	No concerns	Major concerns	No concerns	No concerns	Moderate	Imprecision
**Ace-K + Aspartame + Sucralose:Sucralose**	0	No concerns	Low risk	No concerns	Major concerns	No concerns	No concerns	Moderate	Imprecision
**Ace-K + Sucralose:Saccharin**	0	No concerns	Low risk	No concerns	Major concerns	No concerns	No concerns	Moderate	Imprecision
**Ace-K + Sucralose:Sucralose**	0	No concerns	Low risk	No concerns	Major concerns	No concerns	No concerns	Moderate	Imprecision
**Saccharin:Sucralose**	0	No concerns	Low risk	No concerns	Major concerns	No concerns	No concerns	Moderate	Imprecision

Table S24. *GRADE assessments of DELAYED COUPLING INTERVENTIONS examining the effect of non-nutritive sweetened beverages (NNS beverages) sweetened with individual or blends of non-nutritive sweeteners (NNS) and water on postprandial blood GLUCAGON response in HEALTHY participants*

**Treatments**	**Effect Estimate**	**GRADE Assessments**						
		**1**	**2**	**3**	**4**	**5**	**6**	**Overall**
Saccharin vs. Sucralose	-37.122 (-480.123, 405.879)	-	-	↓	↓	-	-	LOW ⊕⊕
Saccharin vs. Sucralose+Ace-K	-183.248 (-722.058, 355.561)	-	-	↓	↓	-	-	LOW ⊕⊕
Saccharin vs. Water	18.340 (-336.407, 373.087)	-	-	↓	↓	-	-	LOW ⊕⊕
Sucralose vs. Sucralose+Ace-K	-146.126 (-630.765, 338.512)	-	-	↓	↓	-	-	LOW ⊕⊕
Sucralose vs. Water	55.462 (-209.876, 320.800)	-	-	↓	↓	-	-	LOW ⊕⊕
Sucralose+Ace-K vs. Water	201.588 (-203.961, 607.137)	-	-	↓	↓	-	-	LOW ⊕⊕

Data reported as mean difference with 95% confidence intervals (CIs). Due to lack of available trial data, the CINeMA (Confidence in Network Meta-Analysis) web application was unable to make judgements for the six domains. Thus, manual judgements were made based on the CiNEMA framework (27, 28). GRADE domains refer to: (1), within-study bias; (2), reporting bias; (3), indirectness; (4), imprecision; (5) heterogeneity; and (6) incoherence. The risk of bias domain was assessed using the Cochrane RoB2 tool (no downgrade if there was no concern for overall risk of bias; downgraded if there was some or major concern for overall risk of bias). Evidence was downgraded for imprecision if 95% CIs overlapped the minimally important difference of 205 pmol*min/L. Evidence was downgraded for indirectness due lack of available trial data. There is no general consensus on a clinically important size difference for 2-hour blood glucagon incremental area under the curve (iAUC). Health Canada considers a 20% reduction in glucose iAUC to be the minimum physiologically relevant difference as per "Guideline Concerning the Safety and Physiological Effects of Novel Fibre Sources and Food Products Containing Them" (29). Extrapolating the 20% threshold in glucose iAUC, we calculated the MID of glucagon iAUC by taking 20% of 1025 pmol*mins/L which is the median value of glucagon iAUC (water vs. glucose comparisons) in healthy individuals (~850 pmol*mins/L) and those with type 2 diabetes (~1200 pmol*mins/L). Significant results are bolded in black. Non-significant results are grey and not bolded. Trivial (significant) effects (<1 MID) or no effects have a white background; small important effects (>1 MID) have a light blue background; moderate effects (>2 MID) have a darker blue background; large effects (>5 to <10 MID) have a purple background; very large effects (>10 MID) have a black background. Confidence in the effect estimate is shown for each treatment comparison: high confidence ⊕⊕⊕⊕; moderate confidence ⊕⊕⊕; low confidence ⊕⊕; very low confidence ⊕

Table S25. *GRADE assessments for outcomes with single direct trial comparisons*

Outcome	Population	Treatments	Effect Estimate	GRADE Assessments					
				1	2	3	4	5	Overall
Uncoupling interventions (Beverages were consumed without added energy or nutrients)									
Glucose (mmol*min/L)	IGT (N=20)	Aspartame vs. Glucose	357.7 [-25.1, 740.4]	-	↓	-	↓	-	⊕⊕ LOW
Insulin (pmol*min/L)	IGT (N=20)	Aspartame vs. Glucose	25335.8 [-13636.3, 64307.7]	-	↓	-	↓	-	⊕⊕ LOW
PYY (pmol*min/L)	Healthy (N=8)	Sucralose vs. Water	0.1 [-442.8, 442.8]	-	↓	-	↓	-	⊕⊕ LOW
Delayed coupling interventions (Beverages were consumed as a preload prior to added energy and nutrients as carbohydrate)									
Glucose (mmol*min/L)	PLWO (N=11)	Sucralose vs. Water	-30.0 [-87.3, 27.3]	-	↓	-	↓	-	⊕⊕ LOW
	T1D (N=9)	Ace-K+Sucralose vs. Water	-64.0 [-233.1, 105.3]	-	↓	-	↓	-	⊕⊕ LOW
Insulin (pmol*min/L)	PLWO (N=11)	Sucralose vs. Water	-8200.0 [-31642.4, 15242.4]	-	↓	-	↓	-	⊕⊕ LOW
	T1D (N=9)	Ace-K+Sucralose vs. Water	-543.9 [-1779.2, 691.5]	-	↓	-	↓	-	⊕⊕ LOW
GLP-1 (pmol*min/L)	T1D (N=9)	Ace-K+Sucralose vs. Water	-433.5 [-1134.4, 267.5]	-	↓	-	↓	-	⊕⊕ LOW
GIP (pmol*min/L)	T1D (N=9)	Ace-K+Sucralose vs. Water	-934.8 [-2215.7, 346.1]	-	↓	-	↓	-	⊕⊕ LOW
	T2D (N=9)	Ace-K+Sucralose vs. Water	-479.8 [-2181.5, 1222.1]	-	↓	-	↓	-	⊕⊕ LOW
Glucagon (pmol*min/L)	T1D (N=9)	Ace-K+Sucralose vs. Water	-20.3 [-97.4, 46.8]	-	↓	-	↓	-	⊕⊕ LOW
	T2D (N=10)	Ace-K+Sucralose vs. Water	241.9 [-38.8, 522.5]	-	↓	-	↓	-	⊕⊕ LOW

Data reported as mean difference with 95% confidence intervals (CIs). Due to lack of available trials, the CINeMA (Confidence in Network Meta-Analysis) web application could not be utilized. Confidence in the effect estimates were assessed using the traditional GRADE (Grading of Recommendations, Assessment, Development and Evaluations) approach (35). GRADE domains refer to: (1), risk of bias; (2), imprecision; (3), inconsistency; (4), indirectness; and (5), publication bias. The following minimally important differences (MID) were utilized: glucose (100 mmol*min/L), insulin (2250 pmol*mins/L), GLP-1 (82 pmol*mins/L), GIP (94 pmol*mins/L), glucagon (205 pmol*mins/L) and PYY (200 pmol*mins/L). The risk of bias domain was assessed using the Cochrane RoB2 tool (no downgrade if there was no concern for overall risk of bias; downgraded if there was some or major concern for overall risk of bias). Evidence was double downgraded for imprecision due to availability of only a single trial. Inconsistency and publication bias could not be assessed due to limited trial data and thus, no downgrades were applied. ↓ downgrade; "-", no downgrade. IGT, impaired glucose tolerance; PLWO, participants living with obesity; T1D, type 1 diabetes; T2D, type 2 diabetes. 2

Figure S1. *Formulas used to compute incremental area under the curve (iAUC) for primary and secondary outcomes*

Incremental area under the curve (iAUC)

$$iAUC = \frac{(A+B)\times t}{2} + \frac{(B+C)\times t}{2} + \frac{(C+D)\times t}{2} + \frac{(D+E)\times t}{2}\ etc\ \ldots$$

Standard error (SE) computation for iAUC

$$SE\ of\ iAUC = \sqrt{t^2 + average\ SE^2}$$

The iAUC for glucose and insulin was calculated geometrically using the trapezoid rule (36) where A, B, C, D, and E represent positive glucose and insulin increments, and t is the time interval between blood samples. The standard error of the iAUC was computed using the propagation of error approach for area (37). t represents the duration of follow-up and average SE is the average of the SE at individual time points on the glucose and insulin curves.

***Figure S2**. Flow of literature*

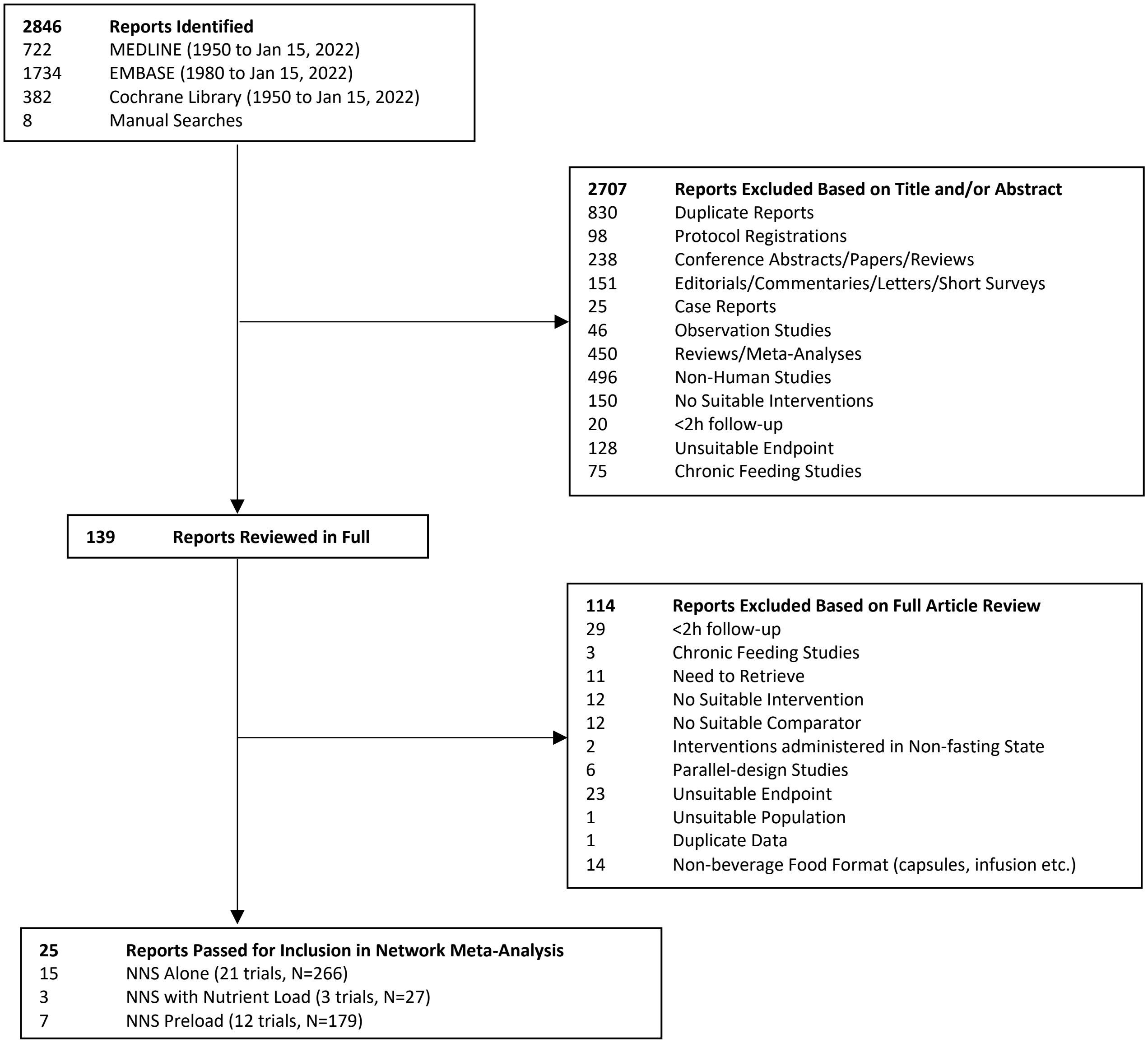

Figure S3. *Individual (top) and summary (bottom) risk of bias assessments of studies with UNCOUPLING INTERVENTIONS*

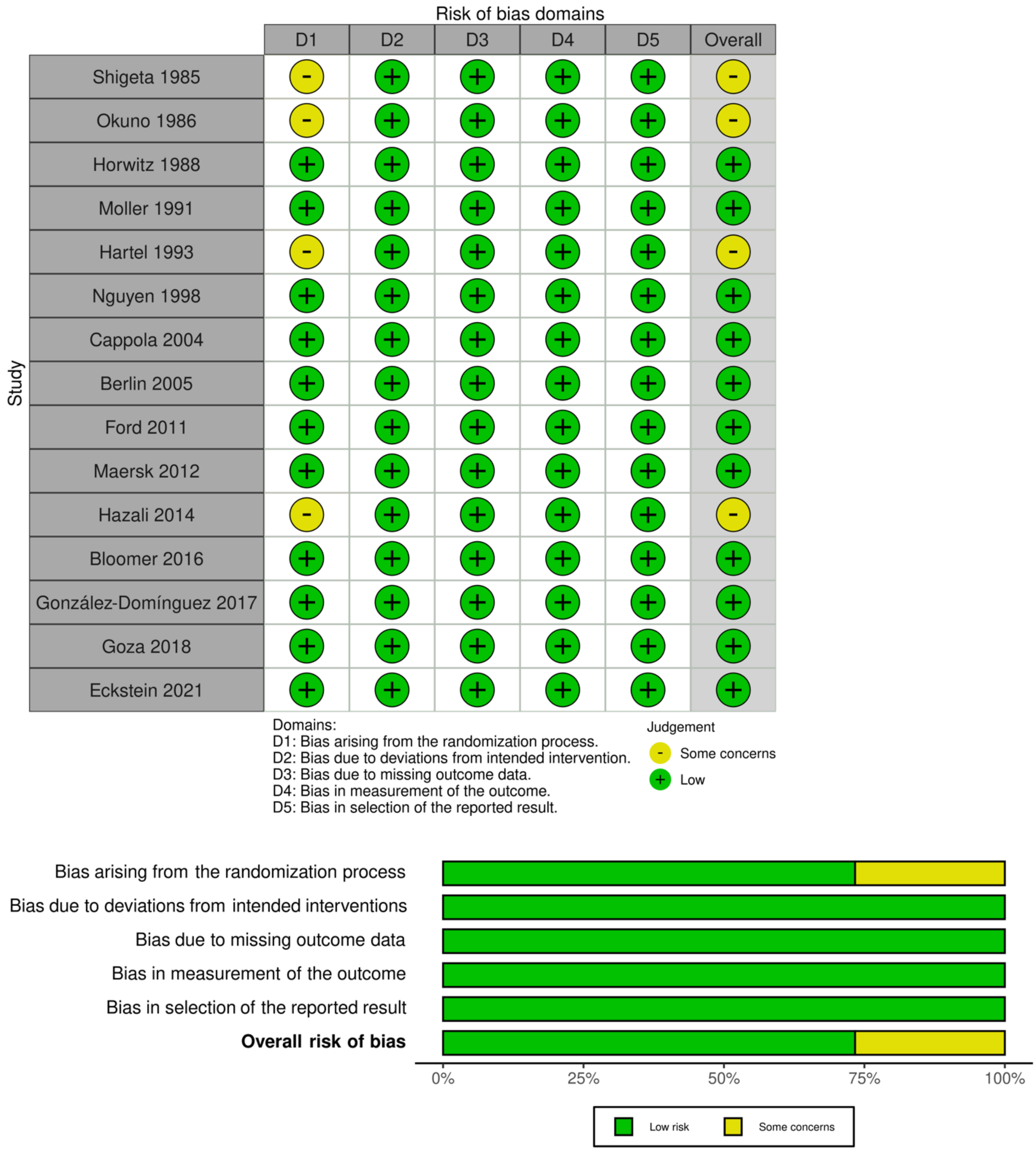

__Figure S4.__ Individual (top) and summary (bottom) risk of bias assessment of studies with COUPLING INTERVENTIONS

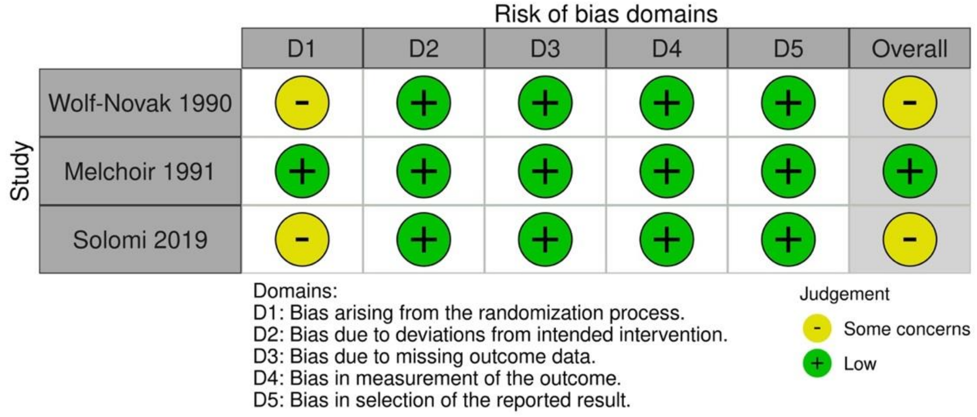

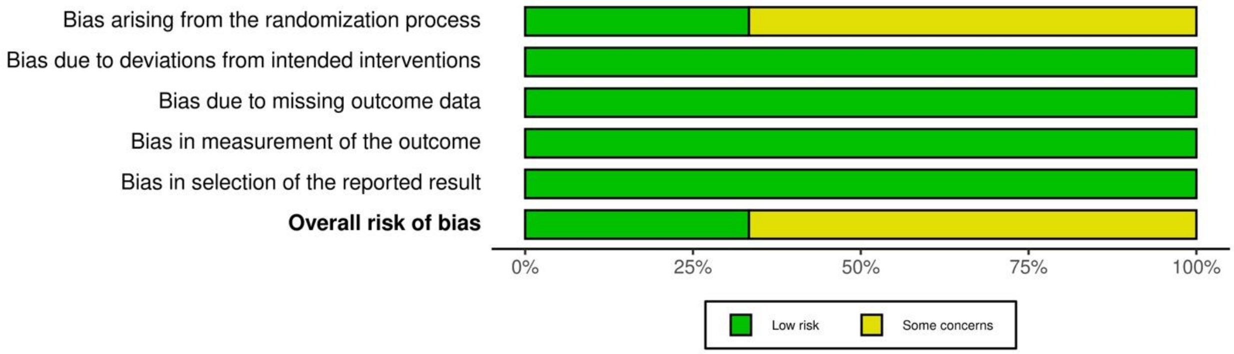

Figure S5. *Individual (top) and summary (bottom) risk of bias assessment of studies with DELAYED COUPLING INTERVENTIONS*

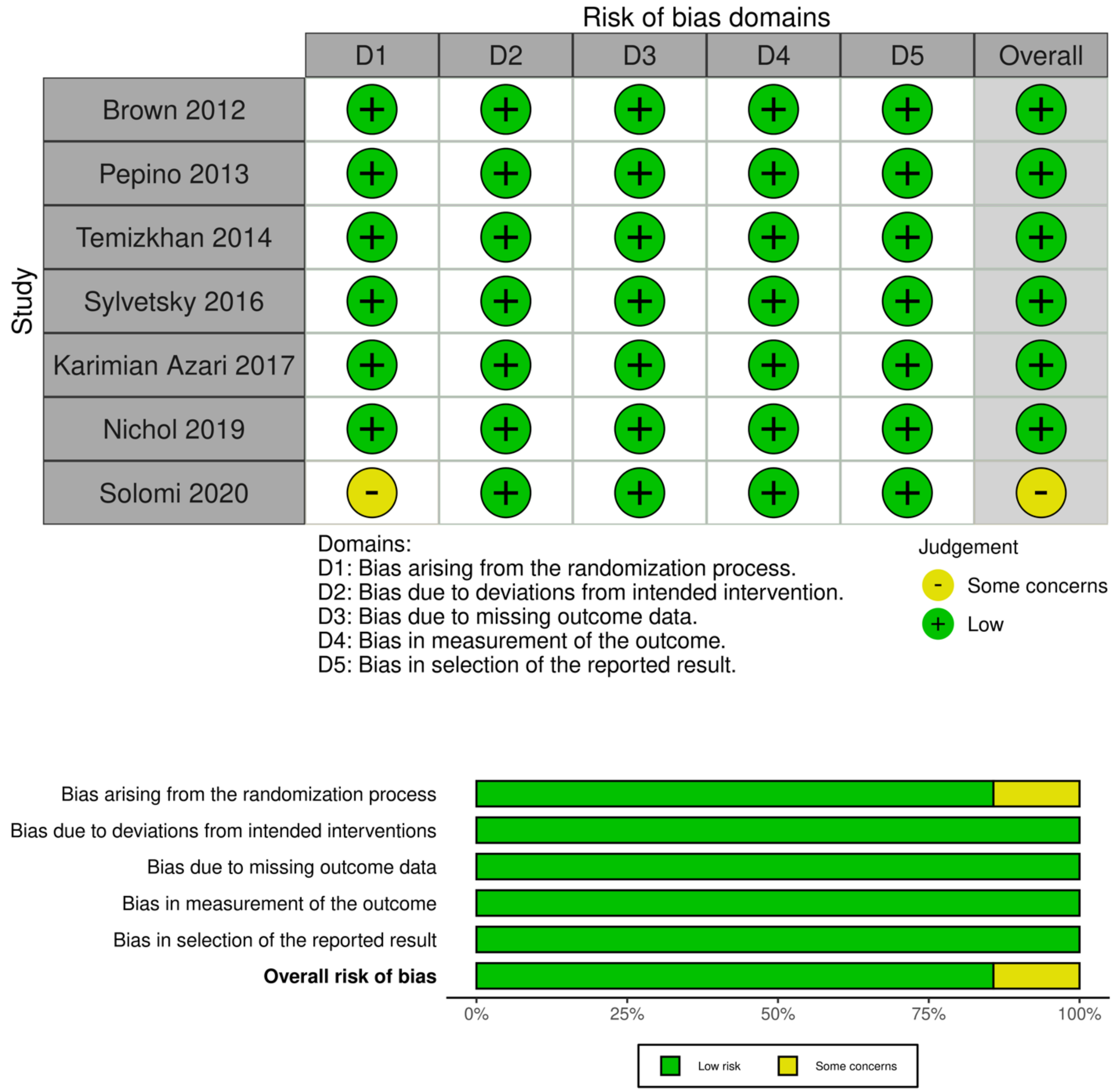

Figure S6. *Risk of bias assessment of UNCOUPLING INTERVENTIONS examining non-nutritive sweetened beverages (NNS beverages) sweetened with individual or blends of non-nutritive sweeteners (NNS), water, and sugar-sweetened beverages (SSBs) sweetened with caloric sweeteners on postprandial blood GLUCOSE response in HEALTHY participants*

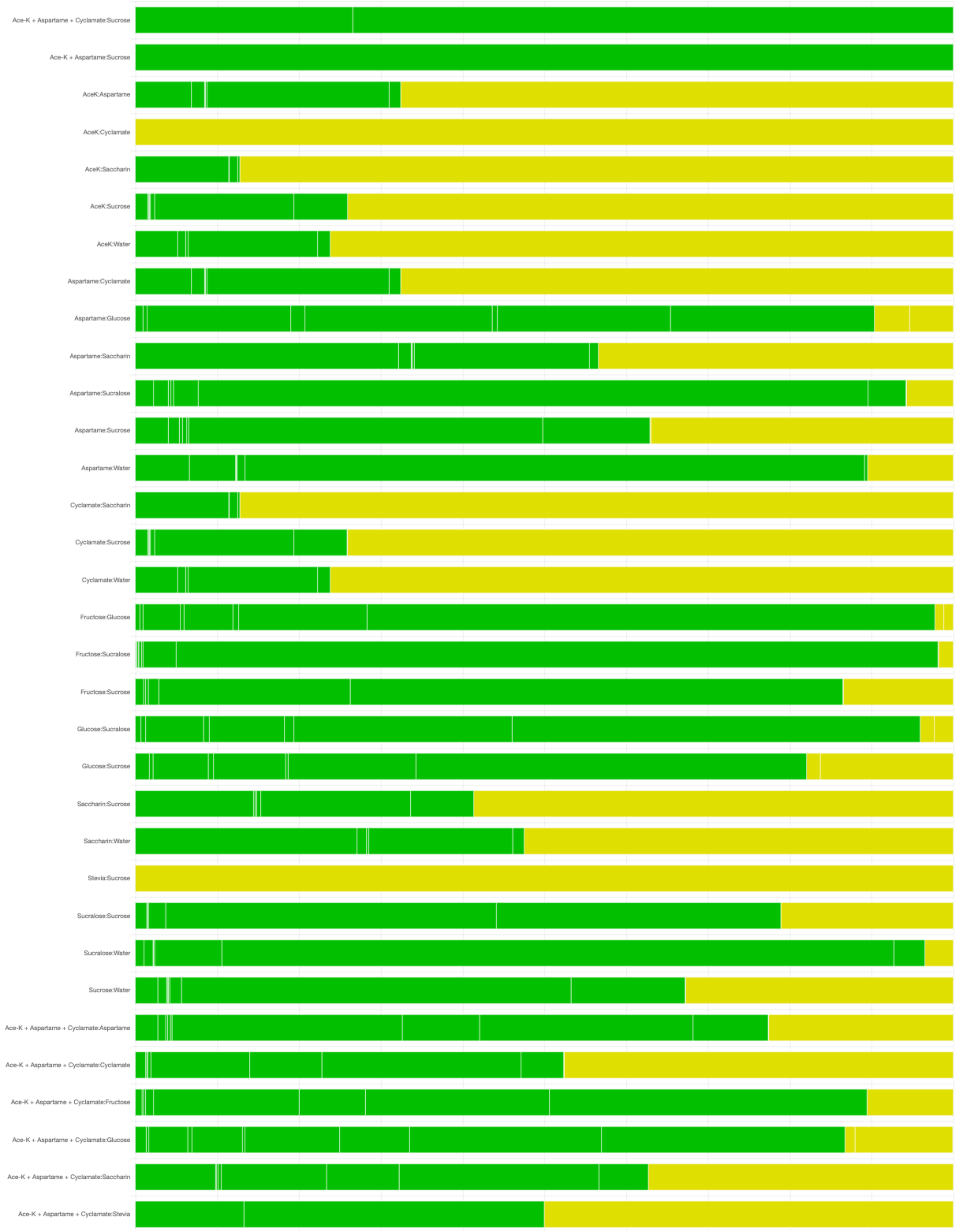

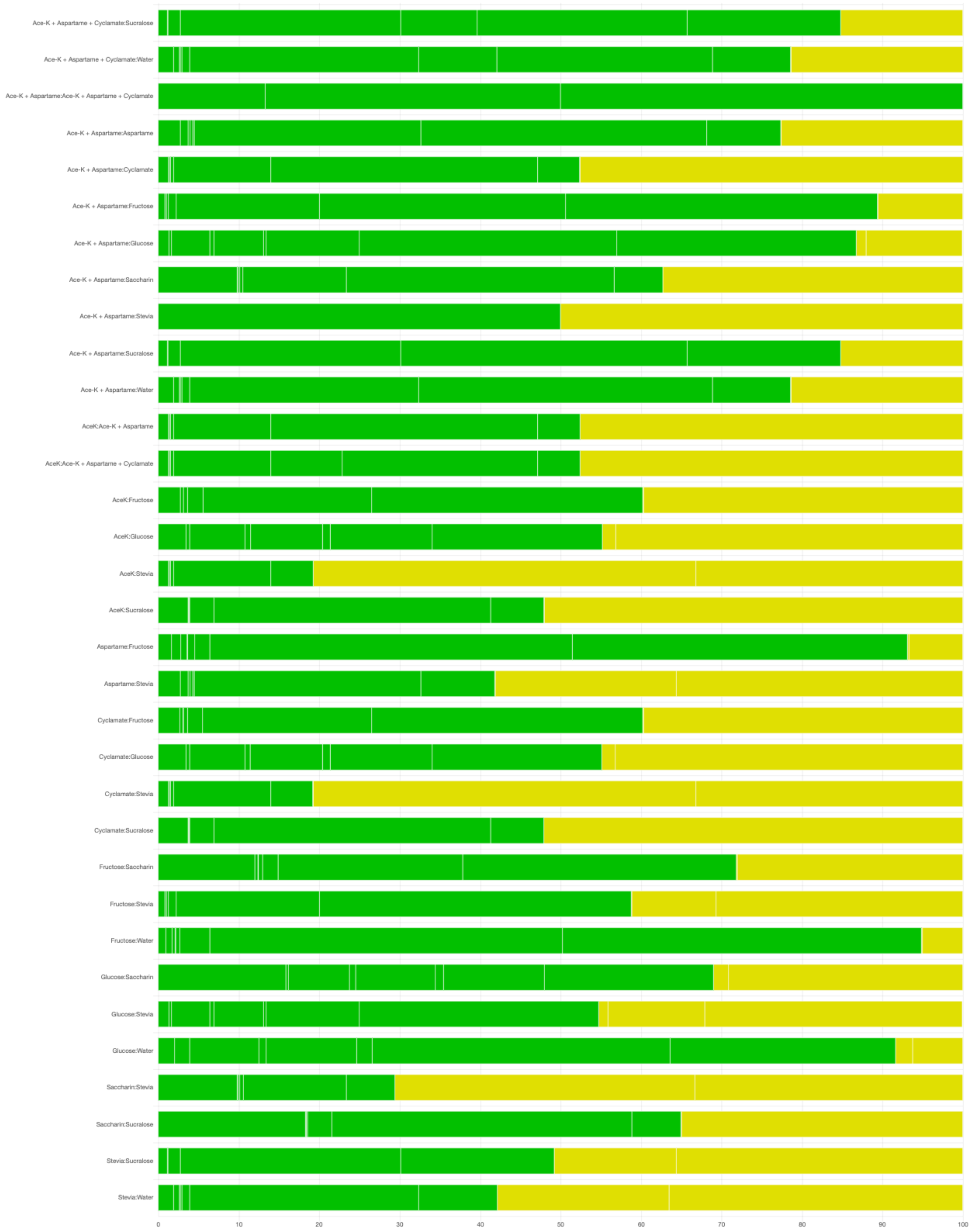

Green = no concerns; yellow = some concerns; red = major concerns

Figure S7. *CINeMA output for the IMPRECISION domain of UNCOUPLING INTERVENTIONS examining non-nutritive sweetened beverages (NNS beverages) sweetened with individual or blends of non-nutritive sweeteners (NNS), water, and sugar-sweetened beverages (SSBs) sweetened with caloric sweeteners on postprandial blood GLUCOSE response in HEALTHY participants*

Imprecision

Define clinically important size of effect: Mean difference 100

Relative effect estimates below ***-100.000*** *and above* ***100.000*** *are considered clinically important.*

Comparison	Evidence	NMA estimate	95% Confidence interval		Imprecision judgment
Ace-K + Aspartame:Sucrose	mixed	-48.167	(-73.331,-23.002)	*Confidence interval does not cross clinically important effect*	No concerns
Ace-K + Aspartame + Cyclamate:Sucrose	mixed	-53.827	(-95.998,-11.656)	*Confidence interval does not cross clinically important effect*	No concerns
AceK:Aspartame	mixed	-2.031	(-34.988,30.925)	*Confidence interval does not cross clinically important effect*	No concerns
AceK:Cyclamate	mixed	2.280	(-35.056,39.616)	*Confidence interval does not cross clinically important effect*	No concerns
AceK:Saccharin	mixed	-9.113	(-44.285,26.058)	*Confidence interval does not cross clinically important effect*	No concerns
AceK:Sucrose	mixed	-104.809	(-146.503,-63.114)	*Confidence interval does not cross clinically important effect*	No concerns
AceK:Water	mixed	7.439	(-24.701,39.580)	*Confidence interval does not cross clinically important effect*	No concerns
Aspartame:Cyclamate	mixed	4.311	(-28.787,37.410)	*Confidence interval does not cross clinically important effect*	No concerns
Aspartame:Glucose	mixed	-237.816	(-293.324,-182.307)	*Confidence interval does not cross clinically important effect*	No concerns
Aspartame:Saccharin	mixed	-7.082	(-33.714,19.550)	*Confidence interval does not cross clinically important effect*	No concerns
Aspartame:Sucralose	mixed	2.680	(-15.319,20.680)	*Confidence interval does not cross clinically important effect*	No concerns
Aspartame:Sucrose	mixed	-102.777	(-135.030,-70.525)	*Confidence interval does not cross clinically important effect*	No concerns
Aspartame:Water	mixed	9.471	(-1.953,20.895)	*Confidence interval does not cross clinically important effect*	No concerns
Cyclamate:Saccharin	mixed	-11.393	(-46.698,23.912)	*Confidence interval does not cross clinically important effect*	No concerns
Cyclamate:Sucrose	mixed	-107.089	(-148.896,-65.282)	*Confidence interval does not cross clinically important effect*	No concerns
Cyclamate:Water	mixed	5.159	(-27.127,37.446)	*Confidence interval does not cross clinically important effect*	No concerns
Fructose:Glucose	mixed	-199.294	(-260.661,-137.927)	*Confidence interval does not cross clinically important effect*	No concerns
Fructose:Sucralose	mixed	41.202	(17.206,65.199)	*Confidence interval does not cross clinically important effect*	No concerns
Fructose:Sucrose	mixed	-64.255	(-104.338,-24.172)	*Confidence interval does not cross clinically important effect*	No concerns
Glucose:Sucralose	mixed	240.496	(183.900,297.092)	*Confidence interval does not cross clinically important effect*	No concerns
Glucose:Sucrose	mixed	135.038	(71.652,198.425)	*Confidence interval does not cross clinically important effect*	No concerns
Saccharin:Sucrose	mixed	-95.695	(-133.868,-57.523)	*Confidence interval does not cross clinically important effect*	No concerns
Saccharin:Water	mixed	16.553	(-9.559,42.664)	*Confidence interval does not cross clinically important effect*	No concerns
Stevia:Sucrose	mixed	-59.000	(-101.962,-16.038)	*Confidence interval does not cross clinically important effect*	No concerns

Comparison Sucralose:Sucrose
Evidence: mixed
NMA estimate: -105.458
95% Confidence interval:
Confidence (-137.729,-73.186)
interval does not cross clinically important effect
Imprecision judgment
No concerns

Comparison Sucralose:Water
Evidence: mixed
NMA estimate: 6.790
95% Confidence interval:
Confidence interval (-7.610,21.191)
does not cross clinically important effect
Imprecision judgment
No concerns

Comparison Sucrose:Water
Evidence: mixed
NMA estimate: 112.248
95% Confidence interval:
Confidence interval (81.526,142.970)
does not cross clinically important effect
Imprecision judgment
No concerns

Comparison
Ace-K + Aspartame:Ace-K + Aspartame + Cyclamate
Evidence: indirect
NMA estimate: 5.660
95% Confidence interval:
Confidence interval (-43.448,54.769)
does not cross clinically important effect
Imprecision judgment
No concerns

Comparison
Evidence: AceK:Ace-K + Aspartame
indirect
NMA estimate: -56.642
95% Confidence interval:
Confidence (-105.342,-7.942)
interval does not cross clinically important effect
Imprecision judgment
No concerns

Comparison
Ace-K + Aspartame:Aspartame
Evidence: indirect
NMA estimate: 54.611
95% Confidence interval:
Confidence interval (13.703,95.519)
does not cross clinically important effect
Imprecision judgment
No concerns

Comparison
Ace-K + Aspartame:Cyclamate
Evidence: indirect
NMA estimate: 58.922
95% Confidence interval:
Confidence interval (10.126,107.718)
does not cross clinically important effect
Imprecision judgment
No concerns

Comparison
Ace-K + Aspartame:Fructose
Evidence: indirect
NMA estimate: 16.089
95% Confidence interval:
Confidence interval (-31.239,63.416)
does not cross clinically important effect
Imprecision judgment
No concerns

Comparison
Ace-K + Aspartame:Glucose
Evidence: indirect
NMA estimate: -183.205
95% Confidence interval:
Confidence (-251.404,-115.007)
interval does not cross clinically important effect
Imprecision judgment
No concerns

Comparison
Ace-K + Aspartame:Saccharin
Evidence: indirect
NMA estimate: 47.529
95% Confidence interval:
Confidence interval (1.808,93.249)
does not cross clinically important effect
Imprecision judgment
No concerns

Comparison
Evidence:Ace-K + Aspartame:Stevia
indirect
NMA estimate: 10.833
95% Confidence interval:
Confidence interval (-38.956,60.623)
does not cross clinically important effect
Imprecision judgment
No concerns

Comparison
Ace-K + Aspartame:Sucralose
Evidence: indirect
NMA estimate: 57.291
95% Confidence interval:
Confidence interval (16.368,98.214)
does not cross clinically important effect
Imprecision judgment
No concerns

Comparison
Evidence: Ace-K + Aspartame:Water
indirect
NMA estimate: 64.081
95% Confidence interval:
Confidence interval (24.369,103.794)
does not cross clinically important effect
Imprecision judgment
No concerns

Comparison
AceK:Ace-K + Aspartame + Cyclamate
Evidence: indirect
NMA estimate: -50.982
95% Confidence interval:
Confidence interval (-110.284,8.321)
extends into clinically important effects
Imprecision judgment
Some concerns

Comparison
Ace-K + Aspartame + Cyclamate:Aspartame
Evidence: indirect
NMA estimate: 48.950
95% Confidence interval:
Confidence interval (-4.141,102.041)
extends into clinically important effects
Imprecision judgment
Some concerns

Comparison
Ace-K + Aspartame + Cyclamate:Cyclamate
Evidence: indirect
NMA estimate: 53.262
95% Confidence interval:
Confidence interval (-6.120,112.643)
extends into clinically important effects
Imprecision judgment
Some concerns

Comparison
Ace-K + Aspartame + Cyclamate:Fructose
Evidence: indirect
NMA estimate: 10.428
95% Confidence interval:
Confidence interval (-47.753,68.609)
does not cross clinically important effect
Imprecision judgment
No concerns

Comparison
Ace-K + Aspartame + Cyclamate:Glucose
Evidence: indirect
NMA estimate: -188.866
95% Confidence interval:
Confidence (-264.998,-112.733)
interval does not cross clinically important effect
Imprecision judgment
No concerns

Comparison
Ace-K + Aspartame + Cyclamate:Saccharin
Evidence: indirect
NMA estimate: 41.868
95% Confidence interval:
Confidence interval (-15.013,98.750)
does not cross clinically important effect
Imprecision judgment
No concerns

Comparison
Ace-K + Aspartame + Cyclamate:Stevia
Evidence: indirect
NMA estimate: 5.173
95% Confidence interval:
Confidence interval (-55.028,65.373)
does not cross clinically important effect
Imprecision judgment
No concerns

Comparison
Ace-K + Aspartame + Cyclamate:Sucralose
Evidence: indirect
NMA estimate: 51.630
95% Confidence interval:
Confidence interval (-1.472,104.733)
extends into clinically important effects
Imprecision judgment
Some concerns

Comparison
Ace-K + Aspartame + Cyclamate:Water
Evidence: indirect
NMA estimate: 58.421
95% Confidence interval:
Confidence interval (6.246,110.596)
does not cross clinically important effect
Imprecision judgment
No concerns

Comparison AceK:Fructose
Evidence: indirect
NMA estimate: -40.553
95% Confidence interval:
Confidence interval (-82.931,1.824)
does not cross clinically important effect
Imprecision judgment
No concerns

Comparison AceK:Glucose
Evidence: indirect
NMA estimate: -239.847
95% Confidence interval:
Confidence (-303.945,-175.749)
interval does not cross clinically important effect
Imprecision judgment
No concerns

Comparison AceK:Stevia
Evidence: indirect
NMA estimate: -45.809
95% Confidence interval:
Confidence interval extends into clinically important effects (-105.676,14.059)
Imprecision judgment
Some concerns

Comparison AceK:Sucralose
Evidence: indirect
NMA estimate: 0.649
95% Confidence interval:
Confidence interval (-34.325,35.623) *does not cross clinically important effect*
Imprecision judgment
No concerns

Comparison Aspartame:Fructose
Evidence: indirect
NMA estimate: -38.522
95% Confidence interval:
Confidence interval (-68.479,-8.565) *does not cross clinically important effect*
Imprecision judgment
No concerns

Comparison Aspartame:Stevia
Evidence: indirect
NMA estimate: -43.777
95% Confidence interval:
Confidence interval (-97.498,9.944) *does not cross clinically important effect*
Imprecision judgment
No concerns

Comparison Cyclamate:Fructose
Evidence: indirect
NMA estimate: -42.833
95% Confidence interval:
Confidence interval (-85.321,-0.345) *does not cross clinically important effect*
Imprecision judgment
No concerns

Comparison Cyclamate:Glucose
Evidence: indirect
NMA estimate: -242.127
95% Confidence interval:
Confidence interval does not cross clinically important effect (-306.299,-177.956)
Imprecision judgment
No concerns

Comparison Cyclamate:Stevia
Evidence: indirect
NMA estimate: -48.089
95% Confidence interval:
Confidence interval extends into clinically important effects (-108.035,11.857)
Imprecision judgment
Some concerns

Comparison Cyclamate:Sucralose
Evidence: indirect
NMA estimate: -1.631
95% Confidence interval:
Confidence interval (-36.739,33.477) *does not cross clinically important effect*
Imprecision judgment
No concerns

Comparison Fructose:Saccharin
Evidence: indirect
NMA estimate: 31.440
95% Confidence interval:
Confidence interval (-6.609,69.490) *does not cross clinically important effect*
Imprecision judgment
No concerns

Comparison Fructose:Stevia
Evidence: indirect
NMA estimate: -5.255
95% Confidence interval:
Confidence interval (-64.012,53.502) *does not cross clinically important effect*
Imprecision judgment
No concerns

Comparison Fructose:Water
Evidence: indirect
NMA estimate: 47.993
95% Confidence interval:
Confidence interval (20.043,75.943) *does not cross clinically important effect*
Imprecision judgment
No concerns

Comparison Glucose:Saccharin
Evidence: indirect
NMA estimate: 230.734
95% Confidence interval:
Confidence interval does not cross clinically important effect (169.572,291.896)
Imprecision judgment
No concerns

Comparison Glucose:Stevia
Evidence: indirect
NMA estimate: 194.038
95% Confidence interval:
Confidence interval does not cross clinically important effect (117.465,270.612)
Imprecision judgment
No concerns

Comparison Glucose:Water
Evidence: indirect
NMA estimate: 247.287
95% Confidence interval:
Confidence interval does not cross clinically important effect (191.300,303.273)
Imprecision judgment
No concerns

Comparison Saccharin:Stevia
Evidence: indirect
NMA estimate: -36.696
95% Confidence interval:
Confidence interval (-94.166,20.775) *does not cross clinically important effect*
Imprecision judgment
No concerns

Comparison Saccharin:Sucralose
Evidence: indirect
NMA estimate: 9.762
95% Confidence interval:
Confidence interval (-19.815,39.340) *does not cross clinically important effect*
Imprecision judgment
No concerns

Comparison Stevia:Sucralose
Evidence: indirect
NMA estimate: 46.458
95% Confidence interval:
Confidence interval (-7.275,100.190) *extends into clinically important effects*
Imprecision judgment
Some concerns

Comparison Stevia:Water
Evidence: indirect
NMA estimate: 53.248
95% Confidence interval:
Confidence interval (0.432,106.064) *does not cross clinically important effect*
Imprecision judgment
No concerns

Relative effect estimates below -100.0 and above 100.0 mmol*min/L (minimal important difference; MID) were considered clinically important. Judgements for IMPRECISION were assigned by the CiNEMA algorithm (27, 28). There is no general consensus on a clinically important size difference for 2-hour blood glucose incremental area under the curve (iAUC). Health Canada considers a 20% reduction in glucose iAUC to be the minimum physiologically relevant difference as per “Guideline Concerning the Safety and Physiological Effects of Novel Fibre Sources and Food Products Containing Them” (29). Thus, we calculated the MID of glucose iAUC by taking 20% of 500 mmol*mins/L which is the median of glucose iAUC in healthy participants (~300 mmol*mins/L) and those with type 2 diabetes (~700 mmol*mins/L) after a 75-g oral glucose tolerance test (32-34).

Figure S8. *CINeMA output for the HETEROGENEITY domain of UNCOUPLING INTERVENTIONS examining non-nutritive sweetened beverages (NNS beverages) sweetened with individual or blends of non-nutritive sweeteners (NNS), water, and sugar-sweetened beverages (SSBs) sweetened with caloric sweeteners on postprandial blood GLUCOSE response in HEALTHY participants*

Heterogeneity

Define clinically important size of effect: Mean difference 100

Relative effect estimates below ***-100.000*** *and above* ***100.000*** *are considered clinically important.*
Importance of heterogeneity depends on the variability of effects in relation to a clinically important size of effect

The estimated value of between-study variance for the network meta-analysis is **0.000**

Comparison Ace-K + Aspartame:Sucrose
Evidence: mixed
NMA estimate: -48.167
95% intervals for NMA estimate
Confidence interval: (-73.331,-23.002)
Prediction interval: (-76.425,-19.908)
Confidence and prediction intervals ***agree*** *in relation to clinically important effect*
Heterogeneity judgment: No concerns

Comparison Ace-K + Aspartame + Cyclamate:Sucrose
Evidence: mixed
NMA estimate: -53.827
95% intervals for NMA estimate
Confidence interval: (-95.998,-11.656)
Prediction interval: (-101.184,-6.470)
Prediction interval ***extends into clinically important or unimportant effects***
Heterogeneity judgment: Some concerns

Comparison AceK:Aspartame
Evidence: mixed
NMA estimate: -2.031
95% intervals for NMA estimate
Confidence interval: (-34.988,30.925)
Prediction interval: (-39.040,34.977)
Confidence and prediction intervals ***agree*** *in relation to clinically important effect*
Heterogeneity judgment: No concerns

Comparison AceK:Cyclamate
Evidence: mixed
NMA estimate: 2.280
95% intervals for NMA estimate
Confidence interval: (-35.056,39.616)
Prediction interval: (-39.648,44.208)
Confidence and prediction intervals ***agree*** *in relation to clinically important effect*
Heterogeneity judgment: No concerns

Comparison AceK:Saccharin
Evidence: mixed
NMA estimate: -9.113
95% intervals for NMA estimate
Confidence interval: (-44.285,26.058)
Prediction interval: (-48.610,30.383)
Confidence and prediction intervals ***agree*** *in relation to clinically important effect*
Heterogeneity judgment: No concerns

Comparison AceK:Sucrose
Evidence: mixed
NMA estimate: -104.809
95% intervals for NMA estimate
Confidence interval: (-146.503,-63.114)
Prediction interval: (-151.630,-57.987)
Confidence and prediction intervals ***agree*** *in relation to clinically important effect*
Heterogeneity judgment: No concerns

Comparison AceK:Water
Evidence: mixed
NMA estimate: 7.439
95% intervals for NMA estimate
Confidence interval: (-24.701,39.580)
Prediction interval: (-28.653,43.532)
Confidence and prediction intervals ***agree*** *in relation to clinically important effect*
Heterogeneity judgment: No concerns

Comparison Aspartame:Cyclamate
Evidence: mixed
NMA estimate: 4.311
95% intervals for NMA estimate
Confidence interval: (-28.787,37.410)
Prediction interval: (-32.857,41.480)
Confidence and prediction intervals ***agree*** *in relation to clinically important effect*
Heterogeneity judgment: No concerns

Comparison Aspartame:Glucose
Evidence: mixed
NMA estimate: -237.816
95% intervals for NMA estimate
Confidence interval: (-293.324,-182.307)
Prediction interval: (-300.150,-175.481)
Confidence and prediction intervals ***agree*** *in relation to clinically important effect*
Heterogeneity judgment: No concerns

Comparison Aspartame:Saccharin
Evidence: mixed
NMA estimate: -7.082
95% intervals for NMA estimate
Confidence interval: (-33.714,19.550)
Prediction interval: (-36.989,22.825)
Confidence and prediction intervals ***agree*** *in relation to clinically important effect*
Heterogeneity judgment: No concerns

Comparison Aspartame:Sucralose
Evidence: mixed
NMA estimate: 2.680
95% intervals for NMA estimate
Confidence interval: (-15.319,20.680)
Prediction interval: (-17.533,22.894)
Confidence and prediction intervals ***agree*** *in relation to clinically important effect*
Heterogeneity judgment: No concerns

Comparison Aspartame:Sucrose
Evidence: mixed
NMA estimate: -102.777
95% intervals for NMA estimate
Confidence interval: (-135.030,-70.525)
Prediction interval: (-138.996,-66.558)
Confidence and prediction intervals ***agree*** *in relation to clinically important effect*
Heterogeneity judgment: No concerns

Comparison Aspartame:Water
Evidence: mixed
NMA estimate: 9.471
95% intervals for NMA estimate
Confidence interval: (-1.953,20.895)
Prediction interval: (-3.358,22.300)
Confidence and prediction intervals ***agree*** *in relation to clinically important effect*
Heterogeneity judgment: No concerns

Comparison Cyclamate:Saccharin
Evidence: mixed
NMA estimate: -11.393
95% intervals for NMA estimate
Confidence interval: (-46.698,23.912)
Prediction interval: (-51.039,28.253)
Confidence and prediction intervals ***agree*** *in relation to clinically important effect*
Heterogeneity judgment: No concerns

Comparison Cyclamate:Sucrose
Evidence: mixed
NMA estimate: -107.089
95% intervals for NMA estimate
Confidence interval: (-148.896,-65.282)
Prediction interval: (-154.037,-60.141)
Confidence and prediction intervals ***agree*** *in relation to clinically important effect*
Heterogeneity judgment: No concerns

Comparison Cyclamate:Water
Evidence: mixed
NMA estimate: 5.159
95% intervals for NMA estimate
Confidence interval: (-27.127,37.446)
Prediction interval: (-31.097,41.416)
Confidence and prediction intervals ***agree*** *in relation to clinically important effect*
Heterogeneity judgment: No concerns

(continued on next page)

Comparison **Fructose:Glucose**
Evidence: mixed
NMA estimate: -199.294
95% intervals for NMA estimate
Confidence interval:
Prediction (-260.661,-137.927)
interval: (-268.207,-130.380)
*Confidence and prediction intervals **agree** in relation to clinically important effect*
Heterogeneity judgment
No concerns

Comparison **Fructose:Sucralose**
Evidence: mixed
NMA estimate: 41.202
95% intervals for NMA estimate
Confidence interval: (17.206,65.199)
Prediction interval: (14.255,68.150)
*Confidence and prediction intervals **agree** in relation to clinically important effect*
Heterogeneity judgment
No concerns

Comparison **Fructose:Sucrose**
Evidence: mixed
NMA estimate: -64.255
95% intervals for NMA estimate
Confidence interval:
Prediction (-104.338,-24.172)
interval: (-109.267,-19.243)
*Confidence and prediction intervals **agree** in relation to clinically important effect*
Heterogeneity judgment
No concerns

Comparison **Glucose:Sucralose**
Evidence: mixed
NMA estimate: 240.496
95% intervals for NMA estimate
Confidence interval:
Prediction (183.900,297.092)
interval: (176.940,304.052)
*Confidence and prediction intervals **agree** in relation to clinically important effect*
Heterogeneity judgment
No concerns

Comparison **Glucose:Sucrose**
Evidence: mixed
NMA estimate: 135.038
95% intervals for NMA estimate
Confidence interval:
Prediction interval: (71.652,198.425)
Confidence and (63.858,206.220) *prediction intervals **agree** in relation to clinically important effect*
Heterogeneity judgment
No concerns

Comparison **Saccharin:Sucrose**
Evidence: mixed
NMA estimate: -95.695
95% intervals for NMA estimate
Confidence interval:
Prediction (-133.868,-57.523)
interval: (-138.562,-52.829)
*Confidence and prediction intervals **agree** in relation to clinically important effect*
Heterogeneity judgment
No concerns

Comparison **Saccharin:Water**
Evidence: mixed
NMA estimate: 16.553
95% intervals for NMA estimate
Confidence interval: (-9.559,42.664)
Prediction interval: (-12.770,45.875)
*Confidence and prediction intervals **agree** in relation to clinically important effect*
Heterogeneity judgment
No concerns

Comparison **Stevia:Sucrose**
Evidence: mixed
NMA estimate: -59.000
95% intervals for NMA estimate
Confidence interval:
Prediction (-101.962,-16.038)
interval: (-107.245,-10.755)
*Confidence and prediction intervals **agree** in relation to clinically important effect*
Heterogeneity judgment
No concerns

Comparison **Sucralose:Sucrose**
Evidence: mixed
NMA estimate: -105.458
95% intervals for NMA estimate
Confidence interval:
Prediction (-137.729,-73.186)
interval: (-141.698,-69.218)
*Confidence and prediction intervals **agree** in relation to clinically important effect*
Heterogeneity judgment
No concerns

Comparison **Sucralose:Water**
Evidence: mixed
NMA estimate: 6.790
95% intervals for NMA estimate
Confidence interval: (-7.610,21.191)
Prediction interval: (-9.381,22.962)
*Confidence and prediction intervals **agree** in relation to clinically important effect*
Heterogeneity judgment
No concerns

Comparison **Sucrose:Water**
Evidence: mixed
NMA estimate: 112.248
95% intervals for NMA estimate
Confidence interval:
Prediction interval: (81.526,142.970)
Confidence and (77.748,146.748) *prediction intervals **agree** in relation to clinically important effect*
Heterogeneity judgment
No concerns

Comparison
Ace-K + Aspartame:Ace-K + Aspartame + Cyclamate
Evidence: indirect
NMA estimate: 5.660
95% intervals for NMA estimate
Confidence interval:
Prediction interval: (-43.448,54.769)
Confidence and (-49.487,60.808) *prediction intervals **agree** in relation to clinically important effect*
Heterogeneity judgment
No concerns

Comparison
Evidence: AceK:Ace-K + Aspartame indirect
NMA estimate: -56.642
95% intervals for NMA estimate
Confidence interval:
Prediction (-105.342,-7.942)
interval: (-111.330,-1.954)
*Confidence and prediction intervals **agree** in relation to clinically important effect*
Heterogeneity judgment
No concerns

Comparison
Ace-K + Aspartame:Aspartame
Evidence: indirect
NMA estimate: 54.611
95% intervals for NMA estimate
Confidence interval: (13.703,95.519)
Prediction interval: (8.672,100.549)
*Prediction interval **extends into clinically important or unimportant effects***
Heterogeneity judgment
Some concerns

Comparison
Ace-K + Aspartame:Cyclamate
Evidence: indirect
NMA estimate: 58.922
95% intervals for NMA estimate
Confidence interval:
Prediction interval: (10.126,107.718)
Confidence and (4.125,113.719) *prediction intervals **agree** in relation to clinically important effect*
Heterogeneity judgment
No concerns

Comparison
Ace-K + Aspartame:Fructose
Evidence: indirect
NMA estimate: 16.089
95% intervals for NMA estimate
Confidence interval:
Prediction interval: (-31.239,63.416)
Confidence and (-37.059,69.236) *prediction intervals **agree** in relation to clinically important effect*
Heterogeneity judgment
No concerns

Comparison
Ace-K + Aspartame:Glucose
Evidence: indirect
NMA estimate: -183.205
95% intervals for NMA estimate
Confidence interval:
Prediction (-251.404,-115.007)
interval: (-259.790,-106.620)
*Confidence and prediction intervals **agree** in relation to clinically important effect*
Heterogeneity judgment
No concerns

Comparison
Ace-K + Aspartame:Saccharin
Evidence: indirect
NMA estimate: 47.529
95% intervals for NMA estimate
Confidence interval: (1.808,93.249)
Prediction interval: (-3.814,98.872)
*Confidence and prediction intervals **agree** in relation to clinically important effect*
Heterogeneity judgment
No concerns

Comparison
Evidence: Ace-K + Aspartame:Stevia indirect
NMA estimate: 10.833
95% intervals for NMA estimate
Confidence interval:
Prediction interval: (-38.956,60.623)
Confidence and (-45.078,66.745) *prediction intervals **agree** in relation to clinically important effect*
Heterogeneity judgment
No concerns

Comparison
Ace-K + Aspartame:Sucralose
Evidence: indirect
NMA estimate: 57.291
95% intervals for NMA estimate
Confidence interval: (16.368,98.214)
Prediction interval: (11.336,103.246)
*Prediction interval **extends into clinically important or unimportant effects***
Heterogeneity judgment
Some concerns

(continued on next page)

Comparison
Evidence: Ace-K + Aspartame:Water indirect
NMA estimate: 64.081
95% intervals for NMA estimate
Confidence interval:
Prediction interval: (24.369,103.794)
Confidence and prediction intervals ***agree*** *in relation to clinically important effect* (19.485,108.677)
Heterogeneity judgment
No concerns

Comparison
AceK:Ace-K + Aspartame + Cyclamate
Evidence: indirect
NMA estimate: -50.982
95% intervals for NMA estimate
Confidence interval:
Prediction interval: (-110.284,8.321)
Confidence and prediction intervals ***agree*** *in relation to clinically important effect* (-117.577,15.614)
Heterogeneity judgment
No concerns

Comparison
Ace-K + Aspartame + Cyclamate:Aspartame
Evidence: indirect
NMA estimate: 48.950
95% intervals for NMA estimate
Confidence interval:
Prediction interval: (-4.141,102.041)
Confidence and prediction intervals ***agree*** *in relation to clinically important effect* (-10.669,108.569)
Heterogeneity judgment
No concerns

Comparison
Ace-K + Aspartame + Cyclamate:Cyclamate
Evidence: indirect
NMA estimate: 53.262
95% intervals for NMA estimate
Confidence interval:
Prediction interval: (-6.120,112.643)
Confidence and prediction intervals ***agree*** *in relation to clinically important effect* (-13.422,119.946)
Heterogeneity judgment
No concerns

Comparison
Ace-K + Aspartame + Cyclamate:Fructose
Evidence: indirect
NMA estimate: 10.428
95% intervals for NMA estimate
Confidence interval:
Prediction interval: (-47.753,68.609)
Confidence and prediction intervals ***agree*** *in relation to clinically important effect* (-54.907,75.764)
Heterogeneity judgment
No concerns

Comparison
Ace-K + Aspartame + Cyclamate:Glucose
Evidence: indirect
NMA estimate: -188.866
95% intervals for NMA estimate
Confidence interval:
Prediction interval: (-264.998,-112.733) (-274.361,-103.371)
Confidence and prediction intervals ***agree*** *in relation to clinically important effect*
Heterogeneity judgment
No concerns

Comparison
Ace-K + Aspartame + Cyclamate:Saccharin
Evidence: indirect
NMA estimate: 41.868
95% intervals for NMA estimate
Confidence interval:
Prediction interval: (-15.013,98.750)
Prediction interval ***extends into clinically important or unimportant effects*** (-22.008,105.745)
Heterogeneity judgment
Some concerns

Comparison
Ace-K + Aspartame + Cyclamate:Stevia
Evidence: indirect
NMA estimate: 5.173
95% intervals for NMA estimate
Confidence interval:
Prediction interval: (-55.028,65.373)
Confidence and prediction intervals ***agree*** *in relation to clinically important effect* (-62.431,72.776)
Heterogeneity judgment
No concerns

Comparison
Ace-K + Aspartame + Cyclamate:Sucralose
Evidence: indirect
NMA estimate: 51.630
95% intervals for NMA estimate
Confidence interval:
Prediction interval: (-1.472,104.733)
Confidence and prediction intervals ***agree*** *in relation to clinically important effect* (-8.002,111.263)
Heterogeneity judgment
No concerns

Comparison
Ace-K + Aspartame + Cyclamate:Water
Evidence: indirect
NMA estimate: 58.421
95% intervals for NMA estimate
Confidence interval: (6.246,110.596)
Prediction interval: (-0.170,117.012)
Prediction interval ***extends into clinically important or unimportant effects***
Heterogeneity judgment
Some concerns

Comparison AceK:Fructose
Evidence: indirect
NMA estimate: -40.553
95% intervals for NMA estimate
Confidence interval: (-82.931,1.824)
Prediction interval: (-88.142,7.035)
Confidence and prediction intervals ***agree*** *in relation to clinically important effect*
Heterogeneity judgment
No concerns

Comparison AceK:Glucose
Evidence: indirect
NMA estimate: -239.847
95% intervals for NMA estimate
Confidence interval:
Prediction interval: (-303.945,-175.749) (-311.828,-167.867)
Confidence and prediction intervals ***agree*** *in relation to clinically important effect*
Heterogeneity judgment
No concerns

Comparison AceK:Stevia
Evidence: indirect
NMA estimate: -45.809
95% intervals for NMA estimate
Confidence interval:
Prediction interval: (-105.676,14.059) (-113.038,21.421)
Confidence and prediction intervals ***agree*** *in relation to clinically important effect*
Heterogeneity judgment
No concerns

Comparison AceK:Sucralose
Evidence: indirect
NMA estimate: 0.649
95% intervals for NMA estimate
Confidence interval:
Prediction interval: (-34.325,35.623)
Confidence and prediction intervals ***agree*** *in relation to clinically important effect* (-38.626,39.924)
Heterogeneity judgment
No concerns

Comparison Aspartame:Fructose
Evidence: indirect
NMA estimate: -38.522
95% intervals for NMA estimate
Confidence interval: (-68.479,-8.565)
Prediction interval: (-72.162,-4.881)
Confidence and prediction intervals ***agree*** *in relation to clinically important effect*
Heterogeneity judgment
No concerns

Comparison Aspartame:Stevia
Evidence: indirect
NMA estimate: -43.777
95% intervals for NMA estimate
Confidence interval: (-97.498,9.944)
Prediction interval:
Prediction interval ***extends into clinically important or unimportant effects*** (-104.105,16.550)
Heterogeneity judgment
Some concerns

Comparison Cyclamate:Fructose
Evidence: indirect
NMA estimate: -42.833
95% intervals for NMA estimate
Confidence interval: (-85.321,-0.345)
Prediction interval: (-90.546,4.880)
Confidence and prediction intervals ***agree*** *in relation to clinically important effect*
Heterogeneity judgment
No concerns

Comparison Cyclamate:Glucose
Evidence: indirect
NMA estimate: -242.127
95% intervals for NMA estimate
Confidence interval:
Prediction interval: (-306.299,-177.956) (-314.190,-170.065)
Confidence and prediction intervals ***agree*** *in relation to clinically important effect*
Heterogeneity judgment
No concerns

Comparison Cyclamate:Stevia
Evidence: indirect
NMA estimate: -48.089
95% intervals for NMA estimate
Confidence interval:
Prediction interval: (-108.035,11.857) (-115.406,19.229)
Confidence and prediction intervals ***agree*** *in relation to clinically important effect*
Heterogeneity judgment
No concerns

Comparison Cyclamate:Sucralose
Evidence: indirect
NMA estimate: -1.631
95% intervals for NMA estimate
Confidence interval:
Prediction interval: (-36.739,33.477)
Confidence and prediction intervals ***agree*** *in relation to clinically important effect* (-41.056,37.794)
Heterogeneity judgment
No concerns

(continued on next page)

Comparison **Fructose:Saccharin**
Evidence: indirect
NMA estimate: **31.440**
95% intervals for NMA estimate
Confidence interval: **(-6.609,69.490)**
Prediction interval: **(-11.289,74.169)**
*Confidence and prediction intervals **agree** in relation to clinically important effect*
Heterogeneity judgment
No concerns

Comparison **Fructose:Stevia**
Evidence: indirect
NMA estimate: **-5.255**
95% intervals for NMA estimate
Confidence interval:
Prediction interval: **(-64.012,53.502)**
(-71.238,60.727)
*Confidence and prediction intervals **agree** in relation to clinically important effect*
Heterogeneity judgment
No concerns

Comparison **Fructose:Water**
Evidence: indirect
NMA estimate: **47.993**
95% intervals for NMA estimate
Confidence interval: **(20.043,75.943)**
Prediction interval: **(16.606,79.380)**
*Confidence and prediction intervals **agree** in relation to clinically important effect*
Heterogeneity judgment
No concerns

Comparison **Glucose:Saccharin**
Evidence: indirect
NMA estimate: **230.734**
95% intervals for NMA estimate
Confidence interval:
Prediction interval: **(169.572,291.896)**
(162.051,299.417)
*Confidence and prediction intervals **agree** in relation to clinically important effect*
Heterogeneity judgment
No concerns

Comparison **Glucose:Stevia**
Evidence: indirect
NMA estimate: **194.038**
95% intervals for NMA estimate
Confidence interval:
Prediction interval: **(117.465,270.612)**
(108.048,280.029)
*Confidence and prediction intervals **agree** in relation to clinically important effect*
Heterogeneity judgment
No concerns

Comparison **Glucose:Water**
Evidence: indirect
NMA estimate: **247.287**
95% intervals for NMA estimate
Confidence interval:
Prediction interval: **(191.300,303.273)**
(184.415,310.158)
*Confidence and prediction intervals **agree** in relation to clinically important effect*
Heterogeneity judgment
No concerns

Comparison **Saccharin:Stevia**
Evidence: indirect
NMA estimate: **-36.696**
95% intervals for NMA estimate
Confidence interval:
Prediction interval: **(-94.166,20.775)**
(-101.233,27.842)
*Prediction interval **extends into clinically important or unimportant effects***
Heterogeneity judgment
Some concerns

Comparison **Saccharin:Sucralose**
Evidence: indirect
NMA estimate: **9.762**
95% intervals for NMA estimate
Confidence interval:
Prediction interval: **(-19.815,39.340)**
(-23.452,42.977)
*Confidence and prediction intervals **agree** in relation to clinically important effect*
Heterogeneity judgment
No concerns

Comparison **Stevia:Sucralose**
Evidence: indirect
NMA estimate: **46.458**
95% intervals for NMA estimate
Confidence interval:
Prediction interval: **(-7.275,100.190)**
(-13.882,106.798)
*Confidence and prediction intervals **agree** in relation to clinically important effect*
Heterogeneity judgment
No concerns

Comparison **Stevia:Water**
Evidence: indirect
NMA estimate: **53.248**
95% intervals for NMA estimate
Confidence interval: **(0.432,106.064)**
Prediction interval: **(-6.063,112.559)**
*Prediction interval **extends into clinically important or unimportant effects***
Heterogeneity judgment
Some concerns

Judgements for were assigned by the CiNEMA algorithm (27, 28).

Figure S9. *CINeMA output for the INCOHERENCE domain of UNCOUPLING INTERVENTIONS examining non-nutritive sweetened beverages (NNS beverages) sweetened with individual or blends of non-nutritive sweeteners (NNS), water, and sugar-sweetened beverages (SSBs) sweetened with caloric sweeteners on postprandial blood GLUCOSE response in HEALTHY participants*

Incoherence

Define clinically important size of effect: Mean difference 100

*Relative effect estimates below **-100.000** and above **100.000** are considered clinically important.*
Importance of Incoherence depends on the variability of direct and indirect effects in relation to a clinically important size of effect

Global test based on a random-effects design-by-treatment interaction model
χ^2 statistic: 9.240 (8 degrees of freedom), P value: 0.323

Local tests: Separating indirect from direct evidence

Comparison **Ace-K + Aspartame:Sucrose**
Evidence: direct
Direct mean difference: **-48.167(-73.331,-23.002)**
Inconsistency measures: Not applicable
Incoherence judgment: No concerns

Comparison **Ace-K + Aspartame + Cyclamate:Sucrose**
Evidence: direct
Direct mean difference: **-53.827(-95.998,-11.656)**
Inconsistency measures: Not applicable
Incoherence judgment: No concerns

Comparison **AceK:Aspartame**
Evidence: mixed
NMA mean difference: **-2.031(-34.988,30.925)**
Direct mean difference: **-1.330(-49.024,46.364)**
Indirect mean difference: **-2.672(-48.264,42.919)**
Inconsistency measures
Difference of mean differences: **1.343(-64.637,67.322)**
P value: **0.968**
Incoherence judgment: No concerns

Comparison **AceK:Cyclamate**
Evidence: direct
Direct mean difference: **2.280(-35.056,39.616)**
Inconsistency measures: Not applicable
Incoherence judgment: No concerns

Comparison **AceK:Saccharin**
Evidence: mixed
NMA mean difference: **-9.113(-44.285,26.058)**
Direct mean difference: **-14.420(-52.401,23.561)**
Indirect mean difference: **22.828(-70.353,116.008)**
Inconsistency measures
Difference of mean differences: **-37.248(-137.871,63.376)**
P value: **0.468**
Incoherence judgment: No concerns

Comparison **AceK:Sucrose**
Evidence: mixed
NMA mean difference: **-104.809(-146.503,-63.114)**
Direct mean difference: **-60.610(-118.001,-3.219)**
Indirect mean difference: **-154.209(-214.884,-93.535)**
Inconsistency measures
Difference of mean differences: **93.599(10.082,177.117)**
P value: **0.028**
Incoherence judgment: No concerns

Comparison **AceK:Water**
Evidence: mixed
NMA mean difference: **7.439(-24.701,39.580)**
Direct mean difference: **1.440(-34.217,37.097)**
Indirect mean difference: **33.435(-40.788,107.657)**
Inconsistency measures
Difference of mean differences: **-31.995(-114.338,50.348)**
P value: **0.446**
Incoherence judgment: No concerns

Comparison **Aspartame:Cyclamate**
Evidence: mixed
NMA mean difference: **4.311(-28.787,37.410)**
Direct mean difference: **3.610(-44.182,51.402)**
Indirect mean difference: **4.958(-40.925,50.841)**
Inconsistency measures
Difference of mean differences: **-1.348(-67.600,64.904)**
P value: **0.968**
Incoherence judgment: No concerns

Comparison **Aspartame:Glucose**
Evidence: mixed
NMA mean difference: **-237.816(-293.324,-182.307)**
Direct mean difference: **-264.561(-331.172,-197.950)**
Indirect mean difference: **-177.035(-277.452,-76.618)**
Inconsistency measures
Difference of mean differences: **-87.526(-208.028,32.975)**
P value: **0.155**
Incoherence judgment: No concerns

Comparison **Aspartame:Saccharin**
Evidence: mixed
NMA mean difference: **-7.082(-33.714,19.550)**
Direct mean difference: **-5.713(-38.059,26.633)**
Indirect mean difference: **-9.962(-56.888,36.964)**
Inconsistency measures
Difference of mean differences: **4.249(-52.745,61.243)**
P value: **0.884**
Incoherence judgment: No concerns

Comparison **Aspartame:Sucralose**
Evidence: mixed
NMA mean difference: **2.680(-15.319,20.680)**
Direct mean difference: **3.298(-16.793,23.388)**
Indirect mean difference: **0.169(-40.355,40.692)**
Inconsistency measures
Difference of mean differences: **3.129(-42.101,48.359)**
P value: **0.892**
Incoherence judgment: No concerns

Comparison **Aspartame:Sucrose**
Evidence: mixed
NMA mean difference: **-102.777(-135.030,-70.525)**
Direct mean difference: **-112.811(-151.223,-74.399)**
Indirect mean difference: **-78.798(-138.180,-19.416)**
Inconsistency measures
Difference of mean differences: **-34.013(-104.736,36.710)**
P value: **0.346**
Incoherence judgment: No concerns

Comparison **Aspartame:Water**
Evidence: mixed
NMA mean difference: **9.471(-1.953,20.895)**
Direct mean difference: **9.451(-2.066,20.969)**
Indirect mean difference: **10.659(-79.202,100.520)**
Inconsistency measures
Difference of mean differences: **-1.207(-91.803,89.388)**
P value: **0.979**
Incoherence judgment: No concerns

Comparison **Cyclamate:Saccharin**
Evidence: mixed
NMA mean difference: **-11.393(-46.698,23.912)**
Direct mean difference: **-16.700(-54.804,21.404)**
Indirect mean difference: **20.790(-73.048,114.628)**
Inconsistency measures
Difference of mean differences: **-37.490(-138.770,63.789)**
P value: **0.468**
Incoherence judgment: No concerns

Comparison **Cyclamate:Sucrose**
Evidence: mixed
NMA mean difference: **-107.089(-148.896,-65.282)**
Direct mean difference: **-62.890(-120.363,-5.417)**
Indirect mean difference: **-156.756(-217.682,-95.831)**
Inconsistency measures
Difference of mean differences: **93.867(10.111,177.623)**
P value: **0.028**
Incoherence judgment: No concerns

Comparison **Cyclamate:Water**
Evidence: mixed
NMA mean difference: **5.159(-27.127,37.446)**
Direct mean difference: **-0.840(-36.628,34.948)**
Indirect mean difference: **31.391(-43.443,106.226)**
Inconsistency measures
Difference of mean differences: **-32.231(-115.183,50.721)**
P value: **0.446**
Incoherence judgment: No concerns

Comparison **Fructose:Glucose**
Evidence: mixed
NMA mean difference: **-199.294(-260.661,-137.927)**
Direct mean difference: **-140.611(-242.066,-39.155)**
Indirect mean difference: **-233.151(-310.214,-156.088)**
Inconsistency measures
Difference of mean differences: **92.540(-34.864,219.945)**
P value: **0.155**
Incoherence judgment: No concerns

Comparison **Fructose:Sucralose**
Evidence: direct
Direct mean difference: **41.059(17.061,65.057)**
Inconsistency measures: Not applicable
Incoherence judgment: No concerns

Comparison **Fructose:Sucrose**
Evidence: mixed
NMA mean difference: **-64.255(-104.338,-24.172)**
Direct mean difference: **-57.407(-122.564,7.751)**
Indirect mean difference: **-68.425(-119.266,-17.584)**
Inconsistency measures
Difference of mean differences: **11.018(-71.628,93.664)**
P value: **0.794**
Incoherence judgment: No concerns

Comparison **Glucose:Sucralose**
Evidence: mixed
NMA mean difference: **240.496(183.900,297.092)**
Direct mean difference: **181.669(82.908,280.430)**
Indirect mean difference: **269.261(200.201,338.321)**
Inconsistency measures
Difference of mean differences: **-87.591(-208.103,32.920)**
P value: **0.154**
Incoherence judgment: No concerns

Comparison **Glucose:Sucrose**
Evidence: mixed
NMA mean difference: **135.038(71.652,198.425)**
Direct mean difference: **83.204(-32.655,199.063)**
Indirect mean difference: **157.181(81.457,232.905)**
Inconsistency measures
Difference of mean differences: **-73.977(-212.388,64.433)**
P value: **0.295**
Incoherence judgment: No concerns

Comparison Saccharin:Sucrose
Evidence: mixed
NMA mean difference: -95.695(-133.868,-57.523)
Direct mean difference: -46.190(-104.084,11.704)
Indirect mean difference: -133.769(-184.541,-82.998)
Inconsistency measures
Difference of mean differences: 87.579(10.576,164.583)
P value: 0.026
Incoherence judgment: No concerns

Comparison Saccharin:Water
Evidence: mixed
NMA mean difference: 16.553(-9.559,42.664)
Direct mean difference: 9.847(-19.181,38.875)
Indirect mean difference: 44.982(-14.788,104.753)
Inconsistency measures
Difference of mean differences: -35.135(-101.582,31.311)
P value: 0.300
Incoherence judgment: No concerns

Comparison Stevia:Sucrose
Evidence: direct
Direct mean difference: -59.000(-101.962,-16.038)
Inconsistency measures: Not applicable
Incoherence judgment: No concerns

Comparison Sucralose:Sucrose
Evidence: mixed
NMA mean difference: -105.458(-137.729,-73.186)
Direct mean difference: -127.048(-165.034,-89.062)
Indirect mean difference: -49.458(-110.635,11.720)
Inconsistency measures
Difference of mean differences: -77.590(-149.602,-5.579)
P value: 0.035
Incoherence judgment: No concerns

Comparison Sucralose:Water
Evidence: mixed
NMA mean difference: 6.790(-7.610,21.191)
Direct mean difference: 5.194(-9.615,20.002)
Indirect mean difference: 34.584(-27.202,96.369)
Inconsistency measures
Difference of mean differences: -29.390(-92.925,34.145)
P value: 0.365
Incoherence judgment: No concerns

Comparison Sucrose:Water
Evidence: mixed
NMA mean difference: 112.248(81.526,142.970)
Direct mean difference: 116.172(80.477,151.868)
Indirect mean difference: 101.035(40.699,161.372)
Inconsistency measures
Difference of mean differences: 15.137(-54.968,85.242)
P value: 0.672
Incoherence judgment: No concerns

Comparison Ace-K + Aspartame:Ace-K + Aspartame + Cyclamate
Evidence: indirect
Indirect mean difference: 5.660(-43.448,54.769)
Inconsistency measures: Not applicable
Incoherence judgment: No concerns

Comparison AceK:Ace-K + Aspartame
Evidence: indirect
Indirect mean difference: -56.642(-105.342,-7.942)
Inconsistency measures: Not applicable
Incoherence judgment: No concerns

Comparison Ace-K + Aspartame:Aspartame
Evidence: indirect
Indirect mean difference: 54.611(13.703,95.519)
Inconsistency measures: Not applicable
Incoherence judgment: No concerns

Comparison Ace-K + Aspartame:Cyclamate
Evidence: indirect
Indirect mean difference: 58.922(10.126,107.718)
Inconsistency measures: Not applicable
Incoherence judgment: No concerns

Comparison Ace-K + Aspartame:Fructose
Evidence: indirect
Indirect mean difference: 16.089(-31.239,63.416)
Inconsistency measures: Not applicable
Incoherence judgment: No concerns

Comparison Ace-K + Aspartame:Glucose
Evidence: indirect
Indirect mean difference: -183.205(-251.404,-115.007)
Inconsistency measures: Not applicable
Incoherence judgment: No concerns

Comparison Ace-K + Aspartame:Saccharin
Evidence: indirect
Indirect mean difference: 47.529(1.808,93.249)
Inconsistency measures: Not applicable
Incoherence judgment: No concerns

Comparison Ace-K + Aspartame:Stevia
Evidence: indirect
Indirect mean difference: 10.833(-38.956,60.623)
Inconsistency measures: Not applicable
Incoherence judgment: No concerns

Comparison Ace-K + Aspartame:Sucralose
Evidence: indirect
Indirect mean difference: 57.291(16.368,98.214)
Inconsistency measures: Not applicable
Incoherence judgment: No concerns

Comparison Ace-K + Aspartame:Water
Evidence: indirect
Indirect mean difference: 64.081(24.369,103.794)
Inconsistency measures: Not applicable
Incoherence judgment: No concerns

Comparison AceK:Ace-K + Aspartame + Cyclamate
Evidence: indirect
Indirect mean difference: -50.982(-110.284,8.321)
Inconsistency measures: Not applicable
Incoherence judgment: No concerns

Comparison Ace-K + Aspartame + Cyclamate:Aspartame
Evidence: indirect
Indirect mean difference: 48.950(-4.141,102.041)
Inconsistency measures: Not applicable
Incoherence judgment: No concerns

Comparison Ace-K + Aspartame + Cyclamate:Cyclamate
Evidence: indirect
Indirect mean difference: 53.262(-6.120,112.643)
Inconsistency measures: Not applicable
Incoherence judgment: No concerns

Comparison Ace-K + Aspartame + Cyclamate:Fructose
Evidence: indirect
Indirect mean difference: 10.428(-47.753,68.609)
Inconsistency measures: Not applicable
Incoherence judgment: No concerns

Comparison Ace-K + Aspartame + Cyclamate:Glucose
Evidence: indirect
Indirect mean difference: -188.866(-264.998,-112.733)
Inconsistency measures: Not applicable
Incoherence judgment: No concerns

Comparison Ace-K + Aspartame + Cyclamate:Saccharin
Evidence: indirect
Indirect mean difference: 41.868(-15.013,98.750)
Inconsistency measures: Not applicable
Incoherence judgment: No concerns

Comparison Ace-K + Aspartame + Cyclamate:Stevia
Evidence: indirect
Indirect mean difference: 5.173(-55.028,65.373)
Inconsistency measures: Not applicable
Incoherence judgment: No concerns

Comparison Ace-K + Aspartame + Cyclamate:Sucralose
Evidence: indirect
Indirect mean difference: 51.630(-1.472,104.733)
Inconsistency measures: Not applicable
Incoherence judgment: No concerns

Comparison Ace-K + Aspartame + Cyclamate:Water
Evidence: indirect
Indirect mean difference: 58.421(6.246,110.596)
Inconsistency measures: Not applicable
Incoherence judgment: No concerns

Comparison AceK:Fructose
Evidence: indirect
Indirect mean difference: -40.553(-82.931,1.824)
Inconsistency measures: Not applicable
Incoherence judgment: No concerns

Comparison AceK:Glucose
Evidence: indirect
Indirect mean difference: -239.847(-303.945,-175.749)
Inconsistency measures: Not applicable
Incoherence judgment: No concerns

Comparison AceK:Stevia
Evidence: indirect
Indirect mean difference: -45.809(-105.676,14.059)
Inconsistency measures: Not applicable
Incoherence judgment: No concerns

Comparison AceK:Sucralose
Evidence: indirect
Indirect mean difference: 0.649(-34.325,35.623)
Inconsistency measures: Not applicable
Incoherence judgment: No concerns

Comparison Aspartame:Fructose
Evidence: indirect
Indirect mean difference: -38.522(-68.479,-8.565)
Inconsistency measures: Not applicable
Incoherence judgment: No concerns

Comparison Aspartame:Stevia
Evidence: indirect
Indirect mean difference: -43.777(-97.498,9.944)
Inconsistency measures: Not applicable
Incoherence judgment: No concerns

Comparison Cyclamate:Fructose
Evidence: indirect
Indirect mean difference: -42.833(-85.321,-0.345)
Inconsistency measures: Not applicable
Incoherence judgment: No concerns

Comparison Cyclamate:Glucose
Evidence: indirect
Indirect mean difference: -242.127(-306.299,-177.956)
Inconsistency measures: Not applicable
Incoherence judgment: No concerns

Comparison Cyclamate:Stevia
Evidence: indirect
Indirect mean difference: -48.089(-108.035,11.857)
Inconsistency measures: Not applicable
Incoherence judgment: No concerns

Comparison Cyclamate:Sucralose
Evidence: indirect
Indirect mean difference: -1.631(-36.739,33.477)
Inconsistency measures: Not applicable
Incoherence judgment: No concerns

Comparison Fructose:Saccharin
Evidence: indirect
Indirect mean difference: 31.440(-6.609,69.490)
Inconsistency measures: Not applicable
Incoherence judgment: No concerns

Comparison Fructose:Stevia
Evidence: indirect
Indirect mean difference: -5.255(-64.012,53.502)
Inconsistency measures: Not applicable
Incoherence judgment: No concerns

Comparison Fructose:Water
Evidence: indirect
Indirect mean difference: 47.993(20.043,75.943)
Inconsistency measures: Not applicable
Incoherence judgment: No concerns

Comparison Glucose:Saccharin
Evidence: indirect
Indirect mean difference: 230.734(169.572,291.896)
Inconsistency measures: Not applicable
Incoherence judgment: No concerns

Comparison Glucose:Stevia
Evidence: indirect
Indirect mean difference: 194.038(117.465,270.612)
Inconsistency measures: Not applicable
Incoherence judgment: No concerns

Comparison Glucose:Water
Evidence: indirect
Indirect mean difference: 247.287(191.300,303.273)
Inconsistency measures: Not applicable
Incoherence judgment: No concerns

Comparison Saccharin:Stevia
Evidence: indirect
Indirect mean difference: -36.696(-94.166,20.775)
Inconsistency measures: Not applicable
Incoherence judgment: No concerns

Comparison Saccharin:Sucralose
Evidence: indirect
Indirect mean difference: 9.762(-19.815,39.340)
Inconsistency measures: Not applicable
Incoherence judgment: No concerns

Comparison Stevia:Sucralose
Evidence: indirect
Indirect mean difference: 46.458(-7.275,100.190)
Inconsistency measures: Not applicable
Incoherence judgment: No concerns

Comparison Stevia:Water
Evidence: indirect
Indirect mean difference: 53.248(0.432,106.064)
Inconsistency measures: Not applicable
Incoherence judgment: No concerns

Figure S10. *Risk of bias assessment of UNCOUPLING INTERVENTIONS examining non-nutritive sweetened beverages (NNS beverages) sweetened with individual or blends of non-nutritive sweeteners (NNS), water, and sugar-sweetened beverages (SSBs) sweetened with caloric sweeteners on postprandial blood GLUCOSE response in participants with TYPE 2 DIABETES*

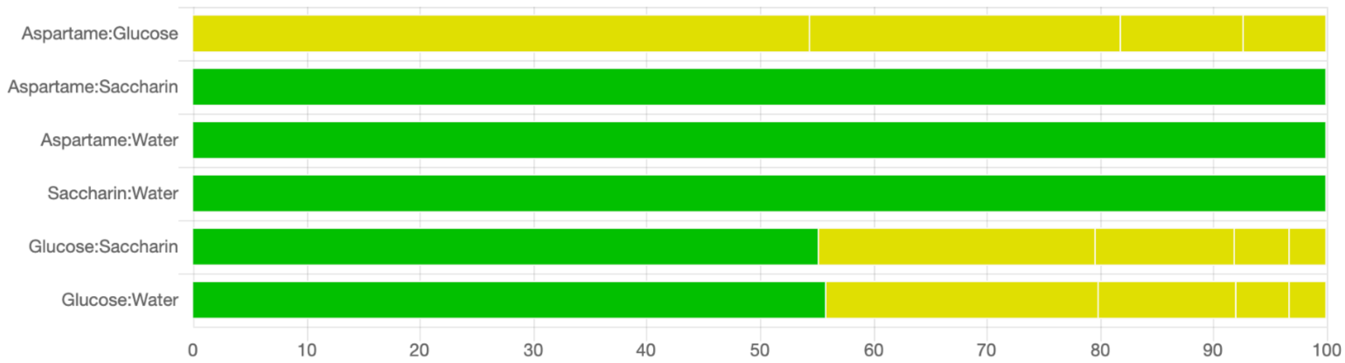

Green = no concerns; yellow = some concerns; red = major concerns

Figure S11. *CINeMA output for the IMPRECISION domain of UNCOUPLING INTERVENTIONS examining non-nutritive sweetened beverages (NNS beverages) sweetened with individual or blends of non-nutritive sweeteners (NNS), water, and sugar-sweetened beverages (SSBs) sweetened with caloric sweeteners on postprandial blood GLUCOSE response in participants with TYPE 2 DIABETES*

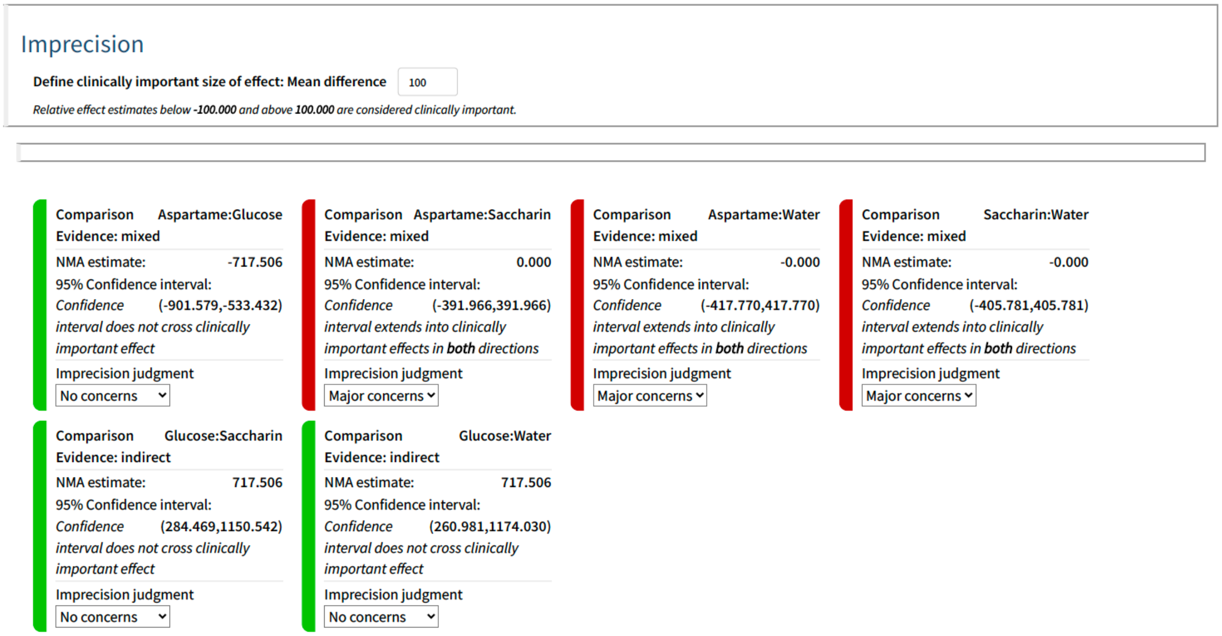

Relative effect estimates below -100.0 and above 100.0 mmol*min/L (minimal important difference; MID) were considered clinically important. Judgements for IMPRECISION were assigned by the CiNEMA algorithm (27, 28). There is no general consensus on a clinically important size difference for 2-hour blood glucose incremental area under the curve (iAUC). Health Canada considers a 20% reduction in glucose iAUC to be the minimum physiologically relevant difference as per "Guideline Concerning the Safety and Physiological Effects of Novel Fibre Sources and Food Products Containing Them" (29). Thus, we calculated the MID of glucose iAUC by taking 20% of 500 mmol*mins/L which is the median of glucose iAUC in healthy participants (~300 mmol*mins/L) and those with type 2 diabetes (~700 mmol*mins/L) after a 75-g oral glucose tolerance test (32-34).

Figure S12. *CINeMA output for the HETEROGENEITY domain of UNCOUPLING INTERVENTIONS examining non-nutritive sweetened beverages (NNS beverages) sweetened with individual or blends of non-nutritive sweeteners (NNS), water, and sugar-sweetened beverages (SSBs) sweetened with caloric sweeteners on postprandial blood GLUCOSE response in participants with TYPE 2 DIABETES*

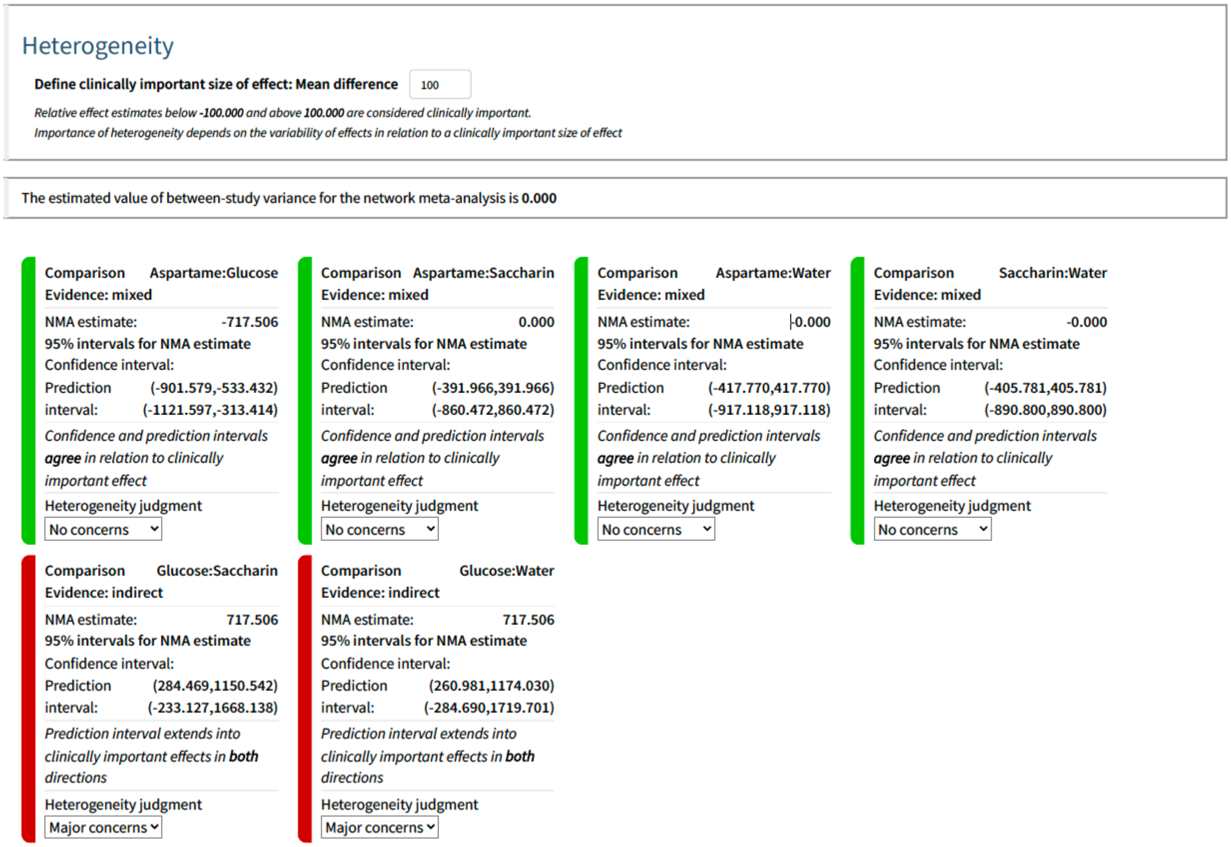

Judgements for were assigned by the CiNEMA algorithm (27, 28).

Figure S13. *CINeMA output for the INCOHERENCE domain of UNCOUPLING INTERVENTIONS examining non-nutritive sweetened beverages (NNS beverages) sweetened with individual or blends of non-nutritive sweeteners (NNS), water, and sugar-sweetened beverages (SSBs) sweetened with caloric sweeteners on postprandial blood GLUCOSE response in participants with TYPE 2 DIABETES*

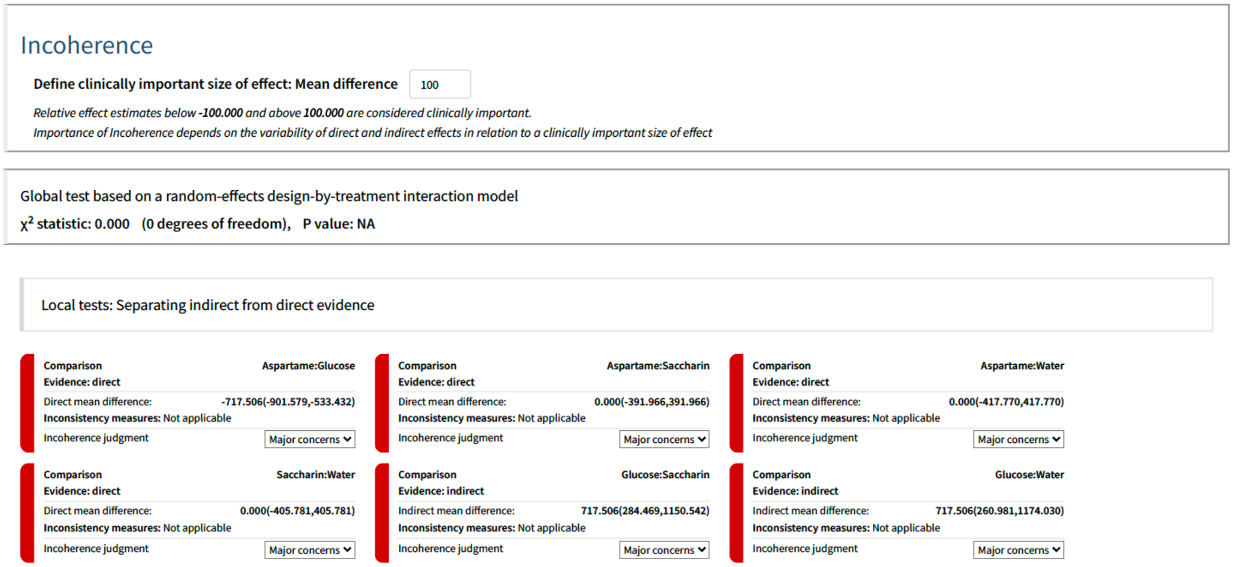

Judgements for were assigned by the CiNEMA algorithm (27, 28).

Figure S14. *Network plot and meta-analysis of UNCOUPLING INTERVENTIONS evaluating the effect of non-nutritive sweetened beverages (NNS beverages) sweetened single or blends of non-nutritive sweeteners (NNS), water, and sugar-sweetened beverages (SSBs) sweetened with caloric sweeteners on postprandial blood INSULIN response in HEALTHY participants*

Network Plot

13 Trials | 134 Participants (Healthy)

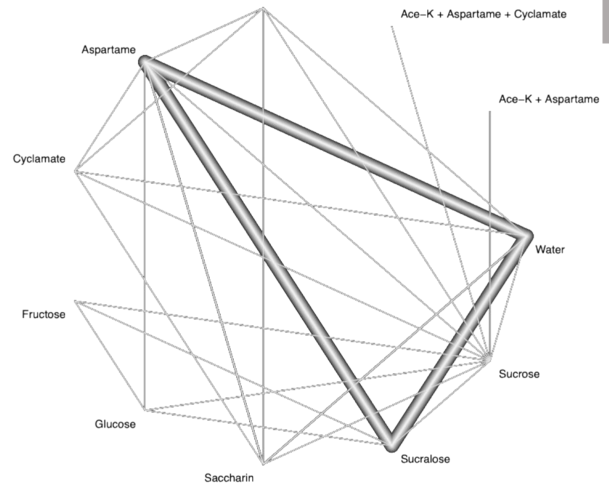

	Individual NNS					NNS Blends		Water	Caloric Sweeteners		
	Ace-K	Aspartame	Cyclamate	Saccharin	Sucralose	Ace-K + Aspartame	Ace-K + Aspartame + Cyclamate	Water	Glucose	Sucrose	Fructose
**Ace-K (1 trial, N=14)**	**Ace-K (1 trial, N=14)**	152.138 (-1134.250, 1438.527) ⊕⊕	413.940 (-1250.544, 2078.424) ⊕⊕	72.681 (-1306.769, 1452.132) ⊕⊕	212.007 (-1086.771, 1510.784) ⊕⊕	-2710.118 (-6756.762, 1336.526) ⊕⊕	-387.005 (-3180.590, 2406.579) ⊕⊕⊕	139.946 (-1149.829, 1429.722) ⊕⊕	**16414.167 (13339.187, 19489.147) ⊕⊕⊕⊕**	**7501.882 (5440.923, 9562.841) ⊕⊕⊕**	**4425.591 (1441.605, 7409.577) ⊕⊕⊕⊕**
**Aspartame (8 trials, N=82)**		**Aspartame (8 trials, N=82)**	261.802 (-1235.685, 1759.288) ⊕⊕	-79.457 (-1105.794, 946.881) ⊕⊕⊕	59.868 (-186.872, 306.609) ⊕⊕⊕⊕	-2862.256 (-6814.142, 1089.629) ⊕⊕	-539.144 (-3193.609, 2115.322) ⊕⊕	-12.192 (-259.197, 234.812) ⊕⊕⊕⊕	**16262.029 (13463.554, 19060.503) ⊕⊕⊕⊕**	**7349.743 (5481.690, 9217.797) ⊕⊕⊕**	**4273.453 (1572.057, 6974.848) ⊕⊕⊕⊕**
**Cyclamate (1 trial, N=14)**			**Cyclamate (1 trial, N=14)**	-341.259 (-1919.408, 1236.891) ⊕⊕	-201.934 (-1710.076, 1306.209) ⊕⊕	-800.945 (-3697.803, 2095.912) ⊕⊕	-800.945 (-3697.803, 2095.912) ⊕⊕	-273.994 (-1774.391, 1226.403) ⊕⊕	**16000.227 (12831.130, 19169.323) ⊕⊕⊕⊕**	**7087.942 (4889.028, 9286.855) ⊕⊕⊕**	**4011.651 (930.768, 7092.534) ⊕⊕⊕**
**Saccharin (2 trials, N=24)**				**Saccharin (2 trials, N=24)**	139.325 (-902.552, 1181.202) ⊕⊕	-459.687 (-3190.421, 2271.048) ⊕⊕	-459.687 (-3190.421, 2271.048) ⊕⊕	67.265 (-962.883, 1097.412) ⊕⊕⊕	**16341.486 (13365.273, 19317.698) ⊕⊕⊕⊕**	**7429.200 (5454.270, 9404.130) ⊕⊕⊕**	**4352.909 (1470.438, 7235.381) ⊕⊕⊕⊕**
**Sucralose (3 trials, N=29)**					**Sucralose (3 trials, N=29)**	-2922.125 (-6876.217, 1031.967) ⊕⊕	-599.012 (-3256.761, 2058.738) ⊕⊕	-72.060 (-315.199, 171.078) ⊕⊕⊕⊕	**16202.160 (13406.276, 18998.044) ⊕⊕⊕⊕**	**7289.875 (5417.159, 9162.592) ⊕⊕⊕⊕**	**4213.584 (1522.787, 6904.382) ⊕⊕⊕⊕**
**Ace-K + Aspartame (1 trial, N=9)**						**Ace-K + Aspartame (1 trial, N=9)**	2323.113 (-1637.233, 6283.459) ⊕⊕	2850.064 (-1102.684, 6802.813) ⊕⊕	**19124.285 (14295.133, 23953.437) ⊕⊕⊕⊕**	**10212.000 (6729.504, 13694.496) ⊕⊕⊕⊕**	**7135.709 (2369.765, 11901.654) ⊕⊕⊕⊕**
**Ace-K + Aspartame + Cyclamate (2 trials, N=20)**							**Ace-K + Aspartame + Cyclamate (2 trials, N=20)**	526.951 (-2128.798, 3182.701) ⊕⊕	**16801.172 (12960.665, 20641.679) ⊕⊕⊕⊕**	**7888.887 (6003.001, 9774.773) ⊕⊕⊕⊕**	**4812.596 (1051.876, 8573.316) ⊕⊕⊕**
**Water (5 trials, N=44)**								**Water (5 trials, N=44)**	**16274.221 (13471.874, 19076.568) ⊕⊕⊕⊕**	**7361.936 (5492.058, 9231.813) ⊕⊕⊕**	**4285.645 (1584.354, 6986.936) ⊕⊕⊕⊕**
**Glucose (5 trials, N=61)**									**Glucose (5 trials, N=61)**	**-8912.285 (-12257.869, -5566.701) ⊕⊕⊕**	**-11988.576 (-15780.748, -8196.404) ⊕⊕⊕⊕**
**Sucrose (6 trials, N=64)**										**Sucrose (6 trials, N=64)**	-3076.291 (-6329.973, 177.391) ⊕⊕⊕
**Fructose (1 trial, N=15)**											**Fructose (1 trial, N=15)**

Network Plot & Meta-Analysis (13 trials, N=134, Healthy Participants)

Network plot: the size of the blue nodes is proportional to the number of participants and the line width is proportional to the number of studies. Network table: treatments are grouped by treatment type (i.e., individual non-nutritive sweeteners (NNS), NNS blends, water and caloric sweeteners) and are reported in alphabetical order. Treatment estimates (pmol*min/L) are MDs and 95% CIs of the column-defining treatment compared with the row-defining treatment. MDs less than 0 favor the column-defining treatment. MDs greater than 0 favor the row-defining treatment. Significant results are bolded in black. Non-significant results are grey and not bolded. The minimally important difference (MID) for postprandial insulin response is 2250 pmol*min/L. Trivial (significant) effects (<1 MID) or no effects have a white background; small important effects (≥1 MID) have a light blue background; moderate effects (≥2 MID) have a darker blue background; large effects (≥5 to <10 MID) have a purple background; very large effects

(≥10 MID) have a black background. Confidence in the effect estimate is shown for each treatment comparison: high confidence ⨁⨁⨁⨁; moderate confidence ⨁⨁⨁; low confidence ⨁⨁; very low confidence ⨁. See **Supplementary Table 8 and Figures 15-18** for detailed assessments of the confidence in the effect estimate using the CINeMA framework.

Figure S15. *Risk of bias assessment of UNCOUPLING INTERVENTIONS evaluating the effect of non-nutritive sweetened beverages (NNS beverages) sweetened single or blends of non-nutritive sweeteners (NNS), water, and sugar-sweetened beverages (SSBs) sweetened with caloric sweeteners on postprandial blood INSULIN response in HEALTHY participants*

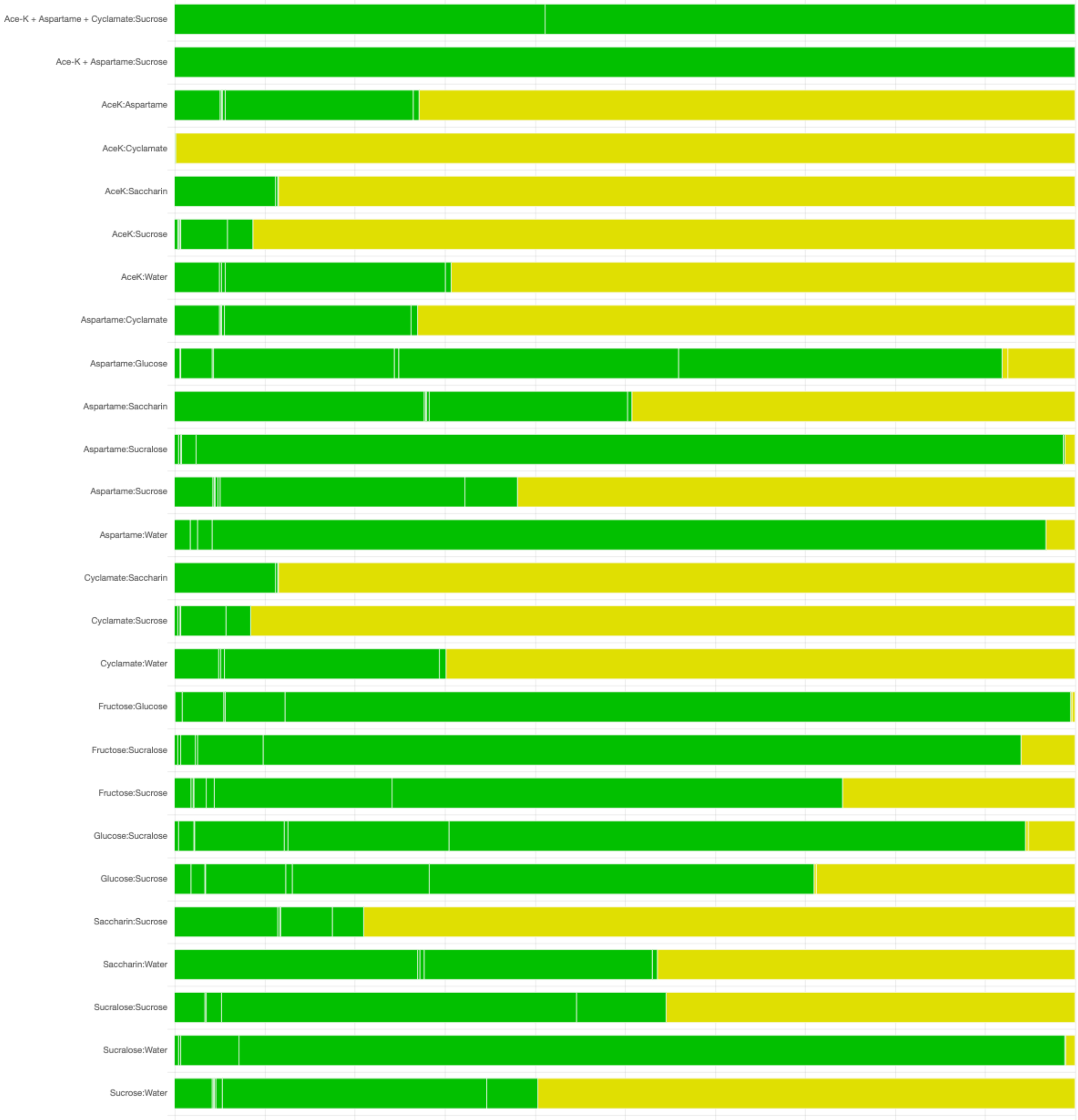

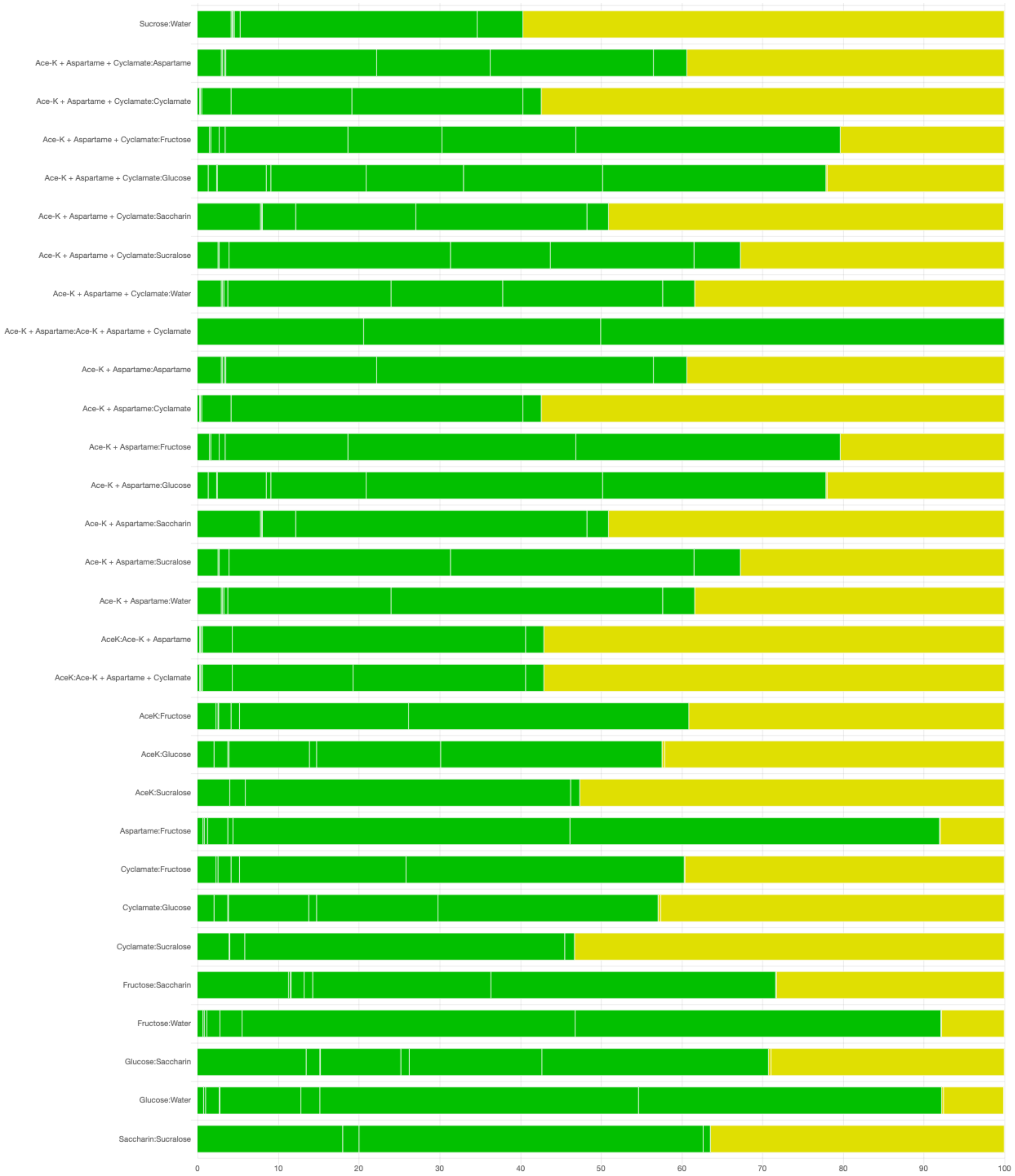

Green = no concerns; yellow = some concerns; red = major concerns

Figure S16. *CINeMA output for the IMPRECISION domain of UNCOUPLING INTERVENTIONS evaluating the effect of non-nutritive sweetened beverages (NNS beverages) sweetened single or blends of non-nutritive sweeteners (NNS), water, and sugar-sweetened beverages (SSBs) sweetened with caloric sweeteners on postprandial blood INSULIN response in HEALTHY participants*

Imprecision

Define clinically important size of effect: Mean difference 2250

*Relative effect estimates below **-2250.000** and above **2250.000** are considered clinically important.*

Comparison	Evidence	NMA estimate	95% Confidence interval	Confidence interval	Imprecision judgment
Ace-K + Aspartame:Sucrose	mixed	-10212.000	(-13694.496,-6729.504)	*Confidence interval does not cross clinically important effect*	No concerns
Ace-K + Aspartame + Cyclamate:Sucrose	mixed	-7888.887	(-9774.773,-6003.001)	*Confidence interval does not cross clinically important effect*	No concerns
AceK:Aspartame	mixed	-152.138	(-1438.527,1134.250)	*Confidence interval does not cross clinically important effect*	No concerns
AceK:Cyclamate	mixed	-413.940	(-2078.424,1250.544)	*Confidence interval does not cross clinically important effect*	No concerns
AceK:Saccharin	mixed	-72.681	(-1452.132,1306.769)	*Confidence interval does not cross clinically important effect*	No concerns
AceK:Sucrose	mixed	-7501.882	(-9562.841,-5440.923)	*Confidence interval does not cross clinically important effect*	No concerns
AceK:Water	mixed	-139.946	(-1429.722,1149.829)	*Confidence interval does not cross clinically important effect*	No concerns
Aspartame:Cyclamate	mixed	-261.802	(-1759.288,1235.685)	*Confidence interval does not cross clinically important effect*	No concerns
Aspartame:Glucose	mixed	-16262.029	(-19060.503,-13463.554)	*Confidence interval does not cross clinically important effect*	No concerns
Aspartame:Saccharin	mixed	79.457	(-946.881,1105.794)	*Confidence interval does not cross clinically important effect*	No concerns
Aspartame:Sucralose	mixed	-59.868	(-306.609,186.872)	*Confidence interval does not cross clinically important effect*	No concerns
Aspartame:Sucrose	mixed	-7349.743	(-9217.797,-5481.690)	*Confidence interval does not cross clinically important effect*	No concerns
Aspartame:Water	mixed	12.192	(-234.812,259.197)	*Confidence interval does not cross clinically important effect*	No concerns
Cyclamate:Saccharin	mixed	341.259	(-1236.891,1919.408)	*Confidence interval does not cross clinically important effect*	No concerns
Cyclamate:Sucrose	mixed	-7087.942	(-9286.855,-4889.028)	*Confidence interval does not cross clinically important effect*	No concerns
Cyclamate:Water	mixed	273.994	(-1226.403,1774.391)	*Confidence interval does not cross clinically important effect*	No concerns
Fructose:Glucose	mixed	-11988.576	(-15780.748,-8196.404)	*Confidence interval does not cross clinically important effect*	No concerns
Fructose:Sucralose	mixed	4213.584	(1522.787,6904.382)	*Confidence interval does not cross clinically important effect*	No concerns
Fructose:Sucrose	mixed	-3076.291	(-6329.973,177.391)	*Confidence interval extends into clinically important effects*	Some concerns
Glucose:Sucralose	mixed	16202.160	(13406.276,18998.044)	*Confidence interval does not cross clinically important effect*	No concerns
Glucose:Sucrose	mixed	8912.285	(5566.701,12257.869)	*Confidence interval does not cross clinically important effect*	No concerns
Saccharin:Sucrose	mixed	-7429.200	(-9404.130,-5454.270)	*Confidence interval does not cross clinically important effect*	No concerns
Saccharin:Water	mixed	-67.265	(-1097.412,962.883)	*Confidence interval does not cross clinically important effect*	No concerns
Sucralose:Sucrose	mixed	-7289.875	(-9162.592,-5417.159)	*Confidence interval does not cross clinically important effect*	No concerns

Comparison Sucralose:Water
Evidence: mixed
NMA estimate: 72.060
95% Confidence interval:
Confidence (-171.078,315.199)
interval does not cross clinically important effect
Imprecision judgment
No concerns

Comparison Sucrose:Water
Evidence: mixed
NMA estimate: 7361.936
95% Confidence interval:
Confidence (5492.058,9231.813)
interval does not cross clinically important effect
Imprecision judgment
No concerns

Comparison
Ace-K + Aspartame:Ace-K + Aspartame + Cyclamate
Evidence: indirect
NMA estimate: -2323.113
95% Confidence interval:
Confidence (-6283.459,1637.233)
interval extends into clinically important effects
Imprecision judgment
Some concerns

Comparison
Evidence: AceK:Ace-K + Aspartame indirect
NMA estimate: 2710.118
95% Confidence interval:
Confidence (-1336.526,6756.762)
interval extends into clinically important effects
Imprecision judgment
Some concerns

Comparison
Ace-K + Aspartame:Aspartame
Evidence: indirect
NMA estimate: -2862.256
95% Confidence interval:
Confidence (-6814.142,1089.629)
interval extends into clinically important effects
Imprecision judgment
Some concerns

Comparison
Ace-K + Aspartame:Cyclamate
Evidence: indirect
NMA estimate: -3124.058
95% Confidence interval:
Confidence (-7242.674,994.558)
interval extends into clinically important effects
Imprecision judgment
Some concerns

Comparison
Ace-K + Aspartame:Fructose
Evidence: indirect
NMA estimate: -7135.709
95% Confidence interval:
Confidence (-11901.654,-2369.765)
interval does not cross clinically important effect
Imprecision judgment
No concerns

Comparison
Ace-K + Aspartame:Glucose
Evidence: indirect
NMA estimate: -19124.285
95% Confidence interval:
Confidence (-23953.437,-14295.133)
interval does not cross clinically important effect
Imprecision judgment
No concerns

Comparison
Ace-K + Aspartame:Saccharin
Evidence: indirect
NMA estimate: -2782.800
95% Confidence interval:
Confidence (-6786.314,1220.715)
interval extends into clinically important effects
Imprecision judgment
Some concerns

Comparison
Ace-K + Aspartame:Sucralose
Evidence: indirect
NMA estimate: -2922.125
95% Confidence interval:
Confidence (-6876.217,1031.967)
interval extends into clinically important effects
Imprecision judgment
Some concerns

Comparison
Evidence: Ace-K + Aspartame:Water indirect
NMA estimate: -2850.064
95% Confidence interval:
Confidence (-6802.813,1102.684)
interval extends into clinically important effects
Imprecision judgment
Some concerns

Comparison
AceK:Ace-K + Aspartame + Cyclamate
Evidence: indirect
NMA estimate: 387.005
95% Confidence interval:
Confidence (-2406.579,3180.590)
interval extends into clinically important effects in ***both*** *directions*
Imprecision judgment
Major concerns

Comparison
Ace-K + Aspartame + Cyclamate:Aspartame
Evidence: indirect
NMA estimate: -539.144
95% Confidence interval:
Confidence (-3193.609,2115.322)
interval extends into clinically important effects
Imprecision judgment
Some concerns

Comparison
Ace-K + Aspartame + Cyclamate:Cyclamate
Evidence: indirect
NMA estimate: -800.945
95% Confidence interval:
Confidence (-3697.803,2095.912)
interval extends into clinically important effects
Imprecision judgment
Some concerns

Comparison
Ace-K + Aspartame + Cyclamate:Fructose
Evidence: indirect
NMA estimate: -4812.596
95% Confidence interval:
Confidence (-8573.316,-1051.876)
interval does not cross clinically important effect
Imprecision judgment
No concerns

Comparison
Ace-K + Aspartame + Cyclamate:Glucose
Evidence: indirect
NMA estimate: -16801.172
95% Confidence interval:
Confidence (-20641.679,-12960.665)
interval does not cross clinically important effect
Imprecision judgment
No concerns

Comparison
Ace-K + Aspartame + Cyclamate:Saccharin
Evidence: indirect
NMA estimate: -459.687
95% Confidence interval:
Confidence (-3190.421,2271.048)
interval extends into clinically important effects in ***both*** *directions*
Imprecision judgment
Major concerns

Comparison
Ace-K + Aspartame + Cyclamate:Sucralose
Evidence: indirect
NMA estimate: -599.012
95% Confidence interval:
Confidence (-3256.761,2058.738)
interval extends into clinically important effects
Imprecision judgment
Some concerns

Comparison
Ace-K + Aspartame + Cyclamate:Water
Evidence: indirect
NMA estimate: -526.951
95% Confidence interval:
Confidence (-3182.701,2128.798)
interval extends into clinically important effects
Imprecision judgment
Some concerns

Comparison AceK:Fructose
Evidence: indirect
NMA estimate: -4425.591
95% Confidence interval:
Confidence (-7409.577,-1441.605)
interval does not cross clinically important effect
Imprecision judgment
No concerns

Comparison AceK:Glucose
Evidence: indirect
NMA estimate: -16414.167
95% Confidence interval:
Confidence (-19489.147,-13339.187)
interval does not cross clinically important effect
Imprecision judgment
No concerns

Comparison AceK:Sucralose
Evidence: indirect
NMA estimate: -212.007
95% Confidence interval:
Confidence (-1510.784,1086.771)
interval does not cross clinically important effect
Imprecision judgment
No concerns

Comparison Aspartame:Fructose
Evidence: indirect
NMA estimate: -4273.453
95% Confidence interval:
Confidence (-6974.848,-1572.057)
interval does not cross clinically important effect
Imprecision judgment
No concerns

Comparison Cyclamate:Fructose
Evidence: indirect
NMA estimate: -4011.651
95% Confidence interval:
Confidence (-7092.534,-930.768)
interval does not cross clinically important effect
Imprecision judgment
No concerns

(continued on next page)

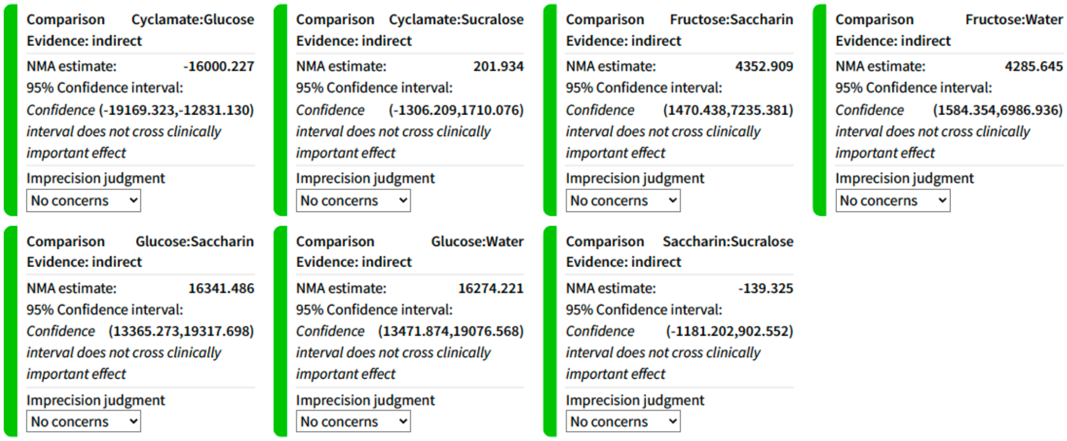

Relative effect estimates below -2250.0 and above 2250.0 pmol*min/L (minimal important difference; MID) were considered clinically important. Judgements for IMPRECISION were assigned by the CiNEMA algorithm (27, 28). There is no general consensus on a clinically important size difference for 2-hour blood insulin incremental area under the curve (iAUC). Health Canada considers a 20% reduction in glucose iAUC to be the minimum physiologically relevant difference as per “Guideline Concerning the Safety and Physiological Effects of Novel Fibre Sources and Food Products Containing Them” (29). Extrapolating the 20% threshold in glucose iAUC, we calculated the MID of insulin iAUC by taking 20% of 11250 pmol*mins/L which is the median of insulin iAUC in participants with type 2 diabetes (~6200 pmol*mins/L) and healthy participants (~16300 pmol*mins/L) after a 75-g oral glucose tolerance test (32-34).

***Figure S17**. CINeMA output for the HETEROGENEITY domain of UNCOUPLING INTERVENTIONS evaluating the effect of non-nutritive sweetened beverages (NNS beverages) sweetened single or blends of non-nutritive sweeteners (NNS), water, and sugar-sweetened beverages (SSBs) sweetened with caloric sweeteners on postprandial blood INSULIN response in HEALTHY participants*

Heterogeneity

Define clinically important size of effect: Mean difference 2250

*Relative effect estimates below **-2250.000** and above **2250.000** are considered clinically important.*
Importance of heterogeneity depends on the variability of effects in relation to a clinically important size of effect

The estimated value of between-study variance for the network meta-analysis is **514666.980**

Comparison Ace-K + Aspartame:Sucrose
Evidence: mixed
NMA estimate: -10212.000
95% intervals for NMA estimate
Confidence interval:
Prediction (-13694.496,-6729.504)
interval: (-14715.374,-5708.626)
*Confidence and prediction intervals **agree** in relation to clinically important effect*
Heterogeneity judgment: No concerns

Comparison Ace-K + Aspartame + Cyclamate:Sucrose
Evidence: mixed
NMA estimate: -7888.887
95% intervals for NMA estimate
Confidence interval:
Prediction (-9774.773,-6003.001)
interval: (-10815.343,-5068.962)
*Confidence and prediction intervals **agree** in relation to clinically important effect*
Heterogeneity judgment: No concerns

Comparison AceK:Aspartame
Evidence: mixed
NMA estimate: -152.138
95% intervals for NMA estimate
Confidence interval:
Prediction (-1438.527,1134.250)
interval: (-2568.588,2554.660)
*Prediction interval extends into clinically important effects in **both** directions*
Heterogeneity judgment: Major concerns

Comparison AceK:Cyclamate
Evidence: mixed
NMA estimate: -413.940
95% intervals for NMA estimate
Confidence interval:
Prediction (-2078.424,1250.544)
interval: (-3326.023,2498.142)
*Prediction interval extends into clinically important effects in **both** directions*
Heterogeneity judgment: Major concerns

Comparison AceK:Saccharin
Evidence: mixed
NMA estimate: -72.681
95% intervals for NMA estimate
Confidence interval:
Prediction (-1452.132,1306.769)
interval: (-2704.735,2580.682)
*Prediction interval extends into clinically important effects in **both** directions*
Heterogeneity judgment: Major concerns

Comparison AceK:Sucrose
Evidence: mixed
NMA estimate: -7501.882
95% intervals for NMA estimate
Confidence interval:
Prediction (-9562.841,-5440.923)
interval: (-10762.179,-4445.507)
*Confidence and prediction intervals **agree** in relation to clinically important effect*
Heterogeneity judgment: No concerns

Comparison AceK:Water
Evidence: mixed
NMA estimate: -139.946
95% intervals for NMA estimate
Confidence interval:
Prediction (-1429.722,1149.829)
interval: (-2787.418,2370.528)
*Prediction interval extends into clinically important effects in **both** directions*
Heterogeneity judgment: Major concerns

Comparison Aspartame:Cyclamate
Evidence: mixed
NMA estimate: -261.802
95% intervals for NMA estimate
Confidence interval:
Prediction (-1759.288,1235.685)
interval: (-3109.384,2295.432)
*Prediction interval extends into clinically important effects in **both** directions*
Heterogeneity judgment: Major concerns

Comparison Aspartame:Glucose
Evidence: mixed
NMA estimate: -16262.029
95% intervals for NMA estimate
Confidence interval:
Prediction (-19060.503,-13463.554)
interval: (-20155.664,-12645.917)
*Confidence and prediction intervals **agree** in relation to clinically important effect*
Heterogeneity judgment: No concerns

Comparison Aspartame:Saccharin
Evidence: mixed
NMA estimate: 79.457
95% intervals for NMA estimate
Confidence interval:
Prediction (-946.881,1105.794)
interval: (-2294.098,2183.972)
*Prediction interval **extends into clinically important or unimportant effects***
Heterogeneity judgment: Some concerns

Comparison Aspartame:Sucralose
Evidence: mixed
NMA estimate: -59.868
95% intervals for NMA estimate
Confidence interval:
Prediction (-306.609,186.872)
interval: (-2172.625,1922.657)
*Confidence and prediction intervals **agree** in relation to clinically important effect*
Heterogeneity judgment: No concerns

Comparison Aspartame:Sucrose
Evidence: mixed
NMA estimate: -7349.743
95% intervals for NMA estimate
Confidence interval:
Prediction (-9217.797,-5481.690)
interval: (-10474.758,-4718.999)
*Confidence and prediction intervals **agree** in relation to clinically important effect*
Heterogeneity judgment: No concerns

Comparison Aspartame:Water
Evidence: mixed
NMA estimate: 12.192
95% intervals for NMA estimate
Confidence interval:
Prediction (-234.812,259.197)
interval: (-2123.344,1720.381)
*Confidence and prediction intervals **agree** in relation to clinically important effect*
Heterogeneity judgment: No concerns

Comparison Cyclamate:Saccharin
Evidence: mixed
NMA estimate: 341.259
95% intervals for NMA estimate
Confidence interval:
Prediction (-1236.891,1919.408)
interval: (-2427.475,3131.302)
*Prediction interval extends into clinically important effects in **both** directions*
Heterogeneity judgment: Major concerns

Comparison Cyclamate:Sucrose
Evidence: mixed
NMA estimate: -7087.942
95% intervals for NMA estimate
Confidence interval:
Prediction (-9286.855,-4889.028)
interval: (-10463.460,-3916.345)
*Confidence and prediction intervals **agree** in relation to clinically important effect*
Heterogeneity judgment: No concerns

Comparison Cyclamate:Water
Evidence: mixed
NMA estimate: 273.994
95% intervals for NMA estimate
Confidence interval:
Prediction (-1226.403,1774.391)
interval: (-2513.364,2924.354)
*Prediction interval extends into clinically important effects in **both** directions*
Heterogeneity judgment: Major concerns

(continued on next page)

Comparison Fructose:Glucose
Evidence: mixed
NMA estimate: -11988.576
95% intervals for NMA estimate
Confidence interval: (-15780.748,-8196.404)
Prediction interval: (-16802.681,-7223.754)
*Confidence and prediction intervals **agree** in relation to clinically important effect*
Heterogeneity judgment
No concerns

Comparison Fructose:Sucralose
Evidence: mixed
NMA estimate: 4213.584
95% intervals for NMA estimate
Confidence interval: (1522.787,6904.382)
Prediction interval: (523.537,8001.642)
*Confidence and prediction intervals **agree** in relation to clinically important effect*
Heterogeneity judgment
No concerns

Comparison Fructose:Sucrose
Evidence: mixed
NMA estimate: -3076.291
95% intervals for NMA estimate
Confidence interval: (-6329.973,177.391)
Prediction interval: (-7616.677,1198.067)
*Confidence and prediction intervals **agree** in relation to clinically important effect*
Heterogeneity judgment
No concerns

Comparison Glucose:Sucralose
Evidence: mixed
NMA estimate: 16202.160
95% intervals for NMA estimate
Confidence interval: (13406.276,18998.044)
Prediction interval: (12560.371,19991.243)
*Confidence and prediction intervals **agree** in relation to clinically important effect*
Heterogeneity judgment
No concerns

Comparison Glucose:Sucrose
Evidence: mixed
NMA estimate: 8912.285
95% intervals for NMA estimate
Confidence interval: (5566.701,12257.869)
Prediction interval: (4473.325,13134.499)
*Confidence and prediction intervals **agree** in relation to clinically important effect*
Heterogeneity judgment
No concerns

Comparison Saccharin:Sucrose
Evidence: mixed
NMA estimate: -7429.200
95% intervals for NMA estimate
Confidence interval: (-9404.130,-5454.270)
Prediction interval: (-10547.703,-4535.929)
*Confidence and prediction intervals **agree** in relation to clinically important effect*
Heterogeneity judgment
No concerns

Comparison Saccharin:Water
Evidence: mixed
NMA estimate: -67.265
95% intervals for NMA estimate
Confidence interval: (-1097.412,962.883)
Prediction interval: (-2404.156,2111.319)
*Prediction interval **extends into clinically important or unimportant effects***
Heterogeneity judgment
Some concerns

Comparison Sucralose:Sucrose
Evidence: mixed
NMA estimate: -7289.875
95% intervals for NMA estimate
Confidence interval: (-9162.592,-5417.159)
Prediction interval: (-10428.627,-4515.162)
*Confidence and prediction intervals **agree** in relation to clinically important effect*
Heterogeneity judgment
No concerns

Comparison Sucralose:Water
Evidence: mixed
NMA estimate: 72.060
95% intervals for NMA estimate
Confidence interval: (-171.078,315.199)
Prediction interval: (-2033.877,1880.883)
*Confidence and prediction intervals **agree** in relation to clinically important effect*
Heterogeneity judgment
No concerns

Comparison Sucrose:Water
Evidence: mixed
NMA estimate: 7361.936
95% intervals for NMA estimate
Confidence interval: (5492.058,9231.813)
Prediction interval: (4515.164,10275.630)
*Confidence and prediction intervals **agree** in relation to clinically important effect*
Heterogeneity judgment
No concerns

Comparison Ace-K + Aspartame:Ace-K + Aspartame + Cyclamate
Evidence: indirect
NMA estimate: -2323.113
95% intervals for NMA estimate
Confidence interval: (-6283.459,1637.233)
Prediction interval: (-7373.021,2833.326)
*Prediction interval **extends** into clinically important or unimportant effects*
Heterogeneity judgment
Some concerns

Comparison AceK:Ace-K + Aspartame
Evidence: indirect
NMA estimate: 2710.118
95% intervals for NMA estimate
Confidence interval: (-1336.526,6756.762)
Prediction interval: (-2660.831,7877.146)
*Prediction interval **extends** into clinically important or unimportant effects*
Heterogeneity judgment
Some concerns

Comparison Ace-K + Aspartame:Aspartame
Evidence: indirect
NMA estimate: -2862.256
95% intervals for NMA estimate
Confidence interval: (-6814.142,1089.629)
Prediction interval: (-7720.936,2490.693)
*Prediction interval **extends** into clinically important or unimportant effects*
Heterogeneity judgment
Some concerns

Comparison Ace-K + Aspartame:Cyclamate
Evidence: indirect
NMA estimate: -3124.058
95% intervals for NMA estimate
Confidence interval: (-7242.674,994.558)
Prediction interval: (-8360.949,2316.754)
*Prediction interval **extends** into clinically important or unimportant effects*
Heterogeneity judgment
Some concerns

Comparison Ace-K + Aspartame:Fructose
Evidence: indirect
NMA estimate: -7135.709
95% intervals for NMA estimate
Confidence interval: (-11901.654,-2369.765)
Prediction interval: (-13102.866,-902.524)
*Confidence and prediction intervals **agree** in relation to clinically important effect*
Heterogeneity judgment
No concerns

Comparison Ace-K + Aspartame:Glucose
Evidence: indirect
NMA estimate: -19124.285
95% intervals for NMA estimate
Confidence interval: (-23953.437,-14295.133)
Prediction interval: (-25060.839,-12970.986)
*Confidence and prediction intervals **agree** in relation to clinically important effect*
Heterogeneity judgment
No concerns

Comparison Ace-K + Aspartame:Saccharin
Evidence: indirect
NMA estimate: -2782.800
95% intervals for NMA estimate
Confidence interval: (-6786.314,1220.715)
Prediction interval: (-7849.230,2508.862)
*Prediction interval **extends** into clinically important or unimportant effects*
Heterogeneity judgment
Some concerns

Comparison Ace-K + Aspartame:Sucralose
Evidence: indirect
NMA estimate: -2922.125
95% intervals for NMA estimate
Confidence interval: (-6876.217,1031.967)
Prediction interval: (-7890.777,2410.566)
*Prediction interval **extends** into clinically important or unimportant effects*
Heterogeneity judgment
Some concerns

Comparison Ace-K + Aspartame:Water
Evidence: indirect
NMA estimate: -2850.064
95% intervals for NMA estimate
Confidence interval: (-6802.813,1102.684)
Prediction interval: (-7923.745,2290.539)
*Prediction interval **extends** into clinically important or unimportant effects*
Heterogeneity judgment
Some concerns

Comparison AceK:Ace-K + Aspartame + Cyclamate
Evidence: indirect
NMA estimate: 387.005
95% intervals for NMA estimate
Confidence interval: (-2406.579,3180.590)
Prediction interval: (-3628.690,4305.310)
*Confidence and prediction intervals **agree** in relation to clinically important effect*
Heterogeneity judgment
No concerns

(continued on next page)

Comparison Ace-K + Aspartame + Cyclamate:Aspartame
Evidence: indirect
NMA estimate: -539.144
95% intervals for NMA estimate
Confidence interval: (-3193.609,2115.322)
Prediction interval: (-4092.833,3402.284)
*Prediction interval **extends** into clinically important or unimportant effects*
Heterogeneity judgment: Some concerns

Comparison Ace-K + Aspartame + Cyclamate:Cyclamate
Evidence: indirect
NMA estimate: -800.945
95% intervals for NMA estimate
Confidence interval: (-3697.803,2095.912)
Prediction interval: (-4811.583,3307.082)
*Prediction interval **extends** into clinically important or unimportant effects*
Heterogeneity judgment: Some concerns

Comparison Ace-K + Aspartame + Cyclamate:Fructose
Evidence: indirect
NMA estimate: -4812.596
95% intervals for NMA estimate
Confidence interval: (-8573.316,-1051.876)
Prediction interval: (-9751.506,285.810)
*Prediction interval **extends into clinically important or unimportant effects***
Heterogeneity judgment: Some concerns

Comparison Ace-K + Aspartame + Cyclamate:Glucose
Evidence: indirect
NMA estimate: -16801.172
95% intervals for NMA estimate
Confidence interval: (-20641.679,-12960.665)
Prediction interval: (-21697.427,-11794.703)
*Confidence and prediction intervals **agree** in relation to clinically important effect*
Heterogeneity judgment: No concerns

Comparison Ace-K + Aspartame + Cyclamate:Saccharin
Evidence: indirect
NMA estimate: -459.687
95% intervals for NMA estimate
Confidence interval: (-3190.421,2271.048)
Prediction interval: (-4247.071,3446.397)
*Confidence and prediction intervals **agree** in relation to clinically important effect*
Heterogeneity judgment: No concerns

Comparison Ace-K + Aspartame + Cyclamate:Sucralose
Evidence: indirect
NMA estimate: -599.012
95% intervals for NMA estimate
Confidence interval: (-3256.761,2058.738)
Prediction interval: (-4278.705,3338.189)
*Prediction interval **extends** into clinically important or unimportant effects*
Heterogeneity judgment: Some concerns

Comparison Ace-K + Aspartame + Cyclamate:Water
Evidence: indirect
NMA estimate: -526.951
95% intervals for NMA estimate
Confidence interval: (-3182.701,2128.798)
Prediction interval: (-4296.122,3202.611)
*Prediction interval **extends** into clinically important or unimportant effects*
Heterogeneity judgment: Some concerns

Comparison AceK:Fructose
Evidence: indirect
NMA estimate: -4425.591
95% intervals for NMA estimate
Confidence interval: (-7409.577,-1441.605)
Prediction interval: (-8710.891,-78.184)
*Confidence and prediction intervals **agree** in relation to clinically important effect*
Heterogeneity judgment: No concerns

Comparison AceK:Glucose
Evidence: indirect
NMA estimate: -16414.167
95% intervals for NMA estimate
Confidence interval: (-19489.147,-13339.187)
Prediction interval: (-20605.599,-12209.911)
*Confidence and prediction intervals **agree** in relation to clinically important effect*
Heterogeneity judgment: No concerns

Comparison AceK:Sucralose
Evidence: indirect
NMA estimate: -212.007
95% intervals for NMA estimate
Confidence interval: (-1510.784,1086.771)
Prediction interval: (-2863.423,2599.527)
*Prediction interval extends into clinically important effects in **both** directions*
Heterogeneity judgment: Major concerns

Comparison Aspartame:Fructose
Evidence: indirect
NMA estimate: -4273.453
95% intervals for NMA estimate
Confidence interval: (-6974.848,-1572.057)
Prediction interval: (-8314.656,-460.492)
*Confidence and prediction intervals **agree** in relation to clinically important effect*
Heterogeneity judgment: No concerns

Comparison Cyclamate:Fructose
Evidence: indirect
NMA estimate: -4011.651
95% intervals for NMA estimate
Confidence interval: (-7092.534,-930.768)
Prediction interval: (-8381.961,420.766)
*Prediction interval **extends into clinically important or unimportant effects***
Heterogeneity judgment: Some concerns

Comparison Cyclamate:Glucose
Evidence: indirect
NMA estimate: -16000.227
95% intervals for NMA estimate
Confidence interval: (-19169.323,-12831.130)
Prediction interval: (-20279.020,-11708.609)
*Confidence and prediction intervals **agree** in relation to clinically important effect*
Heterogeneity judgment: No concerns

Comparison Cyclamate:Sucralose
Evidence: indirect
NMA estimate: 201.934
95% intervals for NMA estimate
Confidence interval: (-1306.209,1710.076)
Prediction interval: (-2581.930,3145.914)
*Prediction interval extends into clinically important effects in **both** directions*
Heterogeneity judgment: Major concerns

Comparison Fructose:Saccharin
Evidence: indirect
NMA estimate: 4352.909
95% intervals for NMA estimate
Confidence interval: (1470.438,7235.381)
Prediction interval: (192.725,8472.297)
*Confidence and prediction intervals **agree** in relation to clinically important effect*
Heterogeneity judgment: No concerns

Comparison Fructose:Water
Evidence: indirect
NMA estimate: 4285.645
95% intervals for NMA estimate
Confidence interval: (1584.354,6986.936)
Prediction interval: (294.621,8077.563)
*Confidence and prediction intervals **agree** in relation to clinically important effect*
Heterogeneity judgment: No concerns

Comparison Glucose:Saccharin
Evidence: indirect
NMA estimate: 16341.486
95% intervals for NMA estimate
Confidence interval: (13365.273,19317.698)
Prediction interval: (12332.184,20359.272)
*Confidence and prediction intervals **agree** in relation to clinically important effect*
Heterogeneity judgment: No concerns

Comparison Glucose:Water
Evidence: indirect
NMA estimate: 16274.221
95% intervals for NMA estimate
Confidence interval: (13471.874,19076.568)
Prediction interval: (12409.777,19988.842)
*Confidence and prediction intervals **agree** in relation to clinically important effect*
Heterogeneity judgment: No concerns

Comparison Saccharin:Sucralose
Evidence: indirect
NMA estimate: -139.325
95% intervals for NMA estimate
Confidence interval: (-1181.202,902.552)
Prediction interval: (-2506.110,2366.267)
*Prediction interval extends into clinically important effects in **both** directions*
Heterogeneity judgment: Major concerns

Judgements for were assigned by the CiNEMA algorithm (27, 28).

Figure S18. *CINeMA output for the INCOHERENCE domain of UNCOUPLING INTERVENTIONS evaluating the effect of non-nutritive sweetened beverages (NNS beverages) sweetened single or blends of non-nutritive sweeteners (NNS), water, and sugar-sweetened beverages (SSBs) sweetened with caloric sweeteners on postprandial blood INSULIN response in HEALTHY participants*

Incoherence

Define clinically important size of effect: Mean difference 2250

*Relative effect estimates below **-2250.000** and above **2250.000** are considered clinically important.*
Importance of Incoherence depends on the variability of direct and indirect effects in relation to a clinically important size of effect

Global test based on a random-effects design-by-treatment interaction model
χ^2 statistic: 3.269 (8 degrees of freedom), P value: 0.916

Local tests: Separating indirect from direct evidence

**Comparison**	**Ace-K + Aspartame:Sucrose**
**Evidence: direct**	
Direct mean difference:	**-10212.000(-13694.496,-6729.504)**
**Inconsistency measures:** Not applicable	
Incoherence judgment	No concerns

**Comparison**	**Ace-K + Aspartame + Cyclamate:Sucrose**
**Evidence: direct**	
Direct mean difference:	**-7888.887(-9774.773,-6003.001)**
**Inconsistency measures:** Not applicable	
Incoherence judgment	No concerns

**Comparison**	**AceK:Aspartame**
**Evidence: mixed**	
NMA mean difference:	**-152.138(-1438.527,1134.250)**
Direct mean difference:	**-69.900(-1570.771,1430.971)**
Indirect mean difference:	**-379.782(-2876.865,2117.301)**
**Inconsistency measures**	
Difference of mean differences:	**309.882(-2603.542,3223.306)**
P value:	**0.835**
Incoherence judgment	No concerns

**Comparison**	**AceK:Cyclamate**
**Evidence: direct**	
Direct mean difference:	**-413.940(-2078.428,1250.548)**
**Inconsistency measures:** Not applicable	
Incoherence judgment	No concerns

**Comparison**	**AceK:Saccharin**
**Evidence: mixed**	
NMA mean difference:	**-72.681(-1452.132,1306.769)**
Direct mean difference:	**-42.840(-1516.091,1430.411)**
Indirect mean difference:	**-284.903(-4213.700,3643.894)**
**Inconsistency measures**	
Difference of mean differences:	**242.063(-3953.878,4438.003)**
P value:	**0.910**
Incoherence judgment	No concerns

**Comparison**	**AceK:Sucrose**
**Evidence: mixed**	
NMA mean difference:	**-7501.882(-9562.841,-5440.923)**
Direct mean difference:	**-6772.740(-9020.087,-4525.393)**
Indirect mean difference:	**-11358.715(-16527.389,-6190.041)**
**Inconsistency measures**	
Difference of mean differences:	**4585.975(-1050.137,10222.087)**
P value:	**0.111**
Incoherence judgment	No concerns

**Comparison**	**AceK:Water**
**Evidence: mixed**	
NMA mean difference:	**-139.946(-1429.722,1149.829)**
Direct mean difference:	**-590.760(-2229.785,1048.265)**
Indirect mean difference:	**593.223(-1496.979,2683.425)**
**Inconsistency measures**	
Difference of mean differences:	**-1183.983(-3840.173,1472.207)**
P value:	**0.382**
Incoherence judgment	No concerns

**Comparison**	**Aspartame:Cyclamate**
**Evidence: mixed**	
NMA mean difference:	**-261.802(-1759.288,1235.685)**
Direct mean difference:	**-344.040(-2029.356,1341.276)**
Indirect mean difference:	**46.685(-3217.403,3310.773)**
**Inconsistency measures**	
Difference of mean differences:	**-390.725(-4064.219,3282.769)**
P value:	**0.835**
Incoherence judgment	No concerns

**Comparison**	**Aspartame:Glucose**
**Evidence: mixed**	
NMA mean difference:	**-16262.029(-19060.503,-13463.554)**
Direct mean difference:	**-20107.282(-24690.853,-15523.710)**
Indirect mean difference:	**-13976.887(-17510.371,-10443.403)**
**Inconsistency measures**	
Difference of mean differences:	**-6130.395(-11917.850,-342.940)**
P value:	**0.038**
Incoherence judgment	No concerns

**Comparison**	**Aspartame:Saccharin**
**Evidence: mixed**	
NMA mean difference:	**79.457(-946.881,1105.794)**
Direct mean difference:	**17.353(-1146.307,1181.013)**
Indirect mean difference:	**296.989(-1880.860,2474.838)**
**Inconsistency measures**	
Difference of mean differences:	**-279.636(-2748.873,2189.602)**
P value:	**0.824**
Incoherence judgment	No concerns

**Comparison**	**Aspartame:Sucralose**
**Evidence: mixed**	
NMA mean difference:	**-59.868(-306.609,186.872)**
Direct mean difference:	**-62.160(-312.870,188.550)**
Indirect mean difference:	**10.780(-1381.236,1402.796)**
**Inconsistency measures**	
Difference of mean differences:	**-72.940(-1487.353,1341.473)**
P value:	**0.919**
Incoherence judgment	No concerns

**Comparison**	**Aspartame:Sucrose**
**Evidence: mixed**	
NMA mean difference:	**-7349.743(-9217.797,-5481.690)**
Direct mean difference:	**-7013.567(-9146.269,-4880.866)**
Indirect mean difference:	**-8457.721(-12329.518,-4585.925)**
**Inconsistency measures**	
Difference of mean differences:	**1444.154(-2976.166,5864.473)**
P value:	**0.522**
Incoherence judgment	No concerns

**Comparison**	**Aspartame:Water**
**Evidence: mixed**	
NMA mean difference:	**12.192(-234.812,259.197)**
Direct mean difference:	**29.603(-219.571,278.777)**
Indirect mean difference:	**-974.385(-2850.067,901.297)**
**Inconsistency measures**	
Difference of mean differences:	**1003.988(-888.172,2896.149)**
P value:	**0.298**
Incoherence judgment	No concerns

**Comparison**	**Cyclamate:Saccharin**
**Evidence: mixed**	
NMA mean difference:	**341.259(-1236.891,1919.408)**
Direct mean difference:	**371.100(-1289.666,2031.866)**
Indirect mean difference:	**63.498(-5003.298,5130.293)**
**Inconsistency measures**	
Difference of mean differences:	**307.602(-5024.430,5639.634)**
P value:	**0.910**
Incoherence judgment	No concerns

**Comparison**	**Cyclamate:Sucrose**
**Evidence: mixed**	
NMA mean difference:	**-7087.942(-9286.855,-4889.028)**
Direct mean difference:	**-6358.800(-8733.299,-3984.301)**
Indirect mean difference:	**-11478.392(-17305.053,-5651.730)**
**Inconsistency measures**	
Difference of mean differences:	**5119.592(-1172.326,11411.510)**
P value:	**0.111**
Incoherence judgment	No concerns

(continued on next page)

Comparison	Cyclamate:Water
Evidence: mixed	
NMA mean difference:	273.994(-1226.403,1774.391)
Direct mean difference:	-176.820(-1986.262,1632.622)
Indirect mean difference:	1266.171(-1418.180,3950.523)
**Inconsistency measures**	
Difference of mean differences:	-1442.991(-4680.247,1794.264)
P value:	0.382
Incoherence judgment	No concerns

Comparison	Fructose:Glucose
Evidence: mixed	
NMA mean difference:	-11988.576(-15780.748,-8196.404)
Direct mean difference:	-9812.530(-14125.289,-5499.771)
Indirect mean difference:	-19404.153(-27365.748,-11442.559)
**Inconsistency measures**	
Difference of mean differences:	9591.623(536.963,18646.284)
P value:	0.038
Incoherence judgment	No concerns

Comparison	Fructose:Sucralose
Evidence: mixed	
NMA mean difference:	4213.584(1522.787,6904.382)
Direct mean difference:	4185.320(1490.947,6879.693)
Indirect mean difference:	14755.722(-37280.641,66792.085)
**Inconsistency measures**	
Difference of mean differences:	-10570.402(-62676.473,41535.670)
P value:	0.691
Incoherence judgment	No concerns

Comparison	Fructose:Sucrose
Evidence: mixed	
NMA mean difference:	-3076.291(-6329.973,177.391)
Direct mean difference:	-6118.220(-11581.148,-655.292)
Indirect mean difference:	-1404.068(-5454.510,2646.374)
**Inconsistency measures**	
Difference of mean differences:	-4714.152(-11514.862,2086.558)
P value:	0.174
Incoherence judgment	No concerns

Comparison	Glucose:Sucralose
Evidence: mixed	
NMA mean difference:	16202.160(13406.276,18998.044)
Direct mean difference:	13997.850(10471.257,17524.443)
Indirect mean difference:	19931.674(15344.448,24518.900)
**Inconsistency measures**	
Difference of mean differences:	-5933.824(-11719.971,-147.677)
P value:	0.044
Incoherence judgment	No concerns

Comparison	Glucose:Sucrose
Evidence: mixed	
NMA mean difference:	8912.285(5566.701,12257.869)
Direct mean difference:	3694.310(-2223.527,9612.147)
Indirect mean difference:	11363.304(7307.377,15419.231)
**Inconsistency measures**	
Difference of mean differences:	-7668.994(-14843.347,-494.642)
P value:	0.036
Incoherence judgment	Some concerns

Comparison	Saccharin:Sucrose
Evidence: mixed	
NMA mean difference:	-7429.200(-9404.130,-5454.270)
Direct mean difference:	-6729.900(-8974.492,-4485.308)
Indirect mean difference:	-9826.303(-13982.045,-5670.561)
**Inconsistency measures**	
Difference of mean differences:	3096.403(-1626.772,7819.578)
P value:	0.199
Incoherence judgment	No concerns

Comparison	Saccharin:Water
Evidence: mixed	
NMA mean difference:	-67.265(-1097.412,962.883)
Direct mean difference:	-321.082(-1572.876,930.711)
Indirect mean difference:	465.292(-1347.939,2278.523)
**Inconsistency measures**	
Difference of mean differences:	-786.374(-2989.734,1416.985)
P value:	0.484
Incoherence judgment	No concerns

Comparison	Sucralose:Sucrose
Evidence: mixed	
NMA mean difference:	-7289.875(-9162.592,-5417.159)
Direct mean difference:	-9979.606(-13849.114,-6110.099)
Indirect mean difference:	-6467.180(-8607.217,-4327.143)
**Inconsistency measures**	
Difference of mean differences:	-3512.427(-7934.287,909.434)
P value:	0.119
Incoherence judgment	No concerns

Comparison	Sucralose:Water
Evidence: mixed	
NMA mean difference:	72.060(-171.078,315.199)
Direct mean difference:	78.873(-165.877,323.623)
Indirect mean difference:	-440.272(-2562.704,1682.160)
**Inconsistency measures**	
Difference of mean differences:	519.145(-1617.352,2655.643)
P value:	0.634
Incoherence judgment	No concerns

Comparison	Sucrose:Water
Evidence: mixed	
NMA mean difference:	7361.936(5492.058,9231.813)
Direct mean difference:	6583.525(4372.751,8794.299)
Indirect mean difference:	9318.438(5813.499,12823.378)
**Inconsistency measures**	
Difference of mean differences:	-2734.913(-6878.840,1409.013)
P value:	0.196
Incoherence judgment	No concerns

Comparison	Ace-K + Aspartame:Ace-K + Aspartame + Cyclamate
Evidence: indirect	
Indirect mean difference:	-2323.113(-6283.459,1637.233)
**Inconsistency measures:** Not applicable	
Incoherence judgment	No concerns

Comparison	AceK:Ace-K + Aspartame
Evidence: indirect	
Indirect mean difference:	2710.118(-1336.526,6756.762)
**Inconsistency measures:** Not applicable	
Incoherence judgment	No concerns

Comparison	Ace-K + Aspartame:Aspartame
Evidence: indirect	
Indirect mean difference:	-2862.256(-6814.142,1089.629)
**Inconsistency measures:** Not applicable	
Incoherence judgment	No concerns

Comparison	Ace-K + Aspartame:Cyclamate
Evidence: indirect	
Indirect mean difference:	-3124.058(-7242.674,994.558)
**Inconsistency measures:** Not applicable	
Incoherence judgment	No concerns

Comparison	Ace-K + Aspartame:Fructose
Evidence: indirect	
Indirect mean difference:	-7135.709(-11901.654,-2369.765)
**Inconsistency measures:** Not applicable	
Incoherence judgment	No concerns

Comparison	Ace-K + Aspartame:Glucose
Evidence: indirect	
Indirect mean difference:	-19124.285(-23953.437,-14295.133)
**Inconsistency measures:** Not applicable	
Incoherence judgment	No concerns

Comparison	Ace-K + Aspartame:Saccharin
Evidence: indirect	
Indirect mean difference:	-2782.800(-6786.314,1220.715)
**Inconsistency measures:** Not applicable	
Incoherence judgment	No concerns

Comparison	Ace-K + Aspartame:Sucralose
Evidence: indirect	
Indirect mean difference:	-2922.125(-6876.217,1031.967)
**Inconsistency measures:** Not applicable	
Incoherence judgment	No concerns

Comparison	Ace-K + Aspartame:Water
Evidence: indirect	
Indirect mean difference:	-2850.064(-6802.813,1102.684)
**Inconsistency measures:** Not applicable	
Incoherence judgment	No concerns

Comparison	AceK:Ace-K + Aspartame + Cyclamate
Evidence: indirect	
Indirect mean difference:	387.005(-2406.579,3180.590)
**Inconsistency measures:** Not applicable	
Incoherence judgment	No concerns

(continued on next page)

Comparison	Evidence	Indirect mean difference	Inconsistency measures	Incoherence judgment
Ace-K + Aspartame + Cyclamate:Aspartame	indirect	-539.144(-3193.609,2115.322)	Not applicable	No concerns
Ace-K + Aspartame + Cyclamate:Cyclamate	indirect	-800.945(-3697.803,2095.912)	Not applicable	No concerns
Ace-K + Aspartame + Cyclamate:Fructose	indirect	-4812.596(-8573.316,-1051.876)	Not applicable	No concerns
Ace-K + Aspartame + Cyclamate:Glucose	indirect	-16801.172(-20641.679,-12960.665)	Not applicable	No concerns
Ace-K + Aspartame + Cyclamate:Saccharin	indirect	-459.687(-3190.421,2271.048)	Not applicable	No concerns
Ace-K + Aspartame + Cyclamate:Sucralose	indirect	-599.012(-3256.761,2058.738)	Not applicable	No concerns
Ace-K + Aspartame + Cyclamate:Water	indirect	-526.951(-3182.701,2128.798)	Not applicable	No concerns
AceK:Fructose	indirect	-4425.591(-7409.577,-1441.605)	Not applicable	No concerns
AceK:Glucose	indirect	-16414.167(-19489.147,-13339.187)	Not applicable	No concerns
AceK:Sucralose	indirect	-212.007(-1510.784,1086.771)	Not applicable	No concerns
Aspartame:Fructose	indirect	-4273.453(-6974.848,-1572.057)	Not applicable	No concerns
Cyclamate:Fructose	indirect	-4011.651(-7092.534,-930.768)	Not applicable	No concerns
Cyclamate:Glucose	indirect	-16000.227(-19169.323,-12831.130)	Not applicable	No concerns
Cyclamate:Sucralose	indirect	201.934(-1306.209,1710.076)	Not applicable	No concerns
Fructose:Saccharin	indirect	4352.909(1470.438,7235.381)	Not applicable	No concerns
Fructose:Water	indirect	4285.645(1584.354,6986.936)	Not applicable	No concerns
Glucose:Saccharin	indirect	16341.486(13365.273,19317.698)	Not applicable	No concerns
Glucose:Water	indirect	16274.221(13471.874,19076.568)	Not applicable	No concerns
Saccharin:Sucralose	indirect	-139.325(-1181.202,902.552)	Not applicable	No concerns

Judgements for were assigned by the CiNEMA algorithm (27, 28).

Figure S19. *Network plot and meta-analysis of UNCOUPLING INTERVENTIONS evaluating the effect of non-nutritive sweetened beverages (NNS beverages) sweetened single or blends of non-nutritive sweeteners (NNS), water, and sugar-sweetened beverages (SSBs) sweetened with caloric sweeteners on postprandial blood INSULIN response in participants with TYPE 2 DIABETES*

Network Plot

5 Trials | 47 Participants
(Type 2 Diabetes)

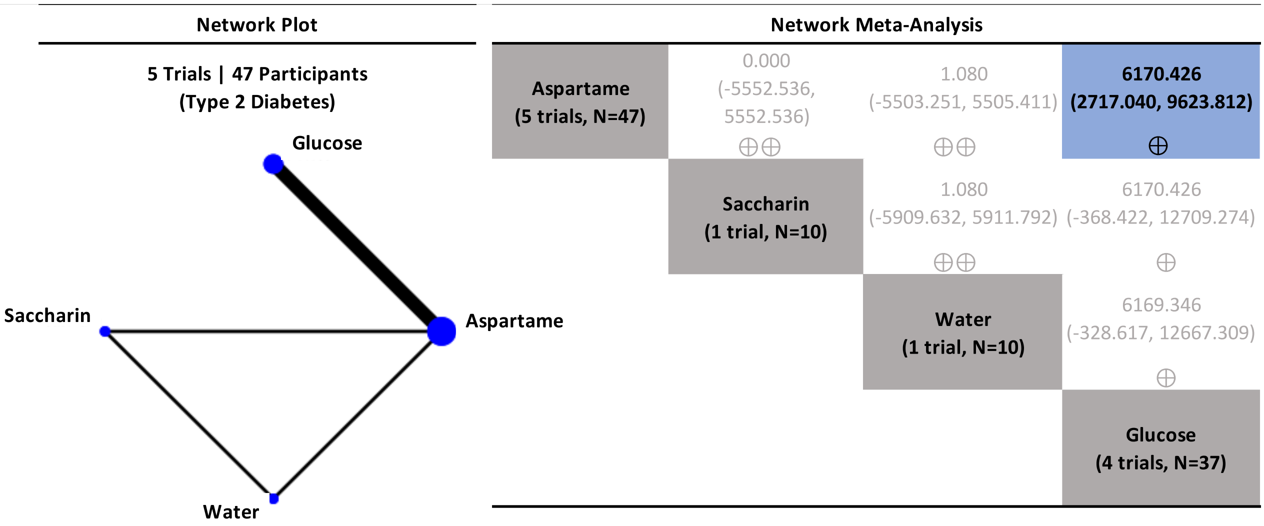

Network Meta-Analysis

**Aspartame (5 trials, N=47)**	0.000 (-5552.536, 5552.536) ⊕⊕	1.080 (-5503.251, 5505.411) ⊕⊕	**6170.426 (2717.040, 9623.812)** ⊕
	**Saccharin (1 trial, N=10)**	1.080 (-5909.632, 5911.792) ⊕⊕	6170.426 (-368.422, 12709.274) ⊕
		**Water (1 trial, N=10)**	6169.346 (-328.617, 12667.309) ⊕
			**Glucose (4 trials, N=37)**

Network plot: the size of the blue nodes is proportional to the number of participants and the line width is proportional to the number of studies. Network table: treatments are grouped by treatment type (i.e., individual non-nutritive sweeteners (NNS), NNS blends, water and caloric sweeteners) and are reported in alphabetical order. Treatment estimates (pmol*min/L) are MDs and 95% CIs of the column-defining treatment compared with the row-defining treatment. MDs less than 0 favor the column-defining treatment. MDs greater than 0 favor the row-defining treatment. Significant results are bolded in black. Non-significant results are grey and not bolded. The minimally important difference (MID) for postprandial glucose response is 2250 pmol*min/L. Trivial (significant) effects (<1 MID) or no effects have a white background; small important effects (≥1 MID) have a light blue background; moderate effects (≥2 MID) have a darker blue background; large effects (≥5 to <10 MID) have a purple background; very large effects (≥10 MID) have a black background. Confidence in the effect estimate is shown for each treatment comparison: high confidence ⊕⊕⊕⊕; moderate confidence ⊕⊕⊕; low confidence ⊕⊕; very low confidence ⊕. See **Supplementary Table 9 and Figures 20-23** for detailed assessments of the confidence in the effect estimate using the CINeMA framework.

Figure S20. *Risk of bias assessment of UNCOUPLING INTERVENTIONS evaluating the effect of non-nutritive sweetened beverages (NNS beverages) sweetened single or blends of non-nutritive sweeteners (NNS), water, and sugar-sweetened beverages (SSBs) sweetened with caloric sweeteners on postprandial blood INSULIN response in participants with TYPE 2 DIABETES*

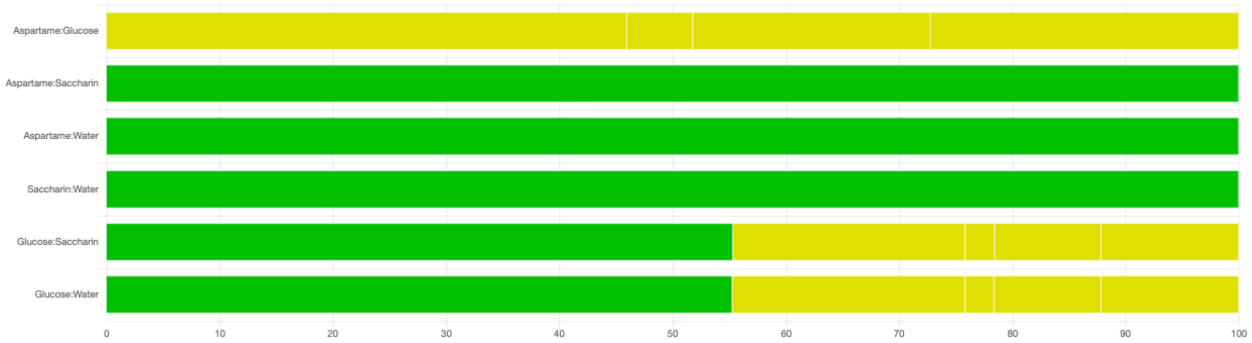

Green = no concerns; yellow = some concerns; red = major concerns

Figure S21. *CINeMA output for the IMPRECISION domain of UNCOUPLING INTERVENTIONS evaluating the effect of non-nutritive sweetened beverages (NNS beverages) sweetened single or blends of non-nutritive sweeteners (NNS), water, and sugar-sweetened beverages (SSBs) sweetened with caloric sweeteners on postprandial blood INSULIN response in participants with TYPE 2 DIABETES*

Imprecision

Define clinically important size of effect: Mean difference 2250

Relative effect estimates below ***-2250.000*** *and above* ***2250.000*** *are considered clinically important.*

Comparison Aspartame:Glucose
Evidence: mixed
NMA estimate: -6170.426
95% Confidence interval:
Confidence (-9623.812,-2717.040) *interval does not cross clinically important effect*
Imprecision judgment
No concerns

Comparison Aspartame:Saccharin
Evidence: mixed
NMA estimate: 0.000
95% Confidence interval:
Confidence (-5552.536,5552.536) *interval extends into clinically important effects in* ***both*** *directions*
Imprecision judgment
Major concerns

Comparison Aspartame:Water
Evidence: mixed
NMA estimate: -1.080
95% Confidence interval:
Confidence (-5505.411,5503.251) *interval extends into clinically important effects in* ***both*** *directions*
Imprecision judgment
Major concerns

Comparison Saccharin:Water
Evidence: mixed
NMA estimate: -1.080
95% Confidence interval:
Confidence (-5911.792,5909.632) *interval extends into clinically important effects in* ***both*** *directions*
Imprecision judgment
Major concerns

Comparison Glucose:Saccharin
Evidence: indirect
NMA estimate: 6170.426
95% Confidence interval:
Confidence (-368.422,12709.274) *interval extends into clinically important effects*
Imprecision judgment
Some concerns

Comparison Glucose:Water
Evidence: indirect
NMA estimate: 6169.346
95% Confidence interval:
Confidence (-328.617,12667.309) *interval extends into clinically important effects*
Imprecision judgment
Some concerns

Relative effect estimates below -2250.0 and above 2250.0 pmol*min/L (minimal important difference; MID) were considered clinically important. Judgements for IMPRECISION were assigned by the CiNEMA algorithm (27, 28). There is no general consensus on a clinically important size difference for 2-hour blood insulin incremental area under the curve (iAUC). Health Canada considers a 20% reduction in glucose iAUC to be the minimum physiologically relevant difference as per "Guideline Concerning the Safety and Physiological Effects of Novel Fibre Sources and Food Products Containing Them" (29). Extrapolating the 20% threshold in glucose iAUC, we calculated the MID of insulin iAUC by taking 20% of 11250 pmol*mins/L which is the median of insulin iAUC in participants with type 2 diabetes (~6200 pmol*mins/L) and healthy participants (~16300 pmol*mins/L) after a 75-g oral glucose tolerance test (32-34).

Figure S22. *CINeMA output for the HETEROGENEITY domain of UNCOUPLING INTERVENTIONS evaluating the effect of non-nutritive sweetened beverages (NNS beverages) sweetened single or blends of non-nutritive sweeteners (NNS), water, and sugar-sweetened beverages (SSBs) sweetened with caloric sweeteners on postprandial blood INSULIN response in participants with TYPE 2 DIABETES*

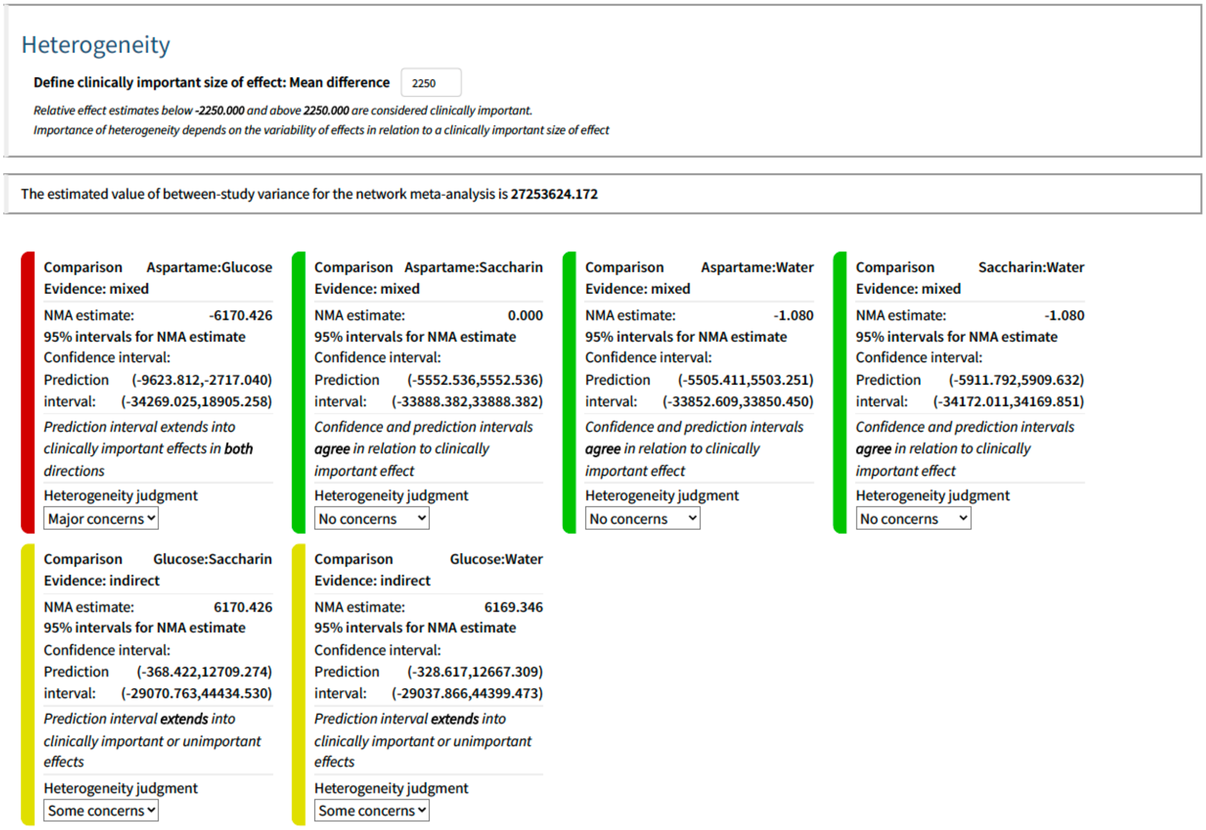

Judgements for were assigned by the CiNEMA algorithm (27, 28).

Figure S23. *CINeMA output for the INCOHERENCE domain of UNCOUPLING INTERVENTIONS evaluating the effect of non-nutritive sweetened beverages (NNS beverages) sweetened single or blends of non-nutritive sweeteners (NNS), water, and sugar-sweetened beverages (SSBs) sweetened with caloric sweeteners on postprandial blood INSULIN response in participants with TYPE 2 DIABETES*

Incoherence

Define clinically important size of effect: Mean difference 2250

*Relative effect estimates below **-2250.000** and above **2250.000** are considered clinically important.*
Importance of Incoherence depends on the variability of direct and indirect effects in relation to a clinically important size of effect

Global test based on a random-effects design-by-treatment interaction model
χ^2 statistic: 0.000 (0 degrees of freedom), P value: NA

Local tests: Separating indirect from direct evidence

Comparison	Evidence	Mean difference	Inconsistency measures	Incoherence judgment
Aspartame:Glucose	direct	Direct mean difference: -6170.426(-9623.812,-2717.040)	Not applicable	Major concerns
Aspartame:Saccharin	direct	Direct mean difference: 0.000(-5552.858,5552.858)	Not applicable	Major concerns
Aspartame:Water	direct	Direct mean difference: -1.080(-5505.724,5503.564)	Not applicable	Major concerns
Saccharin:Water	direct	Direct mean difference: -1.080(-5912.178,5910.018)	Not applicable	Major concerns
Glucose:Saccharin	indirect	Indirect mean difference: 6170.426(-368.422,12709.274)	Not applicable	Major concerns
Glucose:Water	indirect	Indirect mean difference: 6169.346(-328.617,12667.309)	Not applicable	Major concerns

Judgements for were assigned by the CiNEMA algorithm (27, 28).

Figure S24. *Network plot and meta-analysis of UNCOUPLING INTERVENTIONS evaluating the effect of non-nutritive sweetened beverages (NNS beverages) sweetened single or blends of non-nutritive sweeteners (NNS), water, and sugar-sweetened beverages (SSBs) sweetened with caloric sweeteners on postprandial blood GLP-1 response in HEALTHY participants*

Network Plot

4 Trials | 32 Participants
(Healthy)

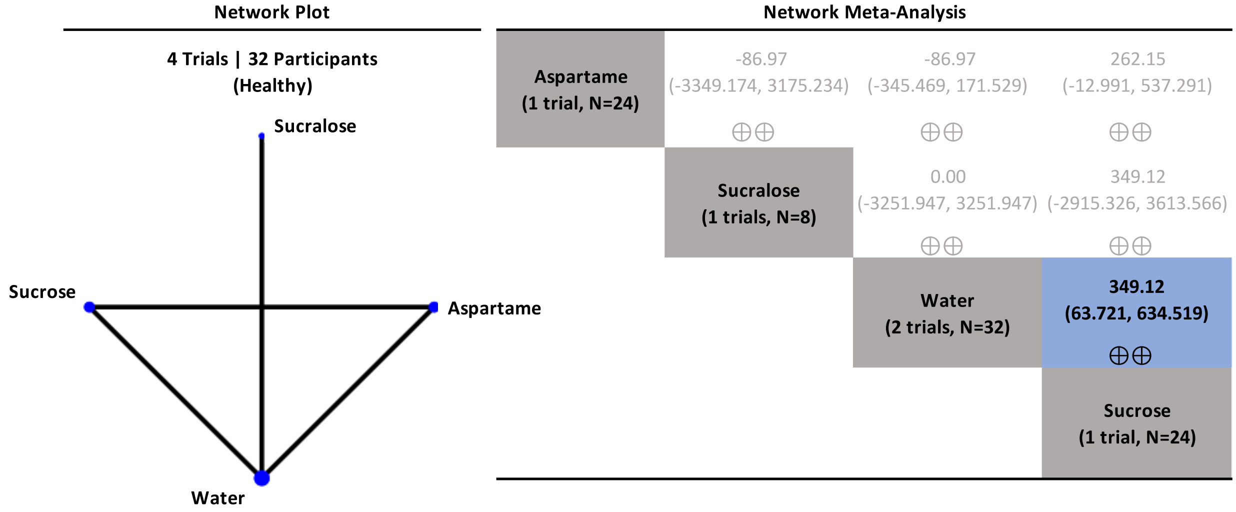

Network Meta-Analysis

**Aspartame (1 trial, N=24)**	-86.97 (-3349.174, 3175.234) ⊕⊕	-86.97 (-345.469, 171.529) ⊕⊕	262.15 (-12.991, 537.291) ⊕⊕
	**Sucralose (1 trials, N=8)**	0.00 (-3251.947, 3251.947) ⊕⊕	349.12 (-2915.326, 3613.566) ⊕⊕
		**Water (2 trials, N=32)**	**349.12 (63.721, 634.519)** ⊕⊕
			**Sucrose (1 trial, N=24)**

Network plot: the size of the blue nodes is proportional to the number of participants and the line width is proportional to the number of studies. Network table: treatments are grouped by treatment type (i.e., individual non-nutritive sweeteners (NNS), NNS blends, water and caloric sweeteners) and are reported in alphabetical order. Treatment estimates (pmol*min/L) are MDs and 95% CIs of the column-defining treatment compared with the row-defining treatment. MDs less than 0 favor the column-defining treatment. MDs greater than 0 favor the row-defining treatment. Significant results are bolded in black. Non-significant results are grey and not bolded. The minimally important difference (MID) for postprandial glucose response is 82 pmol*min/L. Trivial (significant) effects (<1 MID) or no effects have a white background; small important effects (≥1 MID) have a light blue background; moderate effects (≥2 MID) have a darker blue background; large effects (≥5 to <10 MID) have a purple background; very large effects (≥10 MID) have a black background. Confidence in the effect estimate is shown for each treatment comparison: high confidence ⊕⊕⊕⊕; moderate confidence ⊕⊕⊕; low confidence ⊕⊕; very low confidence ⊕. See **Supplementary Table 10 and Figure 25** for detailed assessments of the confidence in the effect estimate using the CINeMA framework.

Figure S25. *Risk of bias assessment of UNCOUPLING INTERVENTIONS evaluating the effect of non-nutritive sweetened beverages (NNS beverages) sweetened single or blends of non-nutritive sweeteners (NNS), water, and sugar-sweetened beverages (SSBs) sweetened with caloric sweeteners on postprandial blood GLP-1 response in HEALTHY participants*

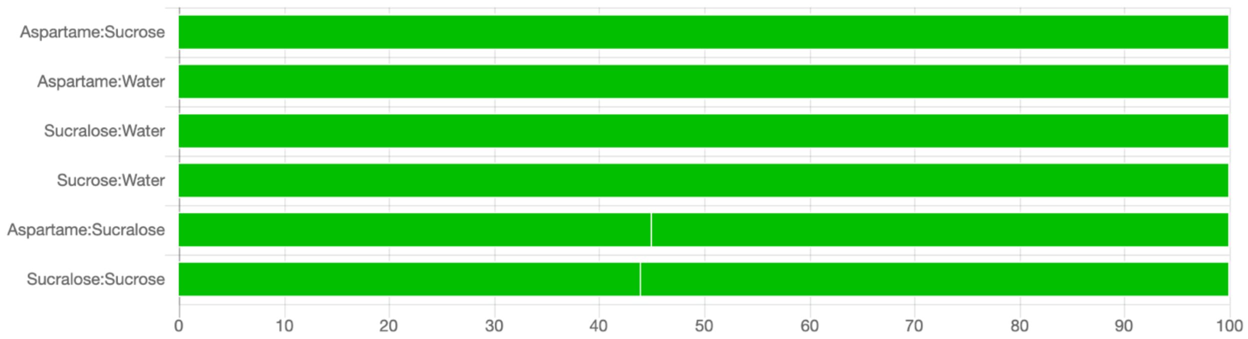

Green = no concerns; yellow = some concerns; red = major concerns

Figure S26. *Network plot and meta-analysis of UNCOUPLING INTERVENTIONS evaluating the effect of non-nutritive sweetened beverages (NNS beverages) sweetened single or blends of non-nutritive sweeteners (NNS), water, and sugar-sweetened beverages (SSBs) sweetened with caloric sweeteners on postprandial blood GIP response in HEALTHY participants*

Network Plot

1 trial | 24 Participants (Healthy)

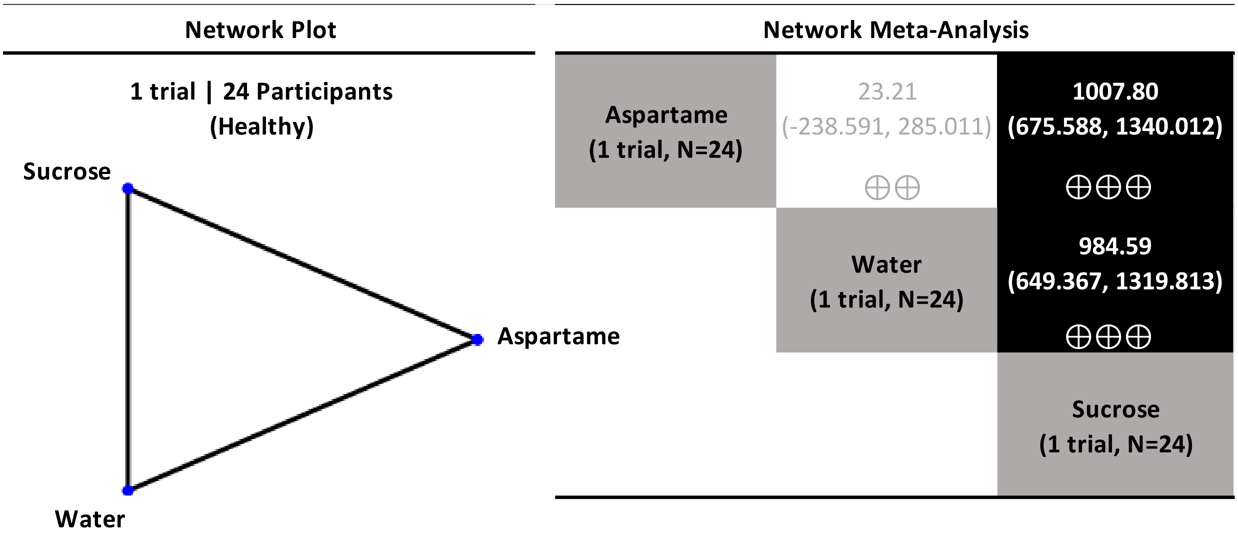

Network Meta-Analysis

**Aspartame (1 trial, N=24)**	23.21 (-238.591, 285.011) ⊕⊕	**1007.80 (675.588, 1340.012)** ⊕⊕⊕
	**Water (1 trial, N=24)**	**984.59 (649.367, 1319.813)** ⊕⊕⊕
		**Sucrose (1 trial, N=24)**

Network plot: the size of the blue nodes is proportional to the number of participants and the line width is proportional to the number of studies. Network table: treatments are grouped by treatment type (i.e., individual non-nutritive sweeteners (NNS), NNS blends, water and caloric sweeteners) and are reported in alphabetical order. Treatment estimates (pmol*min/L) are MDs and 95% CIs of the column-defining treatment compared with the row-defining treatment. MDs less than 0 favor the column-defining treatment. MDs greater than 0 favor the row-defining treatment. Significant results are bolded in white. Non-significant results are grey and not bolded. The minimally important difference (MID) for postprandial glucose response is 82 pmol*min/L. Trivial (significant) effects (<1 MID) or no effects have a white background; small important effects (≥1 MID) have a light blue background; moderate effects (≥2 MID) have a darker blue background; large effects (≥5 to <10 MID) have a purple background; very large effects (≥10 MID) have a black background. Confidence in the effect estimate is shown for each treatment comparison: high confidence ⊕⊕⊕⊕; moderate confidence ⊕⊕⊕; low confidence ⊕⊕; very low confidence ⊕. See **Supplementary Table 11 and Figure 27** for detailed assessments of the confidence in the effect estimate using the CINeMA framework.

Figure S27. *Risk of bias assessment of UNCOUPLING INTERVENTIONS evaluating the effect of non-nutritive sweetened beverages (NNS beverages) sweetened single or blends of non-nutritive sweeteners (NNS), water, and sugar-sweetened beverages (SSBs) sweetened with caloric sweeteners on postprandial blood GIP response in HEALTHY participants*

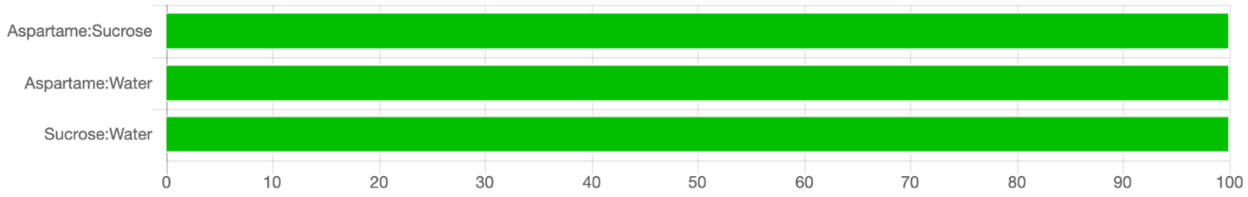

Green = no concerns; yellow = some concerns; red = major concerns

Figure S28. *Network plot and meta-analysis of UNCOUPLING INTERVENTIONS evaluating the effect of non-nutritive sweetened beverages (NNS beverages) sweetened single or blends of non-nutritive sweeteners (NNS), water, and sugar-sweetened beverages (SSBs) sweetened with caloric sweeteners on postprandial blood GHRELIN response in HEALTHY participants*

Network Plot

1 trial | 24 Participants (Healthy)

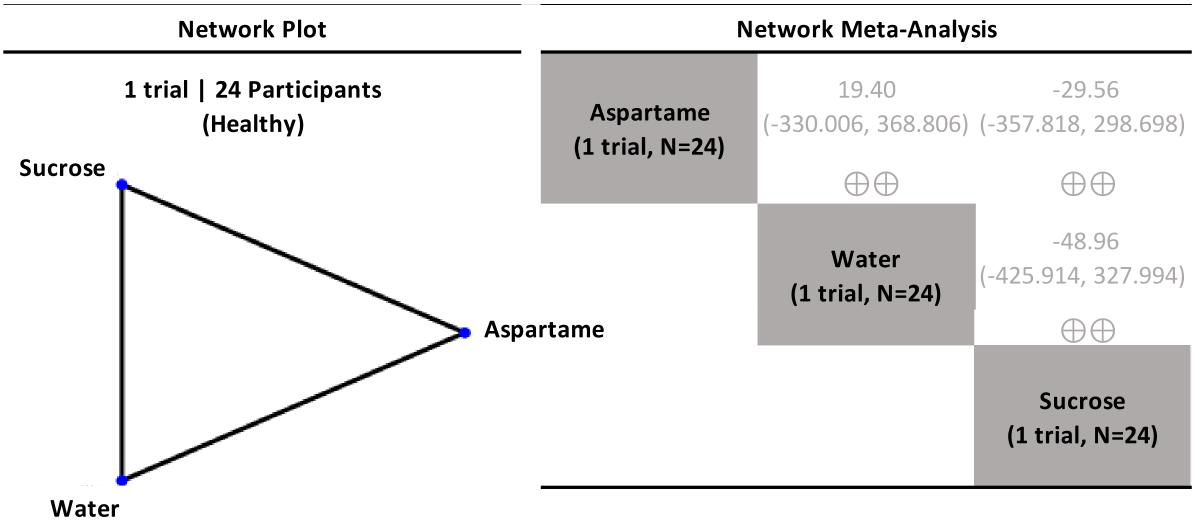

Network Meta-Analysis

**Aspartame (1 trial, N=24)**	19.40 (-330.006, 368.806) ⊕⊕	-29.56 (-357.818, 298.698) ⊕⊕
	**Water (1 trial, N=24)**	-48.96 (-425.914, 327.994) ⊕⊕
		**Sucrose (1 trial, N=24)**

Network plot: the size of the blue nodes is proportional to the number of participants and the line width is proportional to the number of studies. Network table: treatments are grouped by treatment type (i.e., individual non-nutritive sweeteners (NNS), NNS blends, water and caloric sweeteners) and are reported in alphabetical order. Treatment estimates (pmol*min/L) are MDs and 95% CIs of the column-defining treatment compared with the row-defining treatment. MDs less than 0 favor the column-defining treatment. MDs greater than 0 favor the row-defining treatment. Significant results are bolded in white. Non-significant results are grey and not bolded. The minimally important difference (MID) for postprandial glucose response is 10 pmol*min/L. Trivial (significant) effects (<1 MID) or no effects have a white background; small important effects (≥1 MID) have a light blue background; moderate effects (≥2 MID) have a darker blue background; large effects (≥5 to <10 MID) have a purple background; very large effects (≥10 MID) have a black background. Confidence in the effect estimate is shown for each treatment comparison: high confidence ⊕⊕⊕⊕; moderate confidence ⊕⊕⊕; low confidence ⊕⊕; very low confidence ⊕. See **Supplementary Table 12 and Figure 29** for detailed assessments of the confidence in the effect estimate using the CINeMA framework.

Figure S29. *Risk of bias assessment of UNCOUPLING INTERVENTIONS evaluating the effect of non-nutritive sweetened beverages (NNS beverages) sweetened single or blends of non-nutritive sweeteners (NNS), water, and sugar-sweetened beverages (SSBs) sweetened with caloric sweeteners on postprandial blood GHRELIN response in HEALTHY participants*

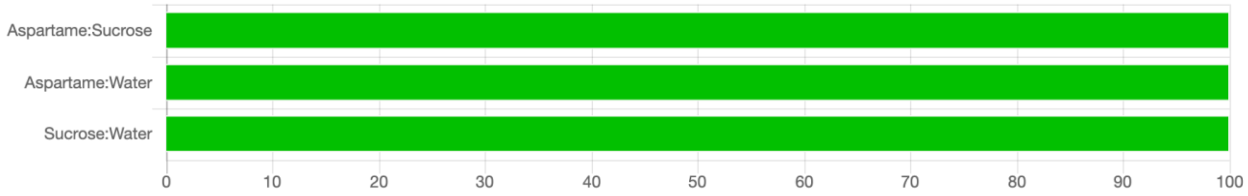

Green = no concerns; yellow = some concerns; red = major concerns

__Figure S30.__ Network plot and meta-analysis of UNCOUPLING INTERVENTIONS evaluating the effect of non-nutritive sweetened beverages (NNS beverages) sweetened single or blends of non-nutritive sweeteners (NNS), water, and sugar-sweetened beverages (SSBs) sweetened with caloric sweeteners on postprandial blood GLUCAGON response in HEALTHY participants

Network Plot

2 Trials | 17 Participants (Healthy)

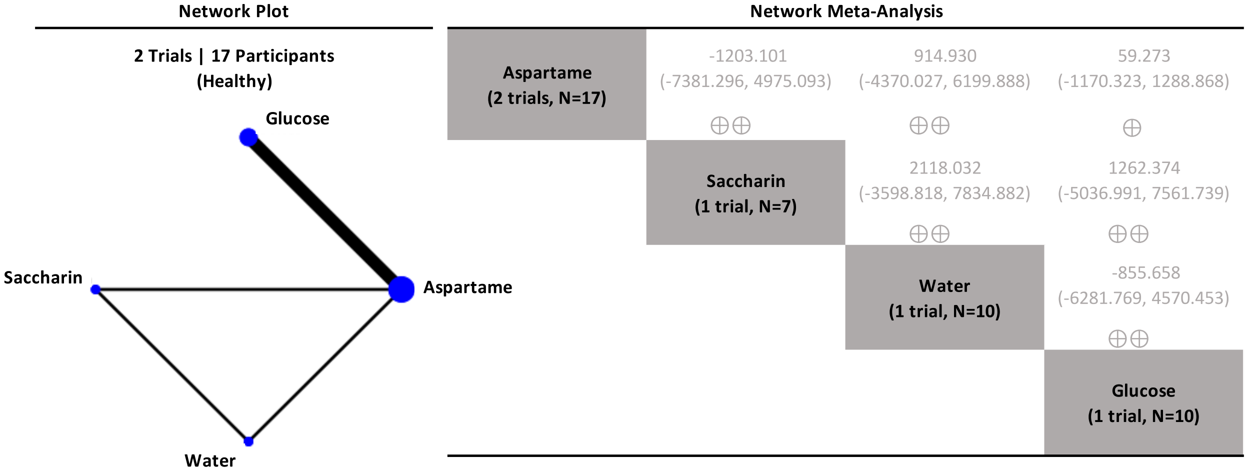

Network Meta-Analysis

**Aspartame (2 trials, N=17)**	-1203.101 (-7381.296, 4975.093) ⊕⊕	914.930 (-4370.027, 6199.888) ⊕⊕	59.273 (-1170.323, 1288.868) ⊕
	**Saccharin (1 trial, N=7)**	2118.032 (-3598.818, 7834.882) ⊕⊕	1262.374 (-5036.991, 7561.739) ⊕⊕
		**Water (1 trial, N=10)**	-855.658 (-6281.769, 4570.453) ⊕⊕
			**Glucose (1 trial, N=10)**

Network plot: the size of the blue nodes is proportional to the number of participants and the line width is proportional to the number of studies. Network table: treatments are grouped by treatment type (i.e., individual non-nutritive sweeteners (NNS), NNS blends, water and caloric sweeteners) and are reported in alphabetical order. Treatment estimates (pmol*min/L) are MDs and 95% CIs of the column-defining treatment compared with the row-defining treatment. MDs less than 0 favor the column-defining treatment. MDs greater than 0 favor the row-defining treatment. Significant results are bolded in white. Non-significant results are grey and not bolded. The minimally important difference (MID) for postprandial glucose response is 205 pmol*min/L. Trivial (significant) effects (<1 MID) or no effects have a white background; small important effects (≥1 MID) have a light blue background; moderate effects (≥2 MID) have a darker blue background; large effects (≥5 to <10 MID) have a purple background; very large effects (≥10 MID) have a black background. Confidence in the effect estimate is shown for each treatment comparison: high confidence ⊕⊕⊕⊕; moderate confidence ⊕⊕⊕; low confidence ⊕⊕; very low confidence ⊕. See **Supplementary Table 13 and Figure 31** for detailed assessments of the confidence in the effect estimate using the CINeMA framework.

Figure S31. *Risk of bias assessment of UNCOUPLING INTERVENTIONS evaluating the effect of non-nutritive sweetened beverages (NNS beverages) sweetened single or blends of non-nutritive sweeteners (NNS), water, and sugar-sweetened beverages (SSBs) sweetened with caloric sweeteners on postprandial blood GLUCAGON response in HEALTHY participants*

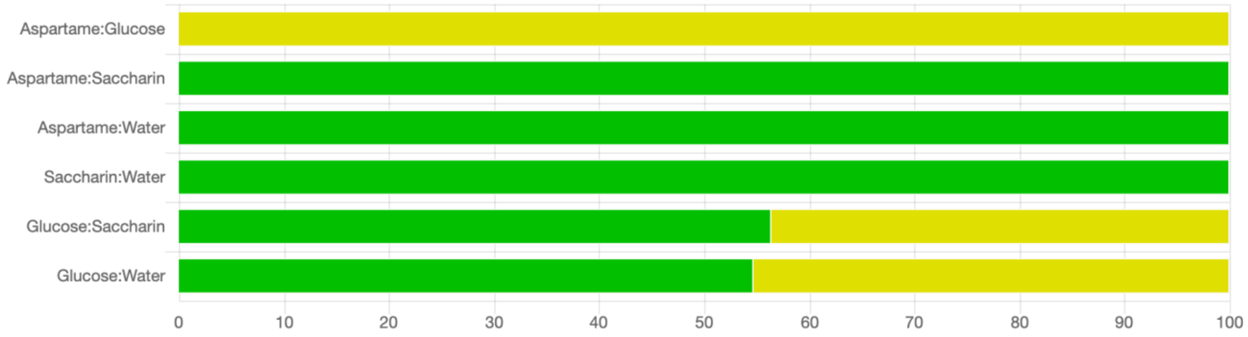

Green = no concerns; yellow = some concerns; red = major concerns

Figure S32. *Network plot and meta-analysis of UNCOUPLING INTERVENTIONS evaluating the effect of non-nutritive sweetened beverages (NNS beverages) sweetened single or blends of non-nutritive sweeteners (NNS), water, and sugar-sweetened beverages (SSBs) sweetened with caloric sweeteners on postprandial blood GLUCAGON response in participants with TYPE 2 DIABETES*

Network Plot

4 Trials | 32 Participants (Type 2 Diabetes)

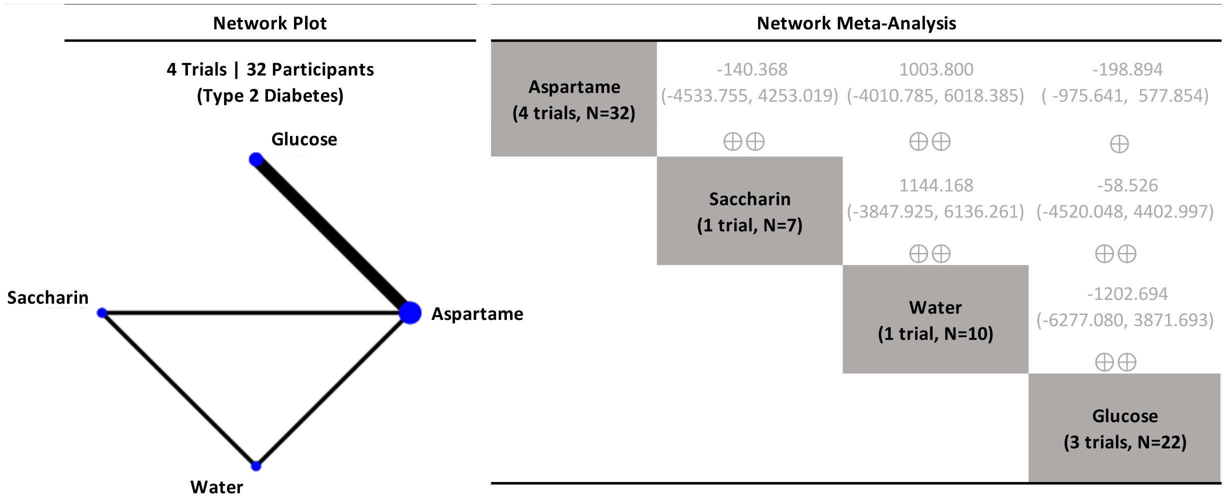

Network Meta-Analysis

**Aspartame (4 trials, N=32)**	-140.368 (-4533.755, 4253.019) ⊕⊕	1003.800 (-4010.785, 6018.385) ⊕⊕	-198.894 (-975.641, 577.854) ⊕
	**Saccharin (1 trial, N=7)**	1144.168 (-3847.925, 6136.261) ⊕⊕	-58.526 (-4520.048, 4402.997) ⊕⊕
		**Water (1 trial, N=10)**	-1202.694 (-6277.080, 3871.693) ⊕⊕
			**Glucose (3 trials, N=22)**

Network plot: the size of the blue nodes is proportional to the number of participants and the line width is proportional to the number of studies. Network table: treatments are grouped by treatment type (i.e., individual non-nutritive sweeteners (NNS), NNS blends, water and caloric sweeteners) and are reported in alphabetical order. Treatment estimates (pmol*min/L) are MDs and 95% CIs of the column-defining treatment compared with the row-defining treatment. MDs less than 0 favor the column-defining treatment. MDs greater than 0 favor the row-defining treatment. Significant results are bolded in white. Non-significant results are grey and not bolded. The minimally important difference (MID) for postprandial glucose response is 205 pmol*min/L. Trivial (significant) effects (<1 MID) or no effects have a white background; small important effects (≥1 MID) have a light blue background; moderate effects (≥2 MID) have a darker blue background; large effects (≥5 to <10 MID) have a purple background; very large effects (≥10 MID) have a black background. Confidence in the effect estimate is shown for each treatment comparison: high confidence ⊕⊕⊕⊕; moderate confidence ⊕⊕⊕; low confidence ⊕⊕; very low confidence ⊕. See **Supplementary Table 14 and Figure 33** for detailed assessments of the confidence in the effect estimate using the CINeMA framework.

Figure S33. *Risk of bias assessment of UNCOUPLING INTERVENTIONS evaluating the effect of non-nutritive sweetened beverages (NNS beverages) sweetened single or blends of non-nutritive sweeteners (NNS), water, and sugar-sweetened beverages (SSBs) sweetened with caloric sweeteners on postprandial blood GLUCAGON response in participants with TYPE 2 DIABETES*

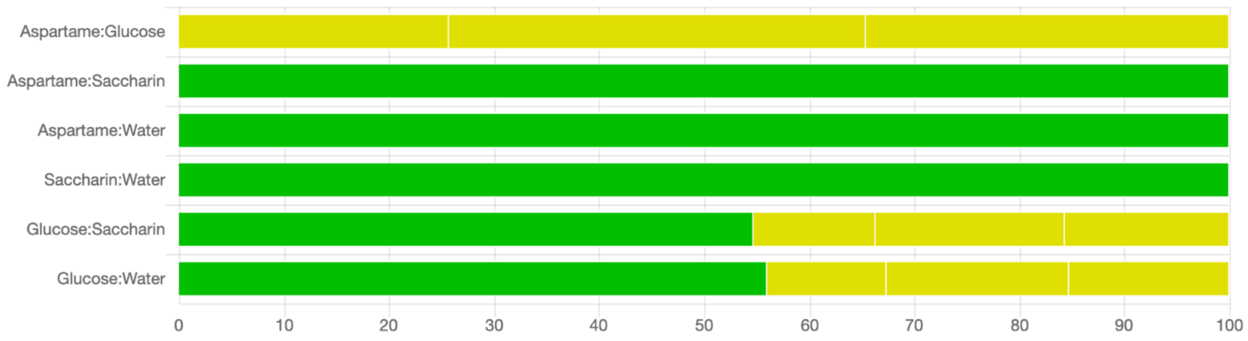

Green = no concerns; yellow = some concerns; red = major concerns

Figure S34. *Risk of bias assessment of COUPLING INTERVENTIONS evaluating the effect of non-nutritive sweetened beverages (NNS beverages) sweetened single or blends of non-nutritive sweeteners (NNS) and controls on postprandial* blood *GLUCOSE response in HEALTHY participants.*

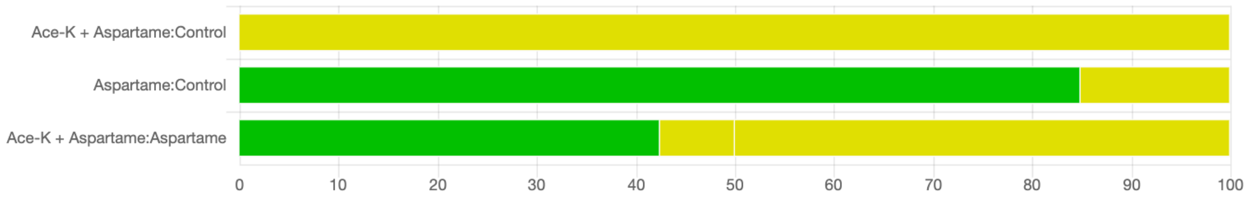

Green = no concerns; yellow = some concerns; red = major concerns

Figure S35. *Network plot and meta-analysis of COUPLING INTERVENTIONS evaluating the effect of non-nutritive sweetened beverages (NNS beverages) sweetened single or blends of non-nutritive sweeteners (NNS) and controls on postprandial blood INSULIN response in HEALTHY participants.*

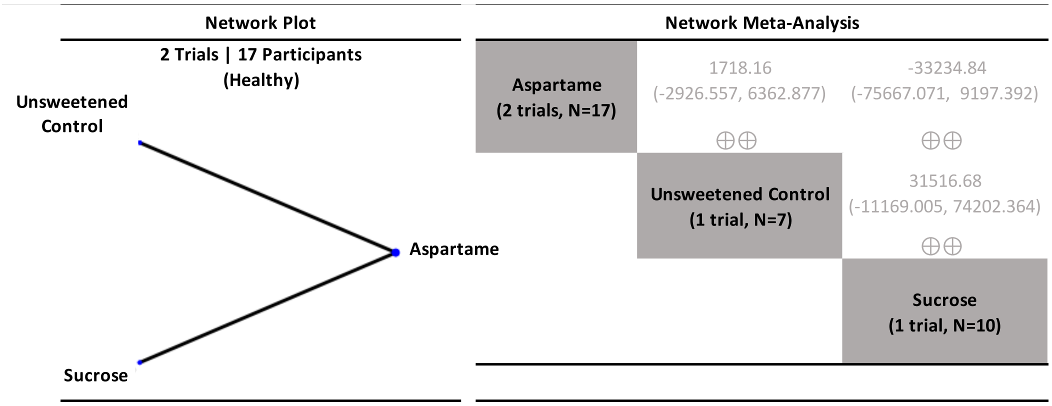

Network Meta-Analysis

**Aspartame (2 trials, N=17)**	1718.16 (-2926.557, 6362.877) ⨁⨁	-33234.84 (-75667.071, 9197.392) ⨁⨁
	**Unsweetened Control (1 trial, N=7)**	31516.68 (-11169.005, 74202.364) ⨁⨁
		**Sucrose (1 trial, N=10)**

Network plot: the size of the blue nodes is proportional to the number of participants and the line width is proportional to the number of studies. Network table: treatments are grouped by treatment type (i.e., individual non-nutritive sweeteners (NNS), NNS blends, water and caloric sweeteners) and are reported in alphabetical order. Treatment estimates (mmol*min/L) are MDs and 95% CIs of the column-defining treatment compared with the row-defining treatment. MDs less than 0 favor the column-defining treatment. MDs greater than 0 favor the row-defining treatment. Significant results are bolded in black. Non-significant results are grey and not bolded. The minimally important difference (MID) for postprandial glucose response is 2250 pmol*min/L. Trivial (significant) effects (<1 MID) or no effects have a white background; small important effects ($\geq$1 MID) have a light blue background; moderate effects ($\geq$2 MID) have a darker blue background; large effects ($\geq$5 to <10 MID) have a purple background; very large effects ($\geq$10 MID) have a black background. Confidence in the effect estimate is shown for each treatment comparison: high confidence ⨁⨁⨁⨁; moderate confidence ⨁⨁⨁; low confidence ⨁⨁; very low confidence ⨁. See **Supplementary Table 16 and Figures 36-40** for detailed assessments of the confidence in the effect estimate using the CINeMA framework.

Figure S36. *Risk of bias assessment of COUPLING INTERVENTIONS evaluating the effect of non-nutritive sweetened beverages (NNS beverages) sweetened single or blends of non-nutritive sweeteners (NNS) and controls on postprandial blood INSULIN response in HEALTHY participants.*

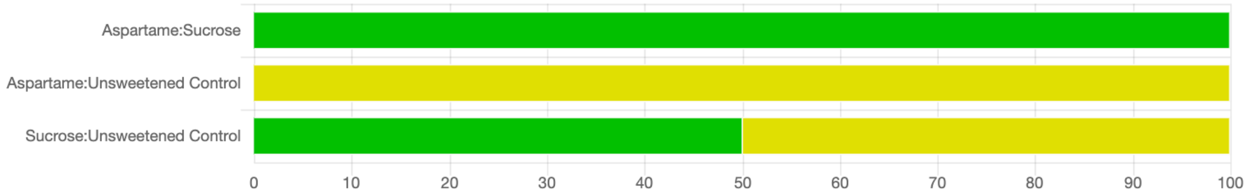

Green = no concerns; yellow = some concerns; red = major concerns

Figure S37. *Risk of bias assessment of DELAYED COUPLING INTERVENTIONS evaluating the effect of non-nutritive sweetened beverages (NNS beverages) sweetened single or blends of non-nutritive sweeteners (NNS), water, and sugar-sweetened beverages (SSBs) sweetened with caloric sweeteners on postprandial blood GLUCOSE response in HEALTHY participants*

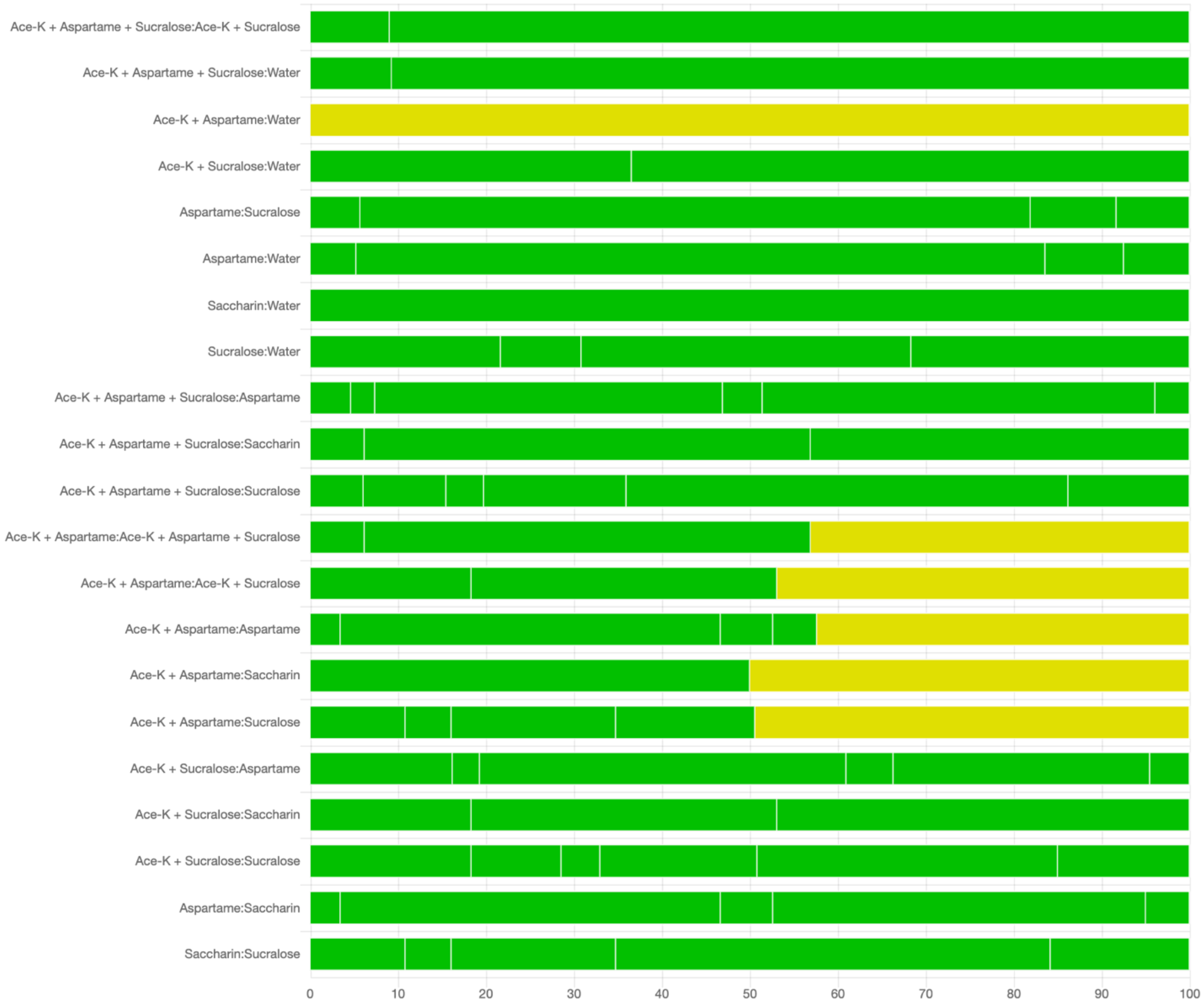

Green = no concerns; yellow = some concerns; red = major concerns

Figure S38. *CINeMA output for the IMPRECISION domain of DELAYED COUPLING INTERVENTIONS evaluating the effect of non-nutritive sweetened beverages (NNS beverages) sweetened single or blends of non-nutritive sweeteners (NNS), water, and sugar-sweetened beverages (SSBs) sweetened with caloric sweeteners on postprandial blood GLUCOSE response in HEALTHY participants*

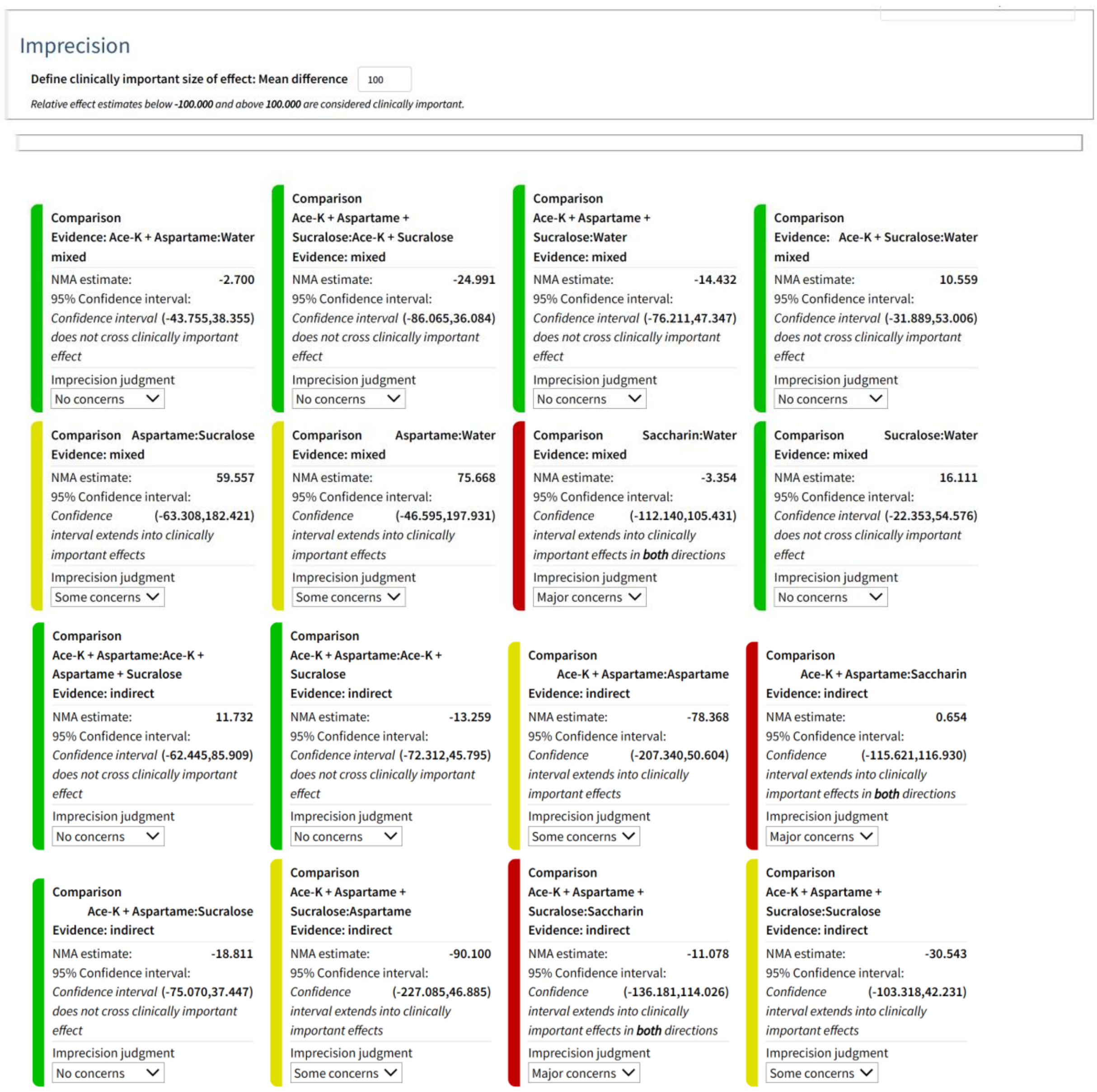

(continued on next page)

Comparison **Ace-K + Sucralose:Aspartame**
Evidence: indirect
NMA estimate: **-65.109**
95% Confidence interval: **(-194.531,64.312)**
Confidence interval extends into clinically important effects
Imprecision judgment
Some concerns

Comparison **Ace-K + Sucralose:Saccharin**
Evidence: indirect
NMA estimate: **13.913**
95% Confidence interval: **(-102.861,130.687)**
Confidence interval extends into clinically important effects in ***both*** *directions*
Imprecision judgment
Major concerns

Comparison **Ace-K + Sucralose:Sucralose**
Evidence: indirect
NMA estimate: **-5.553**
95% Confidence interval: **(-62.835,51.730)**
Confidence interval does not cross clinically important effect
Imprecision judgment
No concerns

Comparison **Aspartame:Saccharin** [illegible] Updated
Evidence: indirect
NMA estimate: **79.022**
95% Confidence interval: **(-84.631,242.676)**
Confidence interval extends into clinically important effects
Imprecision judgment
Some concerns

Comparison **Saccharin:Sucralose**
Evidence: indirect
NMA estimate: **-19.466**
95% Confidence interval: **(-134.851,95.920)**
Confidence interval extends into clinically important effects
Imprecision judgment
Some concerns

Relative effect estimates below -100.0 and above 100.0 mmol*min/L (minimal important difference; MID) were considered clinically important. Judgements for IMPRECISION were assigned by the CiNEMA algorithm (27, 28). There is no general consensus on a clinically important size difference for 2-hour blood glucose incremental area under the curve (iAUC). Health Canada considers a 20% reduction in glucose iAUC to be the minimum physiologically relevant difference as per “Guideline Concerning the Safety and Physiological Effects of Novel Fibre Sources and Food Products Containing Them” (29). Thus, we calculated the MID by taking 20% of 500 mmol*mins/L which is the median of glucose iAUC in healthy participants (~300 mmol*mins/L) and those with type 2 diabetes (~700 mmol*mins/L) after a 75-g oral glucose tolerance test (32-34).

Figure S39. *CINeMA output for the HETEROGENEITY domain of DELAYED COUPLING INTERVENTIONS evaluating the effect of non-nutritive sweetened beverages (NNS beverages) sweetened single or blends of non-nutritive sweeteners (NNS), water, and sugar-sweetened beverages (SSBs) sweetened with caloric sweeteners on postprandial blood GLUCOSE response in HEALTHY participants*

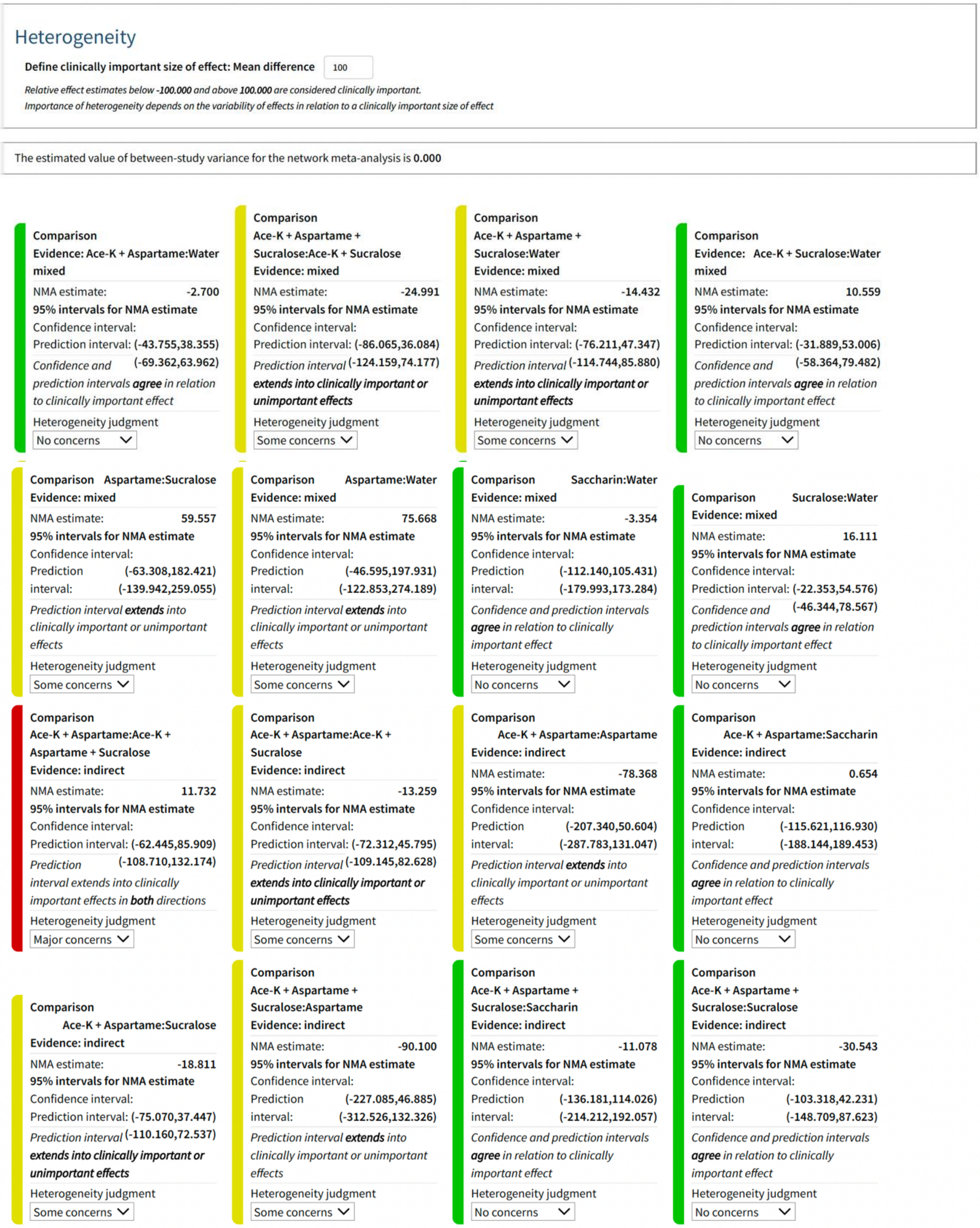

Comparison **Ace-K + Sucralose:Aspartame**
Evidence: indirect

NMA estimate: **-65.109**

95% intervals for NMA estimate

Confidence interval: **(-194.531,64.312)**

Prediction interval: **(-275.255,145.036)**

Prediction interval ***extends*** *into clinically important or unimportant effects*

Heterogeneity judgment

Some concerns

Comparison **Ace-K + Sucralose:Saccharin**
Evidence: indirect

NMA estimate: **13.913**

95% intervals for NMA estimate

Confidence interval: **(-102.861,130.687)**

Prediction interval: **(-175.696,203.522)**

Confidence and prediction intervals ***agree*** *in relation to clinically important effect*

Heterogeneity judgment

No concerns

Comparison **Ace-K + Sucralose:Sucralose**
Evidence: indirect

NMA estimate: **-5.553**

95% intervals for NMA estimate

Confidence interval: **(-62.835,51.730)**

Prediction interval: **(-98.564,87.458)**

Confidence and prediction intervals ***agree*** *in relation to clinically important effect*

Heterogeneity judgment

No concerns

Comparison **Aspartame:Saccharin**
Evidence: indirect

NMA estimate: **79.022**

95% intervals for NMA estimate

Confidence interval: **(-84.631,242.676)**

Prediction interval: **(-186.707,344.752)**

Prediction interval ***extends*** *into clinically important or unimportant effects*

Heterogeneity judgment

Some concerns

Comparison **Saccharin:Sucralose**
Evidence: indirect

NMA estimate: **-19.466**

95% intervals for NMA estimate

Confidence interval: **(-134.851,95.920)**

Prediction interval: **(-206.821,167.889)**

Prediction interval ***extends*** *into clinically important or unimportant effects*

Heterogeneity judgment

Some concerns

Figure S40. *CINeMA output for the INCOHERENCE domain of DELAYED COUPLING INTERVENTIONS evaluating the effect of non-nutritive sweetened beverages (NNS beverages) sweetened single or blends of non-nutritive sweeteners (NNS), water, and sugar-sweetened beverages (SSBs) sweetened with caloric sweeteners on postprandial blood GLUCOSE response in HEALTHY participants*

Incoherence

Define clinically important size of effect: Mean difference 100

*Relative effect estimates below **-100.000** and above **100.000** are considered clinically important.*
Importance of Incoherence depends on the variability of direct and indirect effects in relation to a clinically important size of effect

Global test based on a random-effects design-by-treatment interaction model
χ^2 statistic: 1.617 (2 degrees of freedom), P value: 0.445

Local tests: Separating indirect from direct evidence

Comparison	Ace-K + Aspartame:Water
Evidence: direct	
Direct mean difference:	-2.700(-43.755,38.355)
Inconsistency measures: Not applicable	
Incoherence judgment	No concerns

Comparison	Ace-K + Aspartame + Sucralose:Ace-K + Sucralose
Evidence: mixed	
NMA mean difference:	-24.991(-86.065,36.084)
Direct mean difference:	-30.486(-93.621,32.649)
Indirect mean difference:	55.086(-185.914,296.086)
Inconsistency measures	
Difference of mean differences:	-85.572(-334.705,163.561)
P value:	0.501
Incoherence judgment	No concerns

Comparison	Ace-K + Aspartame + Sucralose:Water
Evidence: mixed	
NMA mean difference:	-14.432(-76.211,47.347)
Direct mean difference:	-8.382(-72.623,55.859)
Indirect mean difference:	-88.855(-314.163,136.454)
Inconsistency measures	
Difference of mean differences:	80.473(-153.815,314.761)
P value:	0.501
Incoherence judgment	No concerns

Comparison	Ace-K + Sucralose:Water
Evidence: direct	
Direct mean difference:	10.559(-31.889,53.006)
Inconsistency measures: Not applicable	
Incoherence judgment	No concerns

Comparison	Aspartame:Sucralose
Evidence: mixed	
NMA mean difference:	59.557(-63.308,182.421)
Direct mean difference:	98.900(-43.237,241.037)
Indirect mean difference:	-56.737(-301.107,187.633)
Inconsistency measures	
Difference of mean differences:	155.637(-127.063,438.337)
P value:	0.281
Incoherence judgment	No concerns

Comparison	Aspartame:Water
Evidence: mixed	
NMA mean difference:	75.668(-46.595,197.931)
Direct mean difference:	43.460(-92.079,178.999)
Indirect mean difference:	216.337(-66.921,499.595)
Inconsistency measures	
Difference of mean differences:	-172.877(-486.893,141.138)
P value:	0.281
Incoherence judgment	No concerns

Comparison	Saccharin:Water
Evidence: direct	
Direct mean difference:	-3.354(-112.140,105.431)
Inconsistency measures: Not applicable	
Incoherence judgment	No concerns

Comparison	Sucralose:Water
Evidence: direct	
Direct mean difference:	16.111(-22.353,54.576)
Inconsistency measures: Not applicable	
Incoherence judgment	No concerns

Comparison	Ace-K + Aspartame:Ace-K + Aspartame + Sucralose
Evidence: indirect	
Indirect mean difference:	11.732(-62.445,85.909)
Inconsistency measures: Not applicable	
Incoherence judgment	No concerns

Comparison	Ace-K + Aspartame:Ace-K + Sucralose
Evidence: indirect	
Indirect mean difference:	-13.259(-72.312,45.795)
Inconsistency measures: Not applicable	
Incoherence judgment	No concerns

Comparison	Ace-K + Aspartame:Aspartame
Evidence: indirect	
Indirect mean difference:	-78.368(-207.340,50.604)
Inconsistency measures: Not applicable	
Incoherence judgment	No concerns

Comparison	Ace-K + Aspartame:Saccharin
Evidence: indirect	
Indirect mean difference:	0.654(-115.621,116.930)
Inconsistency measures: Not applicable	
Incoherence judgment	No concerns

Comparison	Ace-K + Aspartame:Sucralose
Evidence: indirect	
Indirect mean difference:	-18.811(-75.070,37.447)
Inconsistency measures: Not applicable	
Incoherence judgment	No concerns

Comparison	Ace-K + Aspartame + Sucralose:Aspartame
Evidence: indirect	
Indirect mean difference:	-90.100(-227.085,46.885)
Inconsistency measures: Not applicable	
Incoherence judgment	No concerns

Comparison	Ace-K + Aspartame + Sucralose:Saccharin
Evidence: indirect	
Indirect mean difference:	-11.078(-136.181,114.026)
Inconsistency measures: Not applicable	
Incoherence judgment	No concerns

Comparison	Ace-K + Aspartame + Sucralose:Sucralose
Evidence: indirect	
Indirect mean difference:	-30.543(-103.318,42.231)
Inconsistency measures: Not applicable	
Incoherence judgment	No concerns

Comparison	Ace-K + Sucralose:Aspartame
Evidence: indirect	
Indirect mean difference:	-65.109(-194.531,64.312)
Inconsistency measures: Not applicable	
Incoherence judgment	No concerns

Comparison	Ace-K + Sucralose:Saccharin
Evidence: indirect	
Indirect mean difference:	13.913(-102.861,130.687)
Inconsistency measures: Not applicable	
Incoherence judgment	No concerns

Comparison	Ace-K + Sucralose:Sucralose
Evidence: indirect	
Indirect mean difference:	-5.553(-62.835,51.730)
Inconsistency measures: Not applicable	
Incoherence judgment	No concerns

Comparison	Aspartame:Saccharin
Evidence: indirect	
Indirect mean difference:	79.022(-84.631,242.676)
Inconsistency measures: Not applicable	
Incoherence judgment	No concerns

Comparison	Saccharin:Sucralose
Evidence: indirect	
Indirect mean difference:	-19.466(-134.851,95.920)
Inconsistency measures: Not applicable	
Incoherence judgment	No concerns

Figure S41. *Risk of bias assessment of DELAYED COUPLING INTERVENTIONS evaluating the effect of non-nutritive sweetened beverages (NNS beverages) sweetened single or blends of non-nutritive sweeteners (NNS), water, and sugar-sweetened beverages (SSBs) sweetened with caloric sweeteners on postprandial blood GLUCOSE response in participants with TYPE 2 DIABETES*

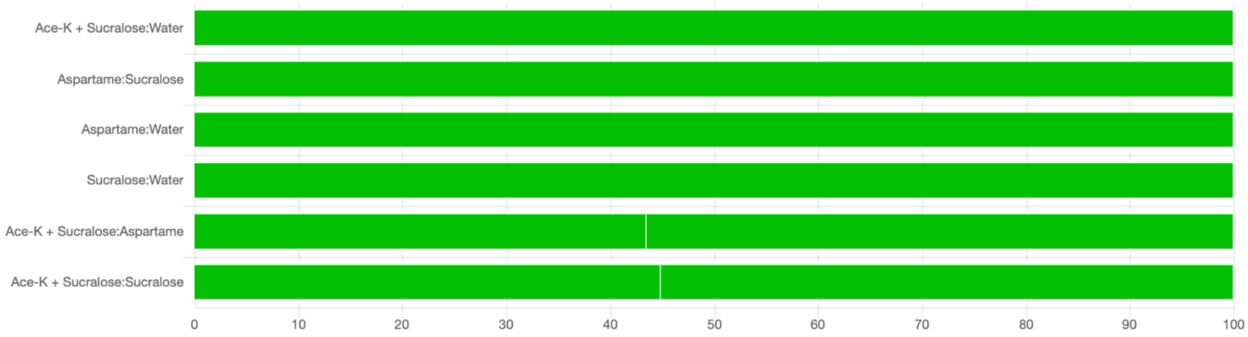

Green = no concerns; yellow = some concerns; red = major concerns

Figure S42. *Network plot and meta-analysis of DELAYED COUPLING INTERVENTIONS evaluating the effect of non-nutritive sweetened beverages (NNS beverages) sweetened single or blends of non-nutritive sweeteners (NNS), water, and sugar-sweetened beverages (SSBs) sweetened with caloric sweeteners on postprandial blood INSULIN response in HEALTHY participants*

Network Plot

7 Trials | 129 Participants (Healthy)

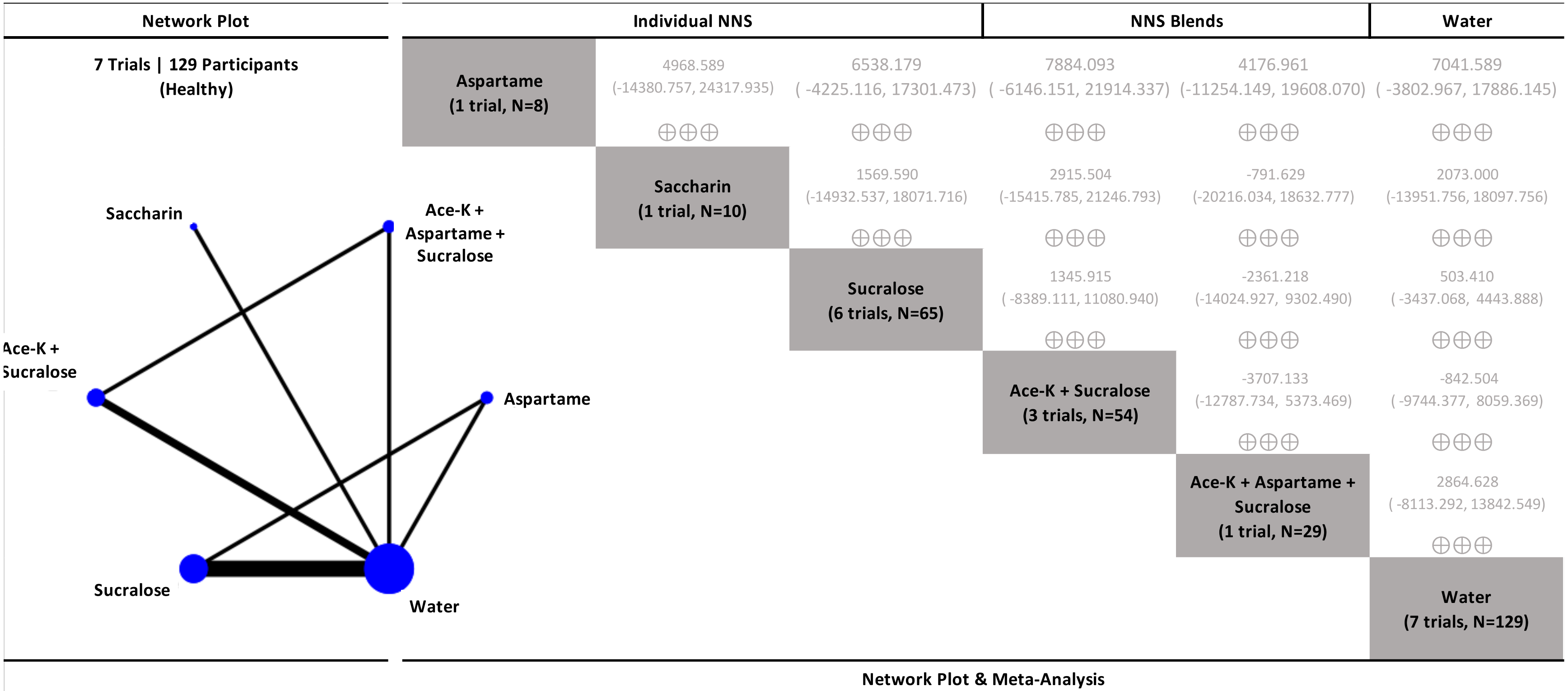

	Individual NNS			NNS Blends		Water
	**Aspartame (1 trial, N=8)**	4968.589 (-14380.757, 24317.935) ⊕⊕⊕	6538.179 (-4225.116, 17301.473) ⊕⊕⊕	7884.093 (-6146.151, 21914.337) ⊕⊕⊕	4176.961 (-11254.149, 19608.070) ⊕⊕⊕	7041.589 (-3802.967, 17886.145) ⊕⊕⊕
		**Saccharin (1 trial, N=10)**	1569.590 (-14932.537, 18071.716) ⊕⊕⊕	2915.504 (-15415.785, 21246.793) ⊕⊕⊕	-791.629 (-20216.034, 18632.777) ⊕⊕⊕	2073.000 (-13951.756, 18097.756) ⊕⊕⊕
			**Sucralose (6 trials, N=65)**	1345.915 (-8389.111, 11080.940) ⊕⊕⊕	-2361.218 (-14024.927, 9302.490) ⊕⊕⊕	503.410 (-3437.068, 4443.888) ⊕⊕⊕
				**Ace-K + Sucralose (3 trials, N=54)**	-3707.133 (-12787.734, 5373.469) ⊕⊕⊕	-842.504 (-9744.377, 8059.369) ⊕⊕⊕
					**Ace-K + Aspartame + Sucralose (1 trial, N=29)**	2864.628 (-8113.292, 13842.549) ⊕⊕⊕
						**Water (7 trials, N=129)**

Network Plot & Meta-Analysis

Network plot: the size of the blue nodes is proportional to the number of participants and the line width is proportional to the number of studies. Network table: treatments are grouped by treatment type (i.e., individual non-nutritive sweeteners (NNS), NNS blends, water and caloric sweeteners) and are reported in alphabetical order. Treatment estimates (pmol*min/L) are MDs and 95% CIs of the column-defining treatment compared with the row-defining treatment. MDs less than 0 favor the column-defining treatment. MDs greater than 0 favor the row-defining treatment. Significant results are bolded in black. Non-significant results are grey and not bolded. The minimally important difference (MID) for postprandial glucose response is 2250 pmol*min/L. Trivial

(significant) effects (<1 MID) or no effects have a white background; small important effects (≥1 MID) have a light blue background; moderate effects (≥2 MID) have a darker blue background; large effects (≥5 to <10 MID) have a purple background; very large effects (≥10 MID) have a black background. Confidence in the effect estimate is shown for each treatment comparison: high confidence ⨁⨁⨁⨁; moderate confidence ⨁⨁⨁; low confidence ⨁⨁; very low confidence ⨁. See **Supplementary Table 19 and Figures 43-46** for detailed assessments of the confidence in the effect estimate using the CINeMA framework.

Figure S43. *Risk of bias assessment of DELAYED COUPLING INTERVENTIONS evaluating the effect of non-nutritive sweetened beverages (NNS beverages) sweetened single or blends of non-nutritive sweeteners (NNS), water, and sugar-sweetened beverages (SSBs) sweetened with caloric sweeteners on postprandial blood INSULIN response in HEALTHY participants*

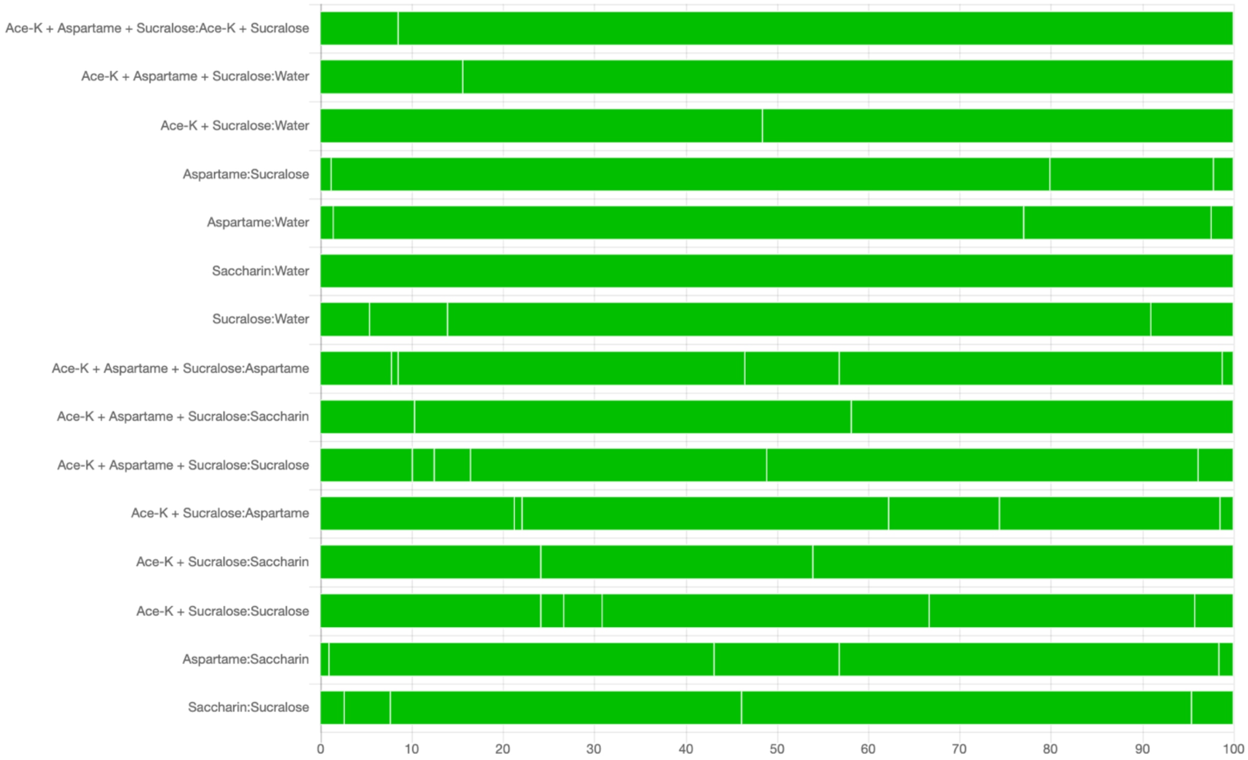

Green = no concerns; yellow = some concerns; red = major concerns

Figure S44. *CINeMA output for the IMPRECISION domain of DELAYED COUPLING INTERVENTIONS evaluating the effect of non-nutritive sweetened beverages (NNS beverages) sweetened single or blends of non-nutritive sweeteners (NNS), water, and sugar-sweetened beverages (SSBs) sweetened with caloric sweeteners on postprandial blood INSULIN response in HEALTHY participants*

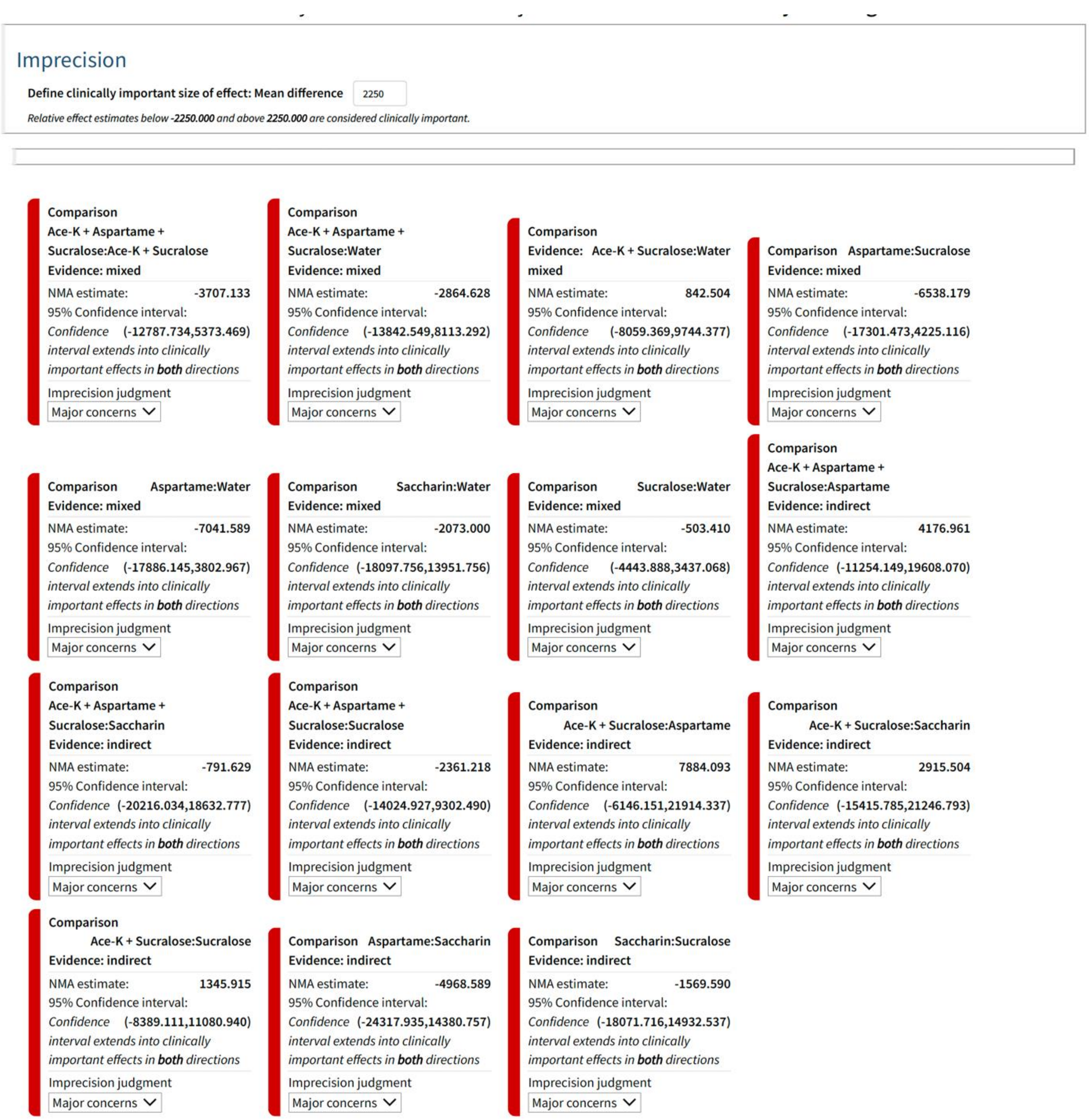

Relative effect estimates below -2250.0 and above 2250.0 pmol*min/L (minimal important difference; MID) were considered clinically important. Judgements for IMPRECISION were assigned by the CiNEMA algorithm (27, 28). There is no general consensus on a clinically important size difference for 2-hour blood insulin incremental area under the curve (iAUC). Health Canada considers a 20% reduction in glucose iAUC to be the minimum physiologically relevant difference as per “Guideline Concerning the Safety and Physiological Effects of Novel Fibre Sources and Food Products Containing Them” (29). Extrapolating the 20% threshold in glucose iAUC, we calculated

the MID of insulin iAUC by taking 20% of 11250 pmol*mins/L which is the median of insulin iAUC in participants with type 2 diabetes (~6200 pmol*mins/L) and healthy participants (~16300 pmol*mins/L) after a 75-g oral glucose tolerance test (32-34).

Figure S45. *CINeMA output for the HETEROGENEITY domain of DELAYED COUPLING INTERVENTIONS evaluating the effect of non-nutritive sweetened beverages (NNS beverages) sweetened single or blends of non-nutritive sweeteners (NNS), water, and sugar-sweetened beverages (SSBs) sweetened with caloric sweeteners on postprandial blood INSULIN response in HEALTHY participants*

Heterogeneity

Define clinically important size of effect: Mean difference 2250

*Relative effect estimates below **-2250.000** and above **2250.000** are considered clinically important.*
Importance of heterogeneity depends on the variability of effects in relation to a clinically important size of effect

The estimated value of between-study variance for the network meta-analysis is 0.000

Comparison Ace-K + Aspartame + Sucralose:Ace-K + Sucralose
Evidence: mixed
NMA estimate: -3707.133
95% intervals for NMA estimate
Confidence interval: (-12787.734,5373.469)
Prediction interval: (-18451.550,11037.285)
*Confidence and prediction intervals **agree** in relation to clinically important effect*
Heterogeneity judgment: No concerns

Comparison Ace-K + Aspartame + Sucralose:Water
Evidence: mixed
NMA estimate: -2864.628
95% intervals for NMA estimate
Confidence interval: (-13842.549,8113.292)
Prediction interval: (-20689.773,14960.516)
*Confidence and prediction intervals **agree** in relation to clinically important effect*
Heterogeneity judgment: No concerns

Comparison Ace-K + Sucralose:Water
Evidence: mixed
NMA estimate: 842.504
95% intervals for NMA estimate
Confidence interval: (-8059.369,9744.377)
Prediction interval: (-13611.707,15296.716)
*Confidence and prediction intervals **agree** in relation to clinically important effect*
Heterogeneity judgment: No concerns

Comparison Aspartame:Sucralose
Evidence: mixed
NMA estimate: -6538.179
95% intervals for NMA estimate
Confidence interval: (-17301.473,4225.116)
Prediction interval: (-24014.829,10938.472)
*Confidence and prediction intervals **agree** in relation to clinically important effect*
Heterogeneity judgment: No concerns

Comparison Aspartame:Water
Evidence: mixed
NMA estimate: -7041.589
95% intervals for NMA estimate
Confidence interval: (-17886.145,3802.967)
Prediction interval: (-24650.187,10567.009)
*Confidence and prediction intervals **agree** in relation to clinically important effect*
Heterogeneity judgment: No concerns

Comparison Saccharin:Water
Evidence: mixed
NMA estimate: -2073.000
95% intervals for NMA estimate
Confidence interval: (-18097.756,13951.756)
Prediction interval: (-28092.828,23946.828)
*Confidence and prediction intervals **agree** in relation to clinically important effect*
Heterogeneity judgment: No concerns

Comparison Sucralose:Water
Evidence: mixed
NMA estimate: -503.410
95% intervals for NMA estimate
Confidence interval: (-4443.888,3437.068)
Prediction interval: (-6901.670,5894.850)
*Confidence and prediction intervals **agree** in relation to clinically important effect*
Heterogeneity judgment: No concerns

Comparison Ace-K + Aspartame + Sucralose:Aspartame
Evidence: indirect
NMA estimate: 4176.961
95% intervals for NMA estimate
Confidence interval: (-11254.149,19608.070)
Prediction interval: (-20878.947,29232.868)
*Confidence and prediction intervals **agree** in relation to clinically important effect*
Heterogeneity judgment: No concerns

Comparison Ace-K + Aspartame + Sucralose:Saccharin
Evidence: indirect
NMA estimate: -791.629
95% intervals for NMA estimate
Confidence interval: (-20216.034,18632.777)
Prediction interval: (-32331.559,30748.302)
*Confidence and prediction intervals **agree** in relation to clinically important effect*
Heterogeneity judgment: No concerns

Comparison Ace-K + Aspartame + Sucralose:Sucralose
Evidence: indirect
NMA estimate: -2361.218
95% intervals for NMA estimate
Confidence interval: (-14024.927,9302.490)
Prediction interval: (-21299.896,16577.460)
*Confidence and prediction intervals **agree** in relation to clinically important effect*
Heterogeneity judgment: No concerns

Comparison Ace-K + Sucralose:Aspartame
Evidence: indirect
NMA estimate: 7884.093
95% intervals for NMA estimate
Confidence interval: (-6146.151,21914.337)
Prediction interval: (-14897.192,30665.378)
*Confidence and prediction intervals **agree** in relation to clinically important effect*
Heterogeneity judgment: No concerns

Comparison Ace-K + Sucralose:Saccharin
Evidence: indirect
NMA estimate: 2915.504
95% intervals for NMA estimate
Confidence interval: (-15415.785,21246.793)
Prediction interval: (-26849.503,32680.512)
*Confidence and prediction intervals **agree** in relation to clinically important effect*
Heterogeneity judgment: No concerns

Comparison Ace-K + Sucralose:Sucralose
Evidence: indirect
NMA estimate: 1345.915
95% intervals for NMA estimate
Confidence interval: (-8389.111,11080.940)
Prediction interval: (-14461.108,17152.937)
*Confidence and prediction intervals **agree** in relation to clinically important effect*
Heterogeneity judgment: No concerns

Comparison Aspartame:Saccharin
Evidence: indirect
NMA estimate: -4968.589
95% intervals for NMA estimate
Confidence interval: (-24317.935,14380.757)
Prediction interval: (-36386.644,26449.466)
*Confidence and prediction intervals **agree** in relation to clinically important effect*
Heterogeneity judgment: No concerns

Comparison Saccharin:Sucralose
Evidence: indirect
NMA estimate: -1569.590
95% intervals for NMA estimate
Confidence interval: (-18071.716,14932.537)
Prediction interval: (-28364.536,25225.357)
*Confidence and prediction intervals **agree** in relation to clinically important effect*
Heterogeneity judgment: No concerns

Figure S46. *CINeMA output for the INCOHERENCE domain of DELAYED COUPLING INTERVENTIONS evaluating the effect of non-nutritive sweetened beverages (NNS beverages) sweetened single or blends of non-nutritive sweeteners (NNS), water, and sugar-sweetened beverages (SSBs) sweetened with caloric sweeteners on postprandial blood INSULIN response in HEALTHY participants*

Incoherence

Define clinically important size of effect: Mean difference 2250

*Relative effect estimates below **-2250.000** and above **2250.000** are considered clinically important.*
Importance of Incoherence depends on the variability of direct and indirect effects in relation to a clinically important size of effect

Global test based on a random-effects design-by-treatment interaction model
χ^2 statistic: 0.454 (2 degrees of freedom), P value: 0.797

Local tests: Separating indirect from direct evidence

Comparison Ace-K + Aspartame + Sucralose:Ace-K + Sucralose
Evidence: mixed
NMA mean difference: -3707.133(-12787.734,5373.469)
Direct mean difference: -3938.260(-13185.817,5309.297)
Indirect mean difference: -330.488(-36206.861,35545.886)
Inconsistency measures
Difference of mean differences: -3607.772(-40656.814,33441.269)
P value: 0.849
Incoherence judgment: No concerns

Comparison Ace-K + Aspartame + Sucralose:Water
Evidence: mixed
NMA mean difference: -2864.628(-13842.549,8113.292)
Direct mean difference: -2206.360(-14723.189,10310.469)
Indirect mean difference: -4728.621(-26217.490,16760.248)
Inconsistency measures
Difference of mean differences: 2522.261(-22346.243,27390.766)
P value: 0.842
Incoherence judgment: No concerns

Comparison Ace-K + Sucralose:Water
Evidence: direct
Direct mean difference: 867.456(-7908.657,9643.570)
Inconsistency measures: Not applicable
Incoherence judgment: No concerns

Comparison Aspartame:Sucralose
Evidence: mixed
NMA mean difference: -6538.179(-17301.473,4225.116)
Direct mean difference: -4534.600(-16797.899,7728.699)
Indirect mean difference: -12662.573(-34341.268,9016.122)
Inconsistency measures
Difference of mean differences: 8127.973(-16778.941,33034.886)
P value: 0.522
Incoherence judgment: No concerns

Comparison Aspartame:Water
Evidence: mixed
NMA mean difference: -7041.589(-17886.145,3802.967)
Direct mean difference: -9556.090(-22702.139,3589.959)
Indirect mean difference: -2036.481(-20790.032,16717.071)
Inconsistency measures
Difference of mean differences: -7519.609(-30421.886,15382.668)
P value: 0.520
Incoherence judgment: No concerns

Comparison Saccharin:Water
Evidence: direct
Direct mean difference: -2073.000(-18097.756,13951.756)
Inconsistency measures: Not applicable
Incoherence judgment: No concerns

Comparison Sucralose:Water
Evidence: direct
Direct mean difference: -514.183(-4449.960,3421.595)
Inconsistency measures: Not applicable
Incoherence judgment: No concerns

Comparison Ace-K + Aspartame + Sucralose:Aspartame
Evidence: indirect
Indirect mean difference: 4176.961(-11254.149,19608.070)
Inconsistency measures: Not applicable
Incoherence judgment: No concerns

Comparison Ace-K + Aspartame + Sucralose:Saccharin
Evidence: indirect
Indirect mean difference: -791.629(-20216.034,18632.777)
Inconsistency measures: Not applicable
Incoherence judgment: No concerns

Comparison Ace-K + Aspartame + Sucralose:Sucralose
Evidence: indirect
Indirect mean difference: -2361.218(-14024.927,9302.490)
Inconsistency measures: Not applicable
Incoherence judgment: No concerns

Comparison Ace-K + Sucralose:Aspartame
Evidence: indirect
Indirect mean difference: 7884.093(-6146.151,21914.337)
Inconsistency measures: Not applicable
Incoherence judgment: No concerns

Comparison Ace-K + Sucralose:Saccharin
Evidence: indirect
Indirect mean difference: 2915.504(-15415.785,21246.793)
Inconsistency measures: Not applicable
Incoherence judgment: No concerns

Comparison Ace-K + Sucralose:Sucralose
Evidence: indirect
Indirect mean difference: 1345.915(-8389.111,11080.940)
Inconsistency measures: Not applicable
Incoherence judgment: No concerns

Comparison Aspartame:Saccharin
Evidence: indirect
Indirect mean difference: -4968.589(-24317.935,14380.757)
Inconsistency measures: Not applicable
Incoherence judgment: No concerns

Comparison Saccharin:Sucralose
Evidence: indirect
Indirect mean difference: -1569.590(-18071.716,14932.537)
Inconsistency measures: Not applicable
Incoherence judgment: No concerns

Figure S47. *Network plot and meta-analysis of DELAYED COUPLING INTERVENTIONS evaluating the effect of non-nutritive sweetened beverages (NNS beverages) sweetened single or blends of non-nutritive sweeteners (NNS), water, and sugar-sweetened beverages (SSBs) sweetened with caloric sweeteners on postprandial blood INSULIN response in participants with TYPE 2 DIABETES*

Network Plot

2 Trials | 18 Participants
(Type 2 Diabetes)

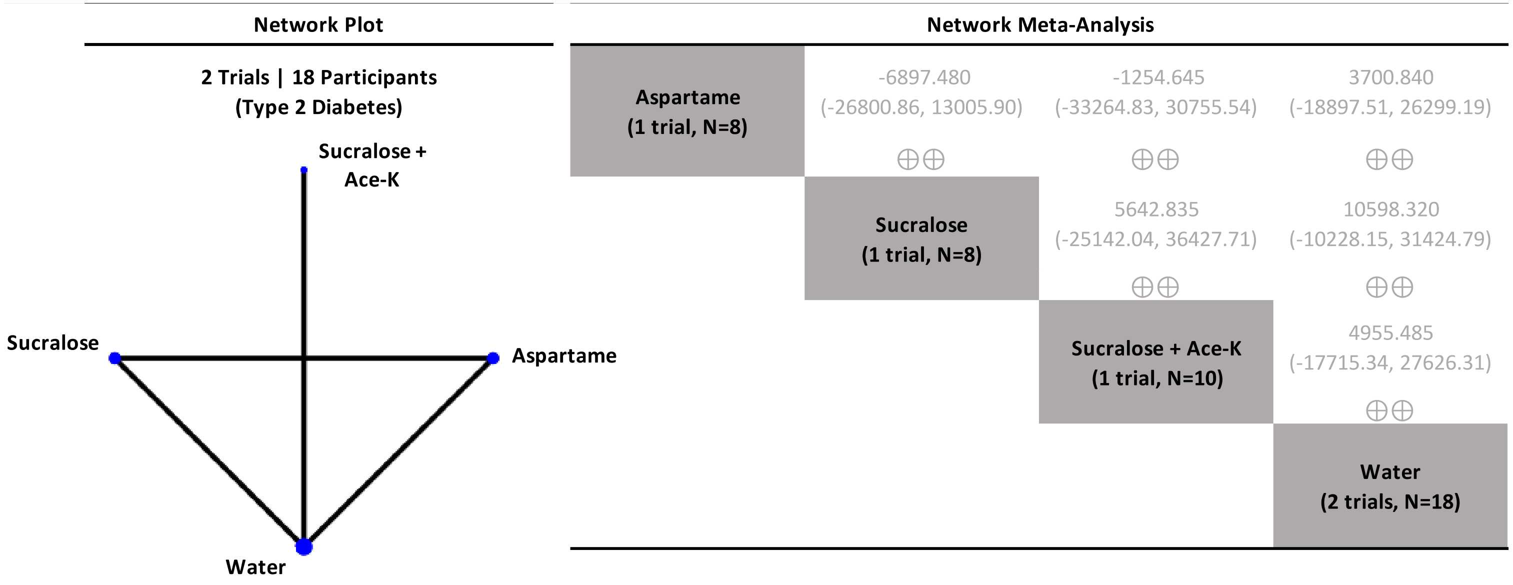

Network Meta-Analysis

**Aspartame (1 trial, N=8)**	-6897.480 (-26800.86, 13005.90) ⊕⊕	-1254.645 (-33264.83, 30755.54) ⊕⊕	3700.840 (-18897.51, 26299.19) ⊕⊕
	**Sucralose (1 trial, N=8)**	5642.835 (-25142.04, 36427.71) ⊕⊕	10598.320 (-10228.15, 31424.79) ⊕⊕
		**Sucralose + Ace-K (1 trial, N=10)**	4955.485 (-17715.34, 27626.31) ⊕⊕
			**Water (2 trials, N=18)**

Network plot: the size of the blue nodes is proportional to the number of participants and the line width is proportional to the number of studies. Network table: treatments are grouped by treatment type (i.e., individual non-nutritive sweeteners (NNS), NNS blends, water and caloric sweeteners) and are reported in alphabetical order. Treatment estimates (pmol*min/L) are MDs and 95% CIs of the column-defining treatment compared with the row-defining treatment. MDs less than 0 favor the column-defining treatment. MDs greater than 0 favor the row-defining treatment. Significant results are bolded in black. Non-significant results are grey and not bolded. The minimally important difference (MID) for postprandial glucose response is 2250 pmol*min/L. Trivial (significant) effects (<1 MID) or no effects have a white background; small important effects (≥1 MID) have a light blue background; moderate effects (≥2 MID) have a darker blue background; large effects (≥5 to <10 MID) have a purple background; very large effects (≥10 MID) have a black background. Confidence in the effect estimate is shown for each treatment comparison: high confidence ⊕⊕⊕⊕; moderate confidence ⊕⊕⊕; low confidence ⊕⊕; very low confidence ⊕. See **Supplementary Table 20 and Figure 48** for detailed assessments of the confidence in the effect estimate using the CINeMA framework.

Figure S48. *Risk of bias assessment of DELAYED COUPLING INTERVENTIONS evaluating the effect of non-nutritive sweetened beverages (NNS beverages) sweetened single or blends of non-nutritive sweeteners (NNS), water, and sugar-sweetened beverages (SSBs) sweetened with caloric sweeteners on postprandial blood INSULIN response in participants with TYPE 2 DIABETES*

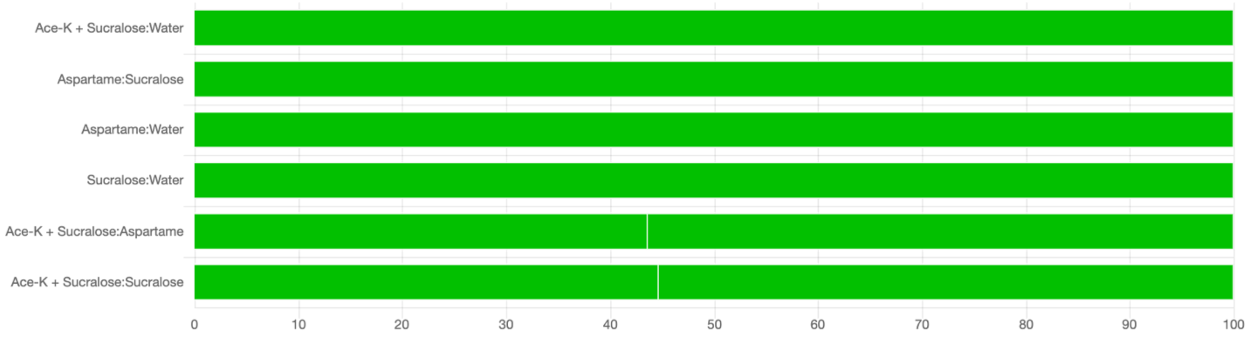

Green = no concerns; yellow = some concerns; red = major concerns

Figure S49. *Network plot and meta-analysis of DELAYED COUPLING INTERVENTIONS evaluating the effect of non-nutritive sweetened beverages (NNS beverages) sweetened single or blends of non-nutritive sweeteners (NNS), water, and sugar-sweetened beverages (SSBs) sweetened with caloric sweeteners on postprandial blood GLP-1 response in HEALTHY participants*

Network Plot

6 Trials | 119 Participants (Healthy)

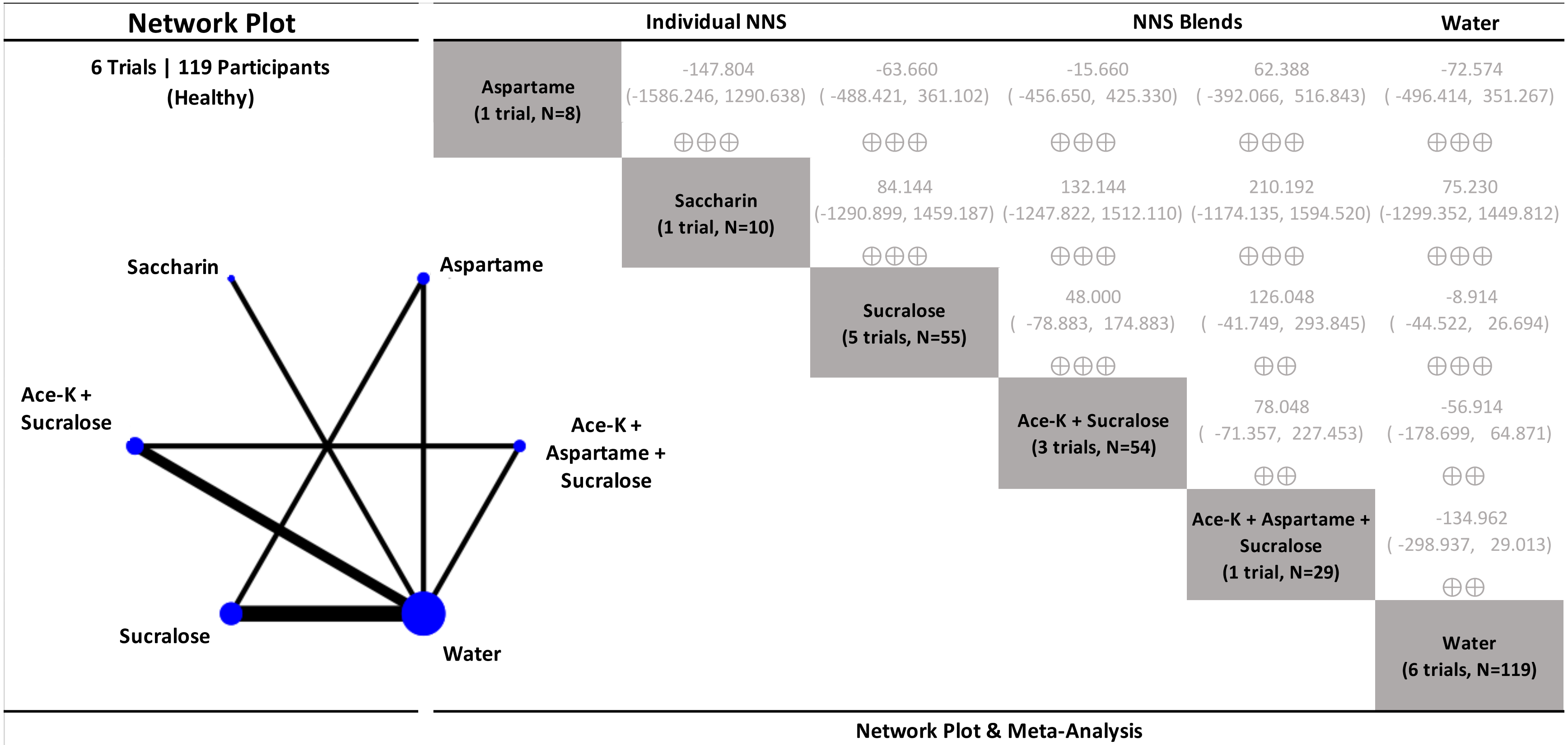

Individual NNS			NNS Blends		Water
**Aspartame (1 trial, N=8)**	-147.804<br>(-1586.246, 1290.638)<br>⊕⊕⊕	-63.660<br>(-488.421, 361.102)<br>⊕⊕⊕	-15.660<br>(-456.650, 425.330)<br>⊕⊕⊕	62.388<br>(-392.066, 516.843)<br>⊕⊕⊕	-72.574<br>(-496.414, 351.267)<br>⊕⊕⊕
	**Saccharin (1 trial, N=10)**	84.144<br>(-1290.899, 1459.187)<br>⊕⊕⊕	132.144<br>(-1247.822, 1512.110)<br>⊕⊕⊕	210.192<br>(-1174.135, 1594.520)<br>⊕⊕⊕	75.230<br>(-1299.352, 1449.812)<br>⊕⊕⊕
		**Sucralose (5 trials, N=55)**	48.000<br>(-78.883, 174.883)<br>⊕⊕⊕	126.048<br>(-41.749, 293.845)<br>⊕⊕	-8.914<br>(-44.522, 26.694)<br>⊕⊕⊕
			**Ace-K + Sucralose (3 trials, N=54)**	78.048<br>(-71.357, 227.453)<br>⊕⊕	-56.914<br>(-178.699, 64.871)<br>⊕⊕
				**Ace-K + Aspartame + Sucralose (1 trial, N=29)**	-134.962<br>(-298.937, 29.013)<br>⊕⊕
					**Water (6 trials, N=119)**

Network Plot & Meta-Analysis

Network plot: the size of the blue nodes is proportional to the number of participants and the line width is proportional to the number of studies. Network table: treatments are grouped by treatment type (i.e., individual non-nutritive sweeteners (NNS), NNS blends, water and caloric sweeteners) and are reported in alphabetical order. Treatment estimates (pmol*min/L) are MDs and 95% CIs of the column-defining treatment compared with the row-defining treatment. MDs less than 0 favor the column-defining treatment. MDs greater than 0 favor the row-defining treatment. Significant results are bolded in black. Non-significant results are

grey and not bolded. The minimally important difference (MID) for postprandial glucose response is 82 pmol*min/L. Trivial (significant) effects (<1 MID) or no effects have a white background; small important effects (≥1 MID) have a light blue background; moderate effects (≥2 MID) have a darker blue background; large effects (≥5 to <10 MID) have a purple background; very large effects (≥10 MID) have a black background. Confidence in the effect estimate is shown for each treatment comparison: high confidence ⨁⨁⨁⨁; moderate confidence ⨁⨁⨁; low confidence ⨁⨁; very low confidence ⨁. See **Supplementary Table 21 and Figures 50-53** for detailed assessments of the confidence in the effect estimate using the CINeMA framework.

Figure S50. *Risk of bias assessment of DELAYED COUPLING INTERVENTIONS evaluating the effect of non-nutritive sweetened beverages (NNS beverages) sweetened single or blends of non-nutritive sweeteners (NNS), water, and sugar-sweetened beverages (SSBs) sweetened with caloric sweeteners on postprandial blood GLP-1 response in HEALTHY participants*

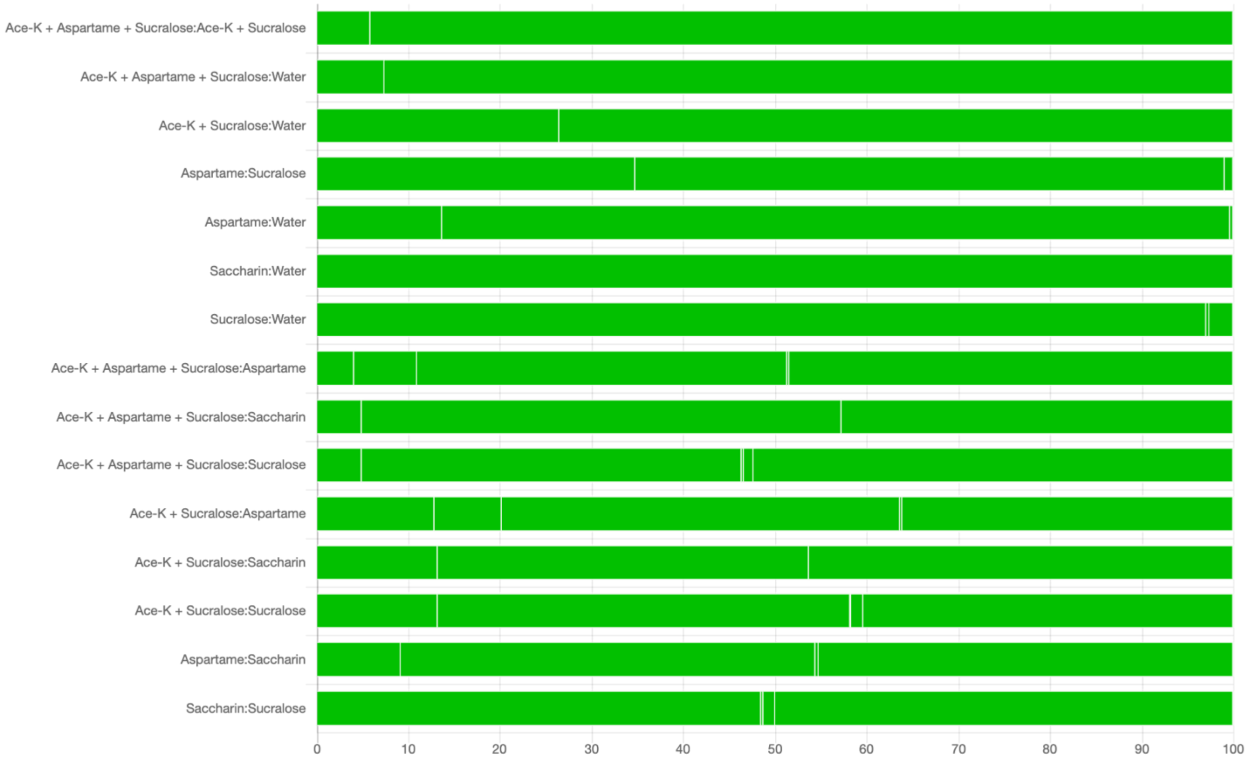

Green = no concerns; yellow = some concerns; red = major concerns

Figure S51. *CINeMA output for the IMPRECISION domain of DELAYED COUPLING INTERVENTIONS evaluating the effect of non-nutritive sweetened beverages (NNS beverages) sweetened single or blends of non-nutritive sweeteners (NNS), water, and sugar-sweetened beverages (SSBs) sweetened with caloric sweeteners on postprandial blood GLP-1 response in HEALTHY participants*

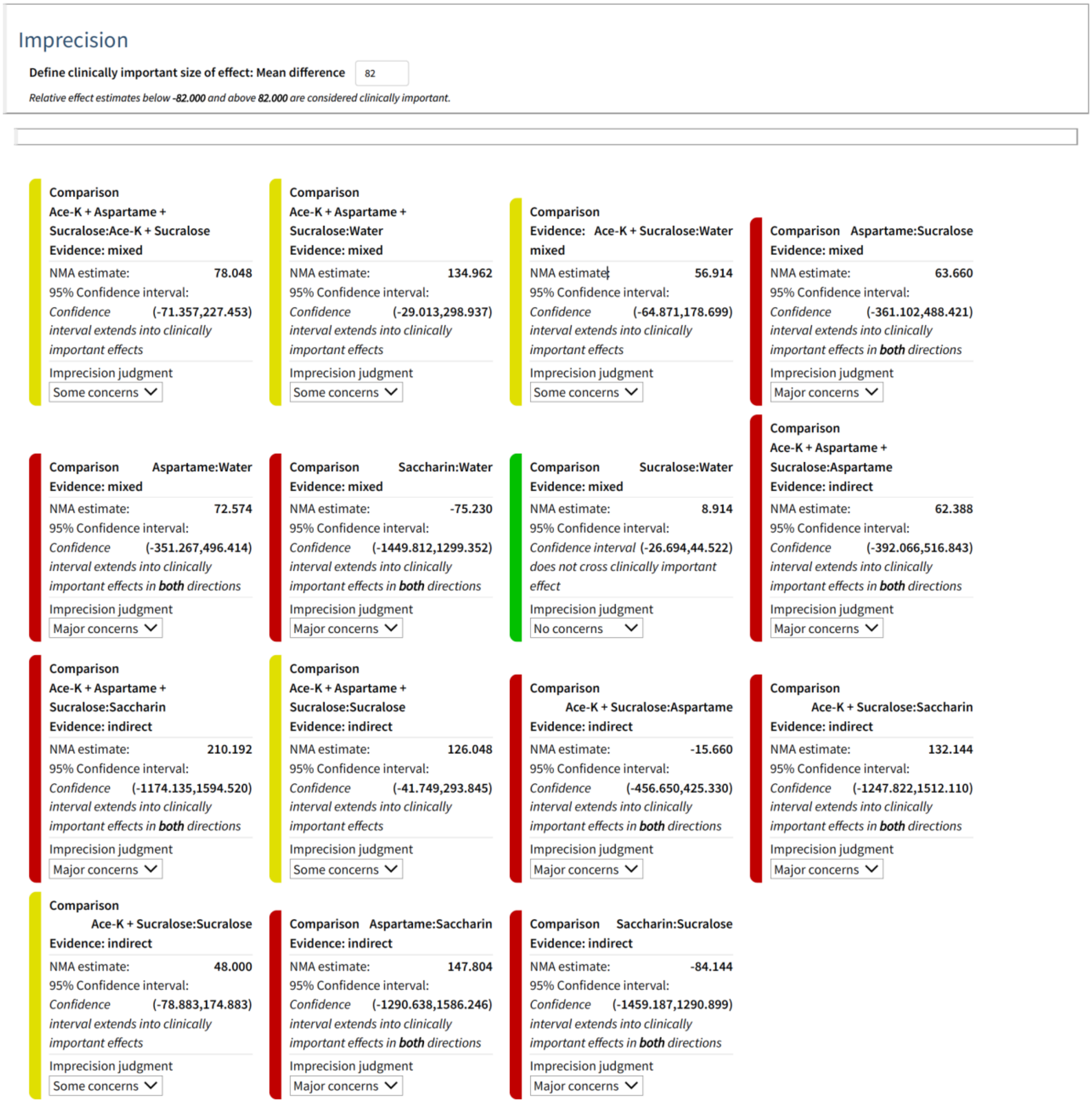

Relative effect estimates below -82.0 and above 82.0 pmol*min/L (minimal important difference; MID) were considered clinically important. Judgements for IMPRECISION were assigned by the CiNEMA algorithm (27, 28). There is no general consensus on a clinically important size difference for 2-hour blood GLP-1 incremental area under the curve (iAUC). Health Canada considers a 20% reduction in glucose iAUC to be the minimum physiologically relevant difference as per "Guideline Concerning the Safety and Physiological Effects of Novel Fibre

Sources and Food Products Containing Them" (29). Extrapolating the 20% threshold in glucose iAUC, we calculated the MID of GLP-1 iAUC by taking 20% of 412 pmol*mins/L which is the median of the difference in GLP-1 iAUC among healthy participants (~95 pmol*mins/L) and those with type 2 diabetes (~633 pmol*mins/L) after a glucose or meal tolerance test (30).

Figure S52. *CINeMA output for the HETEROGENEITY domain of DELAYED COUPLING INTERVENTIONS evaluating the effect of non-nutritive sweetened beverages (NNS beverages) sweetened single or blends of non-nutritive sweeteners (NNS), water, and sugar-sweetened beverages (SSBs) sweetened with caloric sweeteners on postprandial blood GLP-1 response in HEALTHY participants*

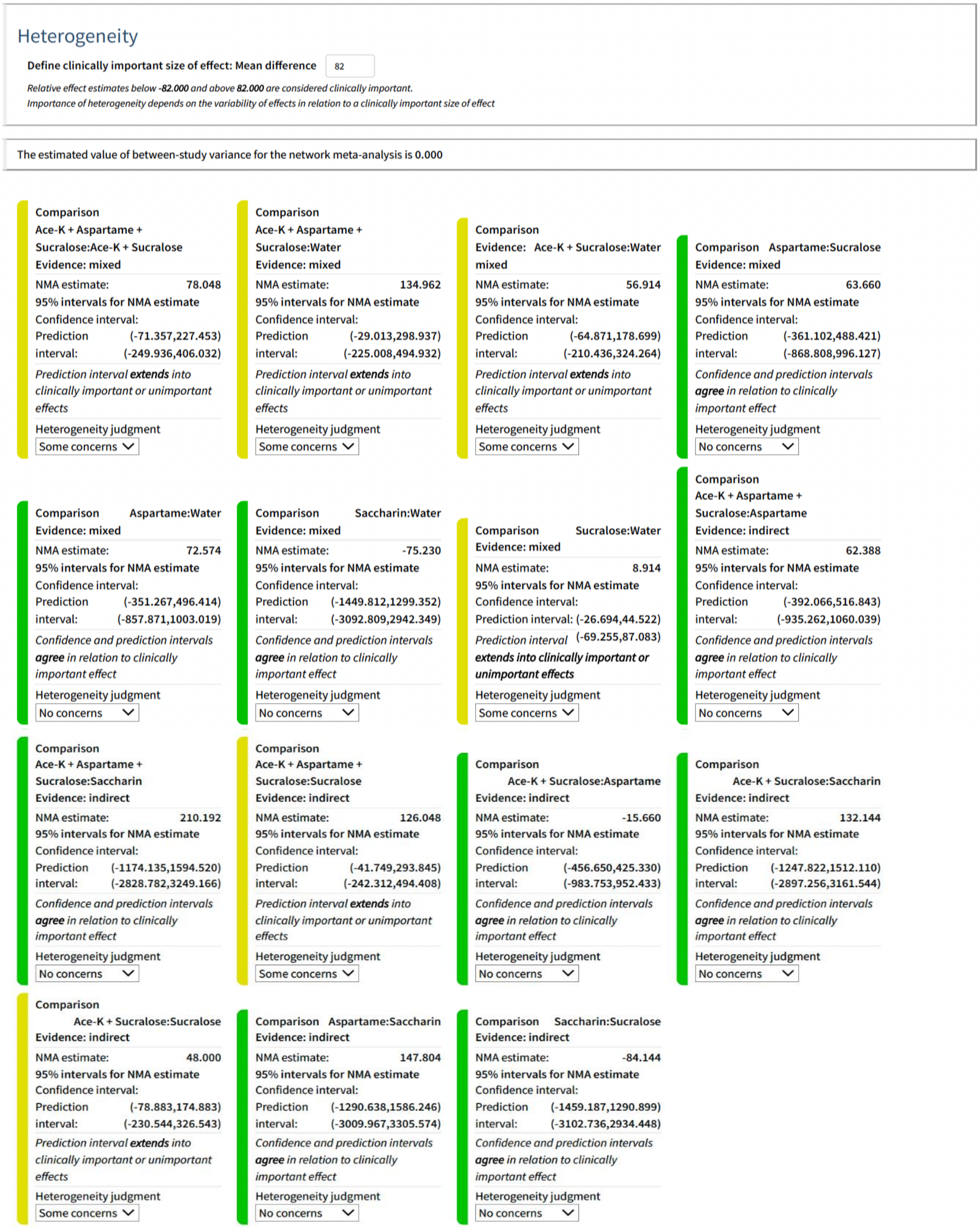

Figure S53. *CINeMA output for the INCOHERENCE domain of DELAYED COUPLING INTERVENTIONS evaluating the effect of non-nutritive sweetened beverages (NNS beverages) sweetened single or blends of non-nutritive sweeteners (NNS), water, and sugar-sweetened beverages (SSBs) sweetened with caloric sweeteners on postprandial blood GLP-1 response in HEALTHY participants*

Incoherence

Define clinically important size of effect: Mean difference 82

*Relative effect estimates below **-82.000** and above **82.000** are considered clinically important.*
Importance of Incoherence depends on the variability of direct and indirect effects in relation to a clinically important size of effect

Global test based on a random-effects design-by-treatment interaction model
χ^2 statistic: 1.860 (2 degrees of freedom), P value: 0.395

Local tests: Separating indirect from direct evidence

Comparison Ace-K + Aspartame + Sucralose:Ace-K + Sucralose
Evidence: mixed
NMA mean difference: 78.048(-71.357,227.453)
Direct mean difference: 84.770(-66.972,236.512)
Indirect mean difference: -135.117(-989.621,719.387)
Inconsistency measures
Difference of mean differences: 219.887(-647.985,1087.760)
P value: 0.620
Incoherence judgment: No concerns

Comparison Ace-K + Aspartame + Sucralose:Water
Evidence: mixed
NMA mean difference: 134.962(-29.013,298.937)
Direct mean difference: 122.260(-49.208,293.728)
Indirect mean difference: 270.847(-289.984,831.677)
Inconsistency measures
Difference of mean differences: -148.587(-735.044,437.870)
P value: 0.620
Incoherence judgment: No concerns

Comparison Ace-K + Sucralose:Water
Evidence: direct
Direct mean difference: 56.914(-64.871,178.699)
Inconsistency measures: Not applicable
Incoherence judgment: No concerns

Comparison Aspartame:Sucralose
Evidence: mixed
NMA mean difference: 63.660(-361.102,488.421)
Direct mean difference: -303.350(-1011.230,404.530)
Indirect mean difference: 270.154(-260.822,801.130)
Inconsistency measures
Difference of mean differences: -573.504(-1458.394,311.386)
P value: 0.204
Incoherence judgment: No concerns

Comparison Aspartame:Water
Evidence: mixed
NMA mean difference: 72.574(-351.267,496.414)
Direct mean difference: 159.590(-285.008,604.188)
Indirect mean difference: -794.585(-2198.097,608.927)
Inconsistency measures
Difference of mean differences: 954.175(-518.073,2426.422)
P value: 0.204
Incoherence judgment: No concerns

Comparison Saccharin:Water
Evidence: direct
Direct mean difference: -75.230(-1449.812,1299.352)
Inconsistency measures: Not applicable
Incoherence judgment: No concerns

Comparison Sucralose:Water
Evidence: direct
Direct mean difference: 8.914(-26.694,44.522)
Inconsistency measures: Not applicable
Incoherence judgment: No concerns

Comparison Ace-K + Aspartame + Sucralose:Aspartame
Evidence: indirect
Indirect mean difference: 62.388(-392.066,516.843)
Inconsistency measures: Not applicable
Incoherence judgment: No concerns

Comparison Ace-K + Aspartame + Sucralose:Saccharin
Evidence: indirect
Indirect mean difference: 210.192(-1174.135,1594.520)
Inconsistency measures: Not applicable
Incoherence judgment: No concerns

Comparison Ace-K + Aspartame + Sucralose:Sucralose
Evidence: indirect
Indirect mean difference: 126.048(-41.749,293.845)
Inconsistency measures: Not applicable
Incoherence judgment: No concerns

Comparison Ace-K + Sucralose:Aspartame
Evidence: indirect
Indirect mean difference: -15.660(-456.650,425.330)
Inconsistency measures: Not applicable
Incoherence judgment: No concerns

Comparison Ace-K + Sucralose:Saccharin
Evidence: indirect
Indirect mean difference: 132.144(-1247.822,1512.110)
Inconsistency measures: Not applicable
Incoherence judgment: No concerns

Comparison Ace-K + Sucralose:Sucralose
Evidence: indirect
Indirect mean difference: 48.000(-78.883,174.883)
Inconsistency measures: Not applicable
Incoherence judgment: No concerns

Comparison Aspartame:Saccharin
Evidence: indirect
Indirect mean difference: 147.804(-1290.638,1586.246)
Inconsistency measures: Not applicable
Incoherence judgment: No concerns

Comparison Saccharin:Sucralose
Evidence: indirect
Indirect mean difference: -84.144(-1459.187,1290.899)
Inconsistency measures: Not applicable
Incoherence judgment: No concerns

Figure S54. *Network plot and meta-analysis of DELAYED COUPLING INTERVENTIONS evaluating the effect of non-nutritive sweetened beverages (NNS beverages) sweetened single or blends of non-nutritive sweeteners (NNS), water, and sugar-sweetened beverages (SSBs) sweetened with caloric sweeteners on postprandial blood GLP-1 response in participants with TYPE 2 DIABETES*

Network Plot

2 Trials | 18 Participants (Type 2 Diabetes)

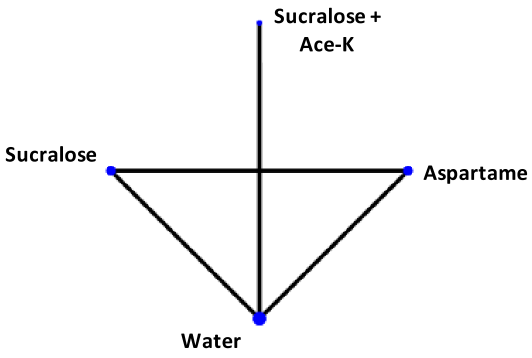

Network Meta-Analysis

**Aspartame (1 trial, N=8)**	-483.260 (-1314.391, 347.871) ⊕⊕	-332.706 (-1360.600, 695.188) ⊕⊕	-156.770 (-1127.779, 814.239) ⊕⊕
	**Sucralose (1 trial, N=8)**	150.554 (-790.271, 1091.379) ⊕⊕	326.490 (-551.829, 1204.809) ⊕⊕
		**Sucralose + Ace-K (1 trial, N=10)**	175.936 (-161.268, 513.141) ⊕⊕
			**Water (2 trials, N=18)**

Network plot: the size of the blue nodes is proportional to the number of participants and the line width is proportional to the number of studies. Network table: treatments are grouped by treatment type (i.e., individual non-nutritive sweeteners (NNS), NNS blends, water and caloric sweeteners) and are reported in alphabetical order. Treatment estimates (pmol*min/L) are MDs and 95% CIs of the column-defining treatment compared with the row-defining treatment. MDs less than 0 favor the column-defining treatment. MDs greater than 0 favor the row-defining treatment. Significant results are bolded in black. Non-significant results are grey and not bolded. The minimally important difference (MID) for postprandial glucose response is 82 pmol*min/L. Trivial (significant) effects (<1 MID) or no effects have a white background; small important effects (≥1 MID) have a light blue background; moderate effects (≥2 MID) have a darker blue background; large effects (≥5 to <10 MID) have a purple background; very large effects (≥10 MID) have a black background. Confidence in the effect estimate is shown for each treatment comparison: high confidence ⊕⊕⊕⊕; moderate confidence ⊕⊕⊕; low confidence ⊕⊕; very low confidence ⊕. See **Supplementary Table 22 and Figure 55** for detailed assessments of the confidence in the effect estimate using the CINeMA framework.

__Figure S55.__ Risk of bias assessment of DELAYED COUPLING INTERVENTIONS evaluating the effect of non-nutritive sweetened beverages (NNS beverages) sweetened single or blends of non-nutritive sweeteners (NNS), water, and sugar-sweetened beverages (SSBs) sweetened with caloric sweeteners on postprandial blood GLP-1 response in participants with TYPE 2 DIABETES

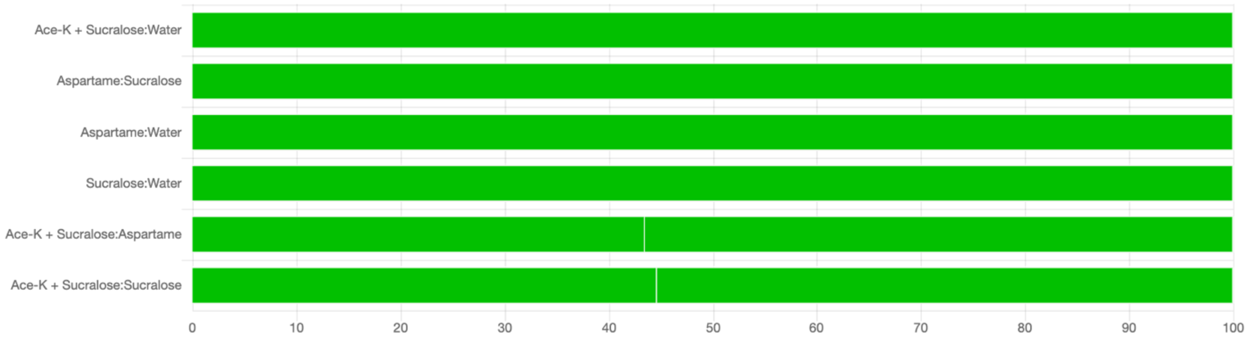

Green = no concerns; yellow = some concerns; red = major concerns

Figure S56. *Network plot and meta-analysis of DELAYED COUPLING INTERVENTIONS evaluating the effect of non-nutritive sweetened beverages (NNS beverages) sweetened single or blends of non-nutritive sweeteners (NNS), water, and sugar-sweetened beverages (SSBs) sweetened with caloric sweeteners on postprandial blood GIP response in HEALTHY participants*

Network Plot

5 Trials | 111 Participants (Healthy)

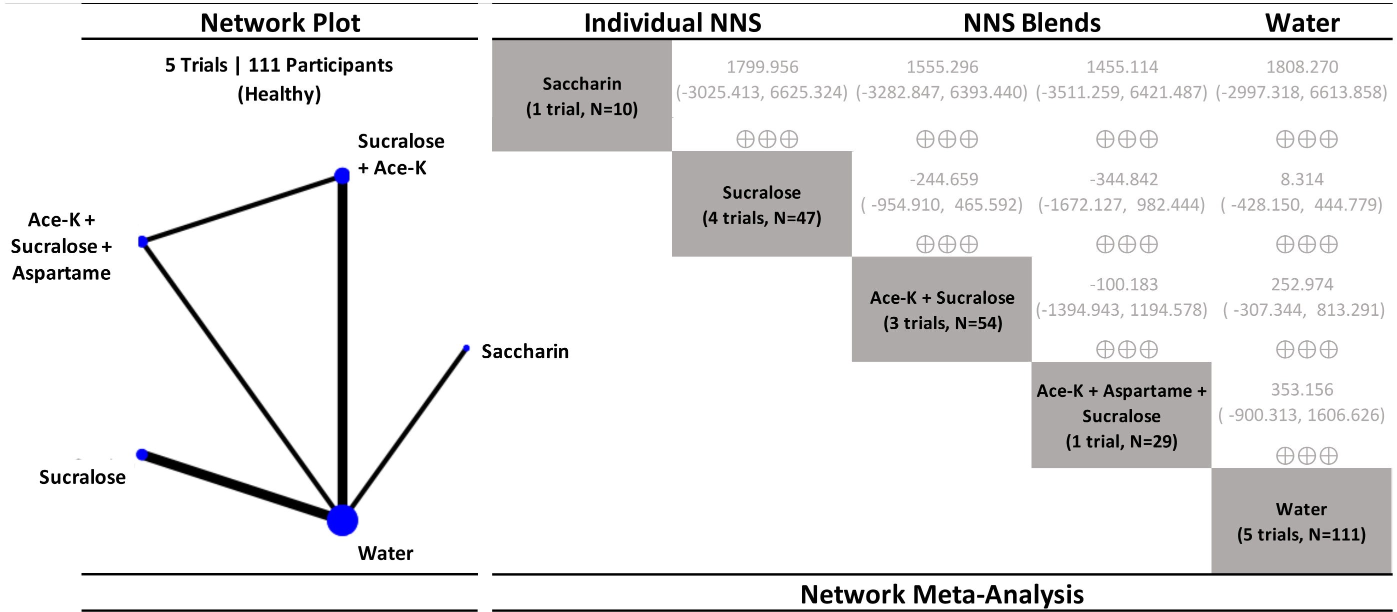

Network Meta-Analysis

Individual NNS		NNS Blends		Water
**Saccharin (1 trial, N=10)**	1799.956 (-3025.413, 6625.324) ⊕⊕⊕	1555.296 (-3282.847, 6393.440) ⊕⊕⊕	1455.114 (-3511.259, 6421.487) ⊕⊕⊕	1808.270 (-2997.318, 6613.858) ⊕⊕⊕
	**Sucralose (4 trials, N=47)**	-244.659 (-954.910, 465.592) ⊕⊕⊕	-344.842 (-1672.127, 982.444) ⊕⊕⊕	8.314 (-428.150, 444.779) ⊕⊕⊕
		**Ace-K + Sucralose (3 trials, N=54)**	-100.183 (-1394.943, 1194.578) ⊕⊕⊕	252.974 (-307.344, 813.291) ⊕⊕⊕
			**Ace-K + Aspartame + Sucralose (1 trial, N=29)**	353.156 (-900.313, 1606.626) ⊕⊕⊕
				**Water (5 trials, N=111)**

Network plot: the size of the blue nodes is proportional to the number of participants and the line width is proportional to the number of studies. Network table: treatments are grouped by treatment type (i.e., individual non-nutritive sweeteners (NNS), NNS blends, water and caloric sweeteners) and are reported in alphabetical order. Treatment estimates (pmol*min/L) are MDs and 95% CIs of the column-defining treatment compared with the row-defining treatment. MDs less than 0 favor the column-defining treatment. MDs greater than 0 favor the row-defining treatment. Significant results are bolded in black. Non-significant results are grey and not bolded. The minimally important difference (MID) for postprandial glucose response is 94 pmol*min/L. Trivial (significant) effects (<1 MID) or no effects have a white background; small important effects (≥1 MID) have a light blue background; moderate effects (≥2 MID) have a darker blue background; large effects (≥5 to <10 MID) have a purple background; very large effects (≥10 MID) have a black background. Confidence in the effect estimate is shown for each treatment comparison: high confidence ⊕⊕⊕⊕; moderate confidence ⊕⊕⊕; low confidence ⊕⊕; very low confidence ⊕. See **Supplementary Table 23 and Figures 57-60** for detailed assessments of the confidence in the effect estimate using the CINeMA framework.

Figure S57. *Risk of bias assessment of DELAYED COUPLING INTERVENTIONS evaluating the effect of non-nutritive sweetened beverages (NNS beverages) sweetened single or blends of non-nutritive sweeteners (NNS), water, and sugar-sweetened beverages (SSBs) sweetened with caloric sweeteners on postprandial blood GIP response in HEALTHY participants*

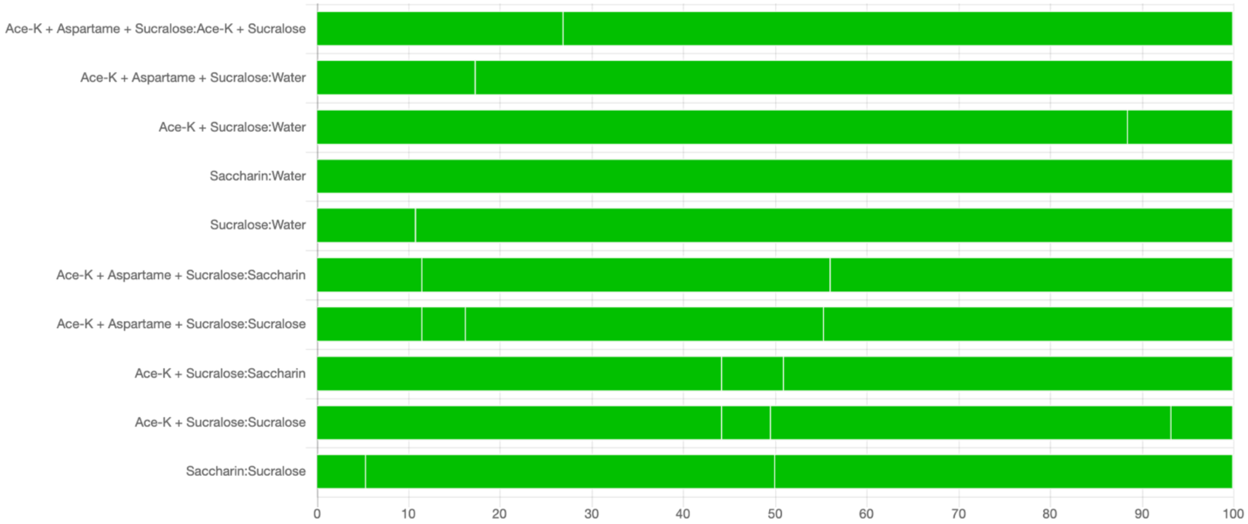

Green = no concerns; yellow = some concerns; red = major concerns

Figure S58. *CINeMA output for the IMPRECISION domain of DELAYED COUPLING INTERVENTIONS evaluating the effect of non-nutritive sweetened beverages (NNS beverages) sweetened single or blends of non-nutritive sweeteners (NNS), water, and sugar-sweetened beverages (SSBs) sweetened with caloric sweeteners on postprandial blood GIP response in HEALTHY participants*

Imprecision

Define clinically important size of effect: Mean difference 94

*Relative effect estimates below **-94.000** and above **94.000** are considered clinically important.*

Comparison Ace-K + Aspartame + Sucralose:Ace-K + Sucralose
Evidence: mixed
NMA estimate: **-100.183**
95% Confidence interval: **(-1394.943,1194.578)**
*Confidence interval extends into clinically important effects in **both** directions*
Imprecision judgment: Major concerns

Comparison Ace-K + Aspartame + Sucralose:Water
Evidence: mixed
NMA estimate: **-353.156**
95% Confidence interval: **(-1606.626,900.313)**
*Confidence interval extends into clinically important effects in **both** directions*
Imprecision judgment: Major concerns

Comparison Ace-K + Sucralose:Water
Evidence: mixed
NMA estimate: **-252.974**
95% Confidence interval: **(-813.291,307.344)**
*Confidence interval extends into clinically important effects in **both** directions*
Imprecision judgment: Major concerns

Comparison Saccharin:Water
Evidence: mixed
NMA estimate: **-1808.270**
95% Confidence interval: **(-6613.858,2997.318)**
*Confidence interval extends into clinically important effects in **both** directions*
Imprecision judgment: Major concerns

Comparison Sucralose:Water
Evidence: mixed
NMA estimate: **-8.314**
95% Confidence interval: **(-444.779,428.150)**
*Confidence interval extends into clinically important effects in **both** directions*
Imprecision judgment: Major concerns

Comparison Ace-K + Aspartame + Sucralose:Saccharin
Evidence: indirect
NMA estimate: **1455.114**
95% Confidence interval: **(-3511.259,6421.487)**
*Confidence interval extends into clinically important effects in **both** directions*
Imprecision judgment: Major concerns

Comparison Ace-K + Aspartame + Sucralose:Sucralose
Evidence: indirect
NMA estimate: **-344.842**
95% Confidence interval: **(-1672.127,982.444)**
*Confidence interval extends into clinically important effects in **both** directions*
Imprecision judgment: Major concerns

Comparison Ace-K + Sucralose:Saccharin
Evidence: indirect
NMA estimate: **1555.296**
95% Confidence interval: **(-3282.847,6393.440)**
*Confidence interval extends into clinically important effects in **both** directions*
Imprecision judgment: Major concerns

Comparison Ace-K + Sucralose:Sucralose
Evidence: indirect
NMA estimate: **-244.659**
95% Confidence interval: **(-954.910,465.592)**
*Confidence interval extends into clinically important effects in **both** directions*
Imprecision judgment: Major concerns

Comparison Saccharin:Sucralose
Evidence: indirect
NMA estimate: **-1799.956**
95% Confidence interval: **(-6625.324,3025.413)**
*Confidence interval extends into clinically important effects in **both** directions*
Imprecision judgment: Major concerns

Relative effect estimates below -94.0 and above 94.0 pmol*min/L (minimal important difference; MID) were considered clinically important. Judgements for IMPRECISION were assigned by the CiNEMA algorithm (27, 28). There is no general consensus on a clinically important size difference for 2-hour blood GIP incremental area under the curve (iAUC). Health Canada considers a 20% reduction in glucose iAUC to be the minimum physiologically relevant difference as per "Guideline Concerning the Safety and Physiological Effects of Novel Fibre Sources and Food Products Containing Them" (29). Extrapolating the 20% threshold in glucose iAUC, we calculated the MID of GIP iAUC by taking 20% of 468 pmol*mins/L which is the median of the difference in GIP iAUC among healthy participants (~168 pmol*mins/L) and those with type 2 diabetes (~768 pmol*mins/L) after a glucose or meal tolerance test (31).

Figure S59. *CINeMA output for the HETEROGENEITY domain of DELAYED COUPLING INTERVENTIONS evaluating the effect of non-nutritive sweetened beverages (NNS beverages) sweetened single or blends of non-nutritive sweeteners (NNS), water, and sugar-sweetened beverages (SSBs) sweetened with caloric sweeteners on postprandial blood GIP response in HEALTHY participants*

Heterogeneity

Define clinically important size of effect: Mean difference 94

*Relative effect estimates below **-94.000** and above **94.000** are considered clinically important.*
Importance of heterogeneity depends on the variability of effects in relation to a clinically important size of effect

The estimated value of between-study variance for the network meta-analysis is **0.000**

Comparison
Ace-K + Aspartame + Sucralose:Ace-K + Sucralose
Evidence: mixed
NMA estimate: **-100.183**
95% intervals for NMA estimate
Confidence interval: **(-1394.943,1194.578)**
Prediction interval: **(-8493.955,8293.590)**
*Confidence and prediction intervals **agree** in relation to clinically important effect*
Heterogeneity judgment
No concerns

Comparison
Ace-K + Aspartame + Sucralose:Water
Evidence: mixed
NMA estimate: **-353.156**
95% intervals for NMA estimate
Confidence interval: **(-1606.626,900.313)**
Prediction interval: **(-8479.243,7772.931)**
*Confidence and prediction intervals **agree** in relation to clinically important effect*
Heterogeneity judgment
No concerns

Comparison
Ace-K + Sucralose:Water
Evidence: mixed
NMA estimate: **-252.974**
95% intervals for NMA estimate
Confidence interval: **(-813.291,307.344)**
Prediction interval: **(-3885.442,3379.494)**
*Confidence and prediction intervals **agree** in relation to clinically important effect*
Heterogeneity judgment
No concerns

Comparison **Saccharin:Water**
Evidence: mixed
NMA estimate: **-1808.270**
95% intervals for NMA estimate
Confidence interval: **(-6613.858,2997.318)**
Prediction interval: **(-32962.305,29345.765)**
*Confidence and prediction intervals **agree** in relation to clinically important effect*
Heterogeneity judgment
No concerns

Comparison **Sucralose:Water**
Evidence: mixed
NMA estimate: **-8.314**
95% intervals for NMA estimate
Confidence interval: **(-444.779,428.150)**
Prediction interval: **(-2837.858,2821.229)**
*Confidence and prediction intervals **agree** in relation to clinically important effect*
Heterogeneity judgment
No concerns

Comparison
Ace-K + Aspartame + Sucralose:Saccharin
Evidence: indirect
NMA estimate: **1455.114**
95% intervals for NMA estimate
Confidence interval: **(-3511.259,6421.487)**
Prediction interval: **(-30741.271,33651.499)**
*Confidence and prediction intervals **agree** in relation to clinically important effect*
Heterogeneity judgment
No concerns

Comparison
Ace-K + Aspartame + Sucralose:Sucralose
Evidence: indirect
NMA estimate: **-344.842**
95% intervals for NMA estimate
Confidence interval: **(-1672.127,982.444)**
Prediction interval: **(-8949.469,8259.785)**
*Confidence and prediction intervals **agree** in relation to clinically important effect*
Heterogeneity judgment
No concerns

Comparison
Ace-K + Sucralose:Saccharin
Evidence: indirect
NMA estimate: **1555.296**
95% intervals for NMA estimate
Confidence interval: **(-3282.847,6393.440)**
Prediction interval: **(-29809.792,32920.384)**
*Confidence and prediction intervals **agree** in relation to clinically important effect*
Heterogeneity judgment
No concerns

Comparison
Ace-K + Sucralose:Sucralose
Evidence: indirect
NMA estimate: **-244.659**
95% intervals for NMA estimate
Confidence interval: **(-954.910,465.592)**
Prediction interval: **(-4849.129,4359.810)**
*Confidence and prediction intervals **agree** in relation to clinically important effect*
Heterogeneity judgment
No concerns

Comparison **Saccharin:Sucralose**
Evidence: indirect
NMA estimate: **-1799.956**
95% intervals for NMA estimate
Confidence interval: **(-6625.324,3025.413)**
Prediction interval: **(-33082.223,29482.311)**
*Confidence and prediction intervals **agree** in relation to clinically important effect*
Heterogeneity judgment
No concerns

Figure S60. *CINeMA output for the INCOHERENCE domain of DELAYED COUPLING INTERVENTIONS evaluating the effect of non-nutritive sweetened beverages (NNS beverages) sweetened single or blends of non-nutritive sweeteners (NNS), water, and sugar-sweetened beverages (SSBs) sweetened with caloric sweeteners on postprandial blood GIP response in HEALTHY participants*

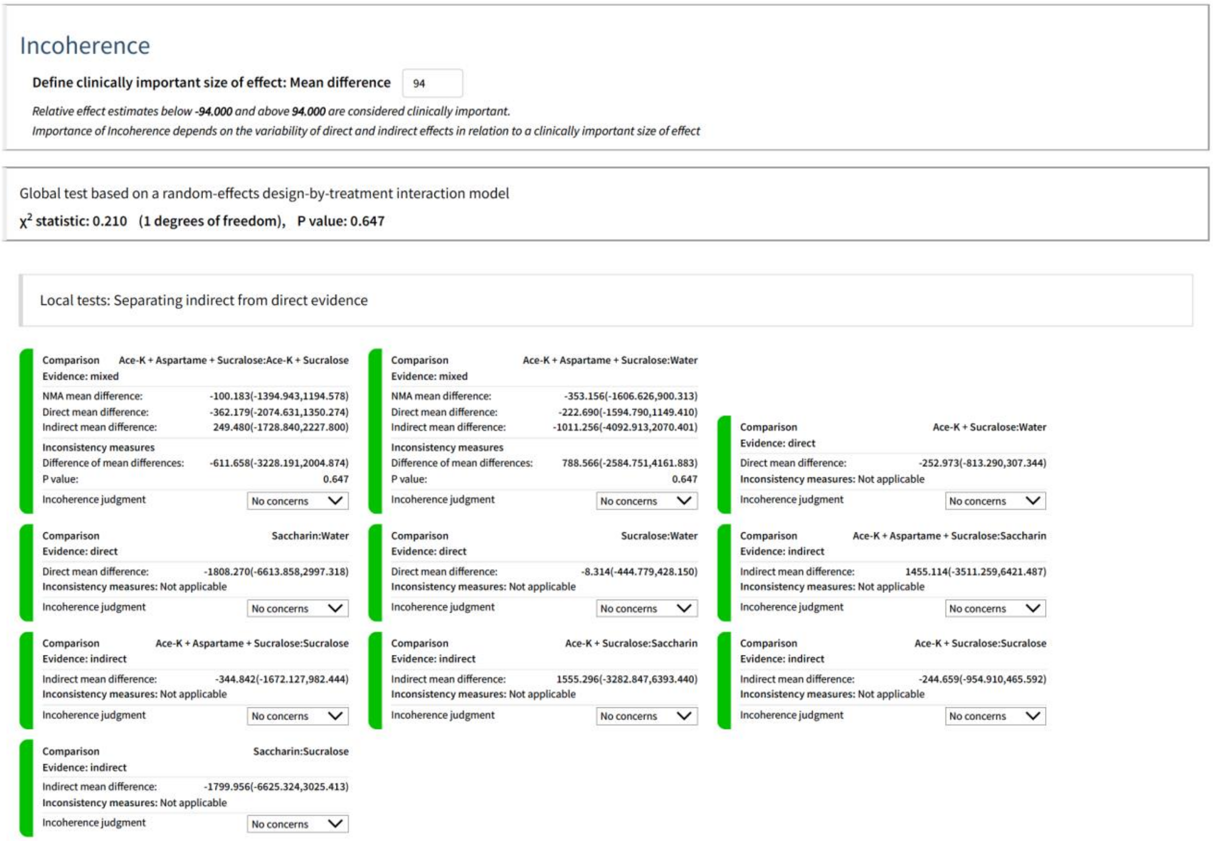

Figure S61. *Network plot and meta-analysis of DELAYED COUPLING INTERVENTIONS evaluating the effect of non-nutritive sweetened beverages (NNS beverages) sweetened single or blends of non-nutritive sweeteners (NNS), water, and sugar-sweetened beverages (SSBs) sweetened with caloric sweeteners on postprandial blood GLUCAGON response in HEALTHY participants*

Network Plot

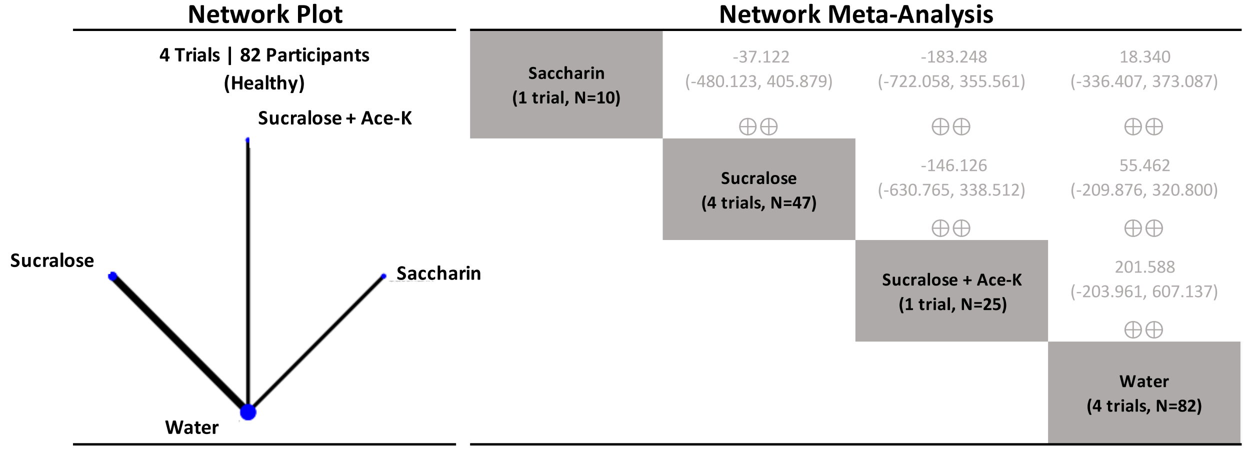

Network Meta-Analysis

**Saccharin (1 trial, N=10)**	-37.122 (-480.123, 405.879) ⊕⊕	-183.248 (-722.058, 355.561) ⊕⊕	18.340 (-336.407, 373.087) ⊕⊕
	**Sucralose (4 trials, N=47)**	-146.126 (-630.765, 338.512) ⊕⊕	55.462 (-209.876, 320.800) ⊕⊕
		**Sucralose + Ace-K (1 trial, N=25)**	201.588 (-203.961, 607.137) ⊕⊕
			**Water (4 trials, N=82)**

Network plot: the size of the blue nodes is proportional to the number of participants and the line width is proportional to the number of studies. Network table: treatments are grouped by treatment type (i.e., individual non-nutritive sweeteners (NNS), NNS blends, water and caloric sweeteners) and are reported in alphabetical order. Treatment estimates (pmol*min/L) are MDs and 95% CIs of the column-defining treatment compared with the row-defining treatment. MDs less than 0 favor the column-defining treatment. MDs greater than 0 favor the row-defining treatment. Significant results are bolded in white. Non-significant results are grey and not bolded. The minimally important difference (MID) for postprandial glucose response is 205 pmol*min/L. Trivial (significant) effects (<1 MID) or no effects have a white background; small important effects ($\geq$1 MID) have a light blue background; moderate effects ($\geq$2 MID) have a darker blue background; large effects ($\geq$5 to <10 MID) have a purple background; very large effects ($\geq$10 MID) have a black background. Confidence in the effect estimate is shown for each treatment comparison: high confidence ⊕⊕⊕⊕; moderate confidence ⊕⊕⊕; low confidence ⊕⊕; very low confidence ⊕. See **Supplementary Table 24 and Figure 62** for detailed assessments of the confidence in the effect estimate using the CINeMA framework.

Figure S62. *Risk of bias assessment of DELAYED COUPLING INTERVENTIONS evaluating the effect of non-nutritive sweetened beverages (NNS beverages) sweetened single or blends of non-nutritive sweeteners (NNS), water, and sugar-sweetened beverages (SSBs) sweetened with caloric sweeteners on postprandial blood GLUCAGON response in HEALTHY participants*

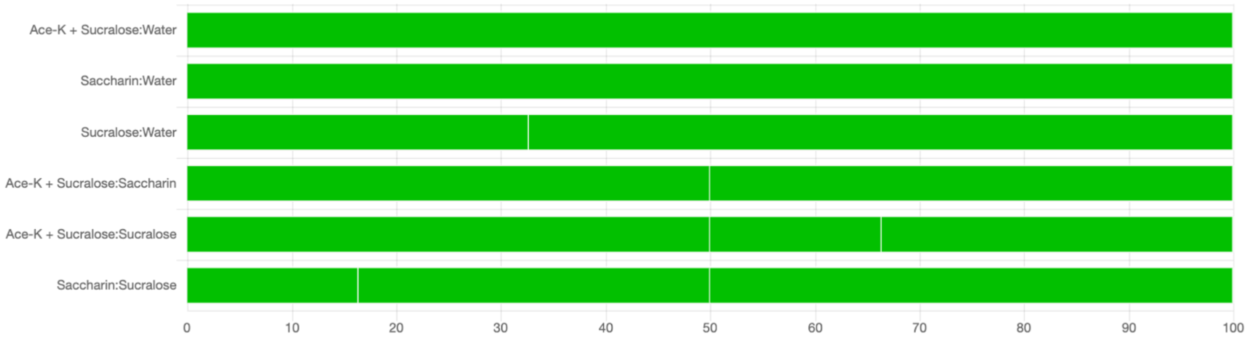

Green = no concerns; yellow = some concerns; red = major concerns

SUPPLEMENTARY REFERENCES

1. U.S. Food and Drug Administration. Additional Information about High-Intensity Sweeteners Permitted for Use in Food in the United States; 2018. Accessed at https://www.fda.gov/food/food-additives-petitions/additional-information-about-high-intensity-sweeteners-permitted-use-food-united-states on October 2, 2021. .
2. Shigeta H, Yoshida T, Nakai M, Mori H, Kano Y, Nishioka H, et al. Effects of aspartame on diabetic rats and diabetic patients. J Nutr Sci Vitaminol (Tokyo). 1985;31(5):533-40.
3. Okuno G, Kawakami F, Tako H, Kashihara T, Shibamoto S, Yamazaki T, et al. Glucose tolerance, blood lipid, insulin and glucagon concentration after single or continuous administration of aspartame in diabetics. Diabetes Res Clin Pract. 1986;2(1):23-7.
4. Horwitz DL, McLane M, Kobe P. Response to single dose of aspartame or saccharin by NIDDM patients. Diabetes Care. 1988;11(3):230-4.
5. Møller SE. Effect of aspartame and protein, administered in phenylalanine-equivalent doses, on plasma neutral amino acids, aspartate, insulin and glucose in man. Pharmacol Toxicol. 1991;68(5):408-12.
6. Härtel B, Graubaum H, Schneider B. The Influence of Sweetener Solutions on the Secretion of Insulin and the Blood Glucose Level. Ernährungsumschau. 1993;40(4):152-55.
7. Nguyen UN, Dumoulin G, Henriet MT, Regnard J. Aspartame ingestion increases urinary calcium, but not oxalate excretion, in healthy subjects. J Clin Endocrinol Metab. 1998;83(1):165-8.
8. Coppola L, Coppola A, Grassia A, Mastrolorenzo L, Lettieri B, De Lucia D, et al. Acute hyperglycemia alters von Willebrand factor but not the fibrinolytic system in elderly subjects with normal or impaired glucose tolerance. Blood Coagul Fibrinolysis. 2004;15(8):629-35.
9. Berlin I, Vorspan F, Warot D, Manéglier B, Spreux-Varoquaux O. Effect of glucose on tobacco craving. Is it mediated by tryptophan and serotonin? Psychopharmacology (Berl). 2005;178(1):27-34.
10. Ford HE, Peters V, Martin NM, Sleeth ML, Ghatei MA, Frost GS, et al. Effects of oral ingestion of sucralose on gut hormone response and appetite in healthy normal-weight subjects. Eur J Clin Nutr. 2011;65(4):508-13.
11. Maersk M, Belza A, Holst JJ, Fenger-Grøn M, Pedersen SB, Astrup A, et al. Satiety scores and satiety hormone response after sucrose-sweetened soft drink compared with isocaloric semi-skimmed milk and with non-caloric soft drink: a controlled trial. Eur J Clin Nutr. 2012;66(4):523-9.
12. Hazali N, Mohamed A, Ibrahim M, Masri M, Anuar K, Norazmir MN, et al. Effect of Acute Stevia Consumption on Blood Glucose Response in Healthy Malay Young Adults. Sains Malaysiana. 2014;43:649-54.
13. Bloomer R, Peel S, Moran R, MacDonnchadh J. Blood glucose and insulin response to artificially- and sugar-sweetened sodas in healthy men. Integrative Food, Nutrition and Metabolism. 2016;3.
14. González-Domínguez R, Mateos RM, Lechuga-Sancho AM, González-Cortés JJ, Corrales-Cuevas M, Rojas-Cots JA, et al. Synergic effects of sugar and caffeine on insulin-mediated

metabolomic alterations after an acute consumption of soft drinks. Electrophoresis. 2017;38(18):2313-22.
15. Goza R, Bunout D, Barrera G, de la Maza M, Hirsch S. Effect of Acute Consumption of Artificially Sweetened Beverages on Blood Glucose and Insulin in Healthy Subjects. Journal of Nutrition & Food Sciences. 2018;08.
16. Eckstein MLB, A. Haupt, S. Schierbauer, J. R. Zimmer, R. T. Wachsmuth, N. Zunner, B. Zimmermann, P. Obermayer-Pietsch, B. Moser, O. Acute Metabolic Responses to Glucose and Fructose Supplementation in Healthy Individuals: A Double-Blind Randomized Crossover Placebo-Controlled Trial. Nutrients. 2021;13(11):16.
17. Wolf-Novak LC, Stegink LD, Brummel MC, Persoon TJ, Filer LJ, Jr., Bell EF, et al. Aspartame ingestion with and without carbohydrate in phenylketonuric and normal subjects: effect on plasma concentrations of amino acids, glucose, and insulin. Metabolism. 1990;39(4):391-6.
18. Melchior JC, Rigaud D, Colas-Linhart N, Petiet A, Girard A, Apfelbaum M. Immunoreactive beta-endorphin increases after an aspartame chocolate drink in healthy human subjects. Physiol Behav. 1991;50(5):941-4.
19. Solomi L, Rees GA, Redfern KM. The acute effects of the non-nutritive sweeteners aspartame and acesulfame-K in UK diet cola on glycaemic response. Int J Food Sci Nutr. 2019;70(7):894-900.
20. Brown RJ, Walter M, Rother KI. Effects of diet soda on gut hormones in youths with diabetes. Diabetes Care. 2012;35(5):959-64.
21. Pepino MY, Tiemann CD, Patterson BW, Wice BM, Klein S. Sucralose affects glycemic and hormonal responses to an oral glucose load. Diabetes Care. 2013;36(9):2530-5.
22. Temizkan S, Deyneli O, Yasar M, Arpa M, Gunes M, Yazici D, et al. Sucralose enhances GLP-1 release and lowers blood glucose in the presence of carbohydrate in healthy subjects but not in patients with type 2 diabetes. Eur J Clin Nutr. 2015;69(2):162-6.
23. Sylvetsky AC, Brown RJ, Blau JE, Walter M, Rother KI. Hormonal responses to non-nutritive sweeteners in water and diet soda. Nutrition & Metabolism. 2016;13(1):71.
24. Karimian Azari E, Smith KR, Yi F, Osborne TF, Bizzotto R, Mari A, et al. Inhibition of sweet chemosensory receptors alters insulin responses during glucose ingestion in healthy adults: a randomized crossover interventional study. Am J Clin Nutr. 2017;105(4):1001-9.
25. Nichol AD, Salame C, Rother KI, Pepino MY. Effects of Sucralose Ingestion versus Sucralose Taste on Metabolic Responses to an Oral Glucose Tolerance Test in Participants with Normal Weight and Obesity: A Randomized Crossover Trial. Nutrients. 2019;12(1).
26. Solomi, L. (2020) ‘Diet cola and glycaemia: The acute effects of a preload containing the non-nutritive sweeteners aspartame and acesulfame-K on the glycaemic response to a glucose load’, The Plymouth Student Scientist, 13(1), p. 97-111.
27. Nikolakopoulou A, Higgins JPT, Papakonstantinou T, Chaimani A, Del Giovane C, Egger M, et al. CINeMA: An approach for assessing confidence in the results of a network meta-analysis. PLOS Medicine. 2020;17(4):e1003082.
28. Papakonstantinou T, Nikolakopoulou A, Higgins JPT, Egger M, Salanti G. CINeMA: Software for semiautomated assessment of the confidence in the results of network meta-analysis. Campbell Systematic Reviews. 2020;16(1):e1080.

29. Bureau of Nutritional Sciences, Food Directorate, Health Products and Food Branch. Summary of Health Canada's Assessment of a Health Claim about a Polysaccharide Complex (Glucomannan, Xanthan Gum, Sodium Alginate) and a Reduction of Post-Prandial Blood Glucose Response. 2016. Accessed at https://www.canada.ca/content/dam/hc-sc/migration/hc-sc/fn-an/alt_formats/pdf/label-etiquet/claims-reclam/assess-evalu/glucose-complex-polysaccharides-complexe-glycemique-eng.pdf on June 20, 2021.
30. Calanna S, Christensen M, Holst JJ, Laferrère B, Gluud LL, Vilsbøll T, et al. Secretion of glucagon-like peptide-1 in patients with type 2 diabetes mellitus: systematic review and meta-analyses of clinical studies. Diabetologia. 2013;56(5):965-72.
31. Calanna S, Christensen M, Holst JJ, Laferrère B, Gluud LL, Vilsbøll T, et al. Secretion of glucose-dependent insulinotropic polypeptide in patients with type 2 diabetes: systematic review and meta-analysis of clinical studies. Diabetes Care. 2013;36(10):3346-52.
32. Sievenpiper JL, Jenkins DJ, Josse RG, Vuksan V. Dilution of the 75-g oral glucose tolerance test increases postprandial glycemia: implications for diagnostic criteria. Cmaj. 2000;162(7):993-6.
33. Braunstein CR, Noronha JC, Glenn AJ, Viguiliouk E, Noseworthy R, Khan TA, et al. A Double-Blind, Randomized Controlled, Acute Feeding Equivalence Trial of Small, Catalytic Doses of Fructose and Allulose on Postprandial Blood Glucose Metabolism in Healthy Participants: The Fructose and Allulose Catalytic Effects (FACE) Trial. Nutrients. 2018;10(6).
34. Noronha JC, Braunstein CR, Glenn AJ, Khan TA, Viguiliouk E, Noseworthy R, et al. The effect of small doses of fructose and allulose on postprandial glucose metabolism in type 2 diabetes: A double-blind, randomized, controlled, acute feeding, equivalence trial. Diabetes Obes Metab. 2018;20(10):2361-70.
35. Schünemann H, Brożek J, Guyatt G, Oxman A, editors. GRADE handbook for grading quality of evidence and strength of recommendations. Updated October 2013. The GRADE Working Group, 2013. www.guidelinedevelopment.org/handbook.
36. Wolever TM, Jenkins DJ, Jenkins AL, Josse RG. The glycemic index: methodology and clinical implications. Am J Clin Nutr. 1991;54(5):846-54.
37. Chapter 2.5.5. Propagation of error considerations. NIST/SEMATECH e-Handbook of Statistical Methods. 2003. Accessed at https://www.itl.nist.gov/div898//handbook/mpc/section5/mpc55.htm#exactMay on 13 Sep 2021. [
